# The 12th Edition of the Scientific Days of the National Institute for Infectious Diseases “Prof. Dr. Matei Bals” and the 12th National Infectious Diseases Conference: Bucharest, Romania. 23–25 November 2016

**DOI:** 10.1186/s12879-016-1877-4

**Published:** 2016-11-01

**Authors:** Cristian-Mihail Niculae, Eliza Manea, Raluca Jipa, Simona Merisor, Ruxandra Moroti, Serban Benea, Adriana Hristea, Alina Cristina Neguț, Oana Săndulescu, Anca Streinu-Cercel, Dana Mărculescu, Magdalena Lorena Andrei, Veronica Ilie, Marcela Popa, Coralia Bleotu, Carmen Chifiriuc, Mircea Ioan Popa, Adrian Streinu-Cercel, Alina Orfanu, Cristina Popescu, Anca Leuștean, Remulus Catană, Anca Negru, Alexandra Badea, Radu Orfanu, Cătălin Tilișcan, Victoria Aramă, Ştefan Sorin Aramă, Constanța-Angelica Vișan, Anca-Cristina Drăgănescu, Anuța Bilașco, Camelia Kouris, Mădălina Merișescu, Magdalena Vasile, Diana-Maria Slavu, Sabina Vintilă, Endis Osman, Alina Oprea, Sabina Sandu, Monica Luminos, Alina Orfanu, Victoria Aramă, Ştefan Sorin Aramă, Anca Leuştean, Remulus Catană, Anca Negru, Gabriel Adrian Popescu, Cristina Popescu, Ramona Georgiana Stanculete, Ana Vaduva Enoiu, Adelina Raluca Marinescu, Voichita Lazureanu, Adelina-Raluca Marinescu, Alexandru Crișan, Voichița Lăzureanu, Virgil Musta, Narcisa Nicolescu, Ruxandra Laza, Anca-Ruxandra Negru, Daniela-Ioana Munteanu, Raluca Mihăilescu, Remulus Catană, Olga Dorobăț, Alexandru Rafila, Emilia Căpraru, Marius Niculescu, Rodica Marinescu, Olivera Lupescu, Vlad Predescu, Adrian Streinu-Cercel, Victoria Aramă, Daniela Tălăpan, Ramona Ștefania Popescu, Luminița Bradu, Dragoș Florea, Adrian Streinu-Cercel, Daniela Anicuta Leca, Elena Bunea, Andra Teodor, Egidia Miftode, Mădălina Merișescu, Gheorghiță Jugulete, Adrian Streinu-Cercel, Dragoș Florea, Monica Luminos, Ramona Ștefania Popescu, Anamaria Dobrotă, Adina Ilie, Liliana Lucia Preoțescu, Adriana Hristea, Raluca Jipa, Nicoleta Irimescu, Irina Panait, Eliza Manea, Simona Merisor, Cristian Niculae, Daniela Tălăpan, Liana Cătălina Gavriliu, Otilia Elisabeta Benea, Șerban Benea, Alexandru Rafila, Olga Dorobăț, Mona Popoiu, Livia Dragonu, Augustin Cupşa, Iulian Diaconescu, Irina Niculescu, Lucian Giubelan, Florentina Dumitrescu, Andreea Cristina Stoian, Camelia Guţă, Simona Puiu, Bunescu Irina, Marilyse Vallée, Ann Huletsky, Dominique K. Boudreau, Ève Bérubé, Richard Giroux, Jean Longtin, Yves Longtin, Michel G. Bergeron, Cleo Nicoleta Roșculeț, Dalila-Ana Toma, Catrinel Ciuca, Daniela Tălăpan, Cătălin Apostolescu, Andrei Rogoz, Andrei Stangaciu, Viorica Mitescu, Tudor Vladoiu, Doina Iovănescu, Michaela Oana, Simona Costin, Alina Cristina Neguț, Oana Săndulescu, Anca Streinu-Cercel, Maria Magdalena Moțoi, Mircea Ioan Popa, Adrian Streinu-Cercel, Daniela Tălăpan, Olga Mihaela Dorobăț, Mona Popoiu, Alexandru Mihai, Doina Iovănescu, Cleo Roşculeț, Cătălin Apostolescu, Gabriel-Adrian Popescu, Adrian Abagiu, Ruxandra Moroti-Constantinescu, Adriana Hristea, Victoria Aramă, Otilia Benea, Mădălina Simoiu, Rodica Bacruban, Adrian Streinu-Cercel, Alexandru Rafila, Olga Mihaela Dorobăț, Daniela Tălăpan, Alexandru Mihai, Ioana Bădicuț, Mona Popoiu, Alina Borcan, Alexandru Rafila, Gabriel Adrian Popescu, Mihnea Hurmuzache, Georgiana Enache, Alexandra Ciocan, Mircea Bararu, Madalina Popazu, Doina Viorica Iovănescu, Cleo Nicoleta Roșculeț, Andrei Rogoz, Cătălin Gabriel Apostolescu, Viorica Mitescu, Tudor Vladoiu, Dalila Toma, Catrinel Ciuca, Laura Iliescu, Georgiana Minzala, Letitia Toma, Mihaela Baciu, Alina Tanase, Carmen Orban, Victor Pantea, Gheorghe Placinta, Valentin Cebotarescu, Lilia Cojuhari, Paulina Jimbei, Cristina Popescu, Anca Leuștean, Cristina Dragomirescu, Alina Orfanu, Cristina Murariu, Laurențiu Stratan, Alexandra Badea, Cătălin Tilișcan, Daniela Munteanu, Raluca Năstase, Violeta Molagic, Mihaela Rădulescu, Remulus Catană, Victoria Aramă, Cristina Popescu, Laurențiu Stratan, Remulus Catană, Anca Leuștean, Cristina Dragomirescu, Alexandra Badea, Cristina Murariu, Raluca Năstase, Violeta Molagic, Daniela Munteanu, Cătălin Tilișcan, Mihaela Rădulescu, Alina Orfanu, Ioan Diaconu, Anca Negru, Iulia Bodosca, Violeta Niță, Victoria Aramă, Anca Leuștean, Victoria Aramă, Alina Orfanu, Remulus Catană, Laurențiu Stratan, Cristina Dragomirescu, Cristina Murariu, Alexandra Badea, Cătălin Tilișcan, Daniela Munteanu, Violeta Molagic, Raluca Năstase, Mihaela Rădulescu, Cristina Popescu, Cristina Popescu, Cristina Dragomirescu, Anca Leuștean, Cristina Murariu, Laurențiu Stratan, Alexandra Badea, Remulus Catană, Alina Orfanu, Raluca Mihaela Năstase, Violeta Molagic, Daniela Munteanu, Cătălin Tilișcan, Victoria Aramă, Victoria Aramă, Remulus Catană, Cristina Dragomirescu, Cristina Murariu, Anca Leuștean, Laurențiu Stratan, Alexandra Badea, Alina Orfanu, Anca Negru, Raluca Năstase, Violeta Molagic, Daniela Munteanu, Cătălin Tilișcan, Mihaela Rădulescu, Ioan Diaconu, Violeta Niță, Iulia Bodoșca, Cristina Popescu, Cristina Popescu, Alexandra Badea, Anca Leuștean, Alina Orfanu, Anca Negru, Laurențiu Stratan, Cristina Dragomirescu, Remulus Catană, Cristina Murariu, Violeta Molagic, Raluca Năstase, Cătălin Tilișcan, Daniela Munteanu, Mihaela Rădulescu, Ioan Diaconu, Violeta Niță, Iulia Bodoșca, Victoria Aramă, Cristina Popescu, Alina Orfanu, Anca Leuștean, Alexandra Badea, Laurențiu Stratan, Remulus Catană, Cătălin Tilișcan, Victoria Aramă, Cristina Popescu, Cristina Murariu, Cristina Dragomirescu, Anca Leuștean, Laurențiu Stratan, Alina Orfanu, Alexandra Badea, Remulus Catană, Anca Negru, Cătălin Tilișcan, Daniela Munteanu, Mihaela Rădulescu, Violeta Molagic, Raluca Mihaela Năstase, Ioan Alexandru Diaconu, Iulia Bodoșca, Violeta Niță, Victoria Aramă, Yagmur Erturk, Oana Săndulescu, Alina Cristina Neguț, Claudiu Mihai Șchiopu, Adrian Streinu-Cercel, Anca Streinu-Cercel, Violeta Molagic, Cătălin Tilișcan, Cristina Popescu, Raluca Mihăilescu, Daniela Munteanu, Raluca Năstase, Anca Negru, Angelica Tenita, Victoria Aramă, Ștefan Sorin Aramă, Simona Alexandra Iacob, Diana Gabriela Iacob, Monica Luminos, Anca Streinu-Cercel, Oana Săndulescu, Mioara Predescu, Alexandra Mărdărescu, Cătălin Tilișcan, Mihai Săndulescu, Claudiu Mihai Șchiopu, Adrian Streinu-Cercel, Cleo Nicoleta Roșculeț, Catrinel Olimpia Ciuca, Dalila Ana Toma, Cătălin Gabriel Apostolescu, Andrei Rogoz, Cristina Elena Mitu, Andrei Stangaciu, Viorica Daniela Mitescu, Tudor Gheorghe Vladoiu, Doina Viorica Iovănescu, Oana Săndulescu, Anca Streinu-Cercel, Monica Andreea Stoica, Liliana Lucia Preoțescu, Daniela Manolache, Gabriela Jana Ceapraga, Maria Magdalena Moțoi, Luminița Bradu, Adina Ilie, Gabriela Mircea, Ionel Durbală, Adrian Streinu-Cercel, Irina Russu, Tiberiu Holban, Tatiana Pantilimonov, Galina Chiriacov, Arcadie Macvovei, Elena Scorohodico, Oleg Dmitriev, Diana Alexandra Costache, Anca Benea, Eliza Manea, Cristian Niculae, Raluca Jipa, Adriana Hristea, Elisabeta Benea, Ruxandra Moroti, Șerban Benea, Mihai Mitran, Carmen Georgescu, Loredana Mitran, Simona Vladareanu, Andreea Ioana Magirescu, Viorica Andreev, Cristina Nicolau, Alexandra Largu, Carmen Dorobat, Carmen Manciuc, Viorica Andreev, Andreea Ioana Magirescu, Ina Isac, Cristina Nicolau, Alexandra Largu, Carmen Dorobat, Carmen Manciuc, Iulia Gabriela Șerban, Ghiulendan Resul, Consuela Marcaș, Iosif Marincu, Patricia Poptelecan, Bogdan Trincă, Sorina Mitrescu, Anca Tudor, Daliborca Vlad, Livius Tirnea, Nurcan Baydaroglu, Alina Cristina Neguț, Oana Săndulescu, Daniela Manolache, Gabriela Ceapraga, Monica Andreea Stoica, Anca Streinu-Cercel, Adrian Streinu-Cercel, Carmen Manciuc, Mariana Pagute, Cristina Nicolau, Carmen Dorobăț, Alexandra Largu, Ioan-Alexandru Diaconu, Laurențiu Stratan, Daniela Ion, Luciana Nichita, Cristina Popescu, Raluca Năstase, Daniela Munteanu, Violeta Molagic, Cătălin Tilișcan, Mihaela Rădulescu, Alexandra Diaconu, Anca Negru, Alina Orfanu, Cristina Dragomirescu, Remulus Catană, Anca Leuștean, Irina Duport-Dodot, Cristina Murariu, Iulia Bodoșca, Violeta Niță, Alexandra Badea, Victoria Aramă, Mariana Mărdărescu, Cristina Petre, Marieta Iancu, Rodica Ungurianu, Alina Cibea, Ruxandra Drăghicenoiu, Ana Maria Tudor, Delia Vlad, Sorin Petrea, Carina Matei, Dan Oțelea, Carmen Crăciun, Cristian Anghelina, Alexandra Mărdărescu, Elena Dumea, Adrian Streinu-Cercel, Sorin Rugină, Lucian Cristian Petcu, Stela Halichidis, Simona Claudia Cambrea, Carmen Chiriac, Nina-Ioana Bodnar, Iringo-Erzsebet Zaharia-Kezdi, Cristina Gîrbovan, Andrea Incze, Anca Meda Georgescu, Simona Alexandra Iacob, Diana Gabriela Iacob, Eugenia Panaitescu, Monica Luminos, Manole Cojocaru, Simona Alexandra Iacob, Diana Gabriela Iacob, Monica Luminos, Vochita Laurențiu, Vochita Andreia, Opreanu Radu, Trinca Bogdan, Rosca Ovidiu, Marincu Iosif, Ramona Zamfir, Alina Angelescu, Alena Andreea Popa, Raluca Jipa, Ruxandra Moroti, Adriana Hristea, Liana Gavriliu, Șerban Benea, Elisabeta Benea, Alena-Andreea Popa, Georgeta Ducu, Daniela Camburu, Alina Cozma, Manuela Podani, Roxana Dumitriu, Liana Gavriliu, Șerban Benea, Elisabeta Benea, Andreea Cristina Stoian, Florentina Dumitrescu, Augustin Cupșa, Lucian Giubelan, Irina Niculescu, Loredana Ionescu, Livia Dragonu, Adrian Octavian Abagiu, Loredana Nicoleta Stoica, Catrinel Blaga, Archontis Koulosousas, Roxana Ștefănescu, Alice Atomoaie, Florentina Paraschiv, Florin Matache Duna, Rodica Olteanu, Roxana Ion, Alexandra Zota, Isra Ennour Jaballah, Lara Mahfoud, Georgeta Preda, Magda Constantin, Ilinca Nicolae, Corina Daniela Ene, Mădălina Irina Mitran, Vasile Benea, Mircea Tampa, Simona Roxana Georgescu, Iulia Cristina Bodoșca, Cristina Murariu, Cătălin Tilișcan, Victoria Aramă, Cristina Popescu, Daniela Munteanu, Mihaela Rădulescu, Violeta Molagic, Raluca Năstase, Alina Orfanu, Anca Leuștean, Remulus Catană, Anca Negru, Adrian Streinu-Cercel, Sorin Aramă, Iuliana Caramăngiu, Ovidiu Rosca, Monica Cialma, Radu Opreanu, Laurențiu Vochita, Iosif Marincu, Vlad Murărescu, Marilena Palaghiță, Alina Cristina Neguț, Cornel Camburu, Adrian Streinu-Cercel, Irina Duşan, Patricia Poptelecan, Bogdan Trincă, Sorina Mitrescu, Livius Tirnea, Iosif Marincu, Narcisa Nicolescu, Alexandru Crișan, Voichița Lăzureanu, Ruxandra Laza, Virgil Musta, Adelina-Raluca Marinescu, Andreea Bîrlad, Victor Daniel Miron, Anca Cristina Drăgănescu, Constanța-Angelica Vișan, Anuța Bilașco, Daniela Pițigoi, Oana Săndulescu, Monica Luminița Luminos, Monica Luminos, Endis Osman, Magdalena Vasile, Anca Cristina Drăgănescu, Constanța-Angelica Vișan, Anuța Bilașco, Camelia Kouris, Sabina Șchiopu, Mădălina Merișescu, Monica Luminos, Anca Cristina Drăgănescu, Constanța-Angelica Vișan, Anuța Bilașco, Camelia Kouris, Endis Osman, Sabina Vintilă, Magda Vasile, Mădălina Merișescu, Liana Cătălina Gavriliu, Otilia Elisabeta Benea, Alina Angelescu, Ramona Zamfir, Daniela Camburu, Georgeta Ducu, Alina Cozma, Roxana Dumitriu, Manuela Podani, Șerban Benea, Mihaela Ionică, Gheorghiță Jugulete, Adina Stăncescu, Cristina Elena Popescu, Luminița Marin, Diana Zaharia, Cristina Dumitrescu, Lucia Tudor, Sabina Vintilă, Constanța-Angelica Vișan, Anca Cristina Drăgănescu, Anuța Bilașco, Magda Vasile, Mădălina Merișescu, Camelia Kouris, Cristina Negulescu, Endis Osman, Diana-Maria Slavu, Sabina Vintilă, Daniela Pițigoi, Monica Luminos, Olga Adriana Caliman-Sturdza, Cleo Roșculeț, Catrinel Olimpia Ciuca, Dalila Toma, Cătălin Apostolescu, Andrei Rogoz, Andrei Stangaciu, Viorica Mitescu, Doina Iovănescu, Cornel Camburu, Bogdana Manu, Ana Vaduva-Enoiu, Ramona Georgiana Stanculete, Adelina Raluca Marinescu, Voichita Elena Lazureanu, Elena-Violeta Niță, Sînziana Dumitru, Daniela-Ioana Munteanu, Anca Ruxandra Negru, Remulus Catană, Ioan Diaconu, Bogdana Manu, Ligia Ionescu, Liliana Ion, Cătălin Tilișcan, Victoria Aramă, Doina Viorica Iovănescu, Cleo Nicoleta Roșculeț, Andrei Rogoz, Cătălin Apostolescu, Viorica Mitescu, Tudor Vladoiu, Dalila Toma, Catrinel Ciuca, Iulia Gabriela Șerban, Marioara Neacșu, Simona Roxana Georgescu, Vasile Benea, Corina Daniela Ene, Mircea Tampa, Cristina Iulia Mitran, Ilinca Nicolae, George Ciprian Pribac, Mirandolina Prisca, Fulvia Ursoiu, Carmen Neamtu, Bogdan Totolici, Coralia Cotoraci, Aurel Ardelean, Simona Elena Albu, Mara Carsote, Beatrice Miclăuș, Diana Mihai, Oana Săndulescu, Cristina Vasiliu, Cristina Vasiliu, Mara Carsote, Corina Gorgoi, Beatrice Miclăuș, Diana Mihai, Oana Săndulescu, Simona Elena Albu, Amelia Blescun, Gelu Breaza, Sabina Vintila, Felicia Mihai, Meilin Omer, Cornel Dragan, Daniela Pitigoi, Mirela Ciucu, Marius-Dan Ionescu, Cristina Roskanovic, Valentina Barbu, Iulian Diaconescu, Florentina Dumitrescu, Irina Niculescu, Mihaela Ionică, Ramona-Alexandra Zamfir, Alina Cozma, Otilia Elisabeta Benea, Alexandra-Sînziana Dumitru, Daniela-Ioana Munteanu, Violeta Niță, Cristina Popescu, Iulia Bodosca, Angelica Tenita, Viorica Ispas, Victoria Aramă, Vasile Benea, Simona Roxana Georgescu, Mircea Tampa, Diana Oana Leahu, Cristina Maria Safta, Mihaela Anca Benea, Oana Săndulescu, Octavian Munteanu, Roxana Bohâlțea, Livia Trașcă, Monica Cîrstoiu, Doina Viorica Iovănescu, Cleo Nicoleta Roșculeț, Andrei Rogoz, Cătălin Gabriel Apostolescu, Viorica Daniela Mitescu, Tudor Gheorghe Vladoiu, Dalila Toma, Catrinel Ciuca, Mădălina Georgescu, Daniela Pițigoi, Alina Elena Ivanciuc, Mihaela Lazar, Teodora Ionescu, Carmen Maria Cherciu, Cristina Țecu, Maria Elena Mihai, Maria Nițescu, Rodica Bacruban, Delia Azamfire, Aura Dumitrescu, Elena Ianosik, Daniela Leca, Elena Duca, Andra Teodor, Codrina Bejan, Emanoil Ceaușu, Simin-Aysel Florescu, Corneliu Popescu, Grațiela Târdei, Codrina Juganariu, Emilia Lupulescu, Ligia Rodina, Maria Elena Cocuz, Gheorghiță Jugulete, Adina Stăncescu, Cristina Elena Popescu, Luminița Marin, Diana Zaharia, Cristina Dumitrescu, Endis Osman, Irina Niculescu, Augustin Cupșa, Iulian Diaconescu, Florentina Dumitrescu, Livia Dragonu, Andreea Stoian, Lucian Giubelan, Cristina Roskanovic, Ramona-Alexandra Zamfir, Mihaela Ionica, Otilia-Elisabeta Benea, Maria-Cristina Sîrbu, AnaMaria Dobrotă, Alina Cristina Neguț, Roxana Duda, Rodica Bacruban, Daniela Pițigoi, Cristiana Cerasella Dragomirescu, Daniela Tălăpan, Olga Dorobăț, Adrian Streinu-Cercel, Anca Streinu-Cercel, Mihaela Ionica, Ramona-Alexandra Zamfir, Alina Cozma, Otilia Elisabeta Benea, Sergiu Fendrihan, Ecaterina Scortan, Mircea Ioan Popa, Corneliu P. Popescu, Șerban N. Benea, Andra E. Petcu, Adriana Hristea, Adrian Abagiu, Iuliana A. Podea, Raluca E. Jipa, Georgeta Ducu, Raluca M. Hrișcă, Dragoș Florea, Manuela Nica, Eliza Manea, Simona Merișor, Cristian M. Nicolae, Simin A. Florescu, Irina M. Dumitru, Emanoil Ceaușu, Sorin Rugină, Ruxandra V. Moroti, Daniela Pițigoi, Teodora Ionescu, Oana Săndulescu, Maria Nițescu, Bogdan Nițescu, Iulia Monica Mustaţă, Sorina Claudia Boldeanu, Florentina Furtunescu, Adrian Streinu-Cercel, Diana Gabriela Iacob, Simona Alexandra Iacob, Mihaela Gheorghe, Adriana Slavcovici, Raluca Tripon, Roxana Iubu, Cristian Marcu, Mihaela Sabou, Monica Muntean, Ion Chiriac, Tiberiu Holban, Liviu Tazlavanu, Raluca Jipa, Eliza Manea, Roxana Cernat, Kezdi Iringo, Andrei Vâță, Manuela Arbune, Teodora Moisil, Adriana Hristea, Corina-Daniela Ene, Ilinca Nicolae, Roxana Simona Georgescu, Corina-Daniela Ene, Cosmin-Victor Ene, Roxana Simona Georgescu, Marilena Ciortea, Lucreția Dulgheru, Ilinca Nicolae, Mihaela Cătălina Luca, Ioana-Alina Harja-Alexa, Roxana Nemescu, Mădălina Popazu, Andrei Ștefan Luca, Gabriela Bancescu, Bogdan Dabu, Adrian Bancescu, Eliza Manea, Raluca Jipa, Adriana Hristea, Adina Elena Ilie, Săftica-Mariana Pohrib, Alina Cristina Neguț, Maria-Sabina Tache, Maria Magdalena Moțoi, Oana Săndulescu, Ion Aurel Iliescu, Adrian Streinu-Cercel, Cristina Tecu, Maria-Elena Mihai, Mihaela Lazăr, Carmen Cherciu, Alina Ivanciuc, Daniela Pițigoi, Emilia Lupulescu, Mirela Paliu, Manuela Curescu, Bianca Cerbu, Iosif Marincu, Maria Elena Mihai, Carmen Maria Cherciu, Alina Elena Ivanciuc, Cristina Tecu, Emilia Lupulescu, Irina Bunescu, Tiberiu Holban, Ana Pasnin, Stela Semeniuc, Raisa Popovici, Galina Chiriacov

**Affiliations:** 1 National Institute for Infectious Diseases “Prof. Dr. Matei Balș”, Bucharest, Romania; 2 Carol Davila University of Medicine and Pharmacy, Bucharest, Romania; 3 National Institute for Infectious Diseases “Prof. Dr. Matei Balș”, Bucharest, Romania; 4 Carol Davila University of Medicine and Pharmacy, Bucharest, Romania; 5 Research Institute of the University of Bucharest, Bucharest, Romania; 6 Microbiology Department, Faculty of Biology, University of Bucharest, Bucharest, Romania; 7 Ștefan S. Nicolau Institute of Virology, Romanian Academy, Bucharest, Romania; 8 National Institute for Infectious Diseases “Prof. Dr. Matei Balș”, Bucharest, Romania; 9 Carol Davila University of Medicine and Pharmacy, Bucharest, Romania; 10 “Foisor” Clinical Hospital of Orthopedics and Traumatology, Bucharest, Romania; 11 National Institute for Infectious Diseases “Prof. Dr. Matei Balș”, Bucharest, Romania; 12 Carol Davila University of Medicine and Pharmacy, Bucharest, Romania; 13 National Institute for Infectious Diseases “Prof. Dr. Matei Balș”, Bucharest, Romania; 14 Carol Davila University of Medicine and Pharmacy, Bucharest, Romania; 15 Dr. Victor Babeş Clinical Hospital of Infectious Diseases and Pneumology, Timişoara, Romania; 16 Dr. Victor Babeş University of Medicine and Pharmacy, Timişoara, Romania; 17 Dr. Victor Babeş University of Medicine and Pharmacy, Timişoara, Romania; 18 National Institute for Infectious Diseases “Prof. Dr. Matei Balș”, Bucharest, Romania; 19 Carol Davila University of Medicine and Pharmacy, Bucharest, Romania; 20 Clinical Hospital Colentina, Bucharest, Romania; 21 Floreasca Emergency Hospital, Bucharest, Romania; 22 “St. Pantelimon” Emergency Clinical Hospital, Bucharest, Romania; 23 Carol Davila University of Medicine and Pharmacy, Bucharest, Romania; 24 National Institute for Infectious Diseases “Prof. Dr. Matei Balș”, Bucharest, Romania; 25 “Gr.T.Popa” University of Medicine and Pharmacy, Iași, Romania; 26 Carol Davila University of Medicine and Pharmacy, Bucharest, Romania; 27 National Institute for Infectious Diseases “Prof. Dr. Matei Balș”, Bucharest, Romania; 28 Carol Davila University of Medicine and Pharmacy, Bucharest, Romania; 29 National Institute for Infectious Diseases “Prof. Dr. Matei Balș”, Bucharest, Romania; 30 National Institute for Infectious Diseases “Prof. Dr. Matei Balș”, Bucharest, Romania; 31 Carol Davila University of Medicine and Pharmacy, Bucharest, Romania; 32 Carol Davila University of Medicine and Pharmacy, Bucharest, Romania; 33 National Institute for Infectious Diseases “Prof. Dr. Matei Balș”, Bucharest, Romania; 34 University of Medicine and Pharmacy Craiova, Craiova, Romania; 35 “Victor Babeș” Clinical Hospital of Infectious Diseases and Pneumology, Craiova, Romania; 36 Department of Infectious Diseases and Medical Parasitology, Medical and Pharmaceutical University “Nicolae Testemițanu”, Chișinău, Republic of Moldova; 37 Research Center of Infectious Diseases of Laval University Québec, Québec, Canada; 38 Hospital Center of Laval University, Québec, Canada; 39 Cardiology and Pulmonology University Institute of Québec, Québec, Canada; 40 National Institute for Infectious Diseases “Prof. Dr. Matei Balș”, Bucharest, Romania; 41 “Dr. Constantin Papilian” Military and Emergency Hospital, Cluj-Napoca, Romania; 42 National Institute for Infectious Diseases “Prof. Dr. Matei Balș”, Bucharest, Romania; 43 Carol Davila University of Medicine and Pharmacy, Bucharest, Romania; 44 National Institute for Infectious Diseases “Prof. Dr. Matei Balș”, Bucharest, Romania; 45 Carol Davila University of Medicine and Pharmacy, Bucharest, Romania; 46 National Institute for Infectious Diseases “Prof. Dr. Matei Balș”, Bucharest, Romania; 47 Carol Davila University of Medicine and Pharmacy, Bucharest, Romania; 48 National Institute for Infectious Diseases “Prof. Dr. Matei Balș”, Bucharest, Romania; 49 Carol Davila University of Medicine and Pharmacy, Bucharest, Romania; 50 Infectious Diseases Hospital “Sf Parascheva”, Iași, Romania; 51 “Gr.T.Popa” University of Medicine and Pharmacy, Iași, Romania; 52 “N. Oblu” Clinical Hospital of Neurosurgery, Iaşi, Romania; 53 National Institute for Infectious Diseases “Prof. Dr. Matei Balș”, Bucharest, Romania; 54 Carol Davila University of Medicine and Pharmacy, Bucharest, Romania; 55 Fundeni Clinical Institute, Bucharest, Romania; 56 Faculty of Medicine, Nicolae Testemițanu State Medical and Pharmacy University, Chișinău, Republic of Moldova; 57 Toma Ciorbă Clinical Hospital for Infectious Diseases, Chișinău, Republic of Moldova; 58 National Institute for Infectious Diseases “Prof. Dr. Matei Balș”, Bucharest, Romania; 59 Carol Davila University of Medicine and Pharmacy, Bucharest, Romania; 60 National Institute for Infectious Diseases “Prof. Dr. Matei Balș”, Bucharest, Romania; 61 Carol Davila University of Medicine and Pharmacy, Bucharest, Romania; 62 National Institute for Infectious Diseases “Prof. Dr. Matei Balș”, Bucharest, Romania; 63 Carol Davila University of Medicine and Pharmacy, Bucharest, Romania; 64 National Institute for Infectious Diseases “Prof. Dr. Matei Balș”, Bucharest, Romania; 65 Carol Davila University of Medicine and Pharmacy, Bucharest, Romania; 66 Carol Davila University of Medicine and Pharmacy, Bucharest, Romania; 67 National Institute for Infectious Diseases “Prof. Dr. Matei Balș”, Bucharest, Romania; 68 National Institute for Infectious Diseases “Prof. Dr. Matei Balș”, Bucharest, Romania; 69 Carol Davila University of Medicine and Pharmacy, Bucharest, Romania; 70 National Institute for Infectious Diseases “Prof. Dr. Matei Balș”, Bucharest, Romania; 71 Carol Davila University of Medicine and Pharmacy, Bucharest, Romania; 72 National Institute for Infectious Diseases “Prof. Dr. Matei Balș”, Bucharest, Romania; 73 Carol Davila University of Medicine and Pharmacy, Bucharest, Romania; 74 Carol Davila University of Medicine and Pharmacy, Bucharest, Romania; 75 National Institute for Infectious Diseases “Prof. Dr. Matei Balș”, Bucharest, Romania; 76 National Institute for Infectious Diseases “Prof. Dr. Matei Balș”, Bucharest, Romania; 77 Carol Davila University of Medicine and Pharmacy, Bucharest, Romania; 78 National Institute for Infectious Diseases “Prof. Dr. Matei Balș”, Bucharest, Romania; 79 Carol Davila University of Medicine and Pharmacy, Bucharest, Romania; 80 National Institute for Infectious Diseases “Prof. Dr. Matei Balș”, Bucharest, Romania; 81 Regional HIV/AIDS Center INBI “Prof. Dr. Matei Balș”, Bucharest, Romania; 82 National Institute for Infectious Diseases “Prof. Dr. Matei Balș”, Bucharest, Romania; 83 Colentina Clinical Hospital, Bucharest, Romania; 84 Carol Davila University of Medicine and Pharmacy, Bucharest, Romania; 85 National Institute for Infectious Diseases “Prof. Dr. Matei Balș”, Bucharest, Romania; 86 Department of Infectious Diseases, Parasitology and Tropical Medicine, Nicolae Testemițanu State Medical and Pharmacy University, Chișinău, Republic of Moldova; 87 Toma Ciorbă Clinical Hospital for Infectious Diseases, Chișinău, Republic of Moldova; 88 National Institute for Infectious Diseases “Prof. Dr. Matei Balș”, Bucharest, Romania; 89 Carol Davila University of Medicine and Pharmacy, Bucharest, Romania; 90 Clinical Hospital of Infectious and Tropical Diseases “Dr. Victor Babeș”, Bucharest, Romania; 91 Clinical Hospital of Obstetrics and Gynecology “Prof. Dr. Panait Sârbu”, Bucharest, Romania; 92 Carol Davila University of Medicine and Pharmacy, Bucharest, Romania; 93 Elias University Emergency Hospital, Bucharest, Romania; 94 Infectious Diseases Hospital “Sf Parascheva”, Iași, Romania; 95 “Gr.T.Popa” University of Medicine and Pharmacy, Iași, Romania; 96 “Gr.T.Popa” University of Medicine and Pharmacy, Iași, Romania; 97 Infectious Diseases Hospital “Sf Parascheva”, Iași, Romania; 98 Clinical Infectious Diseases Hospital of Constanța, Constanța, Romania; 99 Dr. Victor Babeş University of Medicine and Pharmacy, Timişoara, Romania; 100 Dr. Victor Babeş Clinical Hospital of Infectious Diseases and Pneumology, Timişoara, Romania; 101 Carol Davila University of Medicine and Pharmacy, Bucharest, Romania; 102 National Institute for Infectious Diseases “Prof. Dr. Matei Balș”, Bucharest, Romania; 103 Infectious Diseases Hospital “Sf Parascheva”, Iași, Romania; 104 “Gr.T.Popa” University of Medicine and Pharmacy, Iași, Romania; 105 Emergency Hospital for Children “St. Maria”, Iași, Romania; 106 National Institute for Infectious Diseases “Prof. Dr. Matei Balș”, Bucharest, Romania; 107 Carol Davila University of Medicine and Pharmacy, Bucharest, Romania; 108 “Prof. Marius Nasta” National Institute for Pneumology, Bucharest, Romania; 109 National Institute for Infectious Diseases “Prof. Dr. Matei Balș”, Bucharest, Romania; 110 Faculty of Medicine, “Ovidius” University, Constanța, Romania; 111 Clinical Infectious Diseases Hospital of Constanța, Constanța, Romania; 112 Carol Davila University of Medicine and Pharmacy, Bucharest, Romania; 113 Faculty of Dental Medicine, Ovidius University, Constanța, Romania; 114 University of Medicine and Pharmacy Tîrgu Mureș, Tîrgu Mureș, Romania; 115 National Institute for Infectious Diseases “Prof. Dr. Matei Balș”, Bucharest, Romania; 116 Carol Davila University of Medicine and Pharmacy, Bucharest, Romania; 117 Titu Maiorescu University, Bucharest, Romania; 118 National Institute for Infectious Diseases “Prof. Dr. Matei Balș”, Bucharest, Romania; 119 Dr. Victor Babeş Clinical Hospital of Infectious Diseases and Pneumology, Timişoara, Romania; 120 National Institute for Infectious Diseases “Prof. Dr. Matei Balș”, Bucharest, Romania; 121 Carol Davila University of Medicine and Pharmacy, Bucharest, Romania; 122 National Institute for Infectious Diseases “Prof. Dr. Matei Balș”, Bucharest, Romania; 123 Carol Davila University of Medicine and Pharmacy, Bucharest, Romania; 124 University of Medicine and Pharmacy from Craiova, Craiova, Romania; 125 National Institute for Infectious Diseases “Prof. Dr. Matei Balș”, Bucharest, Romania; 126 Romanian Anti-AIDS Association (ARAS), Bucharest, Romania; 127 Dermatology Department, Colentina Clinical Hospital, Bucharest, Romania; 128 National Institute for Infectious Diseases “Prof. Dr. Matei Balș”, Bucharest, Romania; 129 Clinical Hospital of Infectious and Tropical Diseases “Dr. Victor Babeș”, Bucharest, Romania; 130 Clinical Hospital of Nephrology, “Dr. Carol Davila”, Bucharest, Romania; 131 Carol Davila University of Medicine and Pharmacy, Bucharest, Romania; 132 National Institute for Infectious Diseases “Prof. Dr. Matei Balș”, Bucharest, Romania; 133 Carol Davila University of Medicine and Pharmacy, Bucharest, Romania; 134 Dr. Victor Babeş Clinical Hospital of Infectious Diseases and Pneumology, Timişoara, Romania; 135 National Institute for Infectious Diseases “Prof. Dr. Matei Balș”, Bucharest, Romania; 136 Carol Davila University of Medicine and Pharmacy, Bucharest, Romania; 137 Department of Infectious Diseases, Pneumology and Parasitology, Dr. Victor Babeş Clinical Hospital of Infectious Diseases and Pneumology, Timişoara, Romania; 138 Dr. Victor Babeş University of Medicine and Pharmacy, Timişoara, Romania; 139 Dr. Victor Babeş Clinical Hospital of Infectious Diseases and Pneumology, Timişoara, Romania; 140 Carol Davila University of Medicine and Pharmacy, Bucharest, Romania; 141 National Institute for Infectious Diseases “Prof. Dr. Matei Balș”, Bucharest, Romania; 142 Carol Davila University of Medicine and Pharmacy, Bucharest, Romania; 143 National Institute for Infectious Diseases “Prof. Dr. Matei Balș”, Bucharest, Romania; 144 Carol Davila University of Medicine and Pharmacy, Bucharest, Romania; 145 National Institute for Infectious Diseases “Prof. Dr. Matei Balș”, Bucharest, Romania; 146 Carol Davila University of Medicine and Pharmacy, Bucharest, Romania; 147 National Institute for Infectious Diseases “Prof. Dr. Matei Balș”, Bucharest, Romania; 148 National Institute for Infectious Diseases “Prof. Dr. Matei Balș”, Bucharest, Romania; 149 Carol Davila University of Medicine and Pharmacy, Bucharest, Romania; 150 National Institute for Infectious Diseases “Prof. Dr. Matei Balș”, Bucharest, Romania; 151 Carol Davila University of Medicine and Pharmacy, Bucharest, Romania; 152 Emergency County Hospital “New St. John”, Suceava, Romania; 153 National Institute for Infectious Diseases “Prof. Dr. Matei Balș”, Bucharest, Romania; 154 Dr. Victor Babeş Clinical Hospital of Infectious Diseases and Pneumology, Timişoara, Romania; 155 Dr. Victor Babeş University of Medicine and Pharmacy, Timişoara, Romania; 156 National Institute for Infectious Diseases “Prof. Dr. Matei Balș”, Bucharest, Romania; 157 Carol Davila University of Medicine and Pharmacy, Bucharest, Romania; 158 National Institute for Infectious Diseases “Prof. Dr. Matei Balș”, Bucharest, Romania; 159 Carol Davila University of Medicine and Pharmacy, Bucharest, Romania; 160 Clinical Infectious Diseases Hospital of Constanța, Constanța, Romania; 161 Carol Davila University of Medicine and Pharmacy, Bucharest, Romania; 162 Clinical Hospital of Infectious and Tropical Diseases “Dr. Victor Babeș”, Bucharest, Romania; 163 Clinical Hospital of Nephrology, “Dr. Carol Davila”, Bucharest, Romania; 164 Vasile Goldiș Western University, Arad, Romania; 165 Clinical Emergency County Hospital, Arad, Romania; 166 Carol Davila University of Medicine and Pharmacy, Bucharest, Romania; 167 University Emergency Hospital of Bucharest, Bucharest, Romania; 168 Carol Davila University of Medicine and Pharmacy, Bucharest, Romania; 169 University Emergency Hospital of Bucharest, Bucharest, Romania; 170 “Dr. Teodor Andrei” Lugoj County Hospital, Lugoj, Romania; 171 National Institute for Infectious Diseases “Prof. Dr. Matei Balș”, Bucharest, Romania; 172 Colentina Clinical Hospital, Bucharest, Romania; 173 Carol Davila University of Medicine and Pharmacy, Bucharest, Romania; 174 “Victor Babeș” Clinical Hospital of Infectious Diseases and Pneumology, Craiova, Romania; 175 University of Medicine and Pharmacy, Craiova, Romania; 176 National Institute for Infectious Diseases “Prof. Dr. Matei Balș”, Bucharest, Romania; 177 Carol Davila University of Medicine and Pharmacy, Bucharest, Romania; 178 The National Institute for Infectious Diseases “Prof. Dr. Matei Balș”, Bucharest, Romania; 179 Carol Davila University of Medicine and Pharmacy, Bucharest, Romania; 180 Clinical Hospital of Infectious and Tropical Diseases “Dr. Victor Babeș”, Bucharest, Romania; 181 Carol Davila University of Medicine and Pharmacy, Bucharest, Romania; 182 University Emergency Hospital of Bucharest, Bucharest, Romania; 183 National Institute for Infectious Diseases “Prof. Dr. Matei Balș”, Bucharest, Romania; 184 Carol Davila University of Medicine and Pharmacy, Bucharest, Romania; 185 Carol Davila University of Medicine and Pharmacy, Bucharest, Romania; 186 Carol Davila University of Medicine and Pharmacy, Bucharest, Romania; 187 National Institute for Infectious Diseases “Prof. Dr. Matei Balș”, Bucharest, Romania; 188 National Institute for Research Cantacuzino, Bucharest, Romania; 189 National Institute of Endocrinology “C. I. Parhon”, Bucharest, Romania; 190 “Gr.T.Popa” University of Medicine and Pharmacy, Iași, Romania; 191 Public Health Authority, Iași, Romania; 192 Clinical Hospital of Infectious and Tropical Diseases “Dr. Victor Babeș”, Bucharest, Romania; 193 Infectious Diseases Hospital “Sf Parascheva”, Iași, Romania; 194 Infectious Diseases Hospital, Brașov, Romania; 195 Faculty of Medicine, Transylvania University, Brașov, Romania; 196 Carol Davila University of Medicine and Pharmacy, Bucharest, Romania; 197 National Institute for Infectious Diseases “Prof. Dr. Matei Balș”, Bucharest, Romania; 198 University of Medicine and Pharmacy Craiova, Craiova, Romania; 199 “Victor Babeș” Clinical Hospital of Infectious Diseases and Pneumology, Craiova, Romania; 200 National Institute for Infectious Diseases “Prof. Dr. Matei Balș”, Bucharest, Romania; 201 Carol Davila University of Medicine and Pharmacy, Bucharest, Romania; 202 National Institute for Infectious Diseases “Prof. Dr. Matei Balș”, Bucharest, Romania; 203 Carol Davila University of Medicine and Pharmacy, Bucharest, Romania; 204 National Institute of Research “Cantacuzino”, Bucharest, Romania; 205 National Institute for Infectious Diseases “Prof. Dr. Matei Balș”, Bucharest, Romania; 206 Carol Davila University of Medicine and Pharmacy, Bucharest, Romania; 207 Carol Davila University of Medicine and Pharmacy, Bucharest, Romania; 208 “Cantacuzino” National Research Institute, Bucharest, Romania; 209 Vasile Goldiș Western University, Arad, Romania; 210 IMS Health Romania, Bucharest, Romania; 211 Carol Davila University of Medicine and Pharmacy, Bucharest, Romania; 212 Clinical Hospital of Infectious and Tropical Diseases “Dr. Victor Babeș”, Bucharest, Romania; 213 National Institute for Infectious Diseases “Prof. Dr. Matei Balș”, Bucharest, Romania; 214 Faculty of Medicine, “Ovidius” University, Constanța, Romania; 215 Clinical Infectious Diseases Hospital of Constanța, Constanța, Romania; 216 Emergency County Hospital, Pitești, Romania; 217 Central Universitary Emergency Military Hospital Dr Carol Davila, Bucharest, Romania; 218 Carol Davila University of Medicine and Pharmacy, Bucharest, Romania; 219 National Institute for Infectious Diseases “Prof. Dr. Matei Balș”, Bucharest, Romania; 220 Infection Control Department, National Institute of Endocrinology “C. I. Parhon”, Bucharest, Romania; 221 Public Health Authority, Bucharest, Romania; 222 National Institute for Infectious Diseases “Prof. Dr. Matei Balș”, Bucharest, Romania; 223 Department of Infectious Diseases, Iuliu Hațieganu University of Medicine and Pharmacy, Cluj-Napoca, Romania; 224 Teaching Hospital of Infectious Diseases, Cluj-Napoca, Romania; 225 Clinical Hospital of Infectious Diseases, Chișinău, Republic of Moldova; 226 State University of Medicine and Pharmacy, “Nicolae Testemițanu”, Chișinău, Republic of Moldova; 227 National Institute for Infectious Diseases “Prof. Dr. Matei Balș”, Bucharest, Romania; 228 Clinical Infectious Diseases Hospital of Constanța, Constanța, Romania; 229 Clinical County Hospital Mureș, Tîrgu Mureș, Romania; 230 Infectious Diseases Hospital “Sf Parascheva”, Iași, Romania; 231 Infectious Diseases Hospital “Sfanta Paracheva”, Galați, Romania; 232 Dr. Victor Babeş Clinical Hospital of Infectious Diseases and Pneumology, Timişoara, Romania; 233 Carol Davila Clinical Hospital of Nephrology, Bucharest, Romania; 234 Clinical Hospital of Infectious and Tropical Diseases “Dr. Victor Babeș”, Bucharest, Romania; 235 Carol Davila University of Medicine and Pharmacy, Bucharest, Romania; 236 Carol Davila Clinical Hospital of Nephrology, Bucharest, Romania; 237 Carol Davila University of Medicine and Pharmacy, Bucharest, Romania; 238 Saint John Clinical Hospital of Emergency, Bucharest, Romania; 239 “Victor Babeș” Clinical Hospital of Infectious Diseases and Pneumology, Craiova, Romania; 240 Victor Babeș Center of Diagnosis and Treatment, Bucharest, Romania; 241 “Gr.T.Popa” University of Medicine and Pharmacy, Iași, Romania; 242 Infectious Diseases Hospital “Sf Parascheva”, Iași, Romania; 243 Carol Davila University of Medicine and Pharmacy, Bucharest, Romania; 244 National Institute for Infectious Diseases “Prof. Dr. Matei Balș”, Bucharest, Romania; 245 Carol Davila University of Medicine and Pharmacy, Bucharest, Romania; 246 National Institute for Infectious Diseases “Prof. Dr. Matei Balș”, Bucharest, Romania; 247 Carol Davila University of Medicine and Pharmacy, Bucharest, Romania; 248 University Emergency Hospital of Bucharest, Bucharest, Romania; 249 Cantacuzino National Institute of Research, Bucharest, Romania; 250 Carol Davila University of Medicine and Pharmacy, Bucharest, Romania; 251 Dr. Victor Babeş Clinical Hospital of Infectious Diseases and Pneumology, Timişoara, Romania; 252 Cantacuzino National Institute for Research, Bucharest, Romania; 253 Department of Infectious Diseases and Medical Parasitology, Medical and Pharmaceutical University “Nicolae Testemițanu”, Chișinău, Republic of Moldova; 254 Department of Laboratory Medicine, Medical and Pharmaceutical University “Nicolae Testemițanu”, Chișinău, Republic of Moldova; 255 Republican Clinical Hospital of Infectious Diseases “Toma Ciorbă”, Chișinău, Republic of Moldova

## Abstract

A1 The outcome of patients with recurrent versus non-recurrent pneumococcal meningitis in a tertiary health-care hospital in Bucharest

Cristian-Mihail Niculae, Eliza Manea, Raluca Jipa, Simona Merisor, Ruxandra Moroti, Serban Benea, Adriana Hristea

A2 Influence of bacteriophages on sessile Gram-positive and Gram-negative bacteria

Alina Cristina Neguț, Oana Săndulescu, Anca Streinu-Cercel, Dana Mărculescu, Magdalena Lorena Andrei, Veronica Ilie, Marcela Popa, Coralia Bleotu, Carmen Chifiriuc, Mircea Ioan Popa, Adrian Streinu-Cercel

A3 The utility of inflammatory biomarkers in the prognostic evaluation of septic patients – past, present and future

Alina Orfanu, Cristina Popescu, Anca Leuștean, Remulus Catană, Anca Negru, Alexandra Badea, Radu Orfanu, Cătălin Tilișcan, Victoria Aramă, Ştefan Sorin Aramă

A4 Etiologic and clinical features of bacterial meningitis in infants

Constanța-Angelica Vișan, Anca-Cristina Drăgănescu, Anuța Bilașco, Camelia Kouris, Mădălina Merișescu, Magdalena Vasile, Diana-Maria Slavu, Sabina Vintilă, Endis Osman, Alina Oprea, Sabina Sandu, Monica Luminos

A5 The diagnostic and prognostic role of neutrophil to lymphocyte count ratio in sepsis

Alina Orfanu, Victoria Aramă, Ştefan Sorin Aramă, Anca Leuştean, Remulus Catană, Anca Negru, Gabriel Adrian Popescu, Cristina Popescu

A6 Whooping cough in a HIV positive patient

Ramona Georgiana Stanculete, Ana Vaduva Enoiu, Adelina Raluca Marinescu, Voichita Lazureanu

A7 *Cronobacter sakazakii* sepsis in varicella patient

Adelina-Raluca Marinescu, Alexandru Crișan, Voichița Lăzureanu, Virgil Musta, Narcisa Nicolescu, Ruxandra Laza

A8 Anaerobes an underdiagnosed cause of prosthesis joint infection

Anca-Ruxandra Negru, Daniela-Ioana Munteanu, Raluca Mihăilescu, Remulus Catană, Olga Dorobăț, Alexandru Rafila, Emilia Căpraru, Marius Niculescu, Rodica Marinescu, Olivera Lupescu, Vlad Predescu, Adrian Streinu-Cercel, Victoria Aramă, Daniela Tălăpan

A9 *Streptococcus pneumoniae* meningitis presenting with normal CSF – case presentation

Ramona Ștefania Popescu, Luminița Bradu, Dragoș Florea, Adrian Streinu-Cercel

A10 Extrapulmonary manifestations of infection with *Mycoplasma pneumoniae* – study on 24 cases

Daniela Anicuta Leca, Elena Bunea, Andra Teodor, Egidia Miftode

A11 The molecular diagnosis of severe bacterial sepsis in pediatric population

Mădălina Merișescu, Gheorghiță Jugulete, Adrian Streinu-Cercel, Dragoș Florea, Monica Luminos

A12 Acute *Staphylococcus aureus* endocarditis with multiple septic complications in a patient with diabetes mellitus – case presentation

Ramona Ștefania Popescu, Anamaria Dobrotă, Adina Ilie, Liliana Lucia Preoțescu

A13 Is *Streptococcus suis* meningitis an under-diagnosed zoonosis?

Adriana Hristea, Raluca Jipa, Nicoleta Irimescu, Irina Panait, Eliza Manea, Simona Merisor, Cristian Niculae, Daniela Tălăpan

A14 *Klebsiella pneumoniae* isolated from blood. Antimicrobial resistance – past and present

Liana Cătălina Gavriliu, Otilia Elisabeta Benea, Șerban Benea, Alexandru Rafila, Olga Dorobăț, Mona Popoiu

A15 Antibiotics resistance in *Staphylococcus aureus* isolated from blood cultures

Livia Dragonu, Augustin Cupşa, Iulian Diaconescu, Irina Niculescu, Lucian Giubelan, Florentina Dumitrescu, Andreea Cristina Stoian, Camelia Guţă, Simona Puiu

A16 Predominance of CTX-M enzymes in extended-spectrum β-lactamase-producing Enterobacteriaceae in two hospitals of Quebec City

Bunescu Irina, Marilyse Vallée, Ann Huletsky, Dominique K. Boudreau, Ève Bérubé, Richard Giroux, Jean Longtin, Yves Longtin, Michel G. Bergeron

A17 Postoperative meningoencephalitis with *Acinetobacter baumannii* XDR – a therapeutic challenge - Case report

Cleo Nicoleta Roșculeț, Dalila-Ana Toma, Catrinel Ciuca, Daniela Tălăpan, Cătălin Apostolescu, Andrei Rogoz, Andrei Stangaciu, Viorica Mitescu, Tudor Vladoiu, Doina Iovănescu

A18 Septic arthritis with *Burkholderia cepacia*

Michaela Oana, Simona Costin

A19 A novel approach for managing hard-to-treat infections

Alina Cristina Neguț, Oana Săndulescu, Anca Streinu-Cercel, Maria Magdalena Moțoi, Mircea Ioan Popa, Adrian Streinu-Cercel

A20 Nineteen months surveillance for multidrug resistant organisms (MDRO) by detecting asymptomatic colonization

Daniela Tălăpan, Olga Mihaela Dorobăț, Mona Popoiu, Alexandru Mihai, Doina Iovănescu, Cleo Roşculeț, Cătălin Apostolescu, Gabriel-Adrian Popescu, Adrian Abagiu, Ruxandra Moroti-Constantinescu, Adriana Hristea, Victoria Aramă, Otilia Benea, Mădălina Simoiu, Rodica Bacruban, Adrian Streinu-Cercel, Alexandru Rafila

A21 Antimicrobial resistance of Gram-positive cocci isolated from clinical specimens in the National Institute of Infectious Diseases “Prof Dr. Matei Balș” between 2009–2015

Olga Mihaela Dorobăț, Daniela Tălăpan, Alexandru Mihai, Ioana Bădicuț, Mona Popoiu, Alina Borcan, Alexandru Rafila

A22 The high percentage of carbapenem-resistant Gram-negative bacilli in Romania: an analysis and some proposals

Gabriel Adrian Popescu

A23 Etiological, clinical and therapeutic considerations on 78 cases of healthcare associated meningitis or ventriculitis admitted in the “Sf. Parascheva” infectious diseases clinical hospital, Iași, from 2011 to 2015

Mihnea Hurmuzache, Georgiana Enache, Alexandra Ciocan, Mircea Bararu, Madalina Popazu

A24 Nosocomial infection dynamics in an Intensive Care Department – an overview (epidemiological and clinical monitoring, advanced therapeutic intervention).

Doina Viorica Iovănescu, Cleo Nicoleta Roșculeț, Andrei Rogoz Cătălin Gabriel Apostolescu, Viorica Mitescu_,_ Tudor Vladoiu, Dalila Toma, Catrinel Ciuca

A25 Safety and efficacy of interferon free treatment in patients with HCV chronic hepatitis- experience of a single Internal Medicine center

Laura Iliescu, Georgiana Minzala, Letitia Toma, Mihaela Baciu, Alina Tanase, Carmen Orban

A26 Viusid in treatment of chronic viral hepatitis B and C

Victor Pantea, Gheorghe Placinta, Valentin Cebotarescu, Lilia Cojuhari, Paulina Jimbei

A27 The management of hyperbilirubinemia in HCV cirrhotic patients who underwent therapy with direct acting antivirals

Cristina Popescu, Anca Leuștean, Cristina Dragomirescu, Alina Orfanu, Cristina Murariu, Laurențiu Stratan, Alexandra Badea, Cătălin Tilișcan, Daniela Munteanu, Raluca Năstase, Violeta Molagic, Mihaela Rădulescu, Remulus Catana, Victoria Aramă

A28 The efficacy of ombitasvir-paritaprevir/ritonavir, dasabuvir and ribavirin in patients with genotype 1 HCV compensated cirrhosis

Cristina Popescu, Laurențiu Stratan, Remulus Catana, Anca Leuștean, Cristina Dragomirescu, Alexandra Badea, Cristina Murariu, Raluca Năstase, Violeta Molagic, Daniela Munteanu, Cătălin Tilișcan, Mihaela Rădulescu, Alina Orfanu, Ioan Diaconu, Anca Negru, Iulia Bodosca, Violeta Niță, Victoria Aramă

A29 The efficacy of direct acting antivirals regimen without ribavirin in HCV genotype 1b infected patients with compensated cirrhosis

Anca Leuștean, Victoria Aramă, Alina Orfanu, Remulus Catana, Laurențiu Stratan, Cristina Dragomirescu, Cristina Murariu, Alexandra Badea, Cătălin Tilișcan, Daniela Munteanu, Violeta Molagic, Raluca Năstase, Mihaela Rădulescu, Cristina Popescu

A30 Liver decompensation during ombitasvir-paritaprevir/ritonavir-dasabuvir and ribavirin regimen in HCV infected patients with Child-Pugh A cirrhosis

Cristina Popescu, Cristina Dragomirescu, Anca Leuștean, Cristina Murariu, Laurențiu Stratan, Alexandra Badea, Remulus Catană, Alina Orfanu, Raluca Mihaela Năstase, Violeta Molagic, Daniela Munteanu, Cătălin Tilișcan, Victoria Aramă

A31 The safety of direct acting antivirals in HCV compensated cirrhotic patients - an interim analysis

Victoria Aramă, Remulus Catană, Cristina Dragomirescu, Cristina Murariu, Anca Leuștean, Laurențiu Stratan, Alexandra Badea, Alina Orfanu, Anca Negru, Raluca Năstase, Violeta Molagic, Daniela Munteanu, Cătălin Tilișcan, Mihaela Rădulescu, Ioan Diaconu, Violeta Niță, Iulia Bodoșca, Cristina Popescu

A32 The access of patients with HCV compensated cirrhosis to the National Program of therapy with direct acting antivirals

Cristina Popescu, Alexandra Badea, Anca Leuștean, Alina Orfanu, Anca Negru, Laurențiu Stratan, Cristina Dragomirescu, Remulus Catană, Cristina Murariu, Violeta Molagic, Raluca Năstase, Cătălin Tilișcan, Daniela Munteanu, Mihaela Rădulescu, Ioan Diaconu, Violeta Niță, Iulia Bodoșca, Victoria Aramă

A33 Severe reactivation of chronic hepatitis B after discontinuation of nucleos(t)ide analogues – a case series

Cristina Popescu, Alina Orfanu, Anca Leuștean, Alexandra Badea, Laurențiu Stratan, Remulus Catană, Cătălin Tilișcan, Victoria Aramă

A34 The dynamic of hematological disorders during direct acting antivirals therapy for HCV compensated cirrhosis

Cristina Popescu, Cristina Murariu, Cristina Dragomirescu, Anca Leuștean, Laurențiu Stratan, Alina Orfanu, Alexandra Badea, Remulus Catană, Anca Negru, Cătălin Tilișcan, Daniela Munteanu, Mihaela Rădulescu, Violeta Molagic, Raluca Mihaela Năstase, Ioan Alexandru Diaconu, Iulia Bodoșca, Violeta Niță, Victoria Aramă

A35 Behaviors, attitudes and risk factors for viral hepatitis in international medical students vs. the general population in Romania

Yagmur Erturk, Oana Săndulescu, Alina Cristina Neguț, Claudiu Mihai Șchiopu, Adrian Streinu-Cercel, Anca Streinu-Cercel

A36 Characteristics of hepatitis C virus reactivation due to immunosuppressive therapy in Romanian HCV infected patients with hematological malignancies

Violeta Molagic, Cătălin Tilișcan, Cristina Popescu, Raluca Mihăilescu, Daniela Munteanu, Raluca Năstase, Anca Negru, Angelica Tenita, Victoria Aramă, Ștefan Sorin Aramă

A37 The dynamic IFN-gamma serum levels during successful peginterferon-a 2a/ribavirin therapy in HCV chronic infection

Simona Alexandra Iacob, Diana Gabriela Iacob, Monica Luminos

A38 Overlapping risk factors for transmission of HBV, HCV and HIV in the general population in Romania

Anca Streinu-Cercel, Oana Săndulescu, Mioara Predescu, Alexandra Mărdărescu, Cătălin Tilișcan, Mihai Săndulescu, Claudiu Mihai Șchiopu, Adrian Streinu-Cercel

A39 Acute hepatitis - an uncommon neurological complication

Cleo Nicoleta Roșculeț, Catrinel Olimpia Ciuca, Dalila Ana Toma, Cătălin Gabriel Apostolescu, Andrei Rogoz, Cristina Elena Mitu, Andrei Stangaciu, Viorica Daniela Mitescu, Tudor Gheorghe Vladoiu, Doina Viorica Iovănescu

A40 Regression of liver fibrosis following sustained virological response in patients with chronic HCV infection and cirrhosis

Oana Săndulescu, Anca Streinu-Cercel, Monica Andreea Stoica, Liliana Lucia Preoțescu, Daniela Manolache, Gabriela Jana Ceapraga, Maria Magdalena Moțoi, Luminița Bradu, Adina Ilie, Gabriela Mircea, Ionel Durbală, Adrian Streinu-Cercel

A41 Preliminary results of treatment with sofosbuvir and daclatasvir of patients with chronic hepatitis C

Irina Russu, Tiberiu Holban, Tatiana Pantilimonov, Galina Chiriacov, Arcadie Macvovei, Elena Scorohodico, Oleg Dmitriev

A42 HIV-syphilis coinfection

Diana Alexandra Costache, Anca Benea, Eliza Manea, Cristian Niculae, Raluca Jipa, Adriana Hristea, Elisabeta Benea, Ruxandra Moroti, Șerban Benea

A43 Thrombophilia – additional risk factor for the evolution of pregnancy in HIV-positive patients

Mihai Mitran, Carmen Georgescu, Loredana Mitran, Simona Vladareanu

A44 The incidence of oropharyngeal candidiasis in hospitalized HIV infected pediatric Romanian cohort between 1 January - 31 December 2015

Andreea Ioana Magirescu, Viorica Andreev, Cristina Nicolau, Alexandra Largu, Carmen Dorobat, Carmen Manciuc

A45 TB incidence in HIV infected patients during the year of 2015

Viorica Andreev, Andreea Ioana Magirescu, Ina Isac, Cristina Nicolau, Alexandra Largu, Carmen Dorobat, Carmen Manciuc

A46 Retrospective analysis of HIV/AIDS deaths recorded in the Clinical Infectious Diseases Hospital, Constanța in the period 01 January 2014–30 June 2016. Epidemiological considerations.

Iulia Gabriela Șerban, Ghiulendan Resul, Consuela Marcaș

A47 Acute liver failure with favorable evolution in an HIV-HBV coinfected patient

Iosif Marincu, Patricia Poptelecan, Bogdan Trincă, Sorina Mitrescu, Anca Tudor, Daliborca Vlad, Livius Tirnea

A48 Lifestyle impact on HIV management

Nurcan Baydaroglu, Alina Cristina Neguț, Oana Săndulescu, Daniela Manolache, Gabriela Ceapraga, Monica Andreea Stoica, Anca Streinu-Cercel, Adrian Streinu-Cercel

49. HIV positive mothers newborns - clinical experience from January 2012 to June 2016

Carmen Manciuc, Mariana Pagute, Cristina Nicolau, Carmen Dorobăț, Alexandra Largu

A50 Rediscovering HIV-associated progressive multifocal leukoencephalopathy and HIV encephalopathy: clinical suspicion and subsequent brain autopsies

Ioan-Alexandru Diaconu, Laurențiu Stratan, Daniela Ion, Luciana Nichita, Cristina Popescu, Raluca Năstase, Daniela Munteanu, Violeta Molagic, Cătălin Tilișcan, Mihaela Rădulescu, Alexandra Diaconu, Anca Negru, Alina Orfanu, Cristina Dragomirescu, Remulus Catană, Anca Leuștean, Irina Duport-Dodot, Cristina Murariu, Iulia Bodoșca, Violeta Niță, Alexandra Badea, Victoria Aramă

A51 Antenatal surveillance of pregnant women with risk behavior and its impact on mother-to-child HIV transmission in Romania

Mariana Mărdărescu, Cristina Petre, Marieta Iancu, Rodica Ungurianu, Alina Cibea, Ruxandra Drăghicenoiu, Ana Maria Tudor, Delia Vlad, Sorin Petrea, Carina Matei, Dan Oțelea, Carmen Crăciun, Cristian Anghelina, Alexandra Mărdărescu

A52 Noninvasive assessments (APRI, Fib-4, transient elastography) of fibrosis in patients with HIV and HIV/HBV infection

Elena Dumea, Adrian Streinu-Cercel, Sorin Rugină, Lucian Cristian Petcu, Stela Halichidis, Simona Claudia Cambrea

A53 Undetectable HIV viral load – the main goal in the management of HIV-infected patients

Carmen Chiriac, Nina-Ioana Bodnar, Iringo-Erzsebet Zaharia-Kezdi, Cristina Gîrbovan, Andrea Incze, Anca Meda Georgescu

A54 LPS serum levels and correlation with immunological, virological and clinical outcome in HIV infected patients

Simona Alexandra Iacob, Diana Gabriela Iacob, Eugenia Panaitescu, Monica Luminos, Manole Cojocaru

A55 LL37 human cathelicidin serum levels are positively correlated with IFN gamma and alanine aminotransferase level in HCV infection

Simona Alexandra Iacob, Diana Gabriela Iacob, Monica Luminos

A56 Early diagnosis of pulmonary tuberculosis in a non-compliant HIV/AIDS late presenter patient

Vochita Laurențiu, Vochita Andreia, Opreanu Radu, Trinca Bogdan, Rosca Ovidiu, Marincu Iosif

A57 Evolution of antiretroviral regimens in naϊve patients in 2016

Ramona Zamfir, Alina Angelescu, Alena Andreea Popa, Raluca Jipa, Ruxandra Moroti, Adriana Hristea, Liana Gavriliu, Șerban Benea, Elisabeta Benea

A58 The unfavorable risk factors for HIV infected persons with positive blood cultures hospitalized at the National Institute for Infectious Diseases “Prof. Dr. Matei Balș” in 2015

Alena-Andreea Popa, Georgeta Ducu, Daniela Camburu, Alina Cozma, Manuela Podani, Roxana Dumitriu, Liana Gavriliu, Șerban Benea, Elisabeta Benea

A59 Epidemiological aspects of HIV infection in Oltenia region

Andreea Cristina Stoian, Florentina Dumitrescu, Augustin Cupșa, Lucian Giubelan, Irina Niculescu, Loredana Ionescu, Livia Dragonu

A60 HIV risk behaviors and prevalence among patients in methadone maintenance therapy (MMT) from Arena center, Bucharest

Adrian Octavian Abagiu, Loredana Nicoleta Stoica, Catrinel Blaga, Archontis Koulosousas, Roxana Ștefănescu, Alice Atomoaie, Florentina Paraschiv, Florin Matache Duna

A61 Therapeutic options in a case of severe psoriasis associated with both HIV infection and hepatitis C virus previously treated with fumaric acid esters

Rodica Olteanu, Roxana Ion, Alexandra Zota, Isra Ennour Jaballah, Lara Mahfoud, Georgeta Preda, Magda Constantin

A62 Prevalence of autoantibodies against gangliosides in asymptomatic HIV-infected patients

Ilinca Nicolae, Corina Daniela Ene, Mădălina Irina Mitran, Vasile Benea, Mircea Tampa, Simona Roxana Georgescu

A63 Subclinical inflammation in HIV-infected patients undergoing antiretroviral therapy – a cross sectional study

Iulia Cristina Bodoșca, Cristina Murariu, Cătălin Tilișcan, Victoria Aramă, Cristina Popescu, Daniela Munteanu, Mihaela Rădulescu, Violeta Molagic, Raluca Năstase, Alina Orfanu, Anca Leuștean, Remulus Catană, Anca Negru, Adrian Streinu-Cercel, Sorin Aramă

A64 Severe Guillain-Barré syndrome occurring after chickenpox with favorable evolution

Iuliana CAramăngiu, Ovidiu Rosca, Monica Cialma, Radu Opreanu, Laurențiu Vochita, Iosif Marincu

A65 Echovirus 30 infection with pulmonary and cardiac complications – case report

Vlad Murărescu, Marilena Palaghiță, Alina Cristina Neguț, Cornel Camburu, Adrian Streinu-Cercel

A66 Herpetic encephalitis with favorable evolution in an adult immunocompetent patient

Irina Duşan, Patricia Poptelecan, Bogdan Trincă, Sorina Mitrescu, Livius Tirnea, Iosif Marincu

A67 Clinical-evolutional aspects in present-day measles

Narcisa Nicolescu, Alexandru Crișan, Voichița Lăzureanu, Ruxandra Laza, Virgil Musta, Adelina-Raluca Marinescu, Andreea Bîrlad

A68 Pneumococcal superinfection in children with influenza

Victor Daniel Miron, Anca Cristina Drăgănescu, Constanța-Angelica Vișan, Anuța Bilașco, Daniela Pițigoi, Oana Săndulescu, Monica Luminița Luminos

A69 Varicella complicated with transverse myelitis - case presentation

Monica Luminos, Endis Osman, Magdalena Vasile, Anca Cristina Drăgănescu, Constanța-Angelica Vișan, Anuța Bilașco, Camelia Kouris, Sabina Șchiopu, Mădălina Merișescu

A70 Clinical forms of enterovirus infections during the summer season of 2016

Monica Luminos, Anca Cristina Drăgănescu, Constanța-Angelica Vișan, Anuța Bilașco, Camelia Kouris, Endis Osman, Sabina Vintilă, Magda Vasile, Mădălina Merișescu

A71 Face off – HIV and lymphoma – case series presentation

Liana Cătălina Gavriliu, Otilia Elisabeta Benea, Alina Angelescu, Ramona Zamfir, Daniela Camburu, Georgeta Ducu, Alina Cozma, Roxana Dumitriu, Manuela Podani, Șerban Benea, Mihaela Ionică

A72 Coxsackie infection complicated by pancytopenia – pediatric case report

Gheorghiță Jugulete, Adina Stăncescu, Cristina Elena Popescu, Luminița Marin, Diana Zaharia, Cristina Dumitrescu, Lucia Tudor, Sabina Vintilă

A73 Viral respiratory infections in children in the season 2015–2016

Constanța-Angelica Vișan, Anca Cristina Drăgănescu, Anuța Bilașco, Magda Vasile, Mădălina Merișescu, Camelia Kouris, Cristina Negulescu, Endis Osman, Diana-Maria Slavu, Sabina Vintilă, Daniela Pițigoi, Monica Luminos

A75 The severity of A H1N1 Influenza infection in the 2015–2016 season

Cleo Roșculeț, Catrinel Olimpia Ciuca, Dalila Toma, Cătălin Apostolescu, Andrei Rogoz, Andrei Stangaciu, Viorica Mitescu, Doina Iovănescu, Cornel Camburu, Bogdana Manu

A76 Acute respiratory distress syndrome in a child with measles

Ana Vaduva-Enoiu, Ramona Georgiana Stanculete, Adelina Raluca Marinescu, Voichita Elena Lazureanu

A77 Management challenges of right-sided infectious endocarditis in an HIV positive patient – case presentation

Elena-Violeta Niță, Sînziana Dumitru, Daniela-Ioana Munteanu, Anca Ruxandra Negru, Remulus Catană, Ioan Diaconu, Bogdana Manu, Ligia Ionescu, Liliana Ion, Cătălin Tilișcan, Victoria Aramă

A78 Bacterial infection in critical patients with severe A H1N1 influenza virus infection (epidemiology, development, therapeutic decisions)

Doina Viorica Iovănescu, Cleo Nicoleta Roșculeț, Andrei Rogoz, Cătălin Apostolescu, Viorica Mitescu, Tudor Vladoiu, Dalila Toma, Catrinel Ciuca

A79 Epidemiological aspects of severe acute respiratory infection cases (SARI) in the season 2015–2016, in the Clinical Hospital of Infectious Diseases – Constanța, Romania

Iulia Gabriela Șerban, Marioara Neacșu

A80Overexpression of IL-6 trans signaling pathway in viral infections

Simona Roxana Georgescu, Vasile Benea, Corina Daniela Ene, Mircea Tampa, Cristina Iulia Mitran, Ilinca Nicolae

A81 Acute viral hepatitis B with persistent HBsAg – description and evolution

George Ciprian Pribac, Mirandolina Prisca, Fulvia Ursoiu, Carmen Neamtu, Bogdan Totolici, Coralia Cotoraci, Aurel Ardelean

A82 Prevalence of cervical pathogens in a population of pregnant female patients monitored in a tertiary care hospital in Bucharest, Romania

Simona Elena Albu, Mara Carsote, Beatrice Miclăuș, Diana Mihai, Oana Săndulescu, Cristina Vasiliu

A83 Prevalence of group B *Streptococcus* during pregnancy in a cohort of patients monitored in a tertiary care hospital in Bucharest, Romania

Cristina Vasiliu, Mara Carsote, Corina Gorgoi, Beatrice Miclăuș, Diana Mihai, Oana Săndulescu, Simona Elena Albu

A84 Infectious hematoma in the gastrocnemius muscle – case presentation

Amelia Blescun, Gelu Breaza

A85 Reflections towards the underexplored HTLV Romanian viral circulation - adult T‐cell leukemia/lymphomas, a case series

Sabina Vintila, Felicia Mihai, Meilin Omer, Cornel Dragan, Daniela Pitigoi

A86 A febrile confusion syndrome with acute onset – case presentation

Mirela Ciucu, Marius-Dan Ionescu, Cristina Roskanovic, Valentina Barbu, Iulian Diaconescu, Florentina Dumitrescu, Irina Niculescu

A87 Retrobulbar optic neuritis in a HIV-positive patient - case report

Mihaela Ionică, Ramona-Alexandra Zamfir, Alina Cozma, Otilia Elisabeta Benea

A88 A rare presentation of Q fever – case presentation

Alexandra-Sînziana Dumitru, Daniela-Ioana Munteanu, Violeta Niță, Cristina Popescu, Iulia Bodosca, Angelica Tenita, Viorica Ispas, Victoria Aramă

A89 Tinea incognita – case presentation

Vasile Benea, Simona Roxana Georgescu, Mircea Tampa, Diana Oana Leahu, Cristina Maria Safta, Mihaela Anca Benea

A90 Incidence and risk factors associated with TORCH infections during pregnancy

Oana Săndulescu, Octavian Munteanu, Roxana Bohâlțea, Livia Trașcă, Monica Cîrstoiu

A91 Acute respiratory failure in critical patients with sepsis

Doina Viorica Iovănescu, Cleo Nicoleta Roșculeț, Andrei Rogoz, Cătălin Gabriel Apostolescu, Viorica Daniela Mitescu, Tudor Gheorghe Vladoiu, Dalila Toma, Catrinel Ciuca

A92 Cochleo-vestibular deficit secondary to *Granulicatella elegans* meningitis

Mădălina Georgescu

A93 Influenza 2015/2016 – clinical, epidemiological and virological characteristics of cases admitted in three infectious diseases hospitals

Daniela Pițigoi, Alina Elena Ivanciuc, Mihaela Lazar, Teodora Ionescu, Carmen Maria Cherciu, Cristina Țecu, Maria Elena Mihai, Maria Nițescu, Rodica Bacruban, Delia Azamfire, Aura Dumitrescu, Elena Ianosik, Daniela Leca, Elena Duca, Andra Teodor, Codrina Bejan, Emanoil Ceaușu, Simin-Aysel Florescu, Corneliu Popescu, Grațiela Târdei, Codrina Juganariu, Emilia Lupulescu

A94 Severe complications of varicella requiring hospitalization in previously healthy children in Brașov county

Ligia Rodina, Maria Elena Cocuz

A95 Clinical forms of *Clostridium difficile* colitis in children

Gheorghiță Jugulete, Adina Stăncescu, Cristina Elena Popescu, Luminița Marin, Diana Zaharia, Cristina Dumitrescu, Endis Osman

A96 Community-acquired pneumonia – demographic, clinical and etiological aspects

Irina Niculescu, Augustin Cupșa, Iulian Diaconescu, Florentina Dumitrescu, Livia Dragonu, Andreea Stoian, Lucian Giubelan, Cristina Roskanovic

A97 Acute myocarditis in an adult patient with chickenpox - Case report

Ramona-Alexandra Zamfir, Mihaela Ionica, Otilia-Elisabeta Benea

A98 Caustic oropharyngeal wound with acute group F streptococcal superinfection mimicking diphtheria – case report and differential diagnosis

Maria-Cristina Sîrbu, AnaMaria Dobrotă, Alina Cristina Neguț, Roxana Duda, Rodica Bacruban, Daniela Pițigoi, Cristiana Cerasella Dragomirescu, Daniela Tălăpan, Olga Dorobăț, Adrian Streinu-Cercel, Anca Streinu-Cercel

A99 *Clostridium difficile* infection in HIV-positive patients admitted in the National Institute for Infectious Diseases “Prof. Dr. Matei Balș” in 2015

Mihaela Ionica, Ramona-Alexandra Zamfir, Alina Cozma, Otilia Elisabeta Benea

A100 Title: Epidemiology of *Candida* oral infections (stomatitis) in Romania

Sergiu Fendrihan, Ecaterina Scortan, Mircea Ioan Popa

A101 Anthrax case series in south-eastern Romania

Corneliu P Popescu, Șerban N Benea, Andra E Petcu, Adriana Hristea, Adrian Abagiu, Iuliana A Podea, Raluca E Jipa, Georgeta Ducu, Raluca M Hrișcă, Dragoș Florea, Manuela Nica, Eliza Manea, Simona Merișor, Cristian M Nicolae, Simin A Florescu, Irina M Dumitru, Emanoil Ceaușu, Sorin Rugină, Ruxandra V Moroti

A102 Knowledge, risk perception and attitudes of healthcare workers at the National Institute for Infectious Diseases “Prof. Dr. Matei Balș” regarding Ebola

Daniela Pițigoi, Teodora Ionescu, Oana Săndulescu, Maria Nițescu, Bogdan Nițescu, Iulia Monica Mustaţă, Sorina Claudia Boldeanu, Florentina Furtunescu, Adrian Streinu-Cercel

A103 A case of abdominopelvic actinomycosis with successful short-term antibiotic treatment

Diana Gabriela Iacob, Simona Alexandra Iacob, Mihaela Gheorghe

A104 A case of pneumonia caused by *Raoultella planticola*

Iulian Diaconescu, Irina Niculescu, Floretina Dumitrescu, Lucian Giubelan

A105 Vitamin D deficiency and sepsis in childhood

Adriana Slavcovici, Raluca Tripon, Roxana Iubu, Cristian Marcu, Mihaela Sabou, Monica Muntean

A106 The clinical and epidemiological aspects and prophylaxis of Lyme disease among patients who presented with tick bites to the Clinical Infectious Disease Hospital “Toma Ciorbă”

Ion Chiriac, Tiberiu Holban, Liviu Tazlavanu

A107 Drug-resistant tuberculosis in HIV infected patients

Raluca Jipa, Eliza Manea, Roxana Cernat, Kezdi Iringo, Andrei Vâță, Manuela Arbune, Teodora Moisil, Adriana Hristea

A108 Kidney injury molecule-1 and urinary tract infections

Corina-Daniela Ene, Ilinca Nicolae, Roxana Simona Georgescu

A109 The impact of microbiological agents on serum gangliosides in patients with benign prostate hyperplasia

Corina-Daniela Ene, Cosmin-Victor Ene, Roxana Simona Georgescu, Marilena Ciortea , Lucreția Dulgheru, Ilinca Nicolae

A110 Toxocariasis - the experience of the Iași Infectious Diseases Hospital between 2013–2015

Mihaela Cătălina Luca, Ioana-Alina Harja-Alexa, Roxana Nemescu, Mădălina Popazu, Andrei Ștefan Luca

A111 Species of anaerobic Gram-positive cocci involved in odontogenic abscesses

Gabriela Bancescu, Bogdan Dabu, Adrian Bancescu

A112 *Clostridium difficile* infection recurrences

Eliza Manea, Raluca Jipa, Adriana Hristea

A113 Differential diagnosis of staphylococcal and tuberculous osteodiscitis – case report

Adina Elena Ilie, Săftica-Mariana Pohrib, Alina Cristina Neguț, Maria-Sabina Tache, Maria Magdalena Moțoi, Oana Săndulescu, Ion Aurel Iliescu, Adrian Streinu-Cercel

A114 Severe clinical forms of respiratory syncytial virus infections

Cristina Tecu, Maria-Elena Mihai, Mihaela Lazăr, Carmen Cherciu, Alina Ivanciuc, Daniela Pițigoi, Emilia Lupulescu

A115 *Acinetobacter baumannii* postoperative sepsis associated with *Clostridium difficile* enterocolitis in an immune suppressed elderly patient

Mirela Paliu, Manuela Curescu, Bianca Cerbu, Iosif Marincu

A116 Risk factors and their impact on psychopathology and quality of life among people living with HIV/AIDS in Romania

Fulvia Ursoiu, Mirandolina Prișcă, George Ciprian Pribac

A117 Antivirals susceptibility of influenza viruses circulating in Romania

Maria Elena Mihai, Carmen Maria Cherciu, Alina Elena Ivanciuc, Cristina Tecu, Emilia Lupulescu

A118 Retrospective study of hospitalized cases of sepsis at the Hospital Clinic of Infectious Diseases “Toma Ciorbă”

Irina Bunescu, Tiberiu Holban, Ana Pasnin, Stela Semeniuc, Raisa Popovici, Galina Chiriacov

## Pathogenesis of bacterial infections

### A1 The outcome of patients with recurrent versus non-recurrent pneumococcal meningitis in a tertiary health-care hospital in Bucharest

#### Cristian-Mihail Niculae^1^, Eliza Manea^1^, Raluca Jipa^1,2^, Simona Merisor^1^, Ruxandra Moroti^1,2^, Serban Benea^1,2^, Adriana Hristea^1,2^

##### ^1^National Institute for Infectious Diseases “Prof. Dr. Matei Balș”, Bucharest, Romania; ^2^Carol Davila University of Medicine and Pharmacy, Bucharest, Romania

###### **Correspondence:** Cristian-Mihail Niculae (niculae_cristian87@yahoo.com)

**Background**

Pneumococcal meningitis (PM) is a life-threatening disease. Recurrent PM is relatively rare and associated with predisposing conditions. This study analyzed the outcome of patients with recurrent versus non-recurrent PM.

**Methods**

We conducted a retrospective study, analyzing the records of all the patients hospitalized between 2005 and 2015 for PM in our institution. We included patients with the diagnostic of PM based on appearance on Gram stain, a positive latex agglutination reaction of cerebral spinal fluid (CSF) samples and/or a positive culture for *Streptococcus pneumoniae* (CSF culture or blood culture and concomitant meningitis). We defined “recurrent meningitis” as at least two episodes of meningitis, separated by a period of at least 4 weeks.

**Results**

We identified a total of 194 PM episodes in 182 patients. Thirty eight (20 %) patients were diagnosed with recurrent meningitis, and they had 93 prior episodes recorded. The majority of patients with recurrent meningitis experienced two meningitis episodes, but we found 3 patients with 7, 11 and 40 recurrent episodes. Nineteen (50 %) patients in recurrent meningitis group and 90 (58 %) in non-recurrent group were men. Median age of patients with recurrent versus non-recurrent meningitis was 29 versus 57 years (p < 0.001). Nine (24 %) patients with recurrent meningitis versus 64 (41 %) patients with non-recurrent meningitis had an underlying immunosuppressive condition (p = 0.02, OR:0.41, 95 % CI:0.18–0.92). The immunosuppression was: diabetes mellitus in 32 (44 %), alcoholism in 19 (26 %), end-stage liver disease in 7 (9.5 %) and malignancy, malnutrition, pregnancy, splenectomy and immunosuppressive therapy in 15 (20.5 %). We found dissemination of infection from a contiguous site in 74 (38 %) cases, bone defects and/or CSF leakage in 36 (18.5 %) cases and hematogenous spread in 14 (7.2 %) cases. A cranial bone discontinuity and/or CSF leakage were identified in recurrent versus non-recurrent meningitis in 21 (55 %) versus 15 (10 %) episodes (p < 0.001, OR:14.41, 95 % CI:6.21–33.4). Hematogenous spread was observed only in the non-recurrent meningitis group. In recurrent versus non-recurrent meningitis, impaired consciousness on admission was noted in 15 (40 %) versus 64 (41 %). We noted one death (2.6 %) among patients with recurrent PM group vs 42 (27 %) in the non-recurrent group (p<0.001, OR:0.07, 95 % CI:0.01–0.5). Death was associated with non-recurrent meningitis (p < 0.001), contiguous spread (p = 0.014), immunodepression (p = 0.01) and impaired consciousness (p = 0.005).

**Conclusions**

Patients with recurrent meningitis were younger, with less immunosuppression conditions and had a better survival versus those with non-recurrent meningitis. Mortality of PM was associated with immunosuppression and impaired consciousness, but the small number of deaths in the recurrent group did not allow us to analyses the differences between the two groups.

### A2 Influence of bacteriophages on sessile Gram-positive and Gram-negative bacteria

#### Alina Cristina Neguț^1,2^, Oana Săndulescu^1,2^, Anca Streinu-Cercel^1,2^, Dana Mărculescu^1^, Magdalena Lorena Andrei^1^, Veronica Ilie^1^, Marcela Popa^3^, Coralia Bleotu^5^, Carmen Chifiriuc^3,4^, Mircea Ioan Popa^2^, Adrian Streinu-Cercel^1,2^

##### ^1^National Institute for Infectious Diseases “Prof. Dr. Matei Balș”, Bucharest, Romania; ^2^Carol Davila University of Medicine and Pharmacy, Bucharest, Romania; ^3^Research Institute of the University of Bucharest, Bucharest, Romania; ^4^Microbiology Department, Faculty of Biology, University of Bucharest, Bucharest, Romania; ^5^Ștefan S. Nicolau Institute of Virology, Romanian Academy, Bucharest, Romania

###### **Correspondence:** Alina Cristina Neguț (negoitza_alina@yahoo.com)

**Background**

Bacteriophages are hypothesized to contribute to biofilm eradication through multiple actions, such as: 1) amplification in host cells; 2) production of depolymerizing enzymes that degrade the matrix of extracellular polymeric substances; 3) induction of depolymerizing enzymes in host cells; 4) infection of persistent bacterial cells and lysis upon reactivation [1].

**Methods**

We have performed an in vitro experiment in the National Institute for Infectious Diseases „Prof. Dr. Matei Balș”, assessing the influence on biofilm formation and biofilm eradication of commercially-available bacteriophage cocktails Intesti and Pyo (Eliava BioPreparations, Tbilisi, Georgia) in different binary dilutions (1/2 through 1/64) as presented in Neguț et al., GERMS 2014. We have used two bacterial models: Gram-positive model (117 strains of *Staphylococcus* spp.) and Gram-negative model (44 strains of *Pseudomonas aeruginosa*) [2].

**Results**

Intesti and Pyo bacteriophages inhibited biofilm formation of both Gram-positive (p = 0.004, p = 0.001) and Gram-negative (p = 0.001, p = 0.001) models for all the dilutions used, respectively. The median optical density (OD) of staphylococcal culture decreased with 8.114 % (−9.386 %, 16.533 %) for the dilution 1/64 of Intesti, and 8.108 % (0.684 %, 20.143 %) for Pyo, respectively. The median OD decreased with 29.191 % (4.607 %, 48.448 %) for the dilution 1/2 of Intesti, and 35.555 % (6.122 %, 47.852 %) for Pyo, respectively. For the Gram-negative model, the OD decreased with 20.554 % (6.542 %, 44.814 %) for 1/64 Intesti and with 15.817 % (2.711 %, 38.082 %) for 1/64 Pyo dilution. The OD decreased with 40.806 % (10.582 %, 64.275 %) for 1/2 Intesti and 35.101 % (5.445 %, 48.742 %) for 1/2 Pyo dilution, respectively. The decrease in biofilm density as measured by the rate of change (ROC) was higher for the Gram-negative model compared with the Gram-positive model, but statistical significance was ascertained only for Intesti (p < 0.001, Z = −3.712 at 1/64 dilution, and borderline significance at 1/2 dilution: p = 0.050, Z = −1.959). Eradication of preformed biofilm was achieved in both models through the addition of phages: Intesti and Pyo for *Staphylococcus* spp. (p < 0.001, p = 0.009) and Intesti for *Pseudomonas aeruginosa* (p = 0.011).

**Conclusions**

By extrapolating our findings we hypothesize that phages may eventually represent an option for primary prophylaxis through coating of orthopedic prostheses and for secondary prophylaxis, by preventing osteomyelitis relapses through biofilm reduction when administered via local instillations.

**References**

1. Harper D, Parracho H, Walker J, et al. Bacteriophages and biofilms. Antibiotics. 2014;3:270–284

2. Neguț AC, Săndulescu O, Streinu-Cercel A, et al. Bacteriophage-driven inhibition of biofilm in *Pseudomonas aeruginosa*. Paper presented at ASM Microbe 2016, Boston, USA,

**Acknowledgement**

Carol Davila University of Medicine and Pharmacy, Young Researchers Grant 28341/2013; PhD thesis of ACN.

### A3 The utility of inflammatory biomarkers in the prognostic evaluation of septic patients – past, present and future

#### Alina Orfanu^1,2^, Cristina Popescu^1,2^, Anca Leuștean^1^, Remulus Catană^1,2^, Anca Negru^1,2^, Alexandra Badea^1^, Radu Orfanu^3^, Cătălin Tilișcan^1,2^, Victoria Aramă^1,2^, Ştefan Sorin Aramă^2^

##### ^1^National Institute for Infectious Diseases “Prof. Dr. Matei Balș”, Bucharest, Romania; ^2^Carol Davila University of Medicine and Pharmacy, Bucharest, Romania; ^3^“Foisor” Clinical Hospital of Orthopedics and Traumatology, Bucharest, Romania

###### **Correspondence:** Alina Orfanu (alina.lobodan@yahoo.com)

**Background**

Sepsis is a severe, life-threatening syndrome with high incidence and mortality rate, which needs a rapid diagnosis and severity evaluation in order to avoid fatal complications. If the diagnosis has preset criteria (SOFA score more than 2), the prognosis remains an important problem in septic patients management. Over the years, several markers of inflammation were evaluated such as fibrinogen, C reactive protein or procalcitonin (PCT). Objectives: to appreciate the efficacy of neutrophil/lymphocyte ratio (NLR), mean platelet volume (MPV), red cell distribution width (RDW) and PCT in sepsis prognosis and to correlate their values with the severity and with the estimated mortality rate.

**Methods**

Prospective study realized in the Matei Balș Institute between October 2015 – July 2016, including 55 patients diagnosed with sepsis. There were evaluated: number of SIRS criteria, primary sites of infection, incriminated germs, organ failures, septic metastases and there were calculated two scores of severity - APACHE (Acute Physiology and Chronic Health Evaluation), APS (Admission Point Score) and the estimated mortality rate. The statistical analysis was realized using SPSS.

**Results**

The study included 55 patients with a mean age of 57.9 years old and a sex ratio M:F = 1:1.75. The primary septic focus was found in 92.7 % of cases: respiratory (43.1 %), digestive (31.4 %), urinary (21.6 %), others (3.9 %). The most frequent isolated germs were *E. coli* (46.7 %), *Streptococcus pneumoniae* (20 %), *Clostridium difficile* (13.3 %), others including *Klebsiella*, *Legionella*, *Neisseria meningitidis* (20 %). 3.6 % of patients had septic metastases (cerebral or cutaneous) and 67.3 % presented at least one organ failure: hematological (57.3 %), renal (21.8 %), respiratory (5.5 %). The mean value of initial NLR was 17.7 and it was statistically significant correlated with the number of organ failures, with APACHE (p = 0.01) and APS (p = 0.01) scores and with the mortality rate (p = 0.01). This last correlation was stronger than the one between PCT with the mortality. VPM (mean value of 8.1 fl) and RDW (14.5) did not correlate with the two scores. PCT with a median of 14.58 ng/mL was correlated with APACHE (p = 0.02), APS (p = 0.05) and mortality rate (p = 0.05).

**Conclusions**

Septic patients’ outcome can be easily appreciated using NLR, which proved an important prognostic role in sepsis through the statistically correlations with the severity scores and the estimated mortality rate. PCT also remains a good test to appreciate the severity. Although some studies showed the importance of VPM and RDW in sepsis, these markers weren’t associated with the prognosis.

### A4 Etiologic and clinical features of bacterial meningitis in infants

#### Constanța-Angelica Vișan^1,2^, Anca-Cristina Drăgănescu^1^, Anuța Bilașco^1^, Camelia Kouris^1^, Mădălina Merișescu^1^, Magdalena Vasile^1^, Diana-Maria Slavu^1^, Sabina Vintilă^1^, Endis Osman^1^, Alina Oprea^1^, Sabina Sandu^1^, Monica Luminos^1,2^

##### ^1^National Institute for Infectious Diseases “Prof. Dr. Matei Balș”, Bucharest, Romania; ^2^Carol Davila University of Medicine and Pharmacy, Bucharest, Romania

###### **Correspondence:** Constanța-Angelica Vișan (angelica.visan@yahoo.com)

**Background**

Neonatal bacterial meningitis is a rare disease, but characterized by particularly high severity. Group B *Streptococcus* is the leading cause of neonatal meningitis and is responsible for infections followed by serious neurological sequelae. Although *Neisseria meningitidis* is not a common causative agent of meningitis in this age group, in our clinic cases we diagnosed a few cases in newborns and infants up to 3 months. Objectives: Study of bacterial meningitis diagnosed in newborns and infants up to 3 months. Setting peculiarities determined by the etiologic agent and the age of the children on the evolution and development of sequelae.

**Methods**

Retrospective study of cases of bacterial meningitis in children aged 0–3 months, admitted to our clinic between 2014 and 2016.

**Results**

In the time period studied in our clinic 6 cases of bacterial meningitis were diagnosed in infants younger than 3 months: 2 cases of neonatal meningitis produced by group B *Streptococcus* developed serious neurological sequelae (encephalomalacia, cortical atrophy, ventriculomegaly). *Neisseria meningitidis* was isolated from 2 newborns and 2 infants aged two months and caused purulent meningitis that evolved favorably, without sequelae.

**Conclusions**

Group B *Streptococcus* is a major cause of neonatal sepsis and bacterial meningitis respectively and is responsible for particularly severe neurological sequelae. That's why additional measures are required for surveillance of pregnant women, for early detection and intra-partum prophylactic treatment to reduce morbidity newborn by this infection. *Neisseria meningitidis*, although not considered a common etiologic agent of meningitis in infants, was responsible for four of the cases detected. This demonstrates the presence of *N. meningitidis* in the population and the effects of exposing children to highly pathogenic agents in the first days of life. Although cases treated in our clinic have evolved favorably, their presence is an alarm signal for epidemiological measures and an argument for completing the diagnostic guidelines of bacterial meningitis in infants.

### A5 The diagnostic and prognostic role of neutrophil to lymphocyte count ratio in sepsis

#### Alina Orfanu^1,2^, Victoria Aramă^1,2^, Ştefan Sorin Aramă^2^, Anca Leuştean^1^, Remulus Catană^1,2^, Anca Negru^1,2^, Gabriel Adrian Popescu^1,2^, Cristina Popescu^1,2^

##### ^1^National Institute for Infectious Diseases “Prof. Dr. Matei Balș”, Bucharest, Romania; ^2^Carol Davila University of Medicine and Pharmacy, Bucharest, Romania

###### **Correspondence:** Alina Orfanu (alina.lobodan@yahoo.com)

**Background**

The rapid diagnosis in sepsis, especially in cases without the evidence of a septic focus, represents an important challenge in clinical practice. The neutrophil/lymphocyte count ratio (NLCR) is an easily measurable parameter which can have a role in diagnosis of sepsis and also in assessing the severity of septic patients. NLCR was evaluated especially in patients with severe sepsis admitted in ICU and the data about its importance in so called community acquired sepsis are lacunar. Objective: To analyze the diagnostic and prognostic value of NLCR in patients with community acquired sepsis.

**Methods**

We performed a prospective single center study that included consecutive cases of sepsis admitted in Matei Balș Institute. For each patient we determined NLCR at admission. Two groups of patients were created: group 1 with NLCR < 10 and group 2 with NLCR > 10. Clinical and biological parameters and also the severity calculated with APACHE IV and APS scores were comparatively analyzed between the two groups.

**Results**

Fifty-five consecutive patients with sepsis were analyzed, with mean age of 57.92 years old and sex ratio F:M = 1.75:1. Group 1 included 21 patients with NLRC <10 (38.18 %) and group 2–34 patients with NLRC >10 (61.82 %). The comparative analysis between the two groups showed: more patients with 3 or 4 criteria for systemic inflammatory response syndrome (SIRS) in the second group (55.88 % vs. 42.85 %), more patients with identified etiology in the second group (32.32 % vs. 14.28 %) but without statistical significance (p = 0.2543), more patients with at least one organ dysfunction (76.47 % vs. 52.38 %, p = 0.032) and with procalcitonin at baseline > 2 ng/mL (medium PCT in the first group = 2.88 ng/mL vs. medium PCT in the second group = 16.88 ng/mL) in the second group. The correlations of initial value of NLRC with the severity scores (APACHE IV and APS) and with the estimated mortality rate were statistically significant (p = 0.01). The last correlation was stronger than the one between procalcitonin and the mortality rate (p = 0.05).

**Conclusions**

NLRC more than 10 was associated with severity expressed through the number of SIRS criteria and the presence of organ dysfunction at baseline. The value of NLRC was correlated with severity appreciated with APACHE IV and APS scores and with the estimated rate of mortality. NLRC is an easy to obtain biomarker which can be very helpful in order to assess the severity of sepsis.

### A6 Whooping cough in a HIV positive patient

#### Ramona Georgiana Stanculete^1^, Ana Vaduva Enoiu^1^, Adelina Raluca Marinescu^1^, Voichita Lazureanu^2^

##### ^1^Dr. Victor Babeş Clinical Hospital of Infectious Diseases and Pneumology, Timişoara, Romania; ^2^Dr. Victor Babeş University of Medicine and Pharmacy, Timişoara, Romania

###### **Correspondence:** Ramona Georgiana Stanculete (ramona.stanculete@gmail.com)

**Background**

Whooping cough is a highly contagious acute infectious disease caused by bacilli of the genus *Bordetella pertussis*, *parapertussis* and extremely rare in humans *B. bronchiseptica* with paroxysmal cough and variable impaired general condition, hematological changes and high risk of complications.

**Case report**

We present a 28 years patient diagnosed with HIV stage C3 in 1996, for which antiretroviral treatment (Abacavir + Lamivudine, Darunavir, Ritonavir scheme initiated in 2009). The patient came in the Clinic Infectious Diseases because of fever, cough initially dry, thereafter productive in bouts fallowed by episodes of faintness, sweating, exertional dyspnea, weight loss (~15 kg in the last 3 weeks). At the moment of hospitalization, the patient shows influenced general condition orthopnea , oxygen saturation 92 % without O2 mask, discreet congestive pharynx, tongue with chalky deposits, right basal crepitation rales, tachycardia HR = 125 b/min, liver with lower edge at 2 cm under the right costal margin, without meningeal irritation signs. Biological leukocytosis with lymphocytosis, inflammatory syndrome, lymphocyte CD4 = 243 cells/L, viremia = 5087 copies/mL. The chest-ray revealed interstitial drawing that looks like frosted glass designed predominantly perihilar right, dilated bronchi, alveolointerstitial type opacities suprahilar and infrahilar right side. We suspected pneumocystosis pulmonary tuberculosis (which was denied) and whooping cough, *Bordetella pertussis* IgM which was positive. We initiated treatment with oxygen on mask, rebalancing electrolyte solutions antibiotics, antifungical and expectorant, with a slowly favorable evolution.

**Conclusions**

The disease is very contagious and was considered a childhood infection but now it has been identified also in adults.

**Consent**

Written informed consent was obtained from the patient for publication of this Case report and any accompanying images. A copy of the written consent is available for review by the Editor of this journal.

### A7 *Cronobacter sakazakii* sepsis in varicella patient

#### Adelina-Raluca Marinescu, Alexandru Crișan, Voichița Lăzureanu, Virgil Musta, Narcisa Nicolescu, Ruxandra Laza

##### Dr. Victor Babeş University of Medicine and Pharmacy, Timişoara, Romania

###### **Correspondence:** Adelina-Raluca Marinescu (marinescu_adelina24@yahoo.com)

**Background**

Primo-infection with varicella zoster virus causes varicella, one of the most frequent pathologies in the first stage of childhood and not only. The complications of this disease may vary from pyogenic infections to pneumonia and neurological (cerebellar) lesions.

**Case report**

We present the clinical case of a 8-months old male baby with no significant pathological antecedents, admitted into the clinic of the “Victor Babeș” Timișoara Hospital of Infectious Diseases between 29.05-08.06.2016 with the diagnosis of varicella and *Cronobacter sakazakii* sepsis. The present disease started 5 days prior to admission into our clinic with polymorphous eruption in crust stage. Afterwards fever is associated (T = 39 °C). We mention the fact that all along the disease the baby was following ibuprofen treatment. Due to the fact that the baby’s condition remains strongly influenced, the non-steroidal anti-inflammatory treatment is associated with cefuroxime and acyclovir. In the 6^th^ day of disease the tumefaction of the right inferior limb is associated. At admission, the baby had strongly influenced condition, tumefaction of the inferior right limb, was dyspneic, sleepy with polymorphous eruption in crust stage, therefore the baby is admitted directly into the Intensive Care Unit. Hemoculture was positive for *Cronobacter sakazakii*. Thrombocytes = 55.000/μL, VSH = 45 mm/h, CRP = 272.79 mg/dL, serum urea = 43.8 mg/dL, creatinine = 0.33 mg/dL. Evolution was favorable with symptomatology remission under established treatment.

**Conclusions**

Within the last years in medical practice other complications of extreme gravity of varicella emerged: cellulitis, compartment syndrome, sepsis of various etiologies (*Streptococcus*, *Staphylococcus*, Gram negative germs, etc.) Performant modern apparel certainly contributes to establishing the bacteriologic diagnosis and implicitly the antibiogram which led to an optimal therapeutical behavior. From experience, we consider that the varicella zoster virus is more virulent, being able to cause severe forms or complication in certain patients categories.

**Consent**

Written informed consent was obtained from the parents for publication of this Case report and any accompanying images. A copy of the written consent is available for review by the Editor of this journal.

### A8 Anaerobes an underdiagnosed cause of prosthesis joint infection

#### Anca-Ruxandra Negru^1,2^, Daniela-Ioana Munteanu^1^, Raluca Mihăilescu^1^, Remulus Catană^1,2^, Olga Dorobăț^1^, Alexandru Rafila^1,2^, Emilia Căpraru^1^, Marius Niculescu^3^, Rodica Marinescu^2,3^, Olivera Lupescu^2,4^, Vlad Predescu^2,5^, Adrian Streinu-Cercel^1,2^, Victoria Aramă^1,2^, Daniela Tălăpan^1,2^

##### ^1^National Institute for Infectious Diseases “Prof. Dr. Matei Balș”, Bucharest, Romania; ^2^Carol Davila University of Medicine and Pharmacy, Bucharest, Romania; ^3^Clinical Hospital Colentina, Bucharest, Romania; ^4^Floreasca Emergency Hospital, Bucharest, Romania; ^5^“St. Pantelimon” Emergency Clinical Hospital, Bucharest, Romania

###### **Correspondence:** Daniela-Ioana Munteanu (danielaioana.mn@gmail.com)

**Background**

Anaerobic bacteria are not a common cause of prosthesis joint infection (PJI) but their implication may be greater than expected because of many false negative results due to the difficulties in isolating and identifying this type of bacteria. Sonication is a very important tool for the microbiological diagnosis giving the clinician a higher chance to find the microorganism involved in PJI. The aim of this study was to evaluate the prevalence of anaerobic bacteria in PJI and the main epidemiological characteristics of infections produced by anaerobes.

**Methods**

We performed a prospective 4-year study conducted in National Institute for Infectious Diseases Prof. Dr. Matei Balș, Bucharest between 2012 and 2016, in which we included all orthopedic sonicated implants sent from the regional centers. The implants were sonicated at 40 kHz on BactoSonic® ultrasonic bath (Bandelin, Germany) and the sonication fluid was cultured on both aerobe and anaerobe media. The period of incubation was 7 days for aerobe culture and 14 days for anaerobe cultures, respectively. Vitek®2 Compact automated system (Healthcare, BioMérieux, USA) was used for bacteria identification and antibiotic susceptibility. The interpretation of the results was made according to the latest EUCAST breakpoints.

**Results**

Among 94 sonicated orthopedic implants we isolated 5 anaerobic bacteria (5.3 % prevalence). *Propionibacterium acnes* was isolated in 3 cases, on one clavicle plate and on one hip and one shoulder replacement. *Parvimonas micra* and *Finegoldia magna* were the other two anaerobes isolated from a hip and knee prosthesis. Patients from whom anaerobe bacteria were isolated were 3 males and 2 females with a median age of 48 years (45; 67). All the anaerobic infections were monobacterial. The median time between surgery and the onset of the symptoms was 12 months (5;30). All the isolated strains were sensitive to all the antibiotics tested except for metronidazole to which *Propionibacterium acnes* is resistant.

**Conclusions**

In our study the prevalence of anaerobic PJI was 5.3 %, a prevalence similar to the one cited in the literature but this prevalence may be higher. Anaerobic bacteria are slow growing organisms and they require special conditions to grow, thus the number of false negative results can be significant.

### A9 *Streptococcus pneumoniae* meningitis presenting with normal CSF – case presentation

#### Ramona Ștefania Popescu^1,2^, Luminița Bradu^2^, Dragoș Florea^1,2^, Adrian Streinu-Cercel^1,2^

##### ^1^Carol Davila University of Medicine and Pharmacy, Bucharest, Romania; ^2^National Institute for Infectious Diseases “Prof. Dr. Matei Balș”, Bucharest, Romania

###### **Correspondence:** Ramona Ștefania Popescu (ramona.stefania.popescu@gmail.com)

**Background**

*Streptococcus pneumoniae*, along with *Neisseria meningitidis*, represent two of the most frequent causes of bacterial meningitis in adolescents and young adults. Delayed diagnosis or inadequate treatment of this condition may result in serious complications such as brain damage, hearing loss or even death.

**Case report**

We present the case of a 37 year old female, without any known medical condition, under no current medication, who was admitted in our clinic for fever, chills, fronto-occipital headache, nausea, vomiting, photophobia, symptoms which had been present for the last 36 hours with progressive enhancement. Upon physical examination she was febrile (38.3 °C), sweating, no pulmonary crackles or cardiac murmurs, her blood pressure was 100/60 mmHg and pulse rate was 74 bpm, without hepatosplenomegaly, oriented to time and place but with signs of meningeal irritation (Kernig's sign, Brudzinski's sign, and nuchal rigidity). Laboratory tests revealed leukocytosis with neutrophilia, biological inflammatory syndrome, electrolyte imbalance, positive procalcitonin, negative HIV, HTLV serology, negative markers of viral hepatitis B or C, normal urinalysis, negative hemocultures, and sinus X-ray showed bilateral maxillary sinusitis. Lumbar puncture at the moment showed no cerebrospinal fluid (CSF) modification: normal pressure, clear appearance, negative Pandy’s reaction, 1 cell/μL, protein – 31 mg/dL, glucose – 57 mg/dL, chloride – 700 mg/dL, negative CSF cultures. PCR using mass spectrometry (PLEX – ID) performed from CSF identified *Streptococcus pneumoniae*. Cranial magnetic resonance imaging (MRI) with contrast showed minimal meningeal enhancement, with no other modifications. Given all this data (clinical appearance and identification of *Streptococcus pneumoniae* in CSF using PLEX-ID), we considered the case as bacterial meningitis at an early stage, with normal CSF. She received treatment with meropenem 2 g q8h, vancomycin 1 g q12h and dexamethasone for 14 days with very good clinical response. The lumbar puncture repeated after 10 days showed no change from the previous examination. The patient fully recovered with no sequelae.

**Conclusions**

Bacterial meningitis exhibiting apparently normal CSF parameters, even if uncommon, is a very well-recognized phenomenon. A lumbar puncture which shows no abnormalities in CSF cannot exclude bacterial meningitis in the early stage of the disease. Empiric antibiotic should be used when the diagnosis of bacterial meningitis is strongly suspected even in the cases with normal CSF results. PCR technique represents a great tool in establishing the causative pathogen of meningitis, making possible the diagnosis of this condition even in the early stages when no changes in the CSF are noticeable.

**Consent**

Written informed consent was obtained from the patient for publication of this Case report and any accompanying images. A copy of the written consent is available for review by the Editor of this journal.

### A10 Extrapulmonary manifestations of infection with *Mycoplasma pneumoniae* – study on 24 cases

#### Daniela Anicuta Leca, Elena Bunea, Andra Teodor, Egidia Miftode

##### “Gr.T.Popa” University of Medicine and Pharmacy, Iași, Romania

###### **Correspondence:** Daniela Anicuta Leca (lecadaniela@ymail.com)

**Background**

Important etiologic agent of atypical pneumonia, *Mycoplasma pneumoniae* can cause frequent extrapulmonary manifestations (MEP – neurological, cardiac, skin, joints, gastrointestinal), which sometimes evolve isolated, thus making diagnosis and proper treatment difficult.

**Methods**

The study was retrospective and included 24 diagnosed cases of infection with *Mycoplasma pneumoniae*, hospitalized in the Department of Clinical Infectious Diseases, University of Medicine and Pharmacy “Gr.T.Popa” Iaşi, in the period 2013–2016.

**Results**

The annual evolution of morbidity revealed a relatively constant number of cases in the period 2013–2015, with a significant increase in cases in 2016 (45.83 %). The study included 14 children and 10 adults, sex ratio (F/M) 1.18 and a predominant urban origin of cases (15 urban/9 rural). Of the 24 patients, 21 showed signs of single respiratory manifestations (25 %) or in combination with skin manifestations (20.83 %), joints (12.5 %), liver (12.5 %) or neurological manifestations (45.83 %). Respiratory impairment was more common in adults (p = 0.007) and neurological impairment in children (p = 0.008) and in women (p = 0.05), with no difference by gender or age, referred to other extra pulmonary manifestations. MEP patients were admitted to hospital early (8 days versus 16 days). The average duration between the occurrence of respiratory and extrapulmonary events was a day for the joint, 4.6 days for neurological, 7 days for liver manifestations and 12.5 days for skin manifestations. MEP appearance was not favored by the patient’s immune suppressed status (p = 0.807), previously administered antibiotic therapy before hospitalization (p = 0.632). Those with only pulmonary manifestations frequently received macrolides prior to hospitalization (p = 0.011) with no difference between the 2 groups with the administration of beta-lactams (p = 0.102). Leukocytosis was detected in a high proportion of those with MEP (p = 0.05), and we identified no statistically significant difference between the two groups in terms of the values of other inflammation tests, of liver cytolysis tests, pulmonary radiographic changes (p = 0.795) or the frequency of complications (p = 0.345). The average length of hospitalization was higher in patients with MEP (10.83 days versus nine days those without MEP).

**Conclusions**

*Mycoplasma pneumoniae* should be considered in all cases with fever and respiratory symptoms evolving unfavorably treated with betalactamine and associates MEP.

### A11 The molecular diagnosis of severe bacterial sepsis in pediatric population

#### Mădălina Merișescu^1,2^, Gheorghiță Jugulete^1,2^, Adrian Streinu-Cercel^1,2^, Dragoș Florea^1,2^, Monica Luminos^1,2^

##### ^1^Carol Davila University of Medicine and Pharmacy, Bucharest, Romania; ^2^National Institute for Infectious Diseases “Prof. Dr. Matei Balș”, Bucharest, Romania

###### **Correspondence:** Mădălina Merișescu (madalinamerisescu@gmail.com)

**Background**

In children, bacterial sepsis is a not a very common condition but it is often accompanied by severe complications that can leave sequelae. Sometimes it can be life-threatening and requires a complex and quickly set treatment. Sepsis is more common in immunosuppressed patients.

**Methods**

We conducted a 5 years study from Jan 2011 to Dec 2015 on 2851 children admitted in our pediatric intensive care unit of the National Institute for Infectious Diseases for severe forms of SIRS and bacterial sepsis. Some of them were immunosuppressed. We watched the correlation of data obtained by hemocultures, CSF cultures versus PCR and the clinical evolution of the patients.

**Results**

In the 60 months of study, 265 children met the clinical and biological criteria for severe bacterial sepsis; 68 % of the patients came from other hospitals, reason for which we consider that the etiology is most likely nosocomial pathogens. Sex distribution was approximately equal. Considering age distribution, children in the group 3–5 years of age prevailed; 49 of them were immunosuppressed; 10 patients had acquired immunosuppression, 20 congenital and 19 mixed. We obtained 163 hemoculture positive results (61 %) and 112 obtained by molecular methods. The data were correlated with conventional methods of diagnosis.

**Conclusions**

Bacterial sepsis in children is a serious condition resulting in 24 deaths (14 %) in our study. It requires quick etiologic diagnosis and establishment of appropriate emergency treatment. PCR is an effective and rapid diagnosis method, identifying the causal agent in 42 % of cases.

### A12 Acute *Staphylococcus aureus* endocarditis with multiple septic complications in a patient with diabetes mellitus – case presentation

#### Ramona Ștefania Popescu^1,2^, Anamaria Dobrotă^2^, Adina Ilie^2^, Liliana Lucia Preoțescu^1,2^

##### ^1^Carol Davila University of Medicine and Pharmacy, Bucharest, Romania; ^2^National Institute for Infectious Diseases “Prof. Dr. Matei Balș”, Bucharest, Romania

###### **Correspondence:** Anamaria Dobrotă (dobrota_anamaria@yahoo.co.uk)

**Background**

*Staphylococcus aureus* is a common colonizing and infecting agent in humans [1], with increased mortality in case of bacteremia.

**Case report**

A 60 year old man with a history of diabetes and hypertension was referred to our clinic with the suspicion of infective encephalitis. The onset of symptoms was 10 days prior to presentation with high fever, malaise, lower limb muscular weakness which progressed to paresthesia, rigidity and intense headache with bradypsychia and bradylalia. The patient denied using any recreational drugs, recent head trauma or any other open skin lesions. At admission he was febrile, bedridden, with generalized muscular atrophy, Janeway lesions, petechiae, 5^th^ right toe necrosis, left endophthalmitis, holosystolic murmur in the apex with radiation to the axilla, hepatomegaly, with no signs of meningeal irritation. The lab reports showed leukocytosis with neutrophilia, anemia, thrombocytopenia, acute inflammatory syndrome, positive procalcitonin, hyperglycemia with high glycated hemoglobin level, hyponatremia, hypokalemia, hypoalbuminemia. Transthoracic echocardiography showed mobile mitral valve vegetation on the anterior leaflet, 10 mm in diameter, with normal ejection fraction. Two sets of blood cultures grew methicillin-susceptible *Staphylococcus aureus* (MSSA). Chest X-ray showed peripheral, bilateral, lower lobe infiltrative densities. Head magnetic resonance imaging (MRI) showed suggestive images for small abscesses in the supratentorial region. Given this data, the diagnosis of infective endocarditis with multiple septic emboli (pulmonary, ocular, cerebral) was established. Antibiotic therapy with linezolid and rifampin was initiated, due to a very good multiple organ penetration. As musculoskeletal secondary determination (septic arthritis, thigh abscess) and linezolid hematological side effects (anemia) occurred, we switched therapy to oxacillin, amikacin and levofloxacin. The patient had a good clinical response, with a total course of antibiotic therapy of 6 weeks. He was referred to a cardiovascular surgeon for further monitoring.

**Conclusions**

Patients suffering from uncontrolled diabetes have a higher risk of infections with endogenous microbes, with more severe outcomes. In our case, even if the MSSA strain isolated in hemocultures didn’t represent a therapeutic challenge in terms of antibiotics options, it did develop into a severe and life threatening illness. This strain of MSSA possibly used all its energy in the synthesis of multiple virulence genes (adhesion, penetration, invasion) which determined numerous extra-cardiac complications of endocarditis.

**References**

1. Sulla F, Bussius DT, Acquesta F, et al. Vancomycin minimum inhibitory concentrations and lethality in *Staphylococcus aureus* bacteremia. Germs. 2015;5:39–43.

**Consent**

Written informed consent was obtained from the patient for publication of this Case report and any accompanying images. A copy of the written consent is available for review by the Editor of this journal.

### A13 Is *Streptococcus suis* meningitis an under-diagnosed zoonosis?

#### Adriana Hristea^1,2^, Raluca Jipa^1,2^, Nicoleta Irimescu^1^, Irina Panait^1^, Eliza Manea^1^, Simona Merisor^1^, Cristian Niculae^1^, Daniela Tălăpan^1,2^

##### ^1^National Institute for Infectious Diseases “Prof. Dr. Matei Balș”, Bucharest, Romania; ^2^Carol Davila University of Medicine and Pharmacy, Bucharest, Romania

###### **Correspondence:** Adriana Hristea (adriana_hristea@yahoo.com)

**Background**

*Streptococcus suis* is a commensal pathogen of swine, a neglected pathogen that can be transmitted to humans by close contact with sick or carrier pigs. Meningitis and sepsis are the most frequent clinical manifestations in humans. A recently published systematic review identified 24 studies including 913 patients with meningitis due to *Streptococcus suis* [1]. Most cases have been reported in Asia. Objective: to draw attention to a potential under-diagnosed cause of meningitis and sepsis in our country.

**Methods**

We performed a retrospective analysis of patients diagnosed with *Streptococcus suis* infection between January 2005-December 2015 in one tertiary care hospital in Bucharest, Romania. We also reviewed the *Steptococcus pneumoniae* meningitis, according to the microbiological method confirming the etiology.

**Results**

We identified five patients hospitalized for *Streptococcus suis* infection – four patients had meningitis and one patient had endocarditis. Two patients reported contact with pork meat.

In patients with meningitis the median age was 43 years (IQR 40–45), with a male: female ratio of 3:1. Three patients presented with altered level of consciousness and two with severe hearing impairment. Cerebral spinal fluid (CSF) examination showed changes characteristic for bacterial meningitis. Latex agglutination test for *Streptococcus pneumoniae* was positive in two patients, nonetheless, *Streptococcus suis* was isolated in CSF in all patients. They were all treated with a third generation cephalosporin for 14 days and all of them improved. Two patients developed sequelae, consisting in permanent sensorineural deafness and ataxia.

In the studied period we identified a total of 194 cases of pneumococcal meningitis, in which the diagnosis was based on appearance on Gram stain and/or positive latex agglutination reaction of CSF samples in 82 (42 %) and positive culture in 112 (58 %) patients.

The patient with endocarditis was a 40 year old male, who was admitted for fever and malaise. Echocardiography showed aortic valve endocarditis. The patient underwent treatment with a beta-lactam/beta-lactamase inhibitor and an aminoglycoside for 4 weeks, and after valve replacement with a glycopeptide for 4 weeks.

**Conclusions**

*Streptococcus suis* could be miss-identified as other species of streptococci, thus in the settings that lack resources to routinely speciate α-hemolytic streptococci and where (undercooked) pork is a basic diet, underdiagnosis of *Streptococcus sui*s infection is likely. Hearing loss, although is not a complication related to *Streptococcus sui*s meningitis, is frequently reported.

**References**

1. van Samkar A, Brouwer MC, Schultsz C, van der Ende A, van de Beek D. *Streptococcus suis* meningitis: a systematic review and meta-analysis. PLoS Negl Trop Dis. 2015;9:e0004191.

## Antimicrobial resistance

### A14 *Klebsiella pneumoniae* isolated from blood. Antimicrobial resistance – past and present

#### Liana Cătălina Gavriliu^1,2^, Otilia Elisabeta Benea^1,2^, Șerban Benea^1,2^, Alexandru Rafila^1,2^, Olga Dorobăț^1,2^, Mona Popoiu^2^

##### ^1^Carol Davila University of Medicine and Pharmacy, Bucharest, Romania; ^2^National Institute for Infectious Diseases “Prof. Dr. Matei Balș”, Bucharest, Romania

###### **Correspondence:** Liana Cătălina Gavriliu (lianagavriliu@yahoo.com)

**Background**

Romania reports an increasing number of resistant *Klebsiella pneumoniae* (KP) strains isolated from invasive infections every year. The most recent report of the European Antimicrobial Resistance Surveillance Network (EARS-Net) has placed us among the first countries regarding KP resistance to fluoroquinolones, third generation cephalosporins, aminoglycosides, carbapenems, and multidrug resistance. We analyzed the antimicrobial resistance of bloodstream KP isolates in 2010 and 2015 in the National Institute for Infectious Diseases “Prof. Dr. Matei Balș”, Bucharest, Romania.

**Methods**

The antimicrobial susceptibility tests of KP strains isolated from blood between January 1^st^ 2010 - December 31^st^ 2010 and between January 1^st^ 2015 - December 31^st^ 2015 were analyzed. We compared the resistance trend between these two periods. Statistical analysis was performed using Fisher exact test. p < 0.05 was considered significant.

**Results**

We identified 18 strains of KP in 2010: 46.15 % resistant to aminopenicillin-betalactamase inhibitors association, 37.7 % resistant to piperacillin-tazobactam, 38.46 % resistant to third generation cephalosporins, 47.07 % resistant to fluoroquinolones, 41.17 % resistant to aminoglycosides. We didn’t find any strains resistant to carbapenems. 38.88 % of the strains had combined resistance to fluoroquinolones, third generation cephalosporins and aminoglycosides and 22.22 % were extended-spectrum betalactamases (ESBL) producing strains. In 2015, 37 strains of KP were isolated. The resistance rates to aminopenicillin-betalactamase inhibitors association, piperacillin-tazobactam, third generation cephalosporins, fluoroquinolones, aminoglycosides were 36.11 %, 24.32 %, 29.72 %, 27.02 % and 21.62 %, respectively. One strain was resistant to carbapenems. 21.62 % of the strains had combined resistance and 29.73 % were ESBL. The overall resistance to aminopenicillin-betalactamase inhibitors association, piperacillin-tazobactam, third generation cephalosporins, fluoroquinolones, gentamycin, amikacin, trimethoprim-sulfamethoxazole and the combined resistance decreased statistically non-significant. The same thing was noticed for the increasing rate of ESBL producing strains.

**Conclusions**

The percentage of KP strains isolated from blood increased between 2010 and 2015. We didn’t find statistically significant changes of the resistance rates of KP to all the classes of tested antimicrobials. The presence of carbapenem resistance among the isolates from 2015 could be a major problem for the public health and hospital-acquired infections control. Our data regarding the proportion of resistant bloodstream KP strains are different from those reported by EARS-Net. We found lower and decreasing resistance rates to fluoroquinolones, aminoglycosides, third generation cephalosporins, carbapenems and combined resistance. Still, with Romania occupying one of the first places in Europe regarding KP isolated from invasive infections resistance to antibiotics, further monitoring is mandatory and efforts should be made in order to control this problem.

### A15 Antibiotics resistance in *Staphylococcus aureus* isolated from blood cultures

#### Livia Dragonu^1,2^, Augustin Cupşa^1,2^, Iulian Diaconescu^1,2^, Irina Niculescu^1,2^, Lucian Giubelan^1,2^, Florentina Dumitrescu^1,2^, Andreea Cristina Stoian^1^, Camelia Guţă^2^, Simona Puiu^2^

##### ^1^University of Medicine and Pharmacy Craiova, Craiova, Romania; ^2^“Victor Babeș” Clinical Hospital of Infectious Diseases and Pneumology, Craiova, Romania

###### **Correspondence:** Livia Dragonu (livia_dragonu@yahoo.com)

**Background**

*Staphylococcus aureus* has a high incidence in human infectious pathology, registering severe infections caused by antibiotic-resistant strains. In Romania, methicillin-resistant *S. aureus* (MRSA) accounts to up to 53.8 % of *S. aureus* strains isolated from blood cultures (EARS-Net report 2012). In many European countries, the percentage of MRSA invasive infections caused by *S. aureus* decreased below 25 % through the implementation of programs designed to limit bacterial resistance. Objectives: to determine the antibiotics resistance of *S. aureus* strains isolated from blood cultures.

**Methods**

We performed a retrospective study (01.01.2011 - 06.01.2016) analyzing blood cultures results collected from patients hospitalised in the Infections Disease and Pneumology Clinics of “Victor Babeş” Hospital from Craiova. The antibiotics resistance of 180 *S. aureus* strains isolated from blood cultures was analyzed based on the results of disc diffusion sensitivity testing. The methicillin-resistant strains presented an inhibition zone around the disk of cefoxitin (30 mcg) under 22 mm. The results’ evaluation was carried out by comparing percentage differences and by performing the Chi^2^ test.

**Results**

During the study period, 1806 blood cultures were performed, and only 233 (12.9 %) were positive. Blood cultures were positive for *S. aureus* in 77 % of cases, *Klebsiella pneumoniae* in 8.1 % of cases and *Escherichia coli* in 4.5 % of cases. Distribution by age of blood culture positive for *S. aureus* were: under 1 year old - 14.8 % of cases, 1–16 years old - 17.3 % and over 16 years old - 67.8 %. The blood cultures MRSA percentage was 45.5 % (82 out of 180 tested strains) with a peak in 2013 (28 strains) compared to 2015 (8 strains). The antibiotics resistance in *S. aureus* isolated strains was: to penicillin 83.9 %, to oxacillin 45.5 %, to clarithromycin 45.4 %, to amoxicillin/clavulanic acid 31.6 %, to trimethoprim/sulfamethoxazole 30.8 %, to ceftriaxone 24.5 %, to meropenem 19.3 %, to levofloxacin 18 %, to gentamicin 17.7 %, to chloramphenicol 11.9 %, to clindamycin 10.4 %, to vancomycin 7.78 %, to linezolid 4.9 %. The MRSA percentage was higher in infants (53.3 %) compared to adults (44.8 %), without statistically significant difference.

**Conclusions**

*S. aureus* was the most common etiologic agent isolated in blood cultures, with a high prevalence of methicillin-resistant strains (45.5 %). From the tested antibiotics, the lowest resistance was observed at linezolid and vancomycin. Clindamycin, aminoglycosides and fluoroquinolones were active against strains of MRSA without antibiotics multiresistance.

### A16 Predominance of CTX-M enzymes in extended-spectrum β-lactamase-producing Enterobacteriaceae in two hospitals of Quebec City

#### Bunescu Irina^1^, Marilyse Vallée^2^, Ann Huletsky^2^, Dominique K. Boudreau^2^, Ève Bérubé^2^, Richard Giroux^2^, Jean Longtin^3^, Yves Longtin^4^, Michel G. Bergeron^2^

##### ^1^Department of Infectious Diseases and Medical Parasitology, Medical and Pharmaceutical University Nicolae Testemițanu, Chișinău, Republic of Moldova; ^2^Research Center of Infectious Diseases of Laval University Québec, Québec, Canada; ^3^Hospital Center of Laval University, Québec, Canada; ^4^Cardiology and Pulmonology University Institute of Québec, Québec, Canada

###### **Correspondence:** Bunescu Irina (bunescu.irina@yahoo.com)

**Background**

β-lactamases production remains the most important mechanism of β-lactam resistance. The most prevalent extended-spectrum β-lactamases (ESBL) in hospital and community settings that preferentially hydrolyze cefotaxime, belong to plasmid-mediated ESBL, is CTX-M. Our objective was to study bacterial resistance to improve the diagnosis and treatment of infections with these pathogens.

**Methods**

Fifty-nine strains (isolated from urine and several from blood) were collected between June 23^rd^ and September 23^rd^ 2011 from two hospitals of Quebec City in Canada. Strains were sent to the Research Center of Infectious Diseases of Laval University in Quebec City. Identification and antibiotic resistance were determined using the Vitek® 2 System (bioMérieux). ESBL production was tested according to CLSI recommendations. Crude DNA extracts were prepared from each strain by a rapid DNA extraction method. DNA was used for PCR amplification using different primers specific to *bla*_TEM_, *bla*_SHV_, and *bla*_CTX-M_ genes. All amplicons produced were sequenced, to identify the genes of resistance.

**Results**

From 59 strains tested, 32 (54.2 %) were ESBL+, and 28 (45.8 %) were ESBL-. *Escherichia coli* was the most prevalent species with a percentage of 88.1 % of the strains tested. The 32 ESBL producing-strains were positive for at least one of the resistance genes tested. The predominant resistance gene was *bla*_CTX-M_ either alone or in combination with the *bla*_TEM_ gene, all present in *E. coli*. Sequence analysis of the 28 *bla*_CTX-M_ genes found revealed that 18 strains were members of CTX-M group 1, and 10 were members of CTX-M group 9. Two different resistance genes were identified in 15 strains: *bla*_CTX-M_ + *bla*_TEM_ (13) and *bla*_SHV_ + *bla*_TEM_ (2). However, sequence data revealed that all *bla*_TEM_ genes identified in both ESBL+ and ESBL strains did not encode ESBL. This suggests that the resistance phenotype in ESBL-producing strains with two resistance genes should be conferred by *bla*_CTX-M_ or *bla*_SHV_ genes. Another resistance gene, not tested in this study, is most likely responsible for the ESBL resistance phenotype in the strain containing only the *bla*_TEM_-non-ESBL gene.

**Conclusions**

This study showed that *bla*_CTX-M_ are the most prevalent resistance genes in ESBL-producing Enterobacteriaceae in Quebec City and are part of groups 1 and 9. This is consistent with global data showing the predominance of the CTX-M ESBL worldwide and with recent epidemiological data, in other regions in Canada, showing that CTX-M-15 as well as CTX-M-14 and CTX-M-9, being respectively part of groups 1 and 9, are most commonly found.

### A17 Postoperative meningoencephalitis with *Acinetobacter baumannii* XDR – a therapeutic challenge - Case report

#### Cleo Nicoleta Roșculeț, Dalila-Ana Toma, Catrinel Ciuca, Daniela Tălăpan, Cătălin Apostolescu, Andrei Rogoz, Andrei Stangaciu, Viorica Mitescu, Tudor Vladoiu, Doina Iovănescu

##### National Institute for Infectious Diseases “Prof. Dr. Matei Balș”, Bucharest, Romania

###### **Correspondence:** Cleo Nicoleta Roșculeț (cleo_n_rosculet@yahoo.com)

**Background**

*Acinetobacter baumannii* is an aerobic Gram negative bacterium which, depending on the profile of resistance (MDR- multidrug resistance, XDR- extensive drug resistance, PDR- pandrug resistance), may be exceptionally the source of severe infection, more or less therapeutically controllable.

**Case report**

We have chosen to bring forward the peculiar case of a 62 year old man, who was transferred to our department for agitation, confusion (GCS = 7–8) and fever occurring 12 days in the postoperatory period (left otomastoidectomy) under the suspicion of bacterial meningoencephalitis. The patient’s medical records emphasize a history of chronic suppurative otomastoiditis surgically treated twenty years before. From that moment forward, the patient presented numerous relapses (left ear pain, purulent otorrhea) accompanied by neurological involvement (left facial nerve peripheral palsy), for which local treatment and outpatient antibiotic therapy were conducted. Two years before admission, a tympanic-mastoid cavity with purulent discharge was clinically perceived. MRI confirmed the presence of an abscess in the left petrous part of the temporal bone. Successive evaluations were conducted through multidisciplinary approach, but the surgical intervention was delayed. On the current admission, blood and multiple samples were taken and a lumbar puncture was performed. The CSF aspect was highly suggestive of bacterial meningoencephalitis. The patient was immediately started on broad-spectrum antibiotics aimed at covering highly resistant bacteria. CSF cultures came back positive for *Acinetobacter baumannii* XDR (colistin sensitivity present). Following complex therapy, the clinical and biological profile improved. The neurological status became stable (GCS = 12–13), thus permitting surgical reintervention.

**Conclusions**

All minimal ear infectious involvement must be promptly addressed in order to prevent local complications that may lead to destructive irreversible lesions. Once these lesions appear, a multidisciplinary approach becomes compulsory and high financial and human costs are implied.

**Consent**

Written informed consent was obtained from the patient for publication of this Case report and any accompanying images. A copy of the written consent is available for review by the Editor of this journal.

### A18 Septic arthritis with *Burkholderia cepacia*

#### Michaela Oana, Simona Costin

##### “Dr. Constantin Papilian” Military and Emergency Hospital, Cluj-Napoca, Romania

###### **Correspondence:** Michaela Oana (sincostin@yahoo.com)

**Case report**

We present the case of a 39 years urban female, which shortly after an arthroscopic procedure develops fever, chill, partial functional impotence, local swelling. In joint puncture we isolated *Burkholderia cepacia*, susceptible only to meropenem, minocycline and trimethoprim. After 2 weeks of meropenem and 4 weeks of minocycline the evolution was favorable, but soon subfebrility, swelling, pain and functional impotence reappeared. After 8 months *Burkholderia cepacia* (with similar susceptibility) is isolated again. She took meropenem (6 g/day) in combination with doxycycline 14 days, followed by meropenem and trimethoprim. The intra-articular devices were removed. The patient became afebrile, local evolution was favorable and the culture negative in day 18. On day 21 she develops agranulocytosis and we discontinued trimethoprim, decreased the dose of meropenem and supplemented with granulocyte growth factor. Then the evolution was favorable: local, hematological and bacteriological. We continued the therapy another 5 weeks with meropenem and up to 6 weeks with minocycline.

**Consent**

Written informed consent was obtained from the patient for publication of this Case report and any accompanying images. A copy of the written consent is available for review by the Editor of this journal.

### A19 A novel approach for managing hard-to-treat infections

#### Alina Cristina Neguț^1,2^, Oana Săndulescu^1,2^, Anca Streinu-Cercel^1,2^, Maria Magdalena Moțoi^1^, Mircea Ioan Popa^2^, Adrian Streinu-Cercel^1,2^

##### ^1^National Institute for Infectious Diseases “Prof. Dr. Matei Balș”, Bucharest, Romania; ^2^Carol Davila University of Medicine and Pharmacy, Bucharest, Romania

###### **Correspondence:** Alina Cristina Neguț (negoitza_alina@yahoo.com)

**Background**

The emergence and spread of antibiotic resistance has become a worldwide top priority health problem. Besides genetic resistance, bacteria have developed a multitude of mechanisms leading to tolerance to antimicrobials. It is estimated by the Centers for Disease Control and Prevention that in the USA 23,000 people die every year due to infections with multidrug resistant bacteria.

**Methods**

We have performed a pilot experimental study in the National Institute for Infectious Diseases „Prof. Dr. Matei Balș”, treating six patients with combined therapy: antibiotics and bacteriophages. The study received Ethics Committee approvals: Matei Balș c/5101/02.10.2014 and Carol Davila 47/26.01.2015. The pathogenic agents were evaluated for phage susceptibility to commercial Georgian phage cocktails, namely Pyo and Intesti (Eliava BioPreparations, Tbilisi, Georgia). We included in the study patients failing antibiotic therapy, with hard-to-treat infections due to antibiotic resistant bacteria, biofilm formation or hard to sterilize infection sites. Phages were administered orally or/and topically.

**Results**

This pilot study included the following types of infections: recurrent endocarditis with *Staphylococcus aureus*, chronic osteomyelitis with *S. aureus* and *P. aeruginosa*, periprosthetic dorsolumbar soft-tissue infection with *S. aureus*, chronic cutaneous infection with *S. simulans*, *E. coli* and *P. aeruginosa*, axillary hidradenitis with *Proteus mirabilis* and *S. epidermidis* and chronic osteomyelitis with *S. aureus*. For all six cases the combined therapy proved to be safe, with no adverse reactions and no adverse changes in laboratory parameters. A total of 5 patients had negative cultures during therapy, but 7 days after the end of therapy the cultures became positive again for 3 of the cases. For one patient (recurrent endocarditis with *S. aureus*) we concluded that the timespan between relapses was longer after receiving the combined therapy compared with antimicrobial treatment alone. In one case (periprosthetic dorsolumbar soft-tissue infection with *S. aureus*) we achieved sustained bacteriological response, although the patient was considered hard-to-treat due to a history of multiple drug allergy syndrome, presence of multiple foreign bodies (metallic rods and screws), with biofilm formation and a history of unsuccessful long term antibiotic therapy.

**Conclusions**

To our knowledge, this is the first experimental trial conducted in Romania with combined therapy antibiotics and bacteriophages since 1975, which proved the safety of bacteriophages in clinical settings. We consider this study the cornerstone for future research regarding in vivo phage therapy in Romania.

**Acknowledgement**

Carol Davila University of Medicine and Pharmacy, Young Researchers Grant 28341/2013; PhD thesis of ACN.

### A20 Nineteen months surveillance for multidrug resistant organisms (MDRO) by detecting asymptomatic colonization

#### Daniela Tălăpan^1,2^, Olga Mihaela Dorobăț^1^, Mona Popoiu^1^, Alexandru Mihai^1^, Doina Iovănescu^1^, Cleo Roşculeț^1^, Cătălin Apostolescu^1,2^, Gabriel-Adrian Popescu^1,2^, Adrian Abagiu^1^, Ruxandra Moroti-Constantinescu^1,2^, Adriana Hristea^1,2^, Victoria Aramă^1,2^, Otilia Benea^1,2^, Mădălina Simoiu^1^, Rodica Bacruban^1^, Adrian Streinu-Cercel^1,2^, Alexandru Rafila^1,2^

##### ^1^National Institute for Infectious Diseases “Prof. Dr. Matei Balș”, Bucharest, Romania; ^2^Carol Davila University of Medicine and Pharmacy, Bucharest, Romania

###### **Correspondence:** Daniela Tălăpan (dtalapan@gmail.com)

**Background**

Healthcare-associated infections (HAI) surveillance programs may focus, amongst others, on specific microorganisms like multidrug resistant organisms (MDROs). Methicillin-resistant *Staphylococcus aureus* (MRSA) and other MDROs of public health concern may pose problems in acute and non-acute healthcare settings, so detecting and monitoring these is important in hospital-based prevention, surveillance and control efforts. The program of HAI surveillance and control in the National Institute for Infectious Diseases “Prof. Dr. Matei Balș” includes actively screening for MDROs of all patients admitted in the ICU and regardless of the ward, for those transferred from other healthcare settings into our hospital.

**Methods**

The study was conducted between January 1^st^ 2015 – July 31^st^ 2016 by collecting swabs from criteria based selected patients: pharyngeal (908), nasal (880) and rectal (823). A total of 2,611 swabs were processed in 19 months’ interval. The targeted microorganisms were MRSA, vancomycin resistant enterococci (VRE) and other multidrug resistant Gram-negative bacteria. Swabs were inoculated on appropriate chromogenic culture media from Oxoid, UK (Brilliance MRSA agar, Brilliance VRE agar, Brilliance ESBL agar) and were interpreted at 24–48 hours of incubation at 37 °C, aerobic atmosphere. All microorganisms were identified and tested for susceptibility to antimicrobials by MicroScan Walk Away 96 Plus (Siemens, USA). EUCAST guidelines were used for interpretation. The KPC, MBL and OXA-48 Confirm kits (Rosco Diagnostica, Denmark) were used to determine the carbapenemase-producing strains.

**Results**

MRSA was detected in 4.84 % of the pharyngeal and 5.79 % of the nasal swabs. The positive rectal swabs, counted one per patient, were 28.06 % (231/823). A percentage of 68.83 % of rectal swabs were positive for one microorganism, 25.97 % positive for 2 microorganisms and 5.20 % for 3 microorganisms. The total number of microorganisms isolated from rectal swabs was 315 (*Escherichia coli* 32.69 %, VRE 26.34 %, *Klebsiella pneumoniae* 23.17 %, *Pseudomonas aeruginosa* 5.39 %, *Acinetobacter baumannii* 4.12 % and other MDROs less than 3 % each). VRE carriage varied between 21.89 % in 2015 and 30.13 % in 2016. The proportion of *Klebsiella pneumoniae* carbapenemase-producer strains has increased from 5/38 (13.1 %) in all year 2015 to 10/35 (28.5 %) in first 7 months of 2016. Only one other microorganism (*Enterobacter cloacae*) was carbapenemase producer.

**Conclusions**

MRSA colonization varied between 4.84 % (pharyngeal) and 5.79 % (nasal). VRE carriage increased with 10 % in 2016 and carbapenemase-producing *Klebsiella pneumoniae* doubled in the first 7 months of 2016, compared with 2015.

**Acknowledgement**

We are thankful to Emilia Căpraru and Mariana Răduț for their important help in isolation of bacterial strains included in the study.

### A21 Antimicrobial resistance of Gram-positive cocci isolated from clinical specimens in the National Institute of Infectious Diseases “Prof Dr. Matei Balș” between 2009–2015

#### Olga Mihaela Dorobăț^1^, Daniela Tălăpan^1,2^, Alexandru Mihai^1^, Ioana Bădicuț^1,2^, Mona Popoiu^1^, Alina Borcan^1,2^, Alexandru Rafila^1,2^

##### ^1^National Institute for Infectious Diseases “Prof. Dr. Matei Balș”, Bucharest, Romania; ^2^Carol Davila University of Medicine and Pharmacy, Bucharest, Romania

###### **Correspondence:** Olga Mihaela Dorobăț (olgadorobat@yahoo.com)

**Background**

Bacterial infections remain one of the important causes of disease worldwide and the options for treating are limited due to the emergence of antimicrobial resistance (AMR). The aim of this study is to evaluate the AMR of Gram-positive cocci isolated in National Institute of Infectious Diseases “Prof. Dr. Matei Balș” between 2009–2015.

**Methods**

A total of 7808 non-duplicated strains: 2067 *Staphylococcus aureus*, 651 coagulase-negative staphylococci, 292 *Streptococcus pneumoniae*, 922 *Enterococcus faecalis*, 251 *Enterococcus faecium* and 3621 *Streptococcus pyogenes* were tested for their antimicrobial resistance. Susceptibility tests were performed in automated systems Vitek 2C (BioMerieux) and MicroScan Walkaway (Siemens), and also with Sensititre (Thermo Scientific) MIC plates and Etest (BioMerieux); for *Streptococcus pneumoniae* disk diffusion method was used. Results were interpreted using CLSI and EUCAST criteria.

**Results**

*Staphylococcus aureus* resistance to oxacillin varied from 34.4 % in 2009 to 55.7 % in 2011 and has decreased to 44.8 % in 2015. Erythromycin and clindamycin resistant strains of *Staphylococcus aureus* have increased between 2009–2014 from 45.2 % to 58.1 %, and from 13.8 % to 58.3 %, respectively. *Staphylococcus aureus* strains resistant to quinolones have varied annually to ciprofloxacin (13.4–21.9 %) and moxifloxacin (8.4–16.8 %) without a clear increasing or decreasing tendency. Trimethoprim-sulfamethoxazole resistance has remained at low levels (0.5–1.5 %) and no strains have been resistant to linezolid and vancomycin. The incidence of resistance was higher for coagulase-negative staphylococci, most isolated from blood, 60.5–81.7 % for oxacillin, 21.2–42.1 % for trimethoprim-sulfamethoxazole. The incidence of penicillin-resistant *Streptococcus pneumoniae* strains isolated from invasive infections decreased from 37.5 % in 2010 to 12.0 % in 2015. *Enterococcus faecium* was more resistant compared with *Enterococcus faecalis* to all tested antimicrobials, for gentamicin with 45.7–75.5 % compared with 38.7–61.5 % and for ciprofloxacin 35.7–64.5 % compared to 85.7–100 %. The first vancomycin-resistant *Enterococcus faecium* strains were isolated in 2012 with an incidence of 4.3 % which reached 35.0 % in 2014. Only two *Enterococcus faecalis* vancomycin-resistant strain were isolated, one in 2013 and one in 2014. *Streptococcus pyogenes* resistance to erythromycin increased significantly over time from 7.6 % in 2010 to 16.9 % in 2015.

**Conclusions**

Oxacillin resistance incidence in *Staphylococcus aureus* has registered a slight decrease, since 2011, from 55.7 % to 44.8 % in 2015. Coagulase-negative staphylococci are significantly more resistant than *Staphylococcus aureus*. In 2012 the firsts strains of *Enterococcus faecium* resistant to vancomycin were isolated and in 2014 their proportion reached 35.0 %. Continuous monitoring of antimicrobial resistance is highly needed, in particular to guide an efficient empirical therapy.

### A22 The high percentage of carbapenem-resistant Gram-negative bacilli in Romania: an analysis and some proposals

#### Gabriel Adrian Popescu^1,2^ (gabrielp9@yahoo.com)

##### ^1^National Institute for Infectious Diseases “Prof. Dr. Matei Balș”, Bucharest, Romania; ^2^Carol Davila University of Medicine and Pharmacy, Bucharest, Romania

**Background**

Carbapenems are probably the most important last-line antibiotics for treatment of infections involving multidrug-resistant gram-negative bacteria. In the last years, the emergence of carbapenem-resistance of Gram-negative bacilli is considered one of the worldwide most important challenges of public health.

**Methods**

The analysis is based on antimicrobial resistance data collected by NIPH for 2012–2015 and national antibiotics sales for 2011–2015, provided by IMSHealth Romania. The European level data were obtained from EARS Net and ESAC Net newest reports.

**Results**

The percentage of carbapenem-resistance in *Escherichia coli* was low in the past four years, 0 (2012) - 2.1 % (2015) in the past four years, but several resistant isolates represent an alert for the risk of community acquired infections due to XDR strains. For other Gram-negative bacteria the carbapenem-resistance is already high or rapidly emerging: *Klebsiella pneumoniae*, from 15 % (2012) to 35.1 % (2015), third highest resistance level in EARS Net; *Pseudomonas aeruginosa* 60 % (2012) - 66.4 % (2015), first resistance percentage in EARS Net and *Acinetobacter baumannii* 81.5 % (2012) to 82.1 % (2015) fourth resistance level in EARS Net; similar high percentages were obtained when the results only from Infectious Diseases Hospitals were analyzed. The risk factors for carbapenem-resistance are carbapenem usage and carbapenem-resistant bacteria transmission. The carbapenems usage in Romania increased with 113 % after 2013 (R2 = 0.746) and germ transmission is probably at a high level in our hospitals, as several very recent examples had proven.

**Conclusions**

The high percentage of carbapenem-resistance in Romania is related to misuse of carbapenems (generated by some pseudo-science myths) and to germ transmission in Romanian hospitals. Both problems need to be addressed and Infectious Diseases specialists could play a pivotal role in the control of this public health severe emergency

**Acknowledgement**

The author acknowledges support received from National Institute for Public Health and IMSHealth Romania for data collection.

### A23 Etiological, clinical and therapeutic considerations on 78 cases of healthcare associated meningitis or ventriculitis admitted in the “Sf. Parascheva” infectious diseases clinical hospital, Iași, from 2011 to 2015

#### Mihnea Hurmuzache^1,2^, Georgiana Enache^1^, Alexandra Ciocan^1^, Mircea Bararu^3^, Madalina Popazu^1^

##### ^1^Infectious Diseases Hospital “Sf Parascheva”, Iași, Romania; ^2^“Gr.T.Popa” University of Medicine and Pharmacy, Iași, Romania; ^3^“N. Oblu” Clinical Hospital of Neurosurgery, Iaşi, Romania

###### **Correspondence:** Mihnea Hurmuzache (mihnea_hurmuzache@yahoo.com)

**Background**

Healthcare-associated meningitis or ventriculitis is a serious and life-threatening complication of invasive neurosurgical procedures or penetrating head trauma.

**Methods**

The current study reviewed 78 cases of health care-associated meningitis or ventriculitis which were treated in “Sf Parascheva” Infectious Diseases Hospital Iași from January 2011 to December 2015.

**Results**

The reviewed cases involved 78 adult patients. The age of the patients was between 18 and 89, with a higher frequency in the age group 60–80. Healthcare-associated bacterial meningitis may occur after neurosurgical procedures, head trauma, placement of internal or external ventricular catheters, ENT and maxillofacial surgery. From 78 cases, in 42 there was a positive cerebrospinal fluid culture. The most frequent agents are Gram negative bacilli (*Pseudomonas* spp, *Acinetobacter* spp, *Escherichia coli* and *Klebsiella pneumoniae*) and Gram positive cocci (*Staphylococcus aureus* – with a particular focus on MRSA, coagulase-negative *Staphylococcus* and *Streptococcus pneumonia*e). At the admission in the clinic the signs and symptoms were: fever, headache, changes in mental status (Glasgow Coma scale 3–12), nausea/vomiting, focal neurologic deficit, neck stiffness, seizures and photophobia. The diagnostic evaluation involved neuroimaging investigation (CT/MRI) and cerebrospinal fluid analysis. Empiric antimicrobial therapy should be directed towards the likely infecting pathogen. The complications involved were brain abscess, ventriculitis, cranial nerve damage, deafness and blindness. The outcome was negative in 38 % of cases and positive in 62 % (with disability in 30 patients and without disability in 32 patients).

**Conclusions**

Healthcare-associated meningitis or ventriculitis remains challenging in terms of diagnosis, treatment and prevention.

### A24 Nosocomial infection dynamics in an Intensive Care Department – an overview (epidemiological and clinical monitoring, advanced therapeutic intervention)

#### Doina Viorica Iovănescu^1^, Cleo Nicoleta Roșculeț^1^, Andrei Rogoz^1,^ Cătălin Gabriel Apostolescu^1,2^, Viorica Mitescu^1^_,_ Tudor Vladoiu^1^, Dalila Toma^1^, Catrinel Ciuca^1^

##### ^1^National Institute for Infectious Diseases “Prof. Dr. Matei Balș”, Bucharest, Romania; ^2^Carol Davila University of Medicine and Pharmacy, Bucharest, Romania

###### **Correspondence:** Doina Viorica Iovănescu (liviuiovanescu@yahoo.com)

**Background**

In a medical world in which infections are caused by bacteria with increased virulence, nosocomial infection is a serious threat for all medical departments. Epidemiological data must be thoroughly collected and interpreted in the setting of clinical facts, thus guiding the complex process of therapeutic intervention.

**Methods**

We analyzed the clinical data obtained in our ICU department over a period of 6 months in 2016 and outlined the main patterns involved in the occurrence and evolution of nosocomial infections. The main sources of nosocomial infections were venous and arterial catheters responsible for bloodstream infections (4 cases), orotracheal intubation (5 cases) causing severe ventilator-associated pneumonia and bladder catheterization responsible for most cases of urinary tract infections (4 cases). Every nosocomial infection’s ethology was analyzed and correlated to its clinical impact. Suggestive signs developed when peripheral catheters were implanted and fever was the first sign of catheter-associated bloodstream infection. The purulent aspect of pulmonary secretions in intubated patients was the first omen of VAP. The turbid aspect of urine indicated a potentially hospital-acquired infection in patients with bladder catheterization.

**Results**

In our Institute – specialized in diagnosis and treatment of infectious diseases – nosocomial infections in different stages of evolution are part of our current pathology. Based on this argument, in our ICU from January to July 2016, there were 13 cases considered “imported” nosocomial infections – failure of previous treatments – and 1 case was declared “novel” nosocomial infection – ventilator associated pneumonia (VAP) with *A. baumannii* in an immunocompromised patient with end stage AIDS/HIV infection. Unfortunately, his evolution was continually unfavorable with subsequent death.

Ten patients with “imported” nosocomial infections had favorable evolutions, and were discharged in good conditions. The 2 others died as a result of VAP with MRSA and VAP with *P. aeruginosa* respectively.

**Conclusions**

Most of the patients admitted to our clinic over a period of 6 months were infected with multi-resistant bacteria at the time of admission thus emphasizing the spread of germs with high antibiotic resistance in the community and the necessity of a thorough bacteriological survey at the time of inter-hospital transfer. Nosocomial infections remain a constant threat whose dynamics must be closely monitored in the future.

## Viral hepatitis - epidemiology, treatment and monitoring

### A25 Safety and efficacy of interferon free treatment in patients with HCV chronic hepatitis- experience of a single Internal Medicine center

#### Laura Iliescu, Georgiana Minzala, Letitia Toma, Mihaela Baciu, Alina Tanase, Carmen Orban

##### Fundeni Clinical Institute, Bucharest, Romania

###### **Correspondence:** Laura Iliescu (laura_ate@yahoo.com)

**Background**

The use of directly acting antiviral agents in chronic HCV hepatitis has proved to be efficient and safe in clinical trials.

**Methods**

Transaminases, bilirubin, blood cell count at initiation, 2, 4, 8, 12 weeks of treatment were evaluated in 124 patients with HCV associated cirrhosis, undergoing treatment with dasabuvir (Exviera™), ombitasvir, paritaprevir, ritonavir (Viekirax™) and ribavirin. HVC-RNA was determined at initiation and at end of therapy (EOT).

**Results**

We included 124 patients of which 56 were males. The mean age was 51.7 ± 23.4 years. Two patients with liver transplant and a patient with HCV genotype 1a received 24 weeks of treatment. Two patients did not receive ribavirin: one with minor thalassemia, one with polyarthritis. We included 2 patients with diabetic nephropathy with end stage renal disease (ESRD), one undergoing hemodialysis. 58 patients have reached the end of treatment (one patient with liver transplant); 57 of them had undetectable viremia; one had viremia <15 IU/mL. 15 patients have been evaluated at 12 weeks after EOT; they all had undetectable viremia at EOT, and had sustained virologic response. Ribavirin was discontinued in 7 patients - 2 due to severe cutaneous reactions - erythroderma. Both patients with ESRD discontinued ribavirin after week 2 due to severe anemia. One patient with systemic lupus stopped ribavirin at week 2 due to hypotension. A patient with thalassemia presented duodenal hematoma with pancreatic reaction in the first week, with improvement under medical therapy and discontinuation of ribavirin, with undetectable viremia at EOT. Ribavirin doses were decreased at week 4 in a patient with variceal bleeding, and stopped at week 8 due to severe anemia. These patients also had virologic response at EOT. Another patient presented with hepatocellular carcinoma in week 2; he underwent transarterial chemoembolization in week 8, with decompensation by ascites, ultimately with good evolution, undetectable viremia at EOT and complete response of the nodule. The mean initial levels of transaminases were double the normal values; they decreased dramatically after the first two weeks of treatment and low levels were maintained throughout therapy. The mean initial bilirubin level was normal, with values increasing during the first month: 1.4 mg/dL at week 2 and 2.84 mg/dL at week 4. Afterwards there was a small constant decrease and normalization after EOT.

**Conclusions**

Therapy with Exviera™/Viekirax™/ribavirin is well tolerated, safe and efficient in patients with chronic HCV hepatitis and cirrhosis. Ribavirin dose reductions should not be a milestone in therapy.

### A26 Viusid in treatment of chronic viral hepatitis B and C

#### Victor Pantea^1^, Gheorghe Placinta^1^, Valentin Cebotarescu^1^, Lilia Cojuhari^1^, Paulina Jimbei^2^

##### ^1^Faculty of Medicine, Nicolae Testemițanu State Medical and Pharmacy University, Chișinău, Republic of Moldova; ^2^Toma Ciorbă Clinical Hospital for Infectious Diseases, Chișinău, Republic of Moldova

###### **Correspondence:** Victor Pantea (victor.pantea@usmf.md)

**Background**

At the Clinical Hospital for Infectious Diseases, a study has been performed to evaluate antiviral and hepatoprotective effects of the medicinal product Viusid in treatment of viral hepatitis B and C.

**Methods**

The study involved 20 patients with chronic viral hepatitis: B - 10 patients (Group I), and C – 10 patients (Group II), among them in Group I – 7 men and 3 women, and in Group II – 4 and 6, respectively. The patient examination included physical exam, biochemistry tests (ALT, AST, GGT), complete blood count, molecular biology assays (HBV DNA and HCV RNA quantification by PCR). Viusid was prescribed as follows: 1 sachet 3 times daily (every 8 hours) for 3 months. The patient physical examinations were performed monthly, and the laboratory tests – at the beginning and at the end of treatment.

**Results**

Clinical manifestations were scanty in both groups: right hypochondriac pain in 44.4 % of cases in Group I, and 36.4 % – in Group II, weakness or fatigue – 22.2 % in Group I, 9.1 % – in Group II. At the end of treatment no clinical manifestations were present. Complete blood count, leukocyte count remained in the normal range with nonsignificant evolution. Lymphocyte count trended to increase within the normal range. Platelet count did not change significantly. Biochemistry tests: positive evolution of both total and direct bilirubin levels with their normalization in both groups was observed. ALT, AST and GGT were increased in all patients of both groups. In Group II, the average values of these parameters tended to reduce. In patients of Group I, the reduction was more significant than in those of Group II. Molecular biology assays. The HCV RNA level decreased from 442,843,736 IU/mL to 288,801,564 IU/mL, but did not become negative. The HBV DNA level reduced on average by 50 % (at the beginning of treatment – 4,729,167 IU/mL, and 1,833,933 IU/mL – at the end of treatment), and in 4 patients HBV DNA was not detected.

**Conclusions**

The treatment of both patient groups with Viusid showed that the clinical and biochemical improvement that was more apparent in Group I, which indicates the protective effect of the medicinal product Viusid; the reduction of HBV DNA level by 50 % and HCV RNA level by 40 %, denotes an antiviral effect of the drug.

### A27 The management of hyperbilirubinemia in HCV cirrhotic patients who underwent therapy with direct acting antivirals

#### Cristina Popescu^1,2^, Anca Leuștean^1^, Cristina Dragomirescu^1^, Alina Orfanu^1,2^, Cristina Murariu^1^, Laurențiu Stratan^1^, Alexandra Badea^1^, Cătălin Tilișcan^1,2^, Daniela Munteanu^1,2^, Raluca Năstase^1^, Violeta Molagic^1,2^, Mihaela Rădulescu^1,2^, Remulus Catană^1,2^, Victoria Aramă^1,2^

##### ^1^National Institute for Infectious Diseases “Prof. Dr. Matei Balș”, Bucharest, Romania; ^2^Carol Davila University of Medicine and Pharmacy, Bucharest, Romania

###### **Correspondence:** Cristina Popescu (crispopescu3@yahoo.com)

**Background**

The FDA warned that the direct acting antiviral (DAA) can cause severe liver injury in patients with advanced liver disease. However, liver decompensation was not reported in patients with HCV Child-Pugh A cirrhosis. In July 2016 a case of HCV Child-Pugh A patient treated with ombitasvir-paritaprevir/ritionavir-dasabuvir (OPrD) with severe liver decompensation was reported. The jaundice during OPrD therapy was reported especially in HIV-HCV co-infected patients. A guideline regarding the management of these patients has not been published yet. Objective: to analyze the predictive factors for hyperbilirubinemia during DAA therapy for HCV Child-Pugh A cirrhosis and also to establish the management of these patients.

**Methods**

This is a prospective study of patients with HCV genotype 1 Child-Pugh A cirrhosis, treated with OPrD-ribavirin regimen, in the Third Department of Matei Balș Institute. We analyzed the patients who developed hyperbilirubinemia during antiviral therapy in order to identify the risk factors for this side effect. The management of these patients was also analyzed. The statistical analysis was made with open-epi 3.0 program.

**Results**

Eighty-seven patients with HCV compensated cirrhosis are treated in our department with OPrD-ribavirin regimen. Three patients discontinued the antiviral therapy, two of them because of liver decompensation. After one month of therapy, 20 patients had total bilirubin more than 2 mg/dL and 7 of them had total bilirubin more than 4 mg/dL (the maxim value was 21 mg/dL). In the same time, these patients developed anemia and 16 of them permanently discontinued ribavirin. Five patients had high value of bilirubin (more than 10 mg/dL): one patient with predominance of unconjugated bilirubin and severe anemia (with hemolytic mechanism with recovery after ribavirin discontinuation and 2 patients with liver decompensation (with discontinuation of DAA regimen). Three of these patients did not develop liver decompensation and a slow recovery after discontinuation of ribavirin was observed. The risk factors for hyperbilirubinemia were analyzed and two of them were highly correlated with this side effect: Child-Pugh score at baseline 6 (RR 8 (4.48; 14.28) with p < 0.0000001) and baseline level of platelet count less than 100000/cmm (RR 5.36 (1.973; 14.56) with p < 0.0001).

**Conclusions**

Hyperbilirubinemia in patients with compensated cirrhosis treated with OPrD-ribavirin regimen represents a severe side effect. Ribavirin must be discontinued in this situation and sometimes all DAA regimen has to be withdrawn. The most important risk factors for this side effect are: Child-Pugh score at baseline 6 and platelet count at baseline below 100000/cmm.

### A28 The efficacy of ombitasvir-paritaprevir/ritonavir, dasabuvir and ribavirin in patients with genotype 1 HCV compensated cirrhosis

#### Cristina Popescu^1,2^, Laurențiu Stratan^1^, Remulus Catană^1,2^, Anca Leuștean^1^, Cristina Dragomirescu^1^, Alexandra Badea^1^, Cristina Murariu^1^, Raluca Năstase^1^, Violeta Molagic^1^, Daniela Munteanu^1,2^, Cătălin Tilișcan^1,2^, Mihaela Rădulescu^1,2^, Alina Orfanu^1,2^, Ioan Diaconu^1,2^, Anca Negru^1,2^, Iulia Bodosca^1^, Violeta Niță^1^, Victoria Aramă^1,2^

##### ^1^National Institute for Infectious Diseases “Prof. Dr. Matei Balș”, Bucharest, Romania; ^2^Carol Davila University of Medicine and Pharmacy, Bucharest, Romania

###### **Correspondence:** Cristina Popescu (crispopescu3@yahoo.com)

**Background**

The Romanian National Health System has approved the use of direct acting antivirals (DAA) for treatment of HCV compensated cirrhosis. The approved regimen contains a protease inhibitor, paritaprevir (boosted with ritonavir), a NS5A inhibitor - ombitasvir and a non-nucleoside NS5A inhibitor – dasabuvir (OPrD), recommended for 12 weeks in genotype 1b and for 24 weeks in genotype 1a. This DAA regimen is associated with ribavirin. Objective: to evaluate the real life data regarding the efficacy of this regimen in genotype 1 HCV infected patients with compensated cirrhosis.

**Methods**

We performed a prospective analysis of all patients with HCV compensated cirrhosis treated in Third Department of Matei Balș Institute since November 2015 until July 2016. We included all patients who underwent at least four weeks of antiviral therapy. We analyzed the evolution of clinical and biological parameters and also the trend of HCV viral load.

**Results**

Until now 88 of our patients received approval for OPrD therapy, 87 for compensated cirrhosis and one with F3 fibrosis and severe depression. A patient experienced liver decompensation during the third month of therapy, when cholangiocarcinoma was diagnosed, and died 2 weeks later. Our patients are in different stages of therapy: 37 patients at the end of follow-up (EF) – 42.52 %, 30 patients at the end of therapy (EOT) – 34.48 %, 5 patients during the third month of therapy – 5.74 %, 10 patients during the second month of therapy – 11.49 % and 5 patients during the first month. From the 27 patients whose viral load has been analysed after 4 weeks of therapy, 24 registered undetectability (88.88 %) and 2 patients had a viral load under the low limit of quantification. 66 patients finished the therapy and all had undetectable viral load. 38 patients finished the 12 weeks monitoring period and all of them registered sustained virologic response. Two patients prematurely discontinued antiviral therapy due to liver decompensation and cardiac disorders. From 53 patients with abnormal ALT at baseline, 32 (60.37 %) had normal ALT after 2 weeks of therapy and 50 (94.33 %) patients had normal ALT after 4 weeks of therapy.

**Conclusions**

The OPrD–Riba regimen was highly efficient in difficult to treat patients with compensated cirrhosis. All the patients that have completed the therapy achieved undetectable viral load and furthermore, in the case of those who completed the 12 weeks period of follow-up, SVR 12 was achieved.

### A29 The efficacy of direct acting antivirals regimen without ribavirin in HCV genotype 1b infected patients with compensated cirrhosis

#### Anca Leuștean^1^, Victoria Aramă^1,2^, Alina Orfanu^1,2^, Remulus Catană^1,2^, Laurențiu Stratan^1^, Cristina Dragomirescu^1^, Cristina Murariu^1^, Alexandra Badea^1^, Cătălin Tilișcan^1,2^, Daniela Munteanu^1,2^, Violeta Molagic^1,2^, Raluca Năstase^1^, Mihaela Rădulescu^1,2^, Cristina Popescu^1,2^

##### ^1^National Institute for Infectious Diseases “Prof. Dr. Matei Balș”, Bucharest, Romania; ^2^Carol Davila University of Medicine and Pharmacy, Bucharest, Romania

###### **Correspondence:** Anca Leuștean (anca_Leustean@yahoo.com)

**Background**

The regimen approved for the therapy of HCV Child Pugh A cirrhosis, contained at the beginning different combinations of direct acting antivirals and ribavirin. Some recent studies have shown that the association of ribavirin did not increase the efficacy of the regimen. Recently, in some countries the local protocols for therapy of HCV compensated cirrhosis recommend only DAA without ribavirin. Most of the available data about the importance of ribavirin in the therapy of patients with compensated cirrhosis came for clinical studies and data from real life will be very useful. Objective: to analyze the efficacy of ombitasvir-paritaprevir/ritonavir –dasabuvir (OPrD) without ribavirin in patients with HCV Child Pugh A cirrhosis.

**Methods**

This is a prospective analysis of the OPrD regimen efficacy without ribavirin in patients with compensated HCV cirrhosis, monitored in Third Department of Matei Balș Institute.

**Results**

Between November 2015 and July 2016, 87 patients received approval for OPrD and ribavirin therapy for HCV compensated cirrhosis, 86 for genotype 1b and one patient with genotype 1 with undetermined subtype. Some of our patients had contraindications for ribavirin usage (chronic anemia – 2 patients) and we also have patients with prematurely discontinuation of ribavirin. 85 patients started ribavirin, but after one month of antiviral therapy, the ribavirin was discontinued for 16 patients (18.82 %) and for 23 (27.05 %) patients the dose was reduced. The most important reason for ribavirin discontinuation and dose reduction was severe anemia but we also have patients with moderate or mild anemia but with severe jaundice (7 patients with total bilirubin more than 4 mg/dL – among them, 5 patients had bilirubin more than 10 mg/dL). After two more months of therapy, other 7 patients discontinued ribavirin. Out of 81 patients who received at least 2 months of therapy, 23 patients discontinued ribavirin (28.39 %) and for 20 patients the ribavirin dose was reduced (24.69 %). Only 38 patients received full dosage of ribavirin for at least two months. Despite the ribavirin dose reduction or discontinuation all the patients who completed 12 weeks of therapy achieved undetectable viral load and all patients who completed the follow-up period achieved sustained virologic response.

**Conclusions**

The efficacy of OPrD regimen in patients with HCV compensated cirrhosis is similar with or without ribavirin. Because sometimes the ribavirin side effects can conduct to a prematurely discontinuation of all antiviral regimen, we thought that in difficult to treat patients, the regimen without ribavirin could be a better option.

### A30 Liver decompensation during ombitasvir-paritaprevir/ritonavir-dasabuvir and ribavirin regimen in HCV infected patients with Child-Pugh A cirrhosis

#### Cristina Popescu^1,2^, Cristina Dragomirescu^1^, Anca Leuștean^1^, Cristina Murariu^1^, Laurențiu Stratan^1^, Alexandra Badea^1^, Remulus Catană^1,2^, Alina Orfanu^1,2^, Raluca Mihaela Năstase^1^, Violeta Molagic^1,2^, Daniela Munteanu^1,2^, Cătălin Tilișcan^1,2^, Victoria Aramă^1,2^

##### ^1^National Institute for Infectious Diseases “Prof. Dr. Matei Balș”, Bucharest, Romania; ^2^Carol Davila University of Medicine and Pharmacy, Bucharest, Romania

###### **Correspondence:** Cristina Dragomirescu (dragomirescu.cristina@yahoo.com)

**Background**

Patients with HCV cirrhosis need urgent antiviral therapy. However, the patients with liver cirrhosis represent difficult to treat cases and appropriate monitoring is necessary. The most important data regarding the safety of ombitasvir-paritaprevir/ritonavir-dasabuvir (OPrD) and ribavirin regimen in HCV cirrhotic patients came from Turquoise II clinical trial, real life data being lacunar. According to Romanian guideline and also with summary of product characteristics, this regimen is recommended only in Child A cirrhosis. Objective: To analyze the risk of liver decompensation during OPrD-ribavirin regimen in HCV Child-Pugh A cirrhotic patients.

**Methods**

We performed a prospective study of HCV Child A cirrhotic patients monitoring in Third Department of Matei Balș Institute who developed liver decompensation during OPrD therapy. We correlated the liver decompensation with some clinical and biological characteristics at baseline.

**Results**

Eighty seven Child A cirrhotic patients were treated in our Department: 70 patients had 5 points at Child score (80.45 %) and 17 patients 6 points (19.55 %). Five patients (5.74 %) developed liver decompensation during antiviral therapy. Two patients permanently discontinued antiviral therapy: one after 23 days of therapy − because after the discontinuation of ribavirin and supportive therapy the outcome wasn’t good and the second one was diagnosed with cholangiocarcinoma after 9 weeks of therapy. Two patients with liver decompensation had a good outcome after cessation of ribavirin and supportive therapy. They had completed the therapy with OPrD and achieved SVR12. One patient is still in hospital under strict monitoring; ribavirin was stopped but OPrD regimen was not yet discontinued. The mean age was 63 year-old, 3 male and 2 female, 3 naive patients and 2 previously treated with null response. All the patients had Child score 6. All the patients had at baseline: abnormal INR (but less than 1.7 – the limit accepted by Child Pugh score), platelet count under 100000/cmm, mild increase of total bilirubin (between 2 and 3 mg/dL for 4 patients and below 2 mg/dL for one patient) and albumin below 3.5 g/dL in one patient. Four patients had esophageal varices at baseline and all patients had an increased spleen diameter.

**Conclusions**

Liver decompensation in patients with Child Pugh score A during OPrD-ribavirin regimen has a low rate of probability, but this situation is possible. The diagnosis of compensated cirrhosis probably has to take into account more clinical and biological parameters, not only the ones used by Child Pugh score.

### A31. The safety of direct acting antivirals in HCV compensated cirrhotic patients - an interim analysis

#### Victoria Aramă^1,2^, Remulus Catană^1,2^, Cristina Dragomirescu^2^, Cristina Murariu^2^, Anca Leuștean^2^, Laurențiu Stratan^2^, Alexandra Badea^2^, Alina Orfanu^1,2^, Anca Negru^1,2^, Raluca Năstase^2^, Violeta Molagic^2^, Daniela Munteanu^1,2^, Cătălin Tilișcan^1,2^, Mihaela Rădulescu^1,2^, Ioan Diaconu^2^, Violeta Niță^2^, Iulia Bodoșca^2^, Cristina Popescu^1,2^

##### ^1^Carol Davila University of Medicine and Pharmacy, Bucharest, Romania; ^2^National Institute for Infectious Diseases “Prof. Dr. Matei Balș”, Bucharest, Romania

###### **Correspondence:** Remulus Catană (catana.remulus@yahoo.com)

**Background**

The regimen containing NS5A inhibitor - ombitasvir, protease inhibitor paritaprevir boosted with ritonavir and non-nucleoside inhibitor dasabuvir (OPrD) associated with ribavirin was approved in Romania from November 2015 for genotype 1 HCV infected patients with compensated cirrhosis. The safety data regarding this therapeutic regimen came from clinical studies where many patients with severe comorbidities were excluded. The data coming from real-life are more relevant in this context. Objective: the aim of our study is to analyze and to report the side effects that occurred during and after OPrD-riba regimen and also the management of these side effects.

**Methods**

We performed a prospective study using the database of cirrhotic patients treated with OPrD-riba regimen in Third Department of Matei Balș Institute. All the adverse events that occurred in these patients were introduced into a database and we established the correlation between the regimen and each side effect, the grade of each side effect and also its management.

**Results**

A total of 87 patients were followed, with a median age of 63 years (IQR 54–70 years) and 47 % males. 36 patients (41.4 %) reported at least one clinical adverse event. The most common were fatigue (26.4 %), pruritus (13.8 %), dizziness (8 %), sleeping disorders (6.9 %), nausea and/or vomiting (6.9 %), muscle and/or bone pain (4.6 %), headache (3.4 %), diarrhoea (3.4 %) and skin rash (2.3 %). The main laboratory abnormalities were anemia (44.8 %) and hyperbilirubinemia (23 %). After the first month of treatment, 20 patients (23 %) developed mild anemia (hemoglobin level 11–12 g/dL) and 19 (21.8 %) developed moderate anemia (hemoglobin level < 11 g/dL). A total bilirubin level > 2 mg/dL after one month of therapy was observed in 20 patients (23 %) and for 16 (18.4 %) of them ribavirin was discontinued. Three patients discontinued treatment, two of them because of liver decompensation.

**Conclusions**

The most important side effect was anemia which was correlated with ribavirin use and for some cases ribavirin discontinuation was necessary. Jaundice was another side effect more difficult to control. Complete therapy discontinuations due to adverse events were infrequent.

### A32 The access of patients with HCV compensated cirrhosis to the National Program of therapy with direct acting antivirals

#### Cristina Popescu^1,2^, Alexandra Badea^1^, Anca Leuștean^1^, Alina Orfanu^1,2^, Anca Negru^1,2^, Laurențiu Stratan^1^, Cristina Dragomirescu^1^, Remulus Catană^1,2^, Cristina Murariu^1^, Violeta Molagic^1,2^, Raluca Năstase^1^, Cătălin Tilișcan^1,2^, Daniela Munteanu^1,2^, Mihaela Rădulescu^1,2^, Ioan Diaconu^1,2^, Violeta Niță^1^, Iulia Bodoșca^1^, Victoria Aramă^1,2^

##### ^1^National Institute for Infectious Diseases “Prof. Dr. Matei Balș”, Bucharest, Romania; ^2^Carol Davila University of Medicine and Pharmacy, Bucharest, Romania

###### **Correspondence:** Alexandra Badea (alexandrambadea@yahoo.com)

**Background**

The Romanian patients known with genotype 1 HCV compensated cirrhosis have access to direct acting antivirals (DAA) therapy since November 2015 for free, through a National Program financed by Romanian Health Insurance. The eligibility criteria for DAA regimen were: genotype 1 of HCV, detectable viral load, cirrhosis diagnosed by FibroMax (BioPredictive France) if fibrotest is more than 0.75 and compensated cirrhosis according to Child Pugh score (Child Pugh score A – 5 and 6 points). Objectives: to analyze all the causes that led to the failure of access to DAA regimen via Romanian National Program.

**Methods**

We performed a prospective study in which we enrolled all the patients known with compensated cirrhosis who received vouchers for access to the therapy (FibroMax, viral load and HCV genotyping test). The current status of each patient was analyzed.

**Results**

120 patients were included in the DAA therapy program in Third Department of Matei Balș Institute. Among them: 88 (78.33 %) received the approval, 17 patients are awaiting the approval (14.16 %), 3 patients were ineligible despite F4 fibrosis due to the diagnosis of hepatocellular carcinoma and 12 (10 %) had fibrosis less than F4 and were ineligible according to the local guideline. From our patients only 92 (76.66 %) had F4 fibrosis according to the FibroMax. In 4 cases the previous fibrosis investigated by FibroMax or Fibroscan was F3 and the patients had severe comorbidities. Despite these data, the evaluation of FibroMax during the National Program showed F2 fibrosis and were ineligible for DAA therapy. In one case, the result of FibroMax was F2 but the patient had significant clinical signs of cirrhosis and the therapy was approved. For twenty-two patients the FibroMax showed F3 fibrosis (19.16 %). However, they were known with compensated cirrhosis previously diagnosed by: FibroMax, Fibroscan, liver biopsy or by clinical findings like esophageal varices. Among them, 15 patients were considered eligible for therapy (65.21 %): 11 patients have already received the approval (78.57 %) and 4 patients are awaiting the commission’s decision. Eight patients without clinical signs of cirrhosis were declared ineligible (34.78 %), despite the previous evaluation of fibrosis by non-invasive methods.

**Conclusions**

The fibrosis cannot be always correctly determined by FibroMax; it is important to use other alternative test for an accurate diagnosis of cirrhosis. Moreover, even the tests manufacturer from BioPredictive recommends that a fibrotest score with a value more than 0.60 can be interpreted as severe fibrosis and must be treated urgently.

### A33 Severe reactivation of chronic hepatitis B after discontinuation of nucleos(t)ide analogues – a case series

#### Cristina Popescu^1,2^, Alina Orfanu^1,2^, Anca Leuștean^1^, Alexandra Badea^1^, Laurențiu Stratan^1^, Remulus Catană^1,2^, Cătălin Tilișcan^1,2^, Victoria Aramă^1,2^

##### ^1^National Institute for Infectious Diseases “Prof. Dr. Matei Balș”, Bucharest, Romania; ^2^Carol Davila University of Medicine and Pharmacy, Bucharest, Romania

###### **Correspondence:** Alina Orfanu (alina.lobodan@yahoo.com)

**Background**

Nucleos(t)ide analogues (NAs) realize a proper suppression of viral replication in chronic hepatitis B (HBV), but a negligible immune control, so a lifelong therapy is necessary. The highest risk after therapy discontinuation, even in patients who achieved undetectable viral load (VL), is the viral reactivation. Reactivation flares appear in 10 % of cases after therapy cessation and are associated with jaundice, hepatocytolysis and high VL. Some cases can develop fulminant hepatitis with high mortality rate.

**Methods**

We present a series of 3 cases of viral reactivation following discontinuation of Entecavir (ETV), administered for chronic HBV.

**Results**

In 2015–2016, three patients known with chronic HBV were admitted in our department for jaundice and ALT increase. The first case is a young woman, pregnant in 24 weeks, under ETV for 4 years, with negative HBeAg and undetectable VL, who decided to stop therapy when she discovered the pregnancy. Six months later she was admitted in our clinic for important hepatocytolysis. The biological exams revealed: ALT > 20 x upper limit of normal (ULN), positive HBeAg, HBV VL of 9 log IU/mL, normal prothrombin concentration and mild hyperbilirubinemia. The patient received off label lamivudine with slow decrease of ALT and VL of 2 log IU/mL at delivery. ETV therapy was reintroduced after delivery, with favorable outcome. The second case is a young man who discontinued ETV because he lost his medical insurance. During antiviral therapy he had normal ALT and undetectable VL. Five months later, he presented ALT 5xULN, jaundice and high VL. The patient renewed his insurance and ETV was reinitiated, with good outcome. The last patient, a 28 year-old man is still hospitalized. He was under ETV for 6 years with good biological outcome, after a previous therapy with peginterferon. In January 2016, he stopped ETV by himself and in August he was admitted in our clinic for jaundice and vomiting. The tests showed ALT 60xULN, hyperbilirubinemia (16 mg/dL), positive HBeAg and decrease of prothrombin concentration and fibrinogen. The therapy with tenofovir was immediately started and after 5 days, the prothrombin concentration became normal, but ALT and bilirubin remained increased.

**Conclusions**

NAs therapy must be continued until HBs seroconversion, even in patients who achieved undetectable VL. The risk of severe liver decompensation especially in young people is extremely high. In women with childbearing potential, ETV can be switched with tenofovir and the therapy must be continued during the entire pregnancy.

**Consent**

Written informed consent was obtained from the patients for publication of this Case report and any accompanying images. A copy of the written consent is available for review by the Editor of this journal.

### A34 The dynamic of hematological disorders during direct acting antivirals therapy for HCV compensated cirrhosis

#### Cristina Popescu^1,2^, Cristina Murariu^1^, Cristina Dragomirescu^1^, Anca Leuștean^1^, Laurențiu Stratan^1^, Alina Orfanu^1,2^, Alexandra Badea^1^, Remulus Catană^1,2^, Anca Negru^1,2^, Cătălin Tilișcan^1,2^, Daniela Munteanu^1,2^, Mihaela Rădulescu^1,2^, Violeta Molagic^1,2^, Raluca Mihaela Năstase^1^, Ioan Alexandru Diaconu^1,2^, Iulia Bodoșca^1^, Violeta Niță^1^, Victoria Aramă^1,2^

##### ^1^National Institute for Infectious Diseases “Prof. Dr. Matei Balș”, Bucharest, Romania; ^2^Carol Davila University of Medicine and Pharmacy, Bucharest, Romania

###### **Correspondence:** Cristina Murariu (murariucarina@yahoo.com)

**Background**

The regimen approved in Romania for the patients with HCV compensated cirrhosis involves Ombitasvir-Paritaprevir/Ritonavir-Dasabuvir (OPrD) in association with ribavirin. The most important side effect, during ribavirin based therapy, is anemia, well-known from the era of Peginterferon-ribavirin regimen. Objective: to analyze the hematological disorders occurred during OPrD – ribavirin therapeutic regimen for HCV compensated cirrhosis.

**Methods**

**P**rospective study of the HCV cirrhotic patients treated with OPrD-ribavirin regimen from November 2015 until July 2016 in Third Department of Matei Balș Institute which analyzed the dynamic of hemoglobin level and platelet count during 12 weeks of DAA therapy.

**Results**

Eighty-seven patients with HCV compensated cirrhosis were treated in our department. The mean age was 61.93 years old and sex ratio F:M = 1.12:1. After one month of therapy, 19 patients (21.83 %) developed moderate anemia with hemoglobin below 11 g/dL (between 7.8 g/dL and 10.8 g/dL, with a medium value of 9.8 g/dL). Sixteen of these patients permanently discontinued ribavirin during first month of antiviral therapy and two patients permanently discontinued all therapeutic regimen: one patient for severe cardiac disturbances and the other for liver decompensation with important jaundice. For other 3 patients the dosage of ribavirin was reduced. For 20 patients, hemoglobin level after first month of therapy was between 11 and 12 g/dL (mild anemia – 22.98 %) and because of severe fatigue, the dosage of ribavirin was reduced. After 2 months of therapy from 81 patients who reached this endpoint, other 7 patients permanently discontinued ribavirin due to moderate anemia (below 11 g/dL). 37/67 (55.22 %) of patients who completed the therapy had anemia despite the reduction or discontinuation of ribavirin. 37 patients finished the monitoring therapy (SVR12) and all the patients who developed anemia had normal level of hemoglobin. Regarding thrombocytopenia, it was improved during antiviral therapy. The analysis was performed for 67 patients who finished the therapy. At baseline, 13 patients had severe thrombocytopenia (19.40 %) and 38 patients had platelet count more than 150000/cmm. At the end of treatment, 6 patients remained with severe thrombocytopenia (8.9 %) and 53 patients had platelet count more than 150000/cmm (79.1 %). The statistical analysis showed significant increases of platelet count.

**Conclusions**

The most frequent hematological side effect during OPrD-riba therapy for HCV hepatitis was anemia with recovery after adjusting the ribavirin dosage. Sometimes, the discontinuation of ribavirin was necessary, without impact in efficacy of this regimen. In terms of platelet count a significant improvement was shown.

### A35 Behaviors, attitudes and risk factors for viral hepatitis in international medical students vs. the general population in Romania

#### Yagmur Erturk^1^, Oana Săndulescu^1,2^, Alina Cristina Neguț^1,2^, Claudiu Mihai Șchiopu^2^, Adrian Streinu-Cercel^1,2^, Anca Streinu-Cercel^1,2^

##### ^1^Carol Davila University of Medicine and Pharmacy, Bucharest, Romania; ^2^National Institute for Infectious Diseases “Prof. Dr. Matei Balș”, Bucharest, Romania

###### **Correspondence:** Yagmur Erturk (yagmur_910@hotmail.com)

**Background**

The prevalence of risk factors, behaviors and knowledge on HBV and HCV infection varies in different areas of the world. We have performed a study to determine whether classical risk factors for HBV or HCV showed diversity due to different ethnic or geographic origins in two matched young cohorts: a heterogeneous study group of international medical students, and a control group from the general Romanian population.

**Methods**

We have performed a cross sectional study based on a standardized questionnaire and serologic tests to assess the prevalence of risk factors for transmission of viral hepatitis in two study groups. The Z-test was used for checking the statistical significance of the proportions of specific risk factors in the two groups.

**Results**

The study included 371 participants (77 in the study group and 294 in the control group). The median age of the study group was 25 years (interquartile range [IQR]: 24, 26.5 years), and 23 years (IQR: 22, 24 years) in the control group. The male-to-female ratio was 0.9:1 in the study group vs. 1:1 in the control group. The following risk factors for HBV or HCV transmission were significantly more prevalent in the study group: blood transfusion or organ transplantation after 1992 (p = 0.029), history of coagulopathy (p = 0.048), injected drug use (p = 0.048), having lived with someone with hepatitis B (p = 0.005), history of travel to countries with high HBV risk (p < 0.001), never having undergone screening for hepatitis (p < 0.001) or HIV (p < 0.001), history of unprotected oral (p = 0.004) or vaginal (p = 0.004) intercourse with more than one partner in the last year, occupational exposure to HIV/hepatitis (p < 0.001 each), tattoos or piercing (p < 0.001). However, the study group also presented positive health-related behaviors, such as: history of vaccination against hepatitis A (p < 0/001) or hepatitis B (p = 0.001), a previous negative result upon screening for hepatitis (p < 0.001) or HIV (p < 0.001).

**Conclusions**

The worldwide prevalence of certain risk factors for HBV and HCV infection is a serious issue that transcends nationality, gender, ethnic origin or educational level. Our results suggest that a global strategy for prevention and control of hepatitis infection is needed, along with effective vaccination programs.

**Acknowledgements**

1) This abstract is part of the license thesis “Hepatitis risk factors distribution in a young cohort” performed at Carol Davila University of Medicine and Pharmacy, Bucharest, Romania. Coordinator: Dr. Anca Streinu-Cercel, MD, PhD; Supervisor: Dr. Oana Săndulescu, MD, PhD. 2) “Give screening a chance in HCV: GCS-HCV” project – Janssen.

### A36 Characteristics of hepatitis C virus reactivation due to immunosuppressive therapy in Romanian HCV infected patients with hematological malignancies

#### Violeta Molagic^1^, Cătălin Tilișcan^1,2^, Cristina Popescu^1,2^, Raluca Mihăilescu^1^, Daniela Munteanu^1^, Raluca Năstase^1^, Anca Negru^1,2^, Angelica Tenita^1^, Victoria Aramă^1,2^, Ștefan Sorin Aramă^2^

##### ^1^National Institute for Infectious Diseases “Prof. Dr. Matei Balș”, Bucharest, Romania; ^2^Carol Davila University of Medicine and Pharmacy, Bucharest, Romania

###### **Correspondence:** Violeta Molagic (violeta_molagic@yahoo.com)

**Background**

Data on hepatitis C virus reactivation (HCV-R) in patients with hematological malignancies (HM) under immunosuppressive therapy are limited. The prevalence of HCV infection is higher in Romania than in other EU countries. We aimed to evaluate clinical characteristics and outcome of HCV–R in patients with HM undergoing immunosuppressive therapy.

**Methods**

We performed a prospective study that include patients with HM treated with rituximab containing chemotherapy and anti HCV positive. The patients were monitored in the 3^rd^ department of the Prof. Dr. Matei Balș National Institute for Infectious Diseases, Bucharest, Romania, between 2008–2014. Anti HCV, HCV-RNA (by PCR) and biochemical tests were measured at baseline and every three months. Viral reactivation was defined as ≥1log 10 IU/mL increase of HCV-RNA following chemotherapy.

**Results**

We enrolled 30 HCV-infected patients: 8 (26.7 %) males and 22 (73.3 %) females, with mean age 66 ± 9.44 years. Most cases (83.3 %) had non-Hodgkin lymphomas (NHLs), 10 % chronic lymphocytic leukemia (CLL), 3.3 % Hodgkin lymphoma (HL) and 3.3 % Waldenström macroglobulinemia (WM). Based on histologic features the HM indolent subtype was predominant (53.3 %) versus aggressive subtype (46.7 %). Advanced fibrosis (stage F3-F4) was present in 68.2 % of patients and 31.8 % had moderate or severe necroinflammatory activity. Nine patients (30 %) had high HCV-RNA viral loads (>600 000 IU/mL) at baseline. HCV-R was present in 30 % of cases, after a mean of 4 months (range: 3–12 months) from enrolling. A hepatic flare was associated only in 3 of these cases. Three (10 %) patients died, one due to HCV associated fulminant hepatitis and two due to HM complications.The median HCV viral load at baseline was 507525.00 IU/mL (IQR = 3074957 IU/mL) in HCV-R patients versus 471129.00 IU/mL (IQR = 1562720 IU/mL) in patients without HCV-R. Neither sex (p = 0.23), age (p = 0.13), HCV-RNA levels at baseline (p = 1), presence of fibrosis (p = 1), necroinflammatory activity or HM type significantly influenced the risk of HCV-R.

**Conclusions**

One third of our patients had HCV-R. HCV can cause fulminant hepatic failure in patients with HM. Close monitoring of HCV-RNA every 3 months should be performed.

**Acknowledgement**

Study supported by SOP HRD financed from ESF and RG under contract number POSDRU/187/1.5/S/155420.

### A37 The dynamic IFN-gamma serum levels during successful peginterferon-a 2a/ribavirin therapy in HCV chronic infection

#### Simona Alexandra Iacob, Diana Gabriela Iacob, Monica Luminos

##### National Institute for Infectious Diseases “Prof. Dr. Matei Balș”, Bucharest, Romania

###### **Correspondence:** Simona Alexandra Iacob (simonaaiacob@yahoo.com)

**Background**

Interferon gamma (IFN γ) plays a key role in the elimination of hepatitis C virus during the first 8–12 weeks following infection. Once the infection becomes chronic the role of IFN γ becomes unclear and the increased levels of this cytokine are not associated with a favorable evolution. The current study aims to analyze the level of IFN γ in sustained responders on standard peginterferon-α 2a/ribavirin therapy.

**Methods**

The study employed 40 assessments of the serum IFN γ level in 5 patients between 25–36 years hospitalized in the National Institute for Infectious Diseases, Bucharest, Romania with chronic HCV infection, genotype 1 and F2 hepatic fibrosis who received treatment with peginterferon-α 2a 180 mcg weekly and ribavirin 1000–1200 mg/day. All patients showed undetectable HCV RNA viral loads after the first 3 months of treatment and remained undetectable over the next 2 years of follow-up. The study performed a comparative analysis (before/after treatment) on the serum levels of IFN γ (ELISA, Bio Scientific) in the first 6 months of interferon α treatment.

**Results**

All patients showed a constant evolution of IFN ɣ levels with a moderate increase of IFN γ from 59 to 200 pg/mL during the first 6 months of treatment. Interestingly, all patients with a recent infection (<1 year) showed much higher pre-therapy serum levels of IFN γ (984–1282 pg/mL) than those with a more distant infection (IFN ɣ serum levels 120–171 pg/mL).

**Conclusions**

HCV patients with a sustained response to peg interferonα/ribavirin therapy display constant levels of IFN γ during the first 6 months of treatment. The level of IFN γ in recent HCV infections was much higher compared with more distant infections. The pre-therapy level of serum IFN ɣ did not influence the virologic outcome to the standard interferon α therapy.

### A38 Overlapping risk factors for transmission of HBV, HCV and HIV in the general population in Romania

#### Anca Streinu-Cercel^1,2^, Oana Săndulescu^1,2^, Mioara Predescu^2^, Alexandra Mărdărescu^3^, Cătălin Tilișcan^1,2^, Mihai Săndulescu^1^, Claudiu Mihai Șchiopu^2^, Adrian Streinu-Cercel^1,2^

##### ^1^Carol Davila University of Medicine and Pharmacy, Bucharest, Romania; ^2^National Institute for Infectious Diseases “Prof. Dr. Matei Balș”, Bucharest, Romania; ^3^Regional HIV/AIDS Center INBI “Prof. Dr. Matei Balș”, Bucharest, Romania

###### **Correspondence:** Anca Streinu-Cercel (anca_sc@yahoo.com)

**Background**

Risk-reduction campaigns are essential parts of national health programs. To better select the population groups to target, we have undergone an assessment of specific risk factors for transmission of HBV, HCV and HIV infection in Romania.

**Methods**

We implemented a nationwide screening program for chronic viral infections, which also included a standardized questionnaire for assessing the prevalence of risk factors for HBV, HCV or HIV transmission in the general population in Romania. The statistical analysis was performed with IBM SPSS Statistics version 22 (Armonk, USA).

**Results**

We analyzed a significant sample from the general population in Romania, with a median (interquartile range) age of 46 (36, 57) years and a male to female ratio of 0.6:1. We identified a higher prevalence of risk factors in the male population, namely a history of sexually-transmitted infections (STIs – OR: 3.4, CI 95 %: 1.3, 10.0), piercing/tattoos (OR: 1.9 CI95 %: 1.4, 2.6), unprotected anal (OR: 2,6, CI95 %: 1.1, 6.0), vaginal (OR: 2.2, CI95 %: 1.5, 3.3) and oral (OR: 5.5, CI95 %: 2.9, 10.2) intercourse but not homosexual intercourse (p = 0.524), multiple sex partners (p = 0.403), injecting drug use (IDU – p = 0.205), or needle sharing (p = 0.613). The prevalence of IDU was significantly higher in patients who also had a history of STIs (OR: 372, CI95 %: 64, 2180), and the same was true for needle sharing (OR: 124, CI95 %: 26, 598). Similarly, piercings/tattoos were significantly associated with IDU (OR: 69, CI95 %: 14, 346), needle sharing (OR: 16, CI95 %: 4.5, 56.8), all types of unprotected intercourse: homosexual (OR: 10, CI95 %: 4.7, 21.2), oral (OR: 34, CI95 %: 19, 61), vaginal (OR:13, CI95 %: 8, 23), or anal (OR: 25, CI95 %: 10, 64), and multiple sex partners during the past 6 months (OR: 10, CI95 %: 7, 14).

**Conclusions**

Although the overall prevalence of risk factors in the general population in Romania appears to be low or under-declared, we have identified certain high-risk groups, which cumulate a large number of risk factors and have high likelihood of acquiring HBV, HCV or HIV infection. We propose priority targeting of these special population groups though information campaigns and risk reduction interventions.

**Acknowledgment**

This study is part of the RO 19.02. Project “Strengthening the prevention and control of HIV/AIDS, HBV, HCV in Romania”, financed by the Norway Financial Mechanism 2009–2014, “Public Health Initiatives”.

### A39 Acute hepatitis - an uncommon neurological complication

#### Cleo Nicoleta Roșculeț^1^, Catrinel Olimpia Ciuca^1^, Dalila Ana Toma^1^, Cătălin Gabriel Apostolescu^1^, Andrei Rogoz^1^, Cristina Elena Mitu^2^, Andrei Stangaciu^1^, Viorica Daniela Mitescu^1^, Tudor Gheorghe Vladoiu^1^, Doina Viorica Iovănescu^1^

##### ^1^National Institute for Infectious Diseases “Prof. Dr. Matei Balș”, Bucharest, Romania; ^2^Colentina Clinical Hospital, Bucharest, Romania

###### **Correspondence:** Cleo Nicoleta Roșculeț (cleo_n_rosculet@yahoo.com)

**Case report**

A 42 yo patient recently hospitalized in the Râmnicu Vâlcea Hospital for jaundice with immunoglobulin M antibodies against hepatitis A detected in blood is hospitalized in our institute for supervision and the continuation of the treatment. Recent medical history shows the onset of the symptoms 11 days before with fever, jaundice and the patient recognizes contact with a person with confirmed infection with hepatitis A. As a development of the disease, the patient became comatose (GCS 3), with severe respiratory acidosis, depending on advanced respiratory support. After 24 hours, he becomes conscious, but shows limp quadriplegia, facial asymmetry, thenar and hypothenar eminence atrophy, abolished tendinous reflexes and preserved sensitivity. The MRI reveals no alterations that could explain the neurological deficit, the lumbar puncture is within normal limits. The patient had a favorable biological trend with the correction of coagulation disorders and improvement of sepsis markers. The second lumbar puncture showed albuminocytologic dissociation. The findings of motor nerve conduction studies showed markedly reduced amplitudes of compound muscle action potentials and suggested demyelinating polyneuropathy. Based on Guillain-Barré syndrome diagnosis, there were conducted five sessions of plasma exchange and after each of them we noticed progressive improvement of the motor deficit, including of the respiratory muscles, therefore the respiratory support was ceased after 8 days.

**Conclusions**

Acute hepatitis with HAV can also trigger an immune response, therefore clinicians should consider this rare but serious possibility of Guillain-Barré syndrome following acute hepatitis A infection.

**Consent**

Written informed consent was obtained from the patient for publication of this Case report and any accompanying images. A copy of the written consent is available for review by the Editor of this journal.

### A40 Regression of liver fibrosis following sustained virological response in patients with chronic HCV infection and cirrhosis

#### Oana Săndulescu^1,2^, Anca Streinu-Cercel^1,2^, Monica Andreea Stoica^2^, Liliana Lucia Preoțescu^1,2^, Daniela Manolache^2^, Gabriela Jana Ceapraga^2^, Maria Magdalena Moțoi^2^, Luminița Bradu^2^, Adina Ilie^2^, Gabriela Mircea^2^, Ionel Durbală^2^, Adrian Streinu-Cercel^1,2^

##### ^1^Carol Davila University of Medicine and Pharmacy, Bucharest, Romania; ^2^National Institute for Infectious Diseases “Prof. Dr. Matei Balș”, Bucharest, Romania

###### **Correspondence:** Anca Streinu-Cercel (anca_sc@yahoo.com)

**Background**

Liver fibrosis was historically considered an irreversible and progressive process. However, recent data have shown that after obtaining sustained virological response (SVR) to anti-infectious treatment, liver stiffness starts to decrease, potentially returning back to normal post-infection values.

**Methods**

We have analyzed the evolution of liver fibrosis as assessed by shear-waves elastography on Aixplorer (SuperSonic Imagine, France) in patients with chronic HCV infection and cirrhosis, treated with the 3D regimen ombitasvir/paritaprevir/ritonavir plus dasabuvir (AbbVie, USA). Liver stiffness was determined at baseline, at the end of 12 weeks of treatment and at a follow-up visit at 12 weeks post-treatment. Data were analyzed using Wilcoxon signed rank test and Spearman's rank-order correlation in SPSS Statistics for Windows (v22.0, IBM Corp, USA).

**Results**

We present the results for 87 patients, with a male-to-female ratio of 0.9:1, a median (interquartile range – IQR) age of 58 (49.5, 64.5) years and a mean ± standard deviation duration of HCV evolution of 9.2 ± 4.9 years. Follow-up data is currently available for 60/87 patients (69 %) and all achieved sustained virological response. The median (IQR) liver stiffness at baseline was 13.9 (12.6, 19.2) kPa and it decreased significantly to 10.7 (7.8, 15.1) kPa at 12 weeks post-treatment (p = 0.002, Z = −3.146). The median decrease in liver stiffness was −1.8 (−3.1, −0.2) kPa over an interval of 24 weeks, and it was correlated with shorter evolution of HCV infection prior to starting treatment (rs = 0.346, p = 0.023).

**Conclusions**

Based on our results, we would recommend earlier therapy for chronic HCV infection, to ensure faster decrease of liver stiffness after treatment. Further long-term studies are needed to determine the exact pace at which liver fibrosis decreases past the point of SVR.

**Acknowledgement**

1) “Romanian Center for Applied Bio-Molecular Research in Infectious Diseases”, project financed through the Sectoral Operational Programme Increasing of Economic Competitiveness (POS CCE).

2) RO 19.02. Project “Strengthening the prevention and control of HIV/AIDS, HBV, HCV in Romania”, financed by the Norway Financial Mechanism 2009–2014, “Public Health Initiatives”.

### A41 Preliminary results for treatment with sofosbuvir and daclatasvir of patients with chronic hepatitis C

#### Irina Russu^1^, Tiberiu Holban^1^, Tatiana Pantilimonov^2^, Galina Chiriacov^2^, Arcadie Macvovei^2^, Elena Scorohodico^2^, Oleg Dmitriev^2^

##### ^1^Department of Infectious Diseases, Parasitology and Tropical Medicine, Nicolae Testemițanu State Medical and Pharmacy University, Chișinău, Republic of Moldova; ^2^Toma Ciorbă Clinical Hospital for Infectious Diseases, Chișinău, Republic of Moldova

###### **Correspondence:** Irina Russu (irina.rusu@usmf.md)

**Background**

It is estimated that about 103 million people around the world are infected with hepatitis C virus (HCV). Due to population screening for HCV and initiation of antiviral treatment, HCV prevalence decreased in some countries. The purpose of antiviral treatment is to eradicate HCV completely and obtain sustained virologic response (SVR). The bibliographical data confirms that over 99% of patients with SVR remain HCV RNA negative 4-5 years after stopping treatment [1].

**Methods**

We assessed 21 patients diagnosed with chronic HCV genotype 1b who received combination therapy with sofosbuvir plus daclatasvir (SOF/DCV) for 12 weeks. Patients with cirrhosis were not included in our study. The degree of fibrosis was established by Fibroscan and Fibromax.

**Results**

Out of 21 patients with chronic HCV, 18 were naive and 3 pre-treated with peg-IFN and ribavirin. 13 (62%) were men and 8 (38%) women with ages between 21 and 58 years. Most patients had a poor clinical picture like fatigue, right upper quadrant pain. 14 (66.6%) patients had moderate level of hepatic cytolysis (ALT average 188 U/L), bilirubin was within normal limits. HCV RNA detected prior to treatment ranged from 290.000 to 24 million copies/mL (average 3.8 million copies/mL). Degree of fibrosis - F0 was established in 2 patients, F1 - in 3, F2 - in 12 and F3 - in 4 patients. No adverse events occurred in any patient during treatment with SOF/DCV. At 4 weeks of antiviral therapy 20 (95.2%) showed undetectable viremia HCV-RNA, only one patient decreased from 8 million to 500.000 copies/mL. All 21 (100%) patients achieved ALT normal limits. At 12 weeks of treatment all 21 patients experienced virologic response (HCV RNA - undetectable). At 24 weeks after starting of treatment 13 (62%) patients had SVR (HCV RNA - undetectable). 8 patients are on the point of completing treatment with dynamic supervision to 24 weeks.

**Conclusions**

Our study shows a high rate of SVR after treatment with sofosbuvir and daclatasvir in naive as well as pre-treated patients with chronic HCV, and no significant side effects from taking these drugs.

Reference

1. Cornberg M, Höner zu Siederdissen C, Maasoumy B, Manns MP, Wedemeyer H. Standard therapy of chronic hepatitis C virus infection. In: Mauss S, Berg, T, Rockstroh J, Sarrazin C, Wedemeyer W, editors. Hepatology. A clinical textbook. 7th edition. Hamburg: Medizin Fokus Verlag; 2016. p.247.

## HIV infection - epidemiology, treatment and monitoring

### A42 HIV-syphilis coinfection

#### Diana Alexandra Costache^2^, Anca Benea^3^, Eliza Manea^1^, Cristian Niculae^1^, Raluca Jipa^1^, Adriana Hristea^1,2^, Elisabeta Benea^1,2^, Ruxandra Moroti^1,2^, Șerban Benea^1,2^

##### ^1^National Institute for Infectious Diseases “Prof. Dr. Matei Balș”, Bucharest, Romania; ^2^Carol Davila University of Medicine and Pharmacy, Bucharest, Romania; ^3^Clinical Hospital of Infectious and Tropical Diseases “Dr. Victor Babeș”, Bucharest, Romania

###### **Correspondence:** Șerban Benea (serbanb_16@yahoo.com)

**Background**

HIV and syphilis can be found in similar patient groups and the association of these two infections is becoming more frequent. The two diseases can influence each other’s evolution and the presence of one can facilitate infection with the other. In addition, the presentation, diagnosis, and management of syphilis differ in subtle ways between HIV-infected and HIV-uninfected patients. Objectives: Determining the prevalence of syphilis in newly diagnosed HIV patients and identifying epidemiological, clinical and laboratory aspects in those coinfected.

**Methods**

We performed a retrospective study in patients newly diagnosed with HIV infection in the National Institute for Infectious Diseases “Prof. Dr. Matei Balș” between the 1^st^ of January 2015 and 30^th^ of June 2016. Patients were tested for syphilis and we collected data regarding demographics, epidemiology, clinical and laboratory aspects in these patients, at baseline and in dynamics.

**Results**

We included in our study 403 adults newly diagnosed with HIV infection in the National Institute for Infectious Diseases “Prof. Dr. Matei Balș” between the 1^st^ of January 2015 and 30^th^ of June 2016. 295 (73.21 %) of those diagnosed with HIV were men and 108 (26.79 %) were women. A syphilis diagnostic test was performed in 301 (74.68 %) of these patients and 64 (16 %) had HIV-syphilis coinfection. A concomitant HIV-syphilis diagnosis was established for 57 (90 %) out of 64 patients. Out of 9 % of patients in whom a treponemal test was performed from the CSF, 6 % had a negative result and 3 % (2 patients) were diagnosed with neurosyphilis. Most of our patients with HIV syphilis coinfection were of reproductive age: most patients were aged between 30 and 39 years old (21 men and 7 women) while the second age group was that of 20–29 years old (13 men and 6 women). 20 (31.25 %) of the patients diagnosed with HIV and syphilis were diagnosed also with at least one type of hepatitis virus. Most of them, 18 (28.12 %) were infected with hepatitis C virus and 2 (3 %) had hepatitis B virus. Out of 64 patients with coinfection, 23 had AIDS at the time of diagnosis. The medium viral load at baseline was 594.251 copies/cmm and 18 patients had a viral load above 100000 copies/cmm. 192 (47.62 %) patients diagnosed with HIV received antiretroviral therapy.

**Conclusions**

Testing for other sexually transmitted diseases (especially syphilis) remains an important objective in HIV infected patients in order to prevent transmission and improve the outcome.

### A43 Thrombophilia – additional risk factor for the evolution of pregnancy in HIV-positive patients

#### Mihai Mitran^1,2^, Carmen Georgescu^1^, Loredana Mitran^3^, Simona Vladareanu^2,3^

##### ^1^Clinical Hospital of Obstetrics and Gynecology “Prof. Dr. Panait Sârbu”, Bucharest, Romania; ^2^Carol Davila University of Medicine and Pharmacy, Bucharest, Romania; ^3^Elias University Emergency Hospital, Bucharest, Romania

###### **Correspondence:** Mihai Mitran (michael_digital@yahoo.com)

**Background**

Thrombophilias are a group of hereditary or acquired hematological conditions which predispose to thrombotic phenomena and are triggered by molecular dysfunctions of the hemostasis proteins. The thrombophilia comorbidity in HIV-positive pregnant women has been little studied.

**Methods**

The study carried out in 2015 at the Clinical Hospital of Obstetrics and Gynecology „Prof. Dr. Panait Sârbu” from Bucharest refers to a number of 9 HIV positive patients diagnosed with thrombophilia.

**Results**

The study was carried out on a number of 51 HIV positive patients, out of which 45 pregnant women eventually gave birth and 6 had second trimester spontaneous abortion (patients with deliberate pregnancy). Out of the patients who delivered (42 by Caesarian section and 3 by vaginal birth) 6 cases were diagnosed with thrombophilia (2 cases with antiphospholipid syndrome and 4 cases with congenital thrombophilia). The thrombophilia diagnosis was established in 5 of the cases with spontaneous abortion with repetition in the second pregnancy trimester. In all cases the treatment with low molecular weight heparine was initiated.

**Conclusions**

No correlation between the incidence of thrombophilia and HIV infection stage or the duration of ARV therapy has been found.

### A44 The incidence of oropharyngeal candidiasis in hospitalized HIV infected pediatric Romanian cohort between 1 January - 31 December 2015

#### Andreea Ioana Magirescu^1^, Viorica Andreev^1^, Cristina Nicolau^1^, Alexandra Largu^1^, Carmen Dorobat^1,2^, Carmen Manciuc^1,2^

##### ^1^Infectious Diseases Hospital “Sf Parascheva”, Iași, Romania; ^2^“Gr.T.Popa” University of Medicine and Pharmacy, Iași, Romania

###### **Correspondence:** Andreea Ioana Magirescu (m.andreea_ioana_2009@yahoo.com)

**Background**

Relapsed or difficult to treat oropharyngeal candidiasis is in some cases revealing for HIV infection, being the most common fungal infection in these patients. The objective of this study is to determine the prevalence of oropharyngeal candidiasis in HIV infected patients.

**Methods**

We presented a retrospective study on patients born between 1988–1989, in which we included 216 patients diagnosed with AIDS, staged as A3, B3 and C AIDS, monitored in the Infectious Diseases Hospital of Iași and admitted between 1^st^ of January and 31^st^ of December 2015.

**Results**

In the 216 group of patients evaluated in the study, the percentage of female patients was 52.31 %, while in men it was 47.68 %. The prevalence of urban area was (64.56 %), versus only (35.44 %) for rural environment. AIDS C3 stage of disease represented 56.94 %, followed by B3 stage (27.7 %), C2 (8.33 %), and C1 stage (3.25 %). The therapy was established with local antifungal treatment in 32 % of the cases, 68 % receiving systemic treatment with Diflucan, instituted during hospitalization.

**Conclusions**

The degree of immunosuppression correlates with oropharyngeal manifestations of infection, oropharyngeal candidiasis having a very high prevalence in C3 staged patients. Early detection and treatment of these lesions is provided if there is a permanent collaboration between the infectious disease specialist and the dentist.

### A45 TB incidence in HIV infected patients during the year of 2015

#### Viorica Andreev^2^, Andreea Ioana Magirescu^2^, Ina Isac^2^, Cristina Nicolau^2^, Alexandra Largu^2^, Carmen Dorobat^1,2^, Carmen Manciuc^1,2^

##### ^1^“Gr.T.Popa” University of Medicine and Pharmacy, Iași, Romania; ^2^Infectious Diseases Hospital “Sf Parascheva”, Iași, Romania

###### **Correspondence:** Viorica Andreev (andreevviorica@yahoo.com)

**Background**

Tuberculosis is the most common opportunistic infection and a major cause of death in HIV infected patients. The annual risk for active tuberculosis in these patients is 5–10 % per year, compared to 5–10 % for lifetime in uninfected individuals. Objectives: To determine the prevalence of TB infection in HIV infected patients.

**Methods**

We studied retrospectively the observation charts of patients with generalized tuberculosis, lung, lymph node and HIV infected hospitalized in the Regional HIV/AIDS Center Iași, between the 1^st^ of January and 31^st^ of December 2015.

**Results**

During the year of 2015, in Iași Infectious Diseases Hospital 15 cases of TB were registered among the total of 1060 HIV infected patients, that means a prevalence of 1.4 %. Of all patients, a number of 3 (20 %) were non-adherent, non-compliant to ARV therapy; 2 cases of TB (13.3 %) belonged to pediatric cohort with known and treated HIV infection, since childhood. In 4 cases (26.6 %) TB infection was revelatory for AIDS, and we registered 2 deaths (13.3 % of all patients hospitalized for TB). Note that in both cases the patients were ARV non-adherent, and non-compliant. At the moment of detection of TB infection, CD4 levels varied between 80 and 436 cells/cmm, and the values of viremia oscillated between 20 and 364000 copies/mL. The average duration of hospitalization was 9 days, requiring therapeutic approach in infectious diseases specialists team-intensive care specialist-psychologist.

**Conclusions**

Although there is a low prevalence of *Mycobacterium tuberculosis* infection, this condition is a challenge for infectious diseases specialists, psychologists and ATI specialists, regarding the therapeutic adherence and compliance and long-term care and follow-up of HIV positive patients that have also tuberculosis.

### A46 Retrospective analysis of HIV/AIDS deaths recorded in the Clinical Infectious Diseases Hospital, Constanța in the period 01 January 2014–30 June 2016. Epidemiological considerations

#### Iulia Gabriela Șerban, Ghiulendan Resul, Consuela Marcaș

##### Clinical Infectious Diseases Hospital of Constanța, Constanța, Romania

###### **Correspondence:** Iulia Gabriela Șerban (serbaniuliagabriela@gmail.com)

**Background**

By recording data obtained from analysis of HIV/AIDS deaths which were necropsied the last 2½ years in our hospital we propose updating the HIV/AIDS database belonging to the Clinical Hospital of Infectious Diseases, Constanța, focusing on parameters that express the epidemiological tendency of the evolution of HIV/AIDS in the region. Objective: Analysis of the data referring to the HIV/AIDS deaths recorded in the Clinical Infectious Diseases Hospital, Constanța, in the period 01.01.2014 - 30.06.2016. Formulation of general epidemiological considerations.

**Methods**

We conducted a retrospective epidemiological study on data from the Register of deaths and from the Register of pathological examinations (necropsy) of the Clinical Infectious Diseases Hospital, Constanta, in the period 01.01.2014 - 30.06.2016.

**Results**

From a total of 47 deaths (19 F, 28 M) appearing in the records of the Pathology Department, there are 40 deaths (100 %) HIV AIDS (18 F - 45 %, 22 M - 55 %), of which were autopsied 26 - 65 % (11 F - 27.5 %, 15 M - 37.5 %). Distribution of HIV/AIDS deaths according to resident backgrounds - 21 urban (52.5 %), rural 19 (47.5 %). The residence towns mentioned are: Constanța (12 - 30 %), Năvodari (4 - 10 %), Medgidia (2 - 5 %), Cernavodă (2 - 5 %), Mangalia (1–2.5 %). From rural provenance there are the residence localities: Râmnicu de Sus, Târguşor, Poarta Albă, Văleni, Făurei, Cobadin, Drăgăşani, Pecineaga (2014), Lumina, Dobromir, Oituz, Valu Traian (2015), Mihail Kogălniceanu, Ciocârlia, Canlia, Lipniţa, Ostrov, Techirghiol (2016). Of the 40 HIV/AIDS patients who died, 25 (62.5 %) have birth years from 1984 to 1990, of which 21–52.5 % (9 F, 12 M - male predominance) belong to cohort 1987–1989 (1987–6 deaths - 15 %, 1988–9 deaths - 22.5 %, 1989–6 deaths - 15 %). The number of registered HIV/AIDS deaths fell from year to year: 2014–19 deaths (47.5 %), 2015–11 deaths (27.5 %), 2016 (first 6 months) - 10 (25 %). During designated period, the age group 25–29 years had the highest mortality (25 deaths - 62.5 %), followed by the age group 45–49 years (5 deaths - 12.5 %). The common pathological diagnoses are type: liver damage (liver B cirrhosis, chronic hepatitis C), lymphoma (Burkitt) - on growing trend, lung disease (pulmonary TB on decreasing trend, pneumocystosis), sepsis.

**Conclusions**

HIV AIDS patients continue to experience difficulties in specific assessment and monitoring. The role of the epidemiology specialist (the clinician of the community), of the specialist in palliative cares (before death, the HIV/AIDS patients had required palliative care), pathologist (diagnostic certainty) were revised.

### A47 Acute liver failure with favorable evolution in an HIV-HBV coinfected patient

#### Iosif Marincu^1^, Patricia Poptelecan^2^, Bogdan Trincă^2^, Sorina Mitrescu^2^, Anca Tudor^1^, Daliborca Vlad^1^, Livius Tirnea^1^

##### ^1^Dr. Victor Babeş University of Medicine and Pharmacy, Timişoara, Romania; ^2^Dr. Victor Babeş Clinical Hospital of Infectious Diseases and Pneumology, Timişoara, Romania

###### **Correspondence:** Iosif Marincu (imarincu@umft.ro)

**Background**

Patients with HIV/AIDS represent a risk group for viral hepatitis which may progress to severe liver failure or other complications. We present the case of a patient with acute liver failure with chronic B hepatitis known with HIV infection late presenter.

**Case report**

The patient, diagnosed with chronic B hepatitis and HIV infection in August 2015 is admitted in the Clinic of Infectious Diseases Timișoara for: scleral and skin jaundice, hyperchrome urine, hepatalgia, nausea, anorexia, asthenia, etc. The physical examination at admission revealed: influenced general state, afebrile, scleral and skin jaundice, generalized lymphadenopathy, the inferior edge of the liver palpable at 2.5-3-3.5 cm below the costal margin, the lower pole of the spleen was palpable, with no signs of meningeal irritation. Biological samples were collected: cell blood count, biochemistry, coagulation, electrophoresis, CD4, HIV viral load (VL), HBV VL, AcVHD, AcHCV, bilirubin, γ-GT, alkaline phosphatase, α-fetoprotein, cholinesterase, urinalysis, etc. The patient is unemployed, with a low intellectual level (4 elementary grades) and low adherence to ARV therapy (Combivir 2x1 tablet/day, 400 mg Prezista 2x1 tablet/day, 100 mg Norvir 1 tablet/day). The results of blood tests were: RBC 3.27x10^6^/μL, Hb 12.4 g/dL, Ht 33.1 %, PLT 118x10^3^/μL, prothrombin time 19.4 sec (42.6 %), total bilirubin 29.18 mg/dL, direct bilirubin 23.98 mg/dL, TGP 429.9 U/L, TGO 293.8 U/L, total protein 5.44 g/dL, cholinesterase 2147 U/L, γ -GT 63.4 U/L, alkaline phosphatase 141 U/L, α-FP 257.3 IU/mL, Electrophoresis: 44.7 % albumin, α-1 globulin 3 %, α-2 globulin 7.4 %, β-globulins 8.3 %, γ-globulins 36.6 %, CD4 83 cells/μL, HIV VL <20 copies/mL, HBV DNA 461634 IU/mL, negative HDV Ab, negative HCV Ab. The treatment was complex: Ampicillin, Cefort, Arginine-Sorbitol, Aspatofort, Hepa-Merz, Ursofalk, Bilichol, phenobarbital, hydrocortisone hemisuccinate, Omeprazole, KCl, Vitamin B6, calcium gluconate, plasma, glucose 10 %, sodium chloride 0.9 %, etc. After 20 days of hospitalization, the clinical and biological evolution was favorable (TB 16.04 mg/dL, TGP 120 U/L, PT 14.5 sec (55.5 %), total protein 6.6 g/dL, K 4.1 mmol/L), the patient is cooperative, he is fed orally, without nausea or vomiting, without bleeding, and the jaundice is in remission. We note that we made a switch of the antiretroviral therapy using emtricitabine (200 mg/day), Tenofovir (245 mg/day) Raltegravir (400 mg, 2x1 tablets/day).

**Conclusions**

An early diagnosis, associated with specific therapy and rigorous monitoring of clinical and biological data, can contribute to a favorable evolution in patients with HIV-HBV coinfection and acute liver failure.

**Consent**

Written informed consent was obtained from the patient for publication of this Case report and any accompanying images. A copy of the written consent is available for review by the Editor of this journal.

### A48 Lifestyle impact on HIV management

#### Nurcan Baydaroglu^1^, Alina Cristina Neguț^1,2^, Oana Săndulescu^1,2^, Daniela Manolache^2^, Gabriela Ceapraga^2^, Monica Andreea Stoica^2^, Anca Streinu-Cercel^1,2^, Adrian Streinu-Cercel^1,2^

##### ^1^Carol Davila University of Medicine and Pharmacy, Bucharest, Romania; ^2^National Institute for Infectious Diseases “Prof. Dr. Matei Balș”, Bucharest, Romania

###### **Correspondence:** Nurcan Baydaroglu (nurcanbb@hotmail.de)

**Background**

Introduction of highly active antiretroviral therapy moved the step forward in HIV infection, reducing the mortality caused by opportunistic infections and making the disease a manageable chronic condition.

**Methods**

We have performed a cross-sectional study on 119 consenting HIV-infected patients from the National Institute for Infectious Diseases “Prof. Dr. Matei Balș”, Bucharest, in the time span 2014–2015, to assess their medical history, lipid profile, CD4 cell count, viral load, and lifestyle habits.

**Results**

In our study group, the male:female ratio was almost 2:1. The median (interquartile range) age was 35 (26, 44) years and the mean ± standard deviation body mass index (BMI) was 23.65 ± 3.97 kg/sqm. A total of 42 (37 %) patients were overweight/obese. Almost all our patients were on antiretroviral therapy (115/119) and 69 (73 %) patients had comorbidities. The median recent CD4 cell count was 518.00 (305.50, 745.50) cells/mL. Regarding risk factors: most of our patients (70 %) were not consuming alcohol; almost half of our patients (45 %) were smokers. Most of the patients were performing moderate (39 %) or intense (36 %) physical activity. The BMI was influenced by lipid profile, a higher proportion of overweight/obese patients were identified in the group of patients with high triglycerides levels or high LDL level compared with the group with normal values (p = 0.0002, p = 0.013). We also observed that triglycerides and cholesterol were not influenced by alcohol consumption (p = 0.617, p = 0.802) or by smoking (p = 0.317, p = 0.275). Heavy drinking may have a negative impact on glycemia levels, becoming a risk factor for diabetes – in our study there was a higher percentage of alcohol users in the group of patients with high glycemia (p = 0.041). Smoking was not associated with hydroxy-vitamin D concentration (p = 0.568) or parathyroid hormone value (p = 0.944), but smoking was identified as a risk factor for femoral osteopenia (p = 0.013). Physical activity was not associated with HIV status; the proportion of patients performing intense physical activity was similar in the group of patients with recent CD4 cell count less than 200 cells/cmm compared with the group of patients with recent CD4 cell count higher than 500 cells/cmm (p = 0.802).

**Conclusions**

Considering the duration of HIV infection a risk factor for complications due to chronic inflammation, the modifiable risk factor (smoking, alcohol consumption, sedentariness) should be avoided for a better quality of life.

**Acknowledgement**

1. License thesis “Lifestyle impact on HIV management” performed at Carol Davila University of Medicine and Pharmacy, Bucharest. Supervisor: Anca Streinu-Cercel, Coordinator: Alina Neguț.

2. Cardio-metabolic project – Merck Sharp&Dohme.

### A49 HIV positive mothers newborns - clinical experience from January 2012 to June 2016

#### Carmen Manciuc^1,2^, Mariana Pagute^3^, Cristina Nicolau^1^, Carmen Dorobăț^1^, Alexandra Largu^1^

##### ^1^Infectious Diseases Hospital “Sf Parascheva”, Iași, Romania; ^2^“Gr.T.Popa” University of Medicine and Pharmacy, Iași, Romania; ^3^Emergency Hospital for Children “St. Maria” Iaşi, Romania

###### **Correspondence:** Mariana Pagute (pagute.mariana@gmail.com)

**Background**

Women at childbearing age with HIV infection want to procreate, their management represents a challenge for the infectious disease doctor.

**Methods**

We studied retrospectively the observation charts of HIV positive patients, who were under observation in the Regional HIV/AIDS Center Iași, in the period January 2012-June 2016.

**Results**

Iași Regional Center follows 1475 patients that are HIV infected, of which 676 women, that represents a percentage of 45.83 % of the total. A number of 623 represents women at childbearing age (13–49 years), which represents 92.16 % of all HIV positive women. In the mentioned period, 133 women gave birth, representing 21.35 % of all women with HIV infection at childbearing age. Counties distribution showed a higher incidence in Bacău, namely 21.81 % (39 newborn) and the year with most births occurred in 2013 with 33 newborn (24.81 %). 79.7 % of patients received treatment with KLT + CVB, 10 women were detected with HIV infection at birth. 94 % of children were born by Caesarian section. During these years, there have been two deaths in children, one of whom died 10 days after birth and another was stillborn. All newborns received prophylaxis for 6 weeks, in one case prophylaxis was interrupted for a month. Three of the children were declared HIV positive at 18 months.

**Conclusions**

The correct tracking and teamwork (infection disease doctor, social worker, psychologist) made the number of HIV positive patients who gave birth to HIV negative children to be significantly high, due to a good adherence and compliance to ARV therapy.

### A50 Rediscovering HIV-associated progressive multifocal leukoencephalopathy and HIV encephalopathy: clinical suspicion and subsequent brain autopsies

#### Ioan-Alexandru Diaconu^1^, Laurențiu Stratan^1^, Daniela Ion^2^, Luciana Nichita^2,3^, Cristina Popescu^1,2^, Raluca Năstase^1^, Daniela Munteanu^1^, Violeta Molagic^1^, Cătălin Tilișcan^1,2^, Mihaela Rădulescu^1,2^, Alexandra Diaconu^3^, Anca Negru^1,2^, Alina Orfanu^1,2^, Cristina Dragomirescu^1^, Remulus Catană^1,2^, Anca Leuștean^1^, Irina Duport-Dodot^1^, Cristina Murariu^1^, Iulia Bodoșca^1^, Violeta Niță^1^, Alexandra Badea^1^, Victoria Aramă^1,2^

##### ^1^National Institute for Infectious Diseases “Prof. Dr. Matei Balș”, Bucharest, Romania; ^2^Carol Davila University of Medicine and Pharmacy, Bucharest, Romania; ^3^“Prof. Marius Nasta” National Institute for Pneumology, Bucharest, Romania

###### **Correspondence:** Ioan-Alexandru Diaconu (diaconuia@yahoo.com)

**Background**

Two of the foremost neurological impairments developed by HIV late presenters consist of progressive multifocal leukoencephalopathy (PML) and HIV encephalopathy (HIVE). PML is a viral opportunistic pathology, associated almost exclusively with the HIV infection. HIVE is a not-opportunistic disease, directly mediated by HIV.

Diagnosing PML and HIVE represents a challenge for physicians, taking into consideration the myriad of variables, the associated conditions that could be brought about by the HIV infection and the advent of new tools that could be successfully used in order to provide a diagnosis.

**Methods**

Observational and cross-sectional study consisting of 15 HIV-infected patients treated at the National Institute for Infectious Diseases “Prof. Matei Balș”, with exitus due HIV-associated complications, which were subject to brain necropsies. Brain tissue samples were analyzed by pathologists from the Colentina Clinical Hospital in Bucharest.

We have compared the pathological diagnoses with their corresponding clinical ones, enforced by clinical presentation, laboratory and medical imaging data, collected from archived medical records. The end-point was identifying the superposition of clinical and pathological diagnoses.

**Results**

Four PML pathologic diagnoses were issued, three of them as confirmation to their respective three clinical suppositions and one without a corresponding clinical diagnosis. PML was also suspected in a patient that lacked relevant pathologic evidence for its confirmation. However, the patient proved to be suffering from cerebral cryptococcosis. In the case of two patients, PML was the sole pathologic diagnosis, whereas the rest of them exhibited PML accompanied by HIV encephalopathy. There were also three clinical diagnoses that were speculated, alongside PML, all of them being refuted by the post-mortem examination: cerebral hemorrhage, cerebral toxoplasmosis and lymphoma.

Four HIVE pathologic diagnoses were issued, with no correlation with the clinical suppositions inferred in regard to the concerned patients. These pathological diagnoses were associated with ischemic stroke, PML, cerebral hemorrhage, lymphoma or toxoplasmosis. HIVE was clinically suspected in six patients, but all of them were proved to be affected by other disorders, such as meningoencephalithis, hyperemia and cerebral edema, PML or pons/cerebellar decrepitude. In all, there were no confirmed cases of HIV encephalopathy.

**Conclusions**

The superposition of PML clinical and pathological diagnoses was 3/5 (60 %) and there were no pathologically confirmed cases of HIVE, with elevated levels of over- and under- diagnosis. Thus, the observed lack of correlation calls into better understanding of both illnesses. In the extended presentation we provided an up to date diagnosis algorithm for both.

### A51 Antenatal surveillance of pregnant women with risk behavior and its impact on mother-to-child HIV transmission in Romania

#### Mariana Mărdărescu, Cristina Petre, Marieta Iancu, Rodica Ungurianu, Alina Cibea, Ruxandra Drăghicenoiu, Ana Maria Tudor, Delia Vlad, Sorin Petrea, Carina Matei, Dan Oțelea, Carmen Crăciun, Cristian Anghelina, Alexandra Mărdărescu

##### National Institute for Infectious Diseases “Prof. Dr. Matei Balș”, Bucharest, Romania

###### **Correspondence:** Mariana Mărdărescu (mardarescum@yahoo.com)

**Background**

Released in 2014, UNAIDS’s Fast Track-Ending the AIDS Epidemic by 2030 sets certain targets, which in Romania can be achieved through close monitoring of perinatal HIV. Hence, the national antenatal HIV screening process has revealed an increase in the number of HIV infected women – intravenous drug users (IDUs) since 2011, due to consumption of synthetic cannabinoids and cathinones (the so called “legal highs”). Moreover, the percentage of new IDU/HIV cases increased from 2.8 % in 2010 to 20 % in 2015, the highest number of consumers living in or around Bucharest.

**Methods**

Between January 2012 and June 2016, the Pediatric Immunosuppression Unit of the National Institute for Infectious Diseases “Prof. Dr. Matei Balș” surveyed 421 HIV-exposed newborns. 14.7 % (62/421) were perinatally exposed to “new drugs” consumed by their mothers. However, at the end of 2015 the rate of HIV mother-to-child transmission in Romania remained stable-below 2.3 %. The following parameters were evaluated for mothers: time of HIV screening, time of HIV diagnosis, age, prenatal care, time when taken into the active surveillance program, treatment/prophylaxis, type of birth, drugs taken and screening for co-infections (HBV, HCV, STIs). The parameters of interest for children were: sex, age, time of diagnosis, ART prophylaxis, type of birth, type of feeding, neurological assessment, CD4 count, HIV-RNA, ultrasound evaluation.

**Results**

During the 4 year monitoring period, the following results of HIV time of detection in IDU/HIV mothers were obtained: 40 % (25/62 mothers tested) were found with HIV before pregnancy, 11.2 % (7/62) during pregnancy, 41 % (26/62) during delivery and 6.4 % (4/62) in the first 24 hours after birth. Although all newborns perinatally exposed to HIV/IDU benefitted from post-partum prophylaxis and received artificial nutrition, 14.5 % (9/62) exposed to drug use were HIV infected. All children had poor neonatal adaption, mainly a severe withdrawal syndrome - 57 % (40/62) of newborns monitored and more than 50 % had neurological lesions.

**Conclusions**

The majority of pregnant women with HIV-consumers of drugs (especially “new drugs”) have little access to antenatal care, which leads to late discovery of HIV. The use of synthetic cannabinoids and cathinones by HIV pregnant women has important effects on newborns and their subsequent development which poses a challenge for the post-partum surveillance system. These women come from poor families who don’t ensure them a proper support. Furthermore, due to issues associated to risky behavior it is difficult to include them in HIV screening or prevention programmes, which need to be adapted to their needs.

### A52 Noninvasive assessments (APRI, Fib-4, transient elastography) of fibrosis in patients with HIV and HIV/HBV infection

#### Elena Dumea^1,2^, Adrian Streinu-Cercel^3^, Sorin Rugină^1,2^, Lucian Cristian Petcu^4^, Stela Halichidis^1,2^, Simona Claudia Cambrea^1,2^

##### ^1^Faculty of Medicine, “Ovidius” University, Constanța, Romania; ^2^Clinical Infectious Diseases Hospital of Constanța, Constanța, Romania; ^3^Carol Davila University of Medicine and Pharmacy, Bucharest, Romania; ^4^Faculty of Dental Medicine, Ovidius University, Constanța, Romania

###### **Correspondence:** Elena Dumea (elenadumea@yahoo.com)

**Background**

In our country, the prevalence of HIV with hepatitis B virus (HBV) co-infection in young HIV positive patients was high, about 40 %. Measurement of liver fibrosis using (as a noninvasive method) transient elastography (TE) in HIV patients can be used as a tool to determine and monitor hepatic diseases. We evaluated the ability of APRI and FIB-4 score to differentiate between the different stages of fibrosis (no fibrosis/minimal fibrosis = F0-1 and F2-4 = moderate-severe fibrosis/cirrhosis), taking as a reference, in the absence of liver biopsy, hepatic fibrosis stratification by Fibroscan.

**Methods**

We studied a group of patients infected with HIV (110) and 64.5 % of these are co-infected HIV/HBV. We determined the cut-off values for APRI and FIB-4 that identify significant fibrosis with maximum specificity by AUROC for each group. Kappa score was then calculated for the concordance between methods.

**Results**

For HIV/HBV co-infected patients, to identify significant fibrosis comparing APRI versus Fibroscan the Kappa score = 0.494, 95 % CI (0.245, 0.742) on the identification of fibrosis (F0-1 to F ≥ 2); for FIB-4 versus Fibroscan the Kappa score = 0.481, 95 % CI (0.238, 0.725) for both the moderate concordance. Regarding the comparison of the two methods APRI and FIB-4, Kappa score = 0.698, 95 % CI (0.485, 0.910), concordance significant. For patients with HIV, to identify significant fibrosis by APRI versus Fibroscan Kappa = 0.217, 95 % CI (−0.424, 0.858) on the identification of fibrosis (F0-1 to F ≥ 2), for the FIB-4 Kappa = 0.164, 95 % CI (−0.451, 0.779) for both the correlation is reduced. Regarding the comparison of the two methods APRI and FIB-4 Kappa = 0.217, 95 % CI (−0.424, 0.858), which confirms the low correlation.

**Conclusions**

There is some evidence that the tests used: APRI and FIB-4 have the ability to distinguish for both groups of patients (HIV and HIV/HBV) between the two classes of fibrosis (F0-1 to F ≥ 2), patients with and without advanced liver disease. Although in patients with HIV infection a low concordance was seen between non-invasive methods for the diagnosis of fibrosis, in co-infected patients it was moderate and these tests could be used as evaluation methods in their monitoring of liver injury especially when the results of these tests are concordant.

### A53 Undetectable HIV viral load – the main goal in the management of HIV-infected patients

#### Carmen Chiriac, Nina-Ioana Bodnar, Iringo-Erzsebet Zaharia-Kezdi, Cristina Gîrbovan, Andrea Incze, Anca Meda Georgescu

##### University of Medicine and Pharmacy Tîrgu Mureș, Tîrgu Mureș, Romania

###### **Correspondence:** Nina-Ioana Bodnar (ninasincu@yahoo.com)

**Background**

Highly active antiretroviral therapy (HAART) represents the corner-stone in the management of HIV-infected patients, its main goal – immune restoration, rapidly achieved and maintained undetectable HIV-RNA viral load.

**Methods**

We performed a retrospective descriptive study including the HIV-infected patients in care in Mureș county. We selected all patients who achieved rapid and long-lasting undetectable HIV-RNA plasma viral load (36 months). We collected data regarding demographic features, route of transmission and duration of HIV infection, nadir CD4+ T-cells count, maximum HIV-RNA plasma viral load.

**Results**

Out of all 200 HIV-infected patients from Mureș county in care in the Clinic of Infectious Diseases I, County Clinical Hospital Mureș, under HAART at present time, 78 (39 %) have had undetectable viral loads for the past 36 months. 57.69 % are male patients, 42.31 % female. Most of them (72 %) are part of the Romanian cohort, while 28 % have acquired the infection via sexual route. The average length of HIV-infection history was 12 years, with a maximum of 26 years. Nadir CD4+ T-lymphocytes count registered an average of 248 cells/μL, while the highest HIV-RNA plasma viral load had an average level of 295094 copies/mL. The most recent CD4+ T-cells count average is 661 cells/μL. All patients had good adherence to HAART regimen.

**Conclusions**

Combined antiretroviral therapy leads to good results, with immunologic and virologic success and provided a good compliance and correct administration.

### A54 LPS serum levels and correlation with immunological, virological and clinical outcome in HIV infected patients

#### Simona Alexandra Iacob^1^, Diana Gabriela Iacob^1^, Eugenia Panaitescu^2^, Monica Luminos^1^, Manole Cojocaru^3^

##### ^1^National Institute for Infectious Diseases “Prof. Dr. Matei Balș”, Bucharest, Romania; ^2^Carol Davila University of Medicine and Pharmacy, Bucharest, Romania; ^3^Titu Maiorescu University, Bucharest, Romania

###### **Correspondence:** Simona Alexandra Iacob (simonaaiacob@yahoo.com)

**Background**

Lipopolysaccharide (LPS), a heteropolymer synthesized by Gram negative bacilli is considered a marker of microbial translocation and immune activation in HIV infected patients. Nevertheless, low LPS serum levels were not always correlated with a favorable clinical outcome. Our study explores the serum LPS level correlation with virological and immunological markers, treatment adherence and clinical evolution in HIV-infected patients.

**Methods**

We performed a prospective study between 2011–2016 on 44 patients admitted to “Matei Balș” National Institute for Infectious Diseases, of which 34 HIV infected patients (14 women, 20 males aged between 16–64 years) and 10 controls. Thirty-two of the HIV-infected patients had been following antiretroviral treatment for at least 6 months and 23 were considered adherent to treatment; two patients refused the treatment. The serum LPS level (Endoblock ELISA test kit), CD4 T lymphocyte count (flowcytometry) and the patients’ HIV RNA (Real Time PCR) viral load were assessed. The patients were classified depending on their HIV RNA viral load (responder/nondetectable RNA HIV versus nonresponder/detectable RNA HIV), the CD4 T cell count (CD4-T cell below or above 200 cell/cmm), clinical outcome (favorable or unfavorable due to opportunistic infections and malignancies over the next 2 years) and length of previous antiretroviral treatment (<5 years or > 5 years). We compare the serum level of LPS between the different groups of HIV infected patients and between the HIV-infected patients and healthy controls. Statistical analysis was performed using Student’s T test, Mann–Whitney analysis and Pearson’s correlation.

**Results**

The level of LPS was not correlated with the CD4 T lymphocyte count (p = 0.814), virologic response (p = 0.744), clinical outcome (p = 0.210) or therapeutic adherence (p = 0.692). Nevertheless the serum level of LPS was significantly higher in HIV-infected patients (71.34 ± 67.48 pg/mL compared with 13.52 ± 16.87 pg/mL, p value <0.001).

**Conclusions**

HIV infected patients display significantly higher values of LPS irrespective of the length of treatment or clinical outcome. Although LPS levels were not correlated with immunologic or virologic parameters it is possible that dynamic measurements could help ascertain the role of LPS in the clinical evolution of these patients.

### A55 LL37 human cathelicidin serum levels are positively correlated with IFN gamma and alanine aminotransferase level in HCV infection

#### Simona Alexandra Iacob, Diana Gabriela Iacob, Monica Luminos

##### National Institute for Infectious Diseases “Prof. Dr. Matei Balș”, Bucharest, Romania

###### **Correspondence:** Simona Alexandra Iacob (simonaaiacob@yahoo.com)

**Background**

Cathelicidin LL37 is a human immunomodulatory peptide synthesized in monocytes/macrophages through T-cell secreted IFN ɣ induction, following the activation of vitamin D receptors. LL37 was also detected in the biliary and liver endothelium and recent studies indicate a direct antiviral effect against HCV in cell cultures but its role in liver inflammation remains unknown.

**Methods**

We performed a prospective study on 27 patients (17 women, 10 men, aged 22–71 years) with HCV infection and 6 controls hospitalized in the National Institute for Infectious Diseases “Prof. Dr. Matei Balș” Bucharest, Romania. Of the 27 patients, 15 had recently received treatment with peginterferon and ribarivin and showed negative RNA HCV viral loads, 2 patients were cured following acute HCV infection and 10 were untreated (positive RNA HCV viral loads). The study recorded the serum level of LL37 (Elisa Hycult Biotech) and IFN ɣ (Elisa, Bioo Scientific) along with liver injury markers: alanine aminotransferase (ALT), aspartate aminotransferase (AST), alkaline phosphatase (ALP), gamma-glutamyl transferase (GGT), bilirubin (Bil) and CD4 and CD8 T cell lymphocyte count (flowcytometry).

**Results**

The mean level of serum LL37 in all HCV patients was lower than controls 48.75 (±19.02) ng/mL versus 80.98 (±82.78) ng/mL but the difference was not statistically significant (p = 0.435). The mean value of IFNɣ was also only numerically higher in HCV infected patients 604.94 (±1532.17) pg/mL than controls 235 (±131.77) pg/mL, p = 0.6. The level of LL37 exhibited a moderate positive correlation with IFN ɣ (r = 0.571) as well as a mild positive correlation between LL37 and ALT (r = 0.404). No statistically significant correlations of LL37 serum concentrations were found when compared with other biological parameters and virologic response.

**Conclusions**

The positive correlation between the serum level of LL37 and IFN ɣ or LL37 and ALT suggests a possible implication of this peptide in liver injury in HCV patients. Further studies are needed to translate the results from in vitro studies on LL37 into the clinical practice.

### A56 Early diagnosis of pulmonary tuberculosis in a non-compliant HIV/AIDS late presenter patient

#### Vochita Laurențiu, Vochita Andreia, Opreanu Radu, Trinca Bogdan, Rosca Ovidiu, Marincu Iosif

##### Dr. Victor Babeş Clinical Hospital of Infectious Diseases and Pneumology, Timişoara, Romania

###### **Correspondence:** Vochita Laurențiu (vochita_Laurentiu@yahoo.com)

**Background**

Pulmonary tuberculosis is one of the most important opportunistic infections in patients with HIV/AIDS, which frequently associates with difficulties regarding the clinical and therapeutic supervision of patients. The purpose of this paper is to present a clinical case of a patient with C3 stage HIV/AIDS infection, non-complaint with antiretroviral therapy, precociously diagnosed with pulmonary tuberculosis, pneumonia with *Pseudomonas* spp., candidiasis of the digestive system and toxoplasmosis.

**Case report**

The authors present the case of a 29 year old patient diagnosed with HIV infection 5 years ago, for which he refused antiretroviral medication. The patient is admitted into our clinic presenting fever, chills, dysphagia, dysphonia, cough with mucopurulent sputum, vomiting, epigastralgia, cephalalgia, cachexia, the onset and duration of the symptomatology being of approximately two weeks. The physical examination upon admission revealed paleness of the skin and mucous membranes, dry lips, white coating of the tongue, stomatitis, asthenic thorax, bilaterally harsh vesicular murmur, BP 125/79 mmHg, pulse 106, oxygen saturation 99 % on room air, awake, alert and fully oriented, no clinical signs of meningeal irritation. Biological samples were collected for the confirmation of the diagnosis (RBC and WBC count, GOT, GPT, alkaline phosphatase, serum urea and creatinine, C-reactive protein, blood cultures, CD4, viral load, *Toxoplasma* IgM antibodies, GeneExpert specimen BK, coproculture, sputum culture, etc). A pulmonary X-ray and a CT scan of the thorax were also performed. Results of the lab tests included: WBC 3380/μL, RBC 3500000/μL, Hemoglobin 10.4 g/dL, HCT 29.6 %, ESR 90 mm/1 h, GOT 60.8 U/L, GPT 67.8 U/L, GGT 153.4 U/L, ALP 112.7 U/L, CRP 205.33 mg/L, CD4 25 cells/μL, VL 871475 copies/mL, positive IgM Ab for *Toxoplasma*, sputum culture *Pseudomonas* spp. 80 %, *Candida* yeasts 20 %, coproculture *Candida* 30 CFUs/mL, urine culture candida 40.000 CFUs/mL, GeneExpert positive BK, negative BK culture, thorax CT scan condensation process in the right inferior lobe. The following therapeutic regimen was established: Sumetrolim 2x2 tablets/day, Levofloxacin 500 mg/day and Gentamicin 3x80 mg/day according to the antibiogram, Fluconazole 400 mg/day, Dexamethasone 3x8 mg/day, tuberculostatics (Isoniazid 300 mg/day, Rifampicin 600 mg/day, Pyrazinamide 1.5 g/day, Ethambutol 1.6 g/day), ARVs (Combivir, Invirase, Norvir), antipyretics, mucolytics, hydroelectrolytic rebalancing fluids, with slow favorable evolution. After a period of one year, the patient discontinues the ARV medication and dies.

**Conclusions**

Modern molecular biology techniques (GeneExpert) offer the possibility of early pulmonary tuberculosis diagnosis and subsequent treatment in patients with HIV/AIDS. However, the advanced immunosuppression, together with non-compliance to treatment may associate an unfavorable evolution.

**Consent**

Written informed consent was obtained from the next of kin for publication of this Case report and any accompanying images. A copy of the written consent is available for review by the Editor of this journal.

### A57 Evolution of antiretroviral regimens in naϊve patients in 2016

#### Ramona Zamfir^1^, Alina Angelescu^1^, Alena Andreea Popa^1^, Raluca Jipa^1,2^, Ruxandra Moroti^1,2^, Adriana Hristea^1,2^, Liana Gavriliu^1,2^, Șerban Benea^1,2^, Elisabeta Benea^1,2^

##### ^1^National Institute for Infectious Diseases “Prof. Dr. Matei Balș”, Bucharest, Romania; ^2^Carol Davila University of Medicine and Pharmacy, Bucharest, Romania

###### **Correspondence:** Elisabeta Benea (oebenea@yahoo.com)

**Background**

The introduction of new antiretroviral drugs in therapeutic practice has led to changing antiretroviral regimens recommended for naϊve patients with HIV infection. We aimed to identify the main therapeutic changes in naϊve patients diagnosed with HIV infection in 2016 at the National Institute for Infectious Diseases “Prof. Dr. Matei Balș”.

**Methods**

Retrospective analysis of demographic and therapeutic characteristics in patients diagnosed with HIV infection in the period 1 January to 30 June 2016. The data obtained were compared with data of patients diagnosed with HIV infection in 2012–2013. It aimed to identify statistically significant changes (p<0.05) using Epi Info version 7.2.

**Results**

In the analyzed period, 111 adults were confirmed with HIV infection compared to 499 patients in 2012–2013. Antiretroviral treatment was administered to 84 patients (75.68 %) vs 243 (48.69 %) in the control group; p<0.05 (CI 95: 1.353–1.785). The mean age of patients was 36.38 years (18–78) vs 33.39 years (16–72) in the control group. Males accounted for 79.28 % (69/84 patients) vs 62.26 % (161/243 patients) in the control group. Treatment was initiated with the following scheme:

- 2 NRTIs + NNRTI: 12/84 patients (10.81 %) vs 85/243 (34.98 %); p<0.05 (CI 95: 0.2354 – 0.7087),
- 2 NRTIs + PIs/r: 47/84 patients (42.34 %) vs 139/243 (57.20 %),
- 2 NRTIs + integrase inhibitors: 20/84 patients (18.02 %) vs 18/243 (7.41 %); p<0.05 (CI 95: 1.788 – 5.778)
- other schemes: 5/84 patients (4.50 %) vs 1/243 (0.41 %).

At initiation of the treatment the mean value of CD4 was similar (260 cells/cmm) and the proportion of patients with CD4 counts below 200 cells/cmm was 48.15 % (13/84) vs 45.27 % (110/243). Mean viral load was much smaller (152 391 c/mL vs 543 518 c/mL) in the group analyzed.

**Conclusions**

The main changes identified in patients newly diagnosed with HIV infection in the first 6 months of 2016 were: significantly higher percentage of those receiving antiretroviral treatment [75.68 % vs 48.69 %; p<0.05 (CI 95: 1.353 – 1.785)], a significant decrease in the weight of schemes with NNRTIs (10.81 % vs 7.41 %) and a significantly increase of the weight of schemes with integrase inhibitors (18.02 % vs 7.41 %). The percentage of schemes with PIs/r remained constant, these schemes being preferred in our Institute. Detection of new cases in the early stages of HIV infection is still delayed; 48.15 % of our patients had a CD4 count below 200 cells/cmm.

### A58 The unfavorable risk factors for HIV infected persons with positive blood cultures hospitalized at the National Institute for Infectious Diseases “Prof. Dr. Matei Balș” in 2015

#### Alena-Andreea Popa^1^, Georgeta Ducu^1^, Daniela Camburu^1^, Alina Cozma^1^, Manuela Podani^1^, Roxana Dumitriu^1^, Liana Gavriliu^1,2^, Șerban Benea^1,2^, Elisabeta Benea^1,2^

##### ^1^National Institute for Infectious Diseases “Prof. Dr. Matei Balș”, Bucharest, Romania; ^2^Carol Davila University of Medicine and Pharmacy, Bucharest, Romania

###### **Correspondence:** Elisabeta Benea (oebenea@yahoo.com)

**Background**

Systemic infections are commonly encountered disorders in HIV-infected patients and they influence their risk of death. We aimed to analyze the risk factors associated with unfavorable evolution in these patients.

**Methods**

Retrospective analysis of demographic, clinical course and treatment characteristics in patients with HIV infection hospitalized at “Prof. Dr. Matei Balș” Institute in the period 1 January to 31 December 2015 which had positive blood cultures during hospitalization. We watched accurate risk factors associated with death by identifying statistically significant difference (p<0.05) using Epi Info version 7.2.

**Results**

In the analyzed period 62 patients were identified with HIV infection hospitalized who had positive blood cultures during hospitalization; 11 of them have died and 51 have evolved favorably. The average age of the deceased patients was 36.7 years vs. 33.8 years (p<0.05) and the percentage of males was higher in both groups (90.9 % vs. 74.5 %). The acute episode appeared on average after 4.2 years after the detection of HIV infection in those who died vs. 2.8 years (p<0.05). Only 3/11 patients (27.3 %) received antiretroviral (ARV) treatment in history vs. 23/51 (45.1 %). The following entry gates were identified: respiratory (63.6 % vs. 15.7 %), skin (18.2 % vs. 37.3 %), digestive (9.1 % vs. 15.7 %), urinary (0 vs. 3.9 %), catheter IV (0 vs. 3.9 %), unspecified (9.1 % vs. 23.5 %). Only respiratory entry gate was significantly associated with risk of death (p<0.05; CI 95: 1.86–8.82). No significant differences were identified in terms of weight isolated microorganisms (AFB: 38.5 % vs. 16.9 %; GPC: 38.5 % vs. 47.5 %; GPB: 0 vs. 3.4 %; GNB: 7.7 % vs. 28.8 % and fungi: 15.4 % vs. 3.4 %. 6/13 microorganisms had resistance mechanisms to classical antibiotics and antifungals (46.2 %) vs. 19/59 (32.2 %). The average level of CD4 count was significantly lower in the group of HIV deceased (16 cells/cmm vs. 134 cells/cmm; p<0.05), the mean HIV-RNA was significantly lower (299 038 c/mL vs. 844 871 c/mL; p<0.05) and average value of procalcitonin was higher (69.81 pg/mL vs. 19.93 pg/mL). The average length of hospitalization was significantly lower in those who died (11 vs. 20.5 days).

**Conclusions**

Risk factors associated with death in HIV-infected patients with positive blood cultures were: older age of the patient, longer duration of evolution of HIV uncontrolled infection, respiratory entry gate of systemic infection, severe immunosuppression (CD4 count below 50 cells/cmm) and short duration of hospitalization.

### A59 Epidemiological aspects of HIV infection in Oltenia region

#### Andreea Cristina Stoian, Florentina Dumitrescu, Augustin Cupșa, Lucian Giubelan, Irina Niculescu, Loredana Ionescu, Livia Dragonu

##### University of Medicine and Pharmacy from Craiova, Craiova, Romania

###### **Correspondence:** Andreea Cristina Stoian (andreea_plr@yahoo.com)

Objectives: Analysis of epidemiological aspects of HIV infection.

**Methods**

A retrospective study, between 01/01/2007-31/12/2015, in the Craiova Regional Center (CRC), Romania, on two lots of HIV infected persons (HIP): group A-153 HIP, new cases, diagnosed in 01/01/2010-31/12/2015 period and group B-113 HIP, new cases, diagnosed in 01/01/2007-31/12/2009 period.

**Results**

Demographics: group A-F/M = 46 (30.06 %)/107 (69.94 %) vs group B-F/M = 61/52(53.99 %/46.01 %); p = 0.0001. Chronological distribution of new cases show a top of incidence in 2008 (46 cases-40.71 %) and in 2015 (46 cases-30.07 %). The main mode of transmission was sexual-group A vs group B (89.55 % vs 50.44 %); p = 0.0001. Children born from HIV infected mothers were: group A vs group B-5 (3.26 %) vs 2 (1.76 %). Viral-immune status, at the time of HIV diagnosis, was: group A vs group B-average of lymphocytes CD4 = 311.147 ± 97.52 cells/cmm vs 209.22 ± 63.58 (p < 0.0001) and an average of viremia HIV = 5.29 log copies/mL vs 5.12 log copies/mL (p < 0.0001). The most frequent opportunistic infection was tuberculosis-34 HIP (22.22 %) in group A vs 31 HIP (27.43 %) in group B. Cases of HIP late presenters, were: group A vs group B-78 HIP (50.98 %) vs 67 HIP (52.92 %); p = 0.29. In the first 6 months from HIV diagnosis, 15 HIP (9.81 %) from group A vs 14 HIP (12.38 %) from group B, died; p = 0.55.

**Conclusions**

In Oltenia region, new cases of HIV infection were most frequently in men and the predominant mode of transmission was heterosexual; HIP late presenters were present in two groups of patients. Tuberculosis is a major cause of morbi-mortality.

### A60 HIV risk behaviors and prevalence among patients in methadone maintenance therapy (MMT) from Arena center, Bucharest

#### Adrian Octavian Abagiu^1,2^, Loredana Nicoleta Stoica^1^, Catrinel Blaga^1^, Archontis Koulosousas^2^, Roxana Ștefănescu^2^, Alice Atomoaie^1^, Florentina Paraschiv^1^, Florin Matache Duna^1^

##### ^1^National Institute for Infectious Diseases “Prof. Dr. Matei Balș”, Bucharest, Romania; ^2^Romanian Anti-AIDS Association (ARAS), Bucharest, Romania

###### **Correspondence:** Adrian Octavian Abagiu (adyaba@gmail.com)

**Background**

The HIV epidemic in Romania is increasingly driven in the last years by intravenous drug users (IDUs) and men having sex with men (MSM). This study compared sex and injection risk behaviors among male and female IDUs admitted in MMT in our center.

**Methods**

From July 2015 through June 2016, all new patients admitted in MMT (136) were asked to complete a questionnaire regarding HIV risk behaviors and were then tested for HIV, HCV and HBV.

**Results**

24 % of the sample were female and were more likely to be in a stable relationship 49 %, than men 34 %. There were no significant differences between the ages of females vs. males, with the average age being about 34.5 years old. Males reported more daily median injecting than females in the 30 days prior to entering treatment (5 vs. 3) and also report having more STD (38 % vs. 31 %). Females had higher HIV-positive serology results than males (36 % vs. 31 %). On HIV risk, females reported using a common container (55 % vs. 46 %) more frequently. Although male participants reported more sex with multiple partners (23 % vs. 17 %), females reported significantly more sex without a condom (63 % vs. 47 %) and more sex with an IDU partner (65 % vs. 42 %).

**Conclusions**

There is a high risk behavior and HIV prevalence among IDUs. Thus there is a need for rapid introduction of interventions to address this problem.

### A61 Therapeutic options in a case of severe psoriasis associated with both HIV infection and hepatitis C virus previously treated with fumaric acid esters

#### Rodica Olteanu^1^, Roxana Ion^1^, Alexandra Zota^1^, Isra Ennour Jaballah^1^, Lara Mahfoud^1^, Georgeta Preda^2^, Magda Constantin^1^

##### ^1^Dermatology Department, Colentina Clinical Hospital, Bucharest, Romania; ^2^National Institute for Infectious Diseases “Prof. Dr. Matei Balș”, Bucharest, Romania

###### **Correspondence:** Roxana Ion (ion.roxanaa@gmail.com)

**Background**

Psoriasis represents a chronic, immune-mediated skin condition with genetic backgrounds characterized by sharply defined erythematous, scaly plaques with limited or extensive involvement. However, there is a challenge when the patient presents with other diseases and treatment must be adapted carefully, especially when dealing with a patient that has both HIV infection and hepatitis C virus.

**Case report**

Herein we describe a case of a 35-year-old Caucasian male, with a personal history of psoriasis vulgaris with the onset at the age of 20. The patient received treatment with fumaric acid esters for 4 weeks in Germany, with a favorable clinical response, but due to financial issues the treatment was discontinued.

He was admitted to our dermatology department for the assessment of a generalized skin eruption involving the trunk, buttocks, arms, feet and the scalp, characterized by pruritic, sharply demarcated erythematous plaques, covered by silvery scale with a tendency to develop erythoderma and inverse psoriasis. The fingernails were affected and also the distal interphalangeal joints.

The patient was also tested for HIV, viral hepatitis and syphilis. The results showed the presence of HIV infection and hepatitis C. As far as treatment was concerned during the hospitalization, topical therapy was applied including emollients, keratolytics and potent corticoids with a good clinical outcome.

Due to the laboratory findings, the patient was referred to the infectious disease specialist in order to continue the investigations and start treating the HIV infection and viral hepatitis C. Moderate to severe cases can be treated with topical therapy, phototherapy as a first line and as a second line oral retinoids can be an option with careful monitoring for potential adverse events. More refractory and severe disease can be treated with TNF-alpha inhibitors. The positive role of the highly active antiretroviral therapy was observed because it led to the improvement of the psoriasis in our patient too.

**Conclusions**

HIV associated psoriasis is often refractory to traditional therapies, but when the patient has also hepatitis C virus, treatment is even more challenging as it requires careful consideration. The particularity of this case is represented by the fact that the patient was not tested for HIV infection before being treated with fumaric acid esters. The generalized psoriasis eruption in a young patient should lead to more investigations including HIV testing.

**Consent**

Written informed consent was obtained from the patient for publication of this Case report and any accompanying images. A copy of the written consent is available for review by the Editor of this journal.

## HIV infection - immune activation, aging with HIV

### A62 Prevalence of autoantibodies against gangliosides in asymptomatic HIV-infected patients

#### Ilinca Nicolae^1^, Corina Daniela Ene^2^, Mădălina Irina Mitran^1^, Vasile Benea^1^, Mircea Tampa^1,3^, Simona Roxana Georgescu^1,3^

##### ^1^Clinical Hospital of Infectious and Tropical Diseases “Dr. Victor Babeș”, Bucharest, Romania; ^2^Clinical Hospital of Nephrology, “Dr. Carol Davila”, Bucharest, Romania; ^3^Carol Davila University of Medicine and Pharmacy, Bucharest, Romania

###### **Correspondence:** Mădălina Irina Mitran (madalina.irina.mitran@gmail.com)

**Background**

The authors focused on the characterization of the humoral immune profile developed against gangliosides in asymptomatic HIV-infected patients. Host immune response to the viral infection might be influenced by these antibodies.

**Methods**

We investigated 32 asymptomatic HIV-infected patients (with no treatment, without neurologic manifestations) and 32 non-HIV-infected volunteers. The determination of antiganglioside antibodies of IgM type directed against GM1, GM2, GM3, GD1a, GD1b, GT1b, GQ1b was performed by immunoblot technique, using EUROLine kits. The evaluation of the results was realized using EUROLine scan software. After reading the signal intensity on the strips marked with ganglioside antigens, the interpretation of the results was made. A cut-off value equal to 10 was established (according to manufacturer’s instructions). The results were considered positive at values of the signal intensity greater than 10, and negative at values lower than 10.

**Results**

Antiganglioside autoantibodies identified in asymptomatic HIV-infected patients and in the control group exhibit different serologic profiles. In HIV infected patients the prevalence of autoantibodies of IgM type against gangliosides was as follows: 28.12 % anti-GM1, 6.25 % anti-GM2, 9.37 % anti-GM3, 6.25 % anti-GD1a, 3.12 % anti-GD1b, 0 % anti-GT1b, 3.12 % anti-GQ1b. In the control group antiganglioside antibodies of IgM type were absent.

**Conclusions**

The authors consider that gangliosides expressed on the membrane of the HIV-infected cells might induce the synthesis of antiganglioside antibodies. The presence of anti-ganglioside antibodies seems to be a primary immunological event in HIV infection and might play a physiopathological role in the studied viral infection. The ability of the host to produce an early antigangliosidic response might be seen as a defense mechanism directed to the elimination of a danger signal.

### A63 Subclinical inflammation in HIV-infected patients undergoing antiretroviral therapy – a cross sectional study

#### Iulia Cristina Bodoșca^1^, Cristina Murariu^1^, Cătălin Tilișcan^1,2^, Victoria Aramă^1,2^, Cristina Popescu^1,2^, Daniela Munteanu^1^, Mihaela Rădulescu^1,2^, Violeta Molagic^1^, Raluca Năstase^1^, Alina Orfanu^1,2^, Anca Leuștean^1^, Remulus Catană^1,2^, Anca Negru^1,2^, Adrian Streinu-Cercel^1,2^, Sorin Aramă^1,2^

##### ^1^National Institute for Infectious Diseases “Prof. Dr. Matei Balș”, Bucharest, Romania; ^2^Carol Davila University of Medicine and Pharmacy, Bucharest, Romania

###### **Correspondence:** Iulia Cristina Bodoșca (iulia.bodosca@gmail.com)

**Background**

Since its discovery in 1983, HIV-1 has become the most extensively studied pathogen in history. Antiretroviral therapy has markedly improved the quality and the lifespan of people living with HIV. Despite achieving viral suppression many of the HIV infected patients may still harbor persistent low levels of inflammation that may contribute to premature aging and cardiovascular comorbidities. Our objective was to evaluate subclinical chronic inflammation in a cohort of persons living with HIV who were undergoing ART, based on persistent viral replication.

**Methods**

We performed a cross-sectional study in a cohort of Caucasian HIV-1 patients attending the National Institute for Infectious Diseases “Prof. Dr. Matei Balș”, Bucharest, Romania. All included patients were under ART for at least 6 months. Blood samples were tested for: HIV viral load, CD4 cells count, C-reactive protein (CRP), tumor necrosis factor alpha (TNF-alpha) and interleukin-6 (IL-6) levels in all patients. We evaluated inflammatory markers comparatively between two groups: undetectable patients (with HIV viral loads below the level of detection – group 1) and HIV patients with persistent replication (group 2). Independent samples T-test and Mann–Whitney–Wilcoxon test were used for assessing statistical differences between groups.

**Results**

Sixty patients with a mean age of 33.2 (±13.7 years) were included in the study – 60 % were males. The mean time on ART was 80 (±47.8 months). Most patients (71.7 %) had serum HIV loads below the limit of detection (group 1). Mean CD4 counts were 522 (±272.9 cells/cmm) for the entire cohort and 565.4 (±268.3 cells/cmm) in group 1 and 414.2 (±261.2 cells/cmm) in group 2. Median CRP values were 1.79 (IQR = 4.6) mg/L in group 1, compared to 2.57 (IQR = 7.6) mg/L in patients with detectable viremia (without statistical significance, p = 0.92). Mean IL-6 values were 37.2 (±60.7) pg/mL for group 1 vs 66 (±15.5) pg/mL in group 2 (with the level of statistical significance close to the cut-off value, p = 0.06). TNF-alpha mean values were 20 (±14.4) pg/mL in detectable patients (group 2) compared to 16.6 (±17.5) pg/mL in group 2 (p = 0.48).

**Conclusions**

In our cohort undergoing ART we noted similar levels of CRP and TNF-alpha for patients with persistent viral replication and undetectable viremia. The levels of IL-6 seemed to be higher in the group with detectable viremia.

## Pathogenesis of viral infections

### A64 Severe Guillain-Barré syndrome occurring after chickenpox with favorable evolution

#### Iuliana Caramăngiu, Ovidiu Rosca, Monica Cialma, Radu Opreanu, Laurențiu Vochita, Iosif Marincu

##### Dr. Victor Babeş Clinical Hospital of Infectious Diseases and Pneumology, Timişoara, Romania

###### **Correspondence:** Iuliana Caramăngiu (balteanu.iuliana@yahoo.com)

**Background**

Guillain-Barré syndrome, is an acute inflammatory demyelinating polyneuropathy triggered by an acute infectious process. Etiopathogenetically, the condition usually follows a viral infection (cytomegalovirus, Epstein-Barr virus, Varicella-Zoster, herpes). We present a case of acute polyradiculoneuritis, manifested by a pathological picture installed in the evolution of acute varicella (Guillain-Barré syndrome) to an immunocompetent patient.

**Case report**

The authors present the case of a patient aged 37 years with chickenpox, hospitalized in the Clinic of Infectious Diseases “Victor Babeș” Hospital Timișoara, who developed acute tetraparesis and polyradiculoneuritis. Symptoms started two days before admission featuring high fever, malaise and the appearance on the trunk, limbs and face of itchy small blisters “in the drop of dew”, cardio-pulmonary balanced, conscious, with no signs of meningeal irritation. At about 3 weeks of hospitalization the patient presents tetraparetic motor deficit type accompanied by tingling in the limbs, progressive, and is transferred to the Neurology Clinic in the Emergency County Hospital Timișoara. On the day of admission in Neurology, the patient's condition worsens, becomes dyspneic, which is why they decide to transfer her at the Intensive Care Unit where she underwent oro-tracheal intubation with mechanical ventilation. In the intensive care unit, the patient received treatment with immunoglobulin (Octagam 5 fl/day for 5 days), with no significant improvement, then they have done seven sessions of plasmapheresis. Treatment was established with antibiotics, gastric antisecretory, anticoagulants, analgesics, opioids, antipyretics, corticosteroids, vitamins, blood products, solutions and calorie electrolytic rebalancing.

On admission in Neurology biological investigations were carried out, which did not show anything pathological and lumbar puncture was performed (WBC 0/mL, RBC 0/mL, protein 0.54 g/L). Skull and cervical spine MRI did not show anything pathological. Chest radiography highlighted a discrete decrease in transparency in the basal right lung. The result of catheter tip culture showed *Proteus mirabilis* and bronchial aspirate culture was positive for *Klebsiella pneumoniae*. Antibiotic sensitivity was determined and the patient had a favorable evolution.

**Conclusions**

Biological rigorous clinical monitoring, along with an effective interdisciplinary collaboration in chickenpox cases with neurological complications may favorably influence the evolution of the patients.

**Consent**

Written informed consent was obtained from the patient for publication of this Case report and any accompanying images. A copy of the written consent is available for review by the Editor of this journal.

### A65 Echovirus 30 infection with pulmonary and cardiac complications – case report

#### Vlad Murărescu^1^, Marilena Palaghiță^1^, Alina Cristina Neguț^1,2^, Cornel Camburu^1^, Adrian Streinu-Cercel^1,2^

##### ^1^National Institute for Infectious Diseases “Prof. Dr. Matei Balș”, Bucharest, Romania; ^2^Carol Davila University of Medicine and Pharmacy, Bucharest, Romania

###### **Correspondence:** Vlad Murărescu (mura_vlad@yahoo.com)

**Background**

Almost 80 % of the individuals present asymptomatic enterovirus infection with one of the 71 known serotypes. A small percentage of the patients may experience a self-limited non-specific rash along with flu-like symptoms, clinically indistinguishable from other viral infections. Rarely, enteroviruses can be held responsible for severe manifestations such as acute aseptic meningitis, encephalitis, hepatic, pulmonary or cardiac complications.

**Case report**

A 39 year-old man, smoker, with no relevant medical history presented to the hospital for fever (39 °C), chills, dry cough and intense myalgia that persisted after 7-day amoxicillin self-medication. The clinical exam revealed high fever, dyspnea on mild exertion and tachycardia. The lab tests showed intense acute inflammatory syndrome, leukocytosis with lymphocytosis, normochromic-normocytic anemia, moderate cytolysis, high value of N-terminal pro-brain natriuretic peptide (NT-proBNP) (3161 pg/mL) and high antibody titer for Echovirus 30. A chest X-ray showed lesions suggestive for right medial lobe pneumonia, while the cardiology consult with echocardiography diagnosed myocarditis and pericarditis, with an ejection fraction of 40 %. Given the critical state of the patient a bronchoscopy could not be performed at that time point. Before receiving the positive serology for Echovirus 30, his clinical state continued to deteriorate despite adequate cardiologic and antibiotic therapy (levofloxacin and ertapenem), so high-dose corticotherapy (24 milligrams of dexamethasone per day for 3 weeks) was added, with slow improvement of both clinical and biological parameters. After two months of apparently good health, he presented to the hospital for the same clinical and biological findings, but having stable cardiac function. The CT-scan revealed alveolar condensation in the right medial lobe and bronchoalveolar lavage fluid was suggestive for lymphocytic alveolitis. The repeated high dose corticotherapy lead to a slow but steady improvement. All autoimmune markers were negative, and cultures from both sputum and bronchoalveolar fluid were negative for bacteria and fungi.

**Conclusions**

After ruling out bacterial and immunological etiologies two times in a row, echovirus 30 remained the incriminated infectious agent of both lymphocytic alveolitis and cardiac complications. While literature data shows that severe enterovirus infections are mostly illustrated by aseptic meningitis, our case reveals how an echovirus 30 infection can be responsible for severe cardiac complications (myocarditis and pericarditis), but also for recurrent lymphocytic alveolitis, both in the same patient.

**Consent**

Written informed consent was obtained from the patient for publication of this Case report and any accompanying images. A copy of the written consent is available for review by the Editor of this journal.

### A66 Herpetic encephalitis with favorable evolution in an adult immunocompetent patient

#### Irina Duşan, Patricia Poptelecan, Bogdan Trincă, Sorina Mitrescu, Livius Tirnea, Iosif Marincu

##### Department of Infectious Diseases, Pneumology and Parasitology, Dr. Victor Babeş Clinical Hospital of Infectious Diseases and Pneumology, Timişoara, Romania

###### **Correspondence:** Irina Duşan (irinadusan@yahoo.com)

**Backgound**

Although remarkable progress was made on brain imagistic investigations along with the diversification of antiviral therapy, herpetic encephalitis remains a brain disease with poor prognosis and high mortality risk. The purpose is to present a clinical case of herpetic encephalitis occurred in an adult immunocompetent female patient, with favorable evolution

**Case report**

The authors present the case of an adult patient, presenting six days prior to admission: headache, confusional syndrome, nausea, diarrhea, severe fatigue. The patient presented to the County Hospital, Timișoara where she is admitted to the Neurology Clinic. In order to put the correct diagnosis, biological investigations (blood cell identification, ESR, fibrinogen, blood glucose, C-reactive protein, urine analysis, blood cultures, serum urea and creatinine, urine culture, exudate lingual for yeast, etc.) and paraclinical investigations (cerebral computed tomography (CT), cerebral MRI, lumbar puncture) have been made. Based on clinical data in conjunction with the results of biological samples and laboratory investigations, the diagnosis of herpetic encephalitis is established and the patient is transferred to the Clinic of Infectious Diseases for therapeutic and clinical monitoring. Physical examination at admission to our clinic reveals: afebrile patient with moderate influenced general state, temporo-spatial disorientation, but without meningeal signs, tongue with whitish deposits, cardio-pulmonary and digestive systems with no pathological sings. Lumbar puncture was performed and antiviral and symptomatic treatment was set. WBC, RBC, PLT = normal values; neutrophils = 88.2 %, lymphocytes = 6.6 %; lumbar puncture: clear normotensive liquid, glucose = 73 mg/dL, proteins = 0.5 g/L, erythrocytes = 55, leukocytes = 0; cerebral MRI: cortico-subcortical lesion in the uncus, partial temporal lobe (anterior- internal), the left island with no capture, with aspect suggesting herpetic encephalitis. The result of the blood culture and CSF culture for bacterial flora and BK were negative, so the bacterial etiology was excluded. Lingual exudate for yeast *Candida albicans* was positive. The treatment consisted in: Acyclovir 250 mg, 3x2 fl/day, Ceftriaxone 1 g, 2x2g/day, Fluconazole 200 mg, 1tb/day, Dexamethasone, Mannitol, vitamin B6, symptomatic agents and solutions for hydro-electrolytic rebalancing. The patient’s state was favorable, the headache remitted after three days and the confusional syndrome disappeared after six day of hospitalization.

**Conclusions**

Early diagnosis of herpetic encephalitis along with specific antiviral therapy can favorably influence the evolution of the disease in immunocompetent adults with this brain disorder known with a high potential of severity.

**Consent**

Written informed consent was obtained from the patient for publication of this Case report and any accompanying images. A copy of the written consent is available for review by the Editor of this journal.

### A67 Clinical-evolutional aspects in present-day measles

#### Narcisa Nicolescu^1^, Alexandru Crișan^1^, Voichița Lăzureanu^1^, Ruxandra Laza^1^, Virgil Musta^1^, Adelina-Raluca Marinescu^1^, Andreea Bîrlad^2^

##### ^1^Dr. Victor Babeş University of Medicine and Pharmacy, Timişoara, Romania; ^2^Dr. Victor Babeş Clinical Hospital of Infectious Diseases and Pneumology, Timişoara, Romania

###### **Correspondence:** Narcisa Nicolescu (narcisa.nicolescu@yahoo.com)

**Background**

Measles represent a problem in current infectious pathologies through the possibility of appearance of severe forms of measles and/or some types of patients. The absence of vaccination due to various reasons leads to spread of this disease in the receptive population and the evolution of infections (center) or small epidemics (outbreaks). Objectives: 1. The analysis of incidence of measles in the last years in Timiș County; 2. Comparison with the situation from all our country and other countries; 3. Complex analysis regarding conditions for developing severe forms or complications; 4. Concrete measures regarding the evaluation of incidence of this disease.

**Methods**

We have performed many studies regarding cases of measles during the last years hospitalized in Clinic II of Infectious Diseases of V. Babeș Hospital Timișoara.

**Results**

Measles is highly contagious and can occur anytime, but that can be prevented by vaccination. It is to be emphasized that in just three months of 2016 (May-July) 31 cases of measles were counted in our hospital. The most affected age group in 2016 was between 1 to 3 years, which represents 48.3 % of all cases. In the latest years much more atypical forms of measles have been recorded, some of them severe, predominantly with pulmonary complications, even children with interstitial lung disease. In 30 countries from Europe, measles cases didn’t occur in 2014. Likewise in Romania, Timiș county, measles wasn’t present in 2008/2009 and 2013/2014.

**Conclusions**

This is a disease which can be prevented by following the vaccination programs.

### A68 Pneumococcal superinfection in children with influenza

#### Victor Daniel Miron^1^, Anca Cristina Drăgănescu^2^, Constanța-Angelica Vișan^1,2^, Anuța Bilașco^2^, Daniela Pițigoi^1,2^, Oana Săndulescu^1,2^, Monica Luminița Luminos^1,2^

##### ^1^Carol Davila University of Medicine and Pharmacy, Bucharest, Romania; ^2^National Institute for Infectious Diseases “Prof. Dr. Matei Balș”, Bucharest, Romania

###### **Correspondence:** Oana Săndulescu (oanasandulescu1@gmail.com)

**Background**

The pathogenesis of influenza includes an impairment of pulmonary defenses which is said to “open the gates” for bacterial superinfection, and *Streptococcus pneumoniae* is one the leading pathogens of superinfection, particularly in children without history of anti-pneumococcal vaccination.

**Methods**

We have performed a retrospective study of the 2015–2016 influenza season in one pediatrics ward of a tertiary-care hospital in Bucharest, Romania. We collected demographic, clinical and microbiological data regarding influenza disease characteristics and outcome, and regarding the incidence and outcome of pneumococcal colonization/infection. We hereby report the preliminary results of this study.

**Results**

The study included 206 cases of influenza, occurring in children with a median (interquartile range) age of 4.2 (2.5, 6.5) years. The influenza season started late December and lasted until late May, with A viruses being predominant (92.4 %). Complicated influenza was diagnosed in 73.9 % of the cases, and this was also the main presenting complaint. Complications included: myositis (24 %), encephalitis (3.9 %), other central nervous system complications (2.8 %), viral pneumonia (61 %), bacterial superinfection pneumonia (6.7 %), acute otitis media (6.1 %). Eight (3.9 %) patients with influenza presented positive nasal cultures and one other case (0.5 %) presented acute pneumococcal otitis media, identified after a median (IQR) duration of influenza illness of 6 (4, 7) days. In patients with influenza plus pneumococcal colonization the number of complications was significantly higher (median 2 vs. 1, p = 0.037, U = 416). The erythrocyte sedimentation rate was a good indicator of pneumococcal presence (median 26 vs. 16 mm/h, p = 0.038, U = 197), as opposed to fibrinogen (median 281 vs. 266 mg/dL, p = 0.466, U = 545.5) or C-reactive protein (median 4.2 vs. 3.2 mg/L, p = 0.589, U = 603). Importantly, the duration of hospital admission was significantly longer (median 6.5 vs. 5 days, p = 0.026, U = 424.5) in cases with concomitant pneumococcal colonization.

**Conclusions**

We have identified a low prevalence of pneumococcal colonization in pediatric patients with influenza. However, when colonization was present, it associated a significantly longer hospital stay and a significantly higher number of complications, highlighting the need for close monitoring and screening for pneumococcal colonization in children.

**Acknowledgements**

1) This abstract is part of the license thesis “Epidemiology and etiopathogenesis of influenza and pneumococcal colonization in children – clinical, paraclinical and microbiological characteristics” performed at the Carol Davila University of Medicine and Pharmacy, Bucharest, Romania. Coordinator: Prof. Dr. Adrian Streinu-Cercel; Supervisor: Dr. Oana Săndulescu.

2) “Romanian Center for Applied Bio-Molecular Research in Infectious Diseases”, project financed through the Sectoral Operational Programme Increasing of Economic Competitiveness (POS CCE).

### A69 Varicella complicated with transverse myelitis - case presentation

#### Monica Luminos^1,2^, Endis Osman^2^, Magdalena Vasile^2^, Anca Cristina Drăgănescu^2^, Constanța-Angelica Vișan^1,2^, Anuța Bilașco^2^, Camelia Kouris^2^, Sabina Șchiopu^2^, Mădălina Merișescu^1,2^

##### ^1^Carol Davila University of Medicine and Pharmacy, Bucharest, Romania; ^2^National Institute for Infectious Diseases “Prof. Dr. Matei Balș”, Bucharest, Romania

###### **Correspondence:** Monica Luminos (luminosmonica@gmail.com)

**Background**

Varicella is a highly contagious infectious disease, frequently encountered during childhood, with general receptivity and most often, with self-limited evolution. There are cases when varicella can present severe complications, such as acute encephalitis, cerebellitis, and rarely myelitis. Often, the host has a previous immunosuppression, such complications being rare in patients with a normal immunological status.

**Case report**

We present the case of a 10 year old girl, diagnosed 7 days prior with varicella, without any significant medical history, admitted in the Pediatric Intensive Care Unit of the National Institute for Infectious Diseases “Prof. Dr. Matei Balș” for lower back pain, myalgias, muscle weakness, difficulty in walking. The onset of the symptoms was 3 days prior with an evolution towards exacerbation. Clinical exam upon admission confirmed varicella and furthermore, urinary retention with enlarged bladder, diminished patellar, right plantar clonus, decrease in muscle strength, myalgias, and paresthesias, coordination impairment in the lower limbs and asymmetric paraparesis predominantly on the right side. Workup was within the normal range. Myelitis was suspected and therefore a MRI of the head and spine was performed, which showed medullar inflammatory lesions in the cervicothoracic segment. Lumbar puncture was normal, except for a positive varicella-zoster virus PCR from CSF. Positive diagnosis consisted of cervicothoracic varicella-zoster virus myelitis. Treatment with acyclovir (60 mg/kg/day), ceftriaxone 2 g/day, corticotherapy with methylprednisolone, human immunoglobulin, and iv fluids was promptly instituted for 14 days, with a slow favorable evolution. Paresthesias, motor deficit, urinary retention and sensitivity impairment resolved under treatment.

**Conclusions**

Although this patient had a normal immunological status, she presented a severe complication of varicella, which required antiviral and cortisone treatment in high doses for an extended period of time, which eventually led to complete recovery.

**Consent**

Written informed consent was obtained from the parents for publication of this Case report and any accompanying images. A copy of the written consent is available for review by the Editor of this journal.

### A70 Clinical forms of enterovirus infections during the summer season of 2016

#### Monica Luminos^1,2^, Anca Cristina Drăgănescu^2^, Constanța-Angelica Vișan^1,2^, Anuța Bilașco^2^, Camelia Kouris^2^, Endis Osman^2^, Sabina Vintilă^2^, Magda Vasile^2^, Mădălina Merișescu^1,2^

##### ^1^Carol Davila University of Medicine and Pharmacy, Bucharest, Romania; ^2^National Institute for Infectious Diseases “Prof. Dr. Matei Balș”, Bucharest, Romania

###### **Correspondence:** Monica Luminos (madalinamerisescu@gmail.com)

**Background**

Enteroviruses, a group of single-stranded RNA viruses, are commonly encountered infectious agents, especially in infants and children. They are responsible for a broad array of clinical manifestations, from herpangina and hand-foot-and-mouth disease (HFMD), to myocarditis, aseptic meningitis.

**Methods**

Faced with an increased number of enteroviruses associated infections, during the summer season of 2016, the present paper analyzes the managed cases in the pediatric department of the National Institute for Infectious Diseases “Prof. Dr. Matei Balș” – Bucharest, referring specially to the severe and challenging cases, particularly towards the ones with associated meningoencephalitis.

**Results**

The simplest forms of enterovirus infections registered were cases of hand-foot-and-mouth disease, that only required hospital management secondary to dehydration or to associated gastroenteritis; the registered number of HFMD exceeded 170 cases, representing over 80 % from enteroviruses induced infections. There was no record of secondary myocarditis, but an overwhelming number of acute viral meningoencephalitis cases (24) were reported, some of them undergoing severe forms of the meningoencephalitis syndrome, complicated by convulsive status, plegias and/or important motor deficits. The epidemic peak was reached during the mid-summer season (June-July) when the integrated pediatric intensive care (PIC) department managed 20 out of the 24 registered cases of the 2016 season. Important central nervous system involvement, with magnetic resonance imaging documented lesions, was present in 5 patients, but no fatal cases were registered.

**Conclusions**

Traditionally enterovirus infections present with benign symptoms, taking the form of uncomplicated summer illnesses; during the summer of 2016 we registered a high number of severe, life threatening, forms of manifestation which required initial PIC management, long hospital management and, in some particular case of meningoencephalitis with associated motor sequelae, kinesiotherapy and physical therapy, fact that highlighted the associated burden of these summer infections.

## Comorbidities in viral infection

### A71 Face off – HIV and lymphoma – case series presentation

#### Liana Cătălina Gavriliu^1,2^, Otilia Elisabeta Benea^1,2^, Alina Angelescu^2^, Ramona Zamfir^2^, Daniela Camburu^2^, Georgeta Ducu^2^, Alina Cozma^2^, Roxana Dumitriu^2^, Manuela Podani^2^, Șerban Benea^1,2^, Mihaela Ionică^2^

##### ^1^Carol Davila University of Medicine and Pharmacy, Bucharest, Romania; ^2^National Institute for Infectious Diseases “Prof. Dr. Matei Balș”, Bucharest, Romania

###### **Correspondence:** Liana Cătălina Gavriliu (lianagavriliu@yahoo.com)

**Background**

Combined antiretroviral therapy significantly improved the prognosis and the life expectancy of HIV patients. Along with this, the profile of HIV associated malignant pathologies has changed. Among them, lymphoma, having a higher incidence and a poorer prognosis than in general population, is an important cause of mortality and morbidity in HIV infected people.

**Case report**

We present two cases of HIV-associated lymphoma with different outcome. In the first case, a newly HIV diagnosed patient, with a good immunological status (lymphocytes CD4 count 442/cmm, HIV-RNA = 193502 copies/mL), developed non-Hodgkin’s Burkitt-type lymphoma and he had a fulminant evolution towards death. In the second case, a patient known for over twenty years with HIV infection and who had discordant immunological and virological response to antiretroviral therapy (lymphocytes CD4 count 19/cmm, undetectable HIV-RNA), was diagnosed with Hodgkin’s lymphoma and he had a favorable evolution with chemotherapy. Both patients presented with systemic extranodal involvement, B symptoms and were classified in stage IV lymphoma. The extranodal involvement included central nervous system and liver in the case of the patient with non-Hodgkin’s Burkitt-type lymphoma and liver and spleen involvement in the case of patient with Hodgkin’s lymphoma. Both patients required exhaustive medical investigations and a close interdisciplinary cooperation during the entire time of diagnostic and therapeutic procedures.

**Conclusions**

HIV-associated lymphoma manifests in various forms in clinical settings. Unlike in general population, HIV-associated lymphoma is usually more aggressive and has a poor prognostic. Therefore it must be included into the differential diagnosis panel in patients presenting with sudden increase in lymph nodes, neurological manifestation and abrupt decrease in CD4 lymphocytes count. HIV-associated lymphoma differential diagnosis involves a wide panel of infectious and oncologic pathologies, therefore extensive investigation and the implication of specialists from various medical fields are required. A fast and accurate diagnosis may warrant the future evolution of the patients.

**Consent**

Written informed consent was obtained from the patient/from the next of kin for publication of this Case report and any accompanying images. A copy of the written consent is available for review by the Editor of this journal.

### A72 Coxsackie infection complicated by pancytopenia – pediatric case report

#### Gheorghiță Jugulete^1,2^, Adina Stăncescu^1^, Cristina Elena Popescu^1^, Luminița Marin^1,2^, Diana Zaharia^1^, Cristina Dumitrescu^1^, Lucia Tudor^1^, Sabina Vintilă^1^

##### ^1^National Institute for Infectious Diseases “Prof. Dr. Matei Balș”, Bucharest, Romania; ^2^Carol Davila University of Medicine and Pharmacy, Bucharest, Romania

###### **Correspondence:** Gheorghiță Jugulete (georgejugulete@yahoo.com)

**Background**

Among immunocompetent hosts the enteroviral infections are most frequently benign and auto-limited illnesses. Exceptionally these summer viral pathologies can evolve dramatically, presenting under severe forms of disease, with important associated complications sometimes leading to death. In the present paper we present the clinical evolution of an acute Coxsackie virus infection in an immunocompetent child, complicated by pancytopenia.

**Case report**

A young baby girl, 11 months of age, without any particular pathologic medical history is submitted in the 9^th^ Pediatric Department at the National Institute for Infectious Diseases “Prof. Dr. Matei Balș” for fever, poor feeding, sleepiness and diarrhea. The clinical onset, registered 48 hours before her hospital submission was characterized by fever and poor feeding. The initial clinical evolution found a moderately ill appearing child, with pallor identified, without associated petechiae or bruising and without any other particular exanthematous elements, with a stable cardio-circulatory status, with no clinical signs of renal function impairment, diarrheal stools emitted, no nuchal rigidity or neurological focal signs present. The laboratory investigations isolated a franc pancytopenia (NL = 1.600/cmm, Hb = 8.8 g/dL, PLT = 105.000/cmm), with no other pathological findings. The serological screening for HIV, HTLV, adenovirus, EBV, CMV, Parvovirus, Influenza came back negative, with a normal peripheral smear and bone marrow aspiration. No clinical relevant result was communicated by the microbiological laboratory (negative hemocultures, nasal and pharyngeal swabs, uroculture and stool cultures). A positive IgM serology for Coxsackie virus was obtained. Cortisone, i.v. immunoglobulin, endovenous hydration and supportive therapy were initiated, with subsequent favorable evolution, with rapid normalization of the hemogram parameters (a discrete hypochromic anemia persists at a level previously identified in the infant) and clinical restoration.

**Conclusions**

The acute Coxsackie virus infection in the immunocompetent pediatric population can sometimes evolve severely, complicated by acute diarrheal manifestations with subsequent dehydration and possible hematological involvement. In evolution the presented case maintained a residual anemia (Hb = 10.8 g/dL), with no other para-clinical imbalances.

**Consent**

Written informed consent was obtained from the patient’s next of kin for publication of this Case report and any accompanying images. A copy of the written consent is available for review by the Editor of this journal.

### A73 Viral respiratory infections in children in the season 2015–2016

#### Constanța-Angelica Vișan^1^, Anca Cristina Drăgănescu^1^, Anuța Bilașco^1^, Magda Vasile^1^, Mădălina Merișescu^1,2^, Camelia Kouris^1^, Cristina Negulescu^1^, Endis Osman^1^, Diana-Maria Slavu^1^, Sabina Vintilă^1^, Daniela Pițigoi^1,2^, Monica Luminos^1,2^

##### ^1^National Institute for Infectious Diseases “Prof. Dr. Matei Balș”, Bucharest, Romania; ^2^Carol Davila University of Medicine and Pharmacy, Bucharest, Romania

###### **Correspondence:** Constanța-Angelica Vișan (angelica.visan@yahoo.com)

**Background**

Viral respiratory infections are the leading cause of morbidity in children and improving their management is a priority of our clinic. Etiological diagnosis of respiratory viral infections provides the necessary conditions for the proper management and improves clinical outcomes. Objectives: Identification of viral etiology of respiratory infections and establishing correlations between pathogens and clinical manifestations of infections produced in children.

**Methods**

We tested patients aged 0–14 years diagnosed with viral respiratory infections in our clinic between December 1, 2015 - May 31, 2016. Identification of respiratory viruses was achieved by rapid tests (MARI-POC) and by molecular methods - PCR performed in nasal secretions. We recorded changes in biological constants and clinical manifestations caused by respiratory viruses.

**Results**

Identification of etiologic agents allowed appropriate epidemiological measures necessary to isolate the patients and the study of the incidence of seasonal viruses. The season 2015–2016 was dominated by the AH1N1 influenza virus. In much smaller proportions we diagnosed infections with respiratory syncytial virus, influenza B virus, adenovirus and human metapneumovirus. Influenza infection has evolved this season with high severity index. The severe complications of influenza in children were severe neurological diseases represented by febrile seizures, encephalitis and encephalitis reactions. Two cases of encephalitis have progressed to death.

**Conclusions**

The etiological diagnosis of respiratory viral infections has epidemiological and therapeutic implications. Infection with influenza viruses can cause severe complications and deaths, so we need prophylactic measures, early diagnosis and timely treatment administered.

### A74 Hepatocarcinoma – a common complication of liver cirrhosis B and C viral etiology

#### Olga Adriana Caliman-Sturdza

##### Emergency County Hospital “New St. John”, Suceava, Romania

**Background**

Hepatocellular carcinoma (HCC) is the most common malignant tumor of the liver, the overall incidence of approximately 626,000 cases annually, with predominance in males. HCC is considered the third leading cause of death from malignancy and the second leading cause of death respectively in the field of digestive cancers. These patients are 80 % previously diagnosed with hepatitis B or C. The incidence of HCC in patients diagnosed with cirrhosis of viral B and C etiology is increasing, overall survival at 5 years continues to remain below 10 % due on the one hand to the discovery of tumors in advanced stages, ineffective screening in patients with risk factors for HCC and on the other hand to the lack of curative therapies applicable to this category of patients. Curative or palliative treatment of HCC depends mainly on local tumor extension and preexisting liver disease. Angiogenic agents of the type Sorafenib may cause an increase in overall survival by 44 % compared to placebo in patients with advanced HCC.

**Case report**

We present the case of a of 47 years old male, known with cirrhosis of viral etiology B from 2003, consumer of ethanol, who refused antiviral therapy with entecavir in 2009 and was diagnosed in February 2016 with HCC with extrahepatic extension. The patient was admitted in January 2016 at the Suceava Emergency Hospital with jaundice, fever, right upper quadrant pain, loss of appetite, weight loss. Clinical exam: patient with intense jaundice, emaciated, superficial lymph impalpable, ascites fluid quantity average, slightly sensitive right upper quadrant. Laboratory findings: significant hepatic cytolysis, cholestasis, elevated amylase, alpha-fetoprotein slightly elevated, thrombocytopenia, moderate inflammatory syndrome. CT and MRI exam revealed a nodular lesion in segment VI/VII with extra capsular extension and diaphragm and chest wall invasion. Histopathology and immunohistochemistry outlined trabecular hepatocellular carcinoma developed on a background of chronic active hepatitis advanced fibrosis. The patient has no indication for liver transplantation and liver resection, but could benefit from treatment with Sorafenib. The patient devloped important ascites, right massive pleurisy, hepatic encephalopathy phenomena and died 6 months after the diagnosis of HCC and one month after starting treatment with Sorafenib.

**Conclusions**

Current medical treatments are less effective in advanced HCC, and their role after liver transplantation, liver resection, should be explored further. Current hopes are turning to new molecular “target” therapies that explore the biological pathways involved in carcinogenesis signaling in the liver.

**Consent**

Written informed consent was obtained from the next of kin for publication of this Case report and any accompanying images. A copy of the written consent is available for review by the Editor of this journal.

### A75 The severity of A H1N1 Influenza infection in the 2015–2016 season

#### Cleo Roșculeț, Catrinel Olimpia Ciuca, Dalila Toma, Cătălin Apostolescu, Andrei Rogoz, Andrei Stangaciu, Viorica Mitescu, Doina Iovănescu, Cornel Camburu, Bogdana Manu

##### National Institute for Infectious Diseases “Prof. Dr. Matei Balș”, Bucharest, Romania

###### **Correspondence:** Cleo Roșculeț (cleo_n_rosculet@yahoo.com)

**Background**

Influenza seasons represent a major strain on the health care system and they can vary in their severity, timing and duration from one season to another. Most of patients the infected with A H1N1 influenza virus exhibit no symptoms or very mild respiratory symptoms and high fever. In some cases, regardless of age or comorbidities, severe disease and complications due to infection, including death, may occur. Our case series study is meant to analyze the epidemiological, clinical and paraclinical characteristics of patients infected with type A H1N1 Influenza virus, admitted in the Intensive Care Department of INBI “Prof. Dr. Matei Balș” in the 2015-2016 influenza season.

**Methods**

A retrospective, observational case series study was conducted over a 6-month period, on 21 adult patients admitted in the Intensive Care Department, that have been confirmed with severe influenza infection through nasopharyngeal swab and tracheal secretion PCR analysis. In the same time, we collected data on demographics and comorbidities. Clinical data, microbiological samples and imagistics were extracted from patients’ medical charts through thorough analysis. Eventually, therapeutic measures were statistically correlated with the outcome.

**Results**

There were 21 subjects enrolled with median age of 50.6 years old. 57.14 % were male, 100 % Caucasian. High-risk conditions were present in 34 %. The majority of influenza viruses of the patients in the case study matched the strains present in the seasonal vaccine, but none of the patients had been vaccinated. The demographical spectrum impressed through diversity. The mortality rate was 31.8 % taking into account that influenza might have triggered latent disorders or exacerbated chronic afflictions and put the patients at risk of acquiring bacterial superinfections. The data strongly implicate A H1N1 influenza virus infection combined with bacterial superinfection as the primary cause of mortality during the A H1N1 influenza outbreak.

**Conclusions**

The incidence of severe influenza cases in the Intensive Care Department was relatively low, but the severity was high; 28.5 % of the cases might have been prevented by following the recommendation for vaccination of risk groups in Romania.

### A76 Acute respiratory distress syndrome in a child with measles

#### Ana Vaduva-Enoiu^1^, Ramona Georgiana Stanculete^1^, Adelina Raluca Marinescu^1^, Voichita Elena Lazureanu^2^

##### ^1^Dr. Victor Babeş Clinical Hospital of Infectious Diseases and Pneumology, Timişoara, Romania; ^2^Dr. Victor Babeş University of Medicine and Pharmacy, Timişoara, Romania

###### **Correspondence:** Ana Vaduva-Enoiu (annalupu20@yahoo.com)

**Background**

Measles is an acute infectious disease, highly contagious; the death among infants still remains in very high proportion (about 60 %) due to bronchopneumonia. Respiratory diseases are the result of measles virus with associated bacterial infection, which may include any respiratory area, secondary lesions, both viral and immune cellular depression.

**Case report**

We present the case of a child, 11 months old, female, unvaccinated, with multiple hospitalizations for bronchiolitis in the last 3–4 months, suppurative otitis, febrile convulsion syndrome, last hospitalization at “Louis Turcanu” Emergency Hospital for Children Timișoara, 10 days before presentation in our clinic for: emphysematous acute bronchiolitis, otitis media with effusion. Affirmative, 2 days after discharge the patient had fever, nasal obstruction, rhinorrhea, cough and some eruptive elements. The child was guided to our hospital by the general physician. At admission the child presented: influenced general condition, adynamia, nasal obstruction, cough with expiratory 3–4 secuses, saburral tongue, throat congestion, erythemato-macular elements eruptive with tendency to confluence on the face and chest, liver at 2 cm below the lower edge of the costal margin. Biological tests at admission: anemia, inflammatory syndrome present. A diagnosis of measles and bronchial hyperreactivity was suspected (subsequent anti measles IgM positive). Treatment was begun with water and electrolyte rebalancing treatment, bronchodilators, corticosteroids. Antibiotics were administrated after 2 days, due to the persistence of fever (isolated febrile hooks) and positive procalcitonin. After 4 days general condition was altered with polypnea, intercostal draft, oxygen saturation 85 % without oxygen mask, requiring transfer to intensive care. Endotracheal intubation was necessary, because of desaturation episodes, highlighting this radiological image: diffuse fog of both lung fields. In an attempt to extubate, the patient presents pulmonary edema and severe bronchospasm, requiring reintubation after 4 days. After about 2 weeks the child's respiratory status degrades, evolving to acute respiratory distress syndrome with metabolic acidosis and severe episodes of bradycardia. She is mechanically ventilated, but becomes oligoanuric, bradycardia does not respond to atropine then cardiopulmonary arrest occurs.

**Conclusions**

Overall measles prognosis is favorable in children with satisfying nutritional status and good living conditions. Prognosis remains poor in children under 2 years with dystrophic syndromes (anemia, rickets) and multiple comorbidities.

**Consent**

Written informed consent was obtained from the next of kin for publication of this Case report and any accompanying images. A copy of the written consent is available for review by the Editor of this journal.

### A77 Management challenges of right-sided infectious endocarditis in an HIV positive patient – case presentation

#### Elena-Violeta Niță^1^, Sînziana Dumitru^1^, Daniela-Ioana Munteanu^1^, Anca Ruxandra Negru^1,2^, Remulus Catană^1,2^, Ioan Diaconu^1^, Bogdana Manu^1^, Ligia Ionescu^1^, Liliana Ion^1^, Cătălin Tilișcan^1,2^, Victoria Aramă^1,2^

##### ^1^National Institute for Infectious Diseases “Prof. Dr. Matei Balș”, Bucharest, Romania; ^2^Carol Davila University of Medicine and Pharmacy, Bucharest, Romania

###### **Correspondence:** Elena-Violeta Niță (violeta.nita@yahoo.com)

**Background**

Right-sided infectious endocarditis (IE) is a common condition in IV drug users (IVDU), *Staphylococcus* spp. being the most involved pathogen. Many IVDU are HIV positive, condition making them extremely susceptible to infections. Our aim is to present the challenges raised by the management of IE in an HIV positive IVDU.

**Case report**

A 27-year-old former IVDU, currently under methadone substitution, with HCV chronic infection, HIV infection (naive to treatment), was admitted into the hospital for fever (38.3 °C), dyspnea on minimal exertion (SaO_2_ = 87 % spontaneous), cough, generalized arthromyalgia, temporal-spatial disorientation. Taking into account the HIV status correlated with the severe general presentation and important lymphopenia, the patient had inserted a left jugular central venous line (CVL) for easier administration of medication, blood cultures were taken, and he was started on maximal antimicrobial therapy associated with dexamethasone and mannitol to treat a potential meningitis (later ruled out by lumbar puncture). The drug abuse history, the continuously deteriorating state urged for a transthoracic echocardiography (TTE) that revealed 3 vegetations located on the tricuspid valve, evocative for IE. Therapy was adapted for a possible staphylococcal endocarditis (vancomycin IV 2 g q12h 3 days, and gentamycin IV 200 mg single dose 2 days), with diuretic and beta-blocker drugs. A few days later one blood culture came out positive for methicillin-sensitive *S. aureus* (MSSA). The diagnosis was IE with MSSA on tricuspid valve (revised Duke criteria: 1 major criterion - vegetations seen on TTE, 3 minor criteria: predisposing factors, one positive blood culture and fever), the antibiotherapy was changed again to oxacillin IV 12 g per day (7 days). Despite the correct treatment, the patient’s evolution worsened (low fever, intense dyspnea, increased biologic inflammatory syndrome, pulmonary rales), the chest radiography was consistent with pneumonia, bronchoscopy with bronchoalveolar lavage was performed, tuberculosis was excluded, but MSSA was confirmed (probable septic emboli). Blood cultures were taken from the CVL, and one was positive for vancomycin-resistant *Enterococcus faecium* (the new finding was interpreted as a nosocomial infection due to the patient’s poor adherence to the hygienic procedure involving the CVL). Linezolid was used for 2 weeks, and then oxacillin was resumed, completing a 6 week successful antibiotherapy.

**Conclusions**

An HIV positive patient, especially an IVDU, is characterized by a great susceptibility to infections, making him a challenge for the physician to diagnose and treat. Even with adequate therapy, these patients are prone to develop life-threatening complications, mainly because of poor adherence to treatment.

**Consent**

Written informed consent was obtained from the patient for publication of this Case report and any accompanying images. A copy of the written consent is available for review by the Editor of this journal.

### A78 Bacterial infection in critical patients with severe A H1N1 influenza virus infection (epidemiology, development, therapeutic decisions)

#### Doina Viorica Iovănescu^1^, Cleo Nicoleta Roșculeț^1^, Andrei Rogoz^1^, Cătălin Apostolescu^1,2^, Viorica Mitescu^1^, Tudor Vladoiu^1^, Dalila Toma^1^, Catrinel Ciuca^1^

##### ^1^National Institute for Infectious Diseases “Prof. Dr. Matei Balș”, Bucharest, Romania; ^2^Carol Davila University of Medicine and Pharmacy, Bucharest, Romania

###### **Correspondence:** Doina Viorica Iovănescu (liviuiovanescu@yahoo.com)

**Background**

Infection with influenza virus through the many types and subtypes but also by its airborne spread, remains a public health problem. Lack of specific therapies, safe, decreased immunity and host aggression by germs from the external environment, the possible source of super infection lead to the great severity of this condition. This is reflected in mortality rates: from 21 patients with H1N1 flu (confirmed PCR), 8 patients died (38.1 % death rate during 2015–2016 season).

**Methods**

Retrospective analysis of cases hospitalized in the period December 2015 - April 2016, confirmed with influenza AH1N1 virus, with severe respiratory failure (ARF). We highlight clinical data, biological, viral, and biological imaging specific to each patient.

**Results**

Bacterial reinfection with unique or mixed germs, type MDR, PDR, both cocci (MRSA pneumonia *E. faecium*) and Gram negative bacilli (*A. baumannii*, *K. pneumoniae*, *E. coli*) and fungi (*C. albicans, C.* non *albicans*: *glabrata, kefir*) detected in both carriage and pathological products or on the surface of catheters or probes, were the targets of antimicrobial therapy (AB latest dose and multiple association and supportive therapy ± antifungal therapy appropriate to the stage of sepsis + antiviral therapy - oseltamivir) initiated early and individualized casework. Advanced support of vital functions and physiopathogenic supportive therapy with intensive nursing formed the basis of therapeutic complex behavior. The death of the 8 patients was carefully analyzed from a bacteriological standpoint: 5 cases died of severe sepsis or septic complications caused by pneumococcus, *K. pneumoniae*, P C, *A. baumannii* + *A. fumigatus*, MRSA and 3 cases, admitted in extreme status of serious critical conditions, unstable condition, in septic shock and MSOF had unidentified etiology and death between 3–9 days after admission.

**Conclusions**

The data presented above show us great seriousness of respiratory infections with influenza with quick development of respiratory failure, requiring advanced support through IOT + VM method, imminent help, but increasing the risk of superinfection. Extremely severe bacterial infections with multiple resistance, create great difficulties in ensuring the success of bacteriological control. To optimize this success particular material resources are needed, to cover the acquisition of highest technology and the latest equipment, as well as human resources, highly qualified and experienced practice to be able to make quick decisions, accurate and responsible multidisciplinary working groups.

## Interdisciplinary studies in infectious diseases

### A79 Epidemiological aspects of severe acute respiratory infection cases (SARI) in the season 2015–2016, in the Clinical Hospital of Infectious Diseases – Constanța, Romania

#### Iulia Gabriela Șerban, Marioara Neacșu

##### ^1^Clinical Infectious Diseases Hospital of Constanța, Constanța, Romania

###### **Correspondence:** Iulia Gabriela Șerban (serbaniuliagabriela@gmail.com)

**Background**

Influenza is an acute infectious disease with increased epidemic potential. Flu and severe acute respiratory infections (SARI) are an important public health problem. Objectives: 1. Evaluation of types/subtypes of influenza in the 2015–2016 season, in patients from the Clinical Hospital of Infectious Diseases (SCBI), Constanța. 2. Epidemiological characterization of SARI hospitalized in SCBI Constanța, according to the criteria in the surveillance methodology for influenza, acute respiratory infections (ARI) severe acute respiratory infection (SARI), during the 2015–2016 season.

**Methods**

Retrospective analysis of laboratory-confirmed flu cases in the 2015–2016 season in SCBI Constanța. Retrospective analysis of SARI cases, according to epidemiological criteria – distribution by gender, age, area of origin, prior vaccination status, seasonal travel history, contact with a confirmed case of influenza, associated chronic diseases, signs of severity (clinical signs of pneumonia, complications such as acute respiratory failure or secondary bacterial infection, oxygen therapy, mechanical ventilation, antibiotic therapy).

**Results**

The total number of laboratory confirmed influenza cases was 359, of which 334 were Type A subtype H1N1, 13 type A, negative subtype H1N1, 8 type A, 1 type A subtype H3, 3 type B. Hospitalization was necessary for 74 patients with SARI (45 females and 29 males), belonging to different age groups (2–4 years – 1, 15–49 years – 21, 50–64 – 36, over 65 – 16), backgrounds of different origin (urban 52, rural 22) with comorbidities (cardiovascular 38, bronchopulmonary 12, diabetes 4 cases, other 44, pregnancy 3, obesity 37), with history of travel abroad (Bulgaria, Libya) – 2 cases, previous contact with a person with confirmed influenza – 1 person, and only 4 persons with a history of influenza vaccination. Sixty-six patients had clinical signs of pneumonia. There were medical complications like acute respiratory failure – 15 cases and secondary bacterial infection (pneumonia) – 56 cases. 68 patients received antibiotic therapy, 38 patients received oxygen. All survived. No patient was mechanically ventilated.

**Conclusions**

In the Clinical Hospital of Infectious Diseases, Constanța, Romania, SARI mortality was zero. The receptive population was unprotected by vaccination and mostly consisted of adults with comorbidities. AH1N1 was the most prevalent subtype in the flu in season 2015–2016.

### A80 Overexpression of IL-6 trans signaling pathway in viral infections

#### Simona Roxana Georgescu^1,2^, Vasile Benea^2^, Corina Daniela Ene^3^, Mircea Tampa^1,2^, Cristina Iulia Mitran^2^, Ilinca Nicolae^2^

##### ^1^Carol Davila University of Medicine and Pharmacy, Bucharest, Romania; ^2^Clinical Hospital of Infectious and Tropical Diseases “Dr. Victor Babeș”, Bucharest, Romania; ^3^Clinical Hospital of Nephrology, “Dr. Carol Davila”, Bucharest, Romania

###### **Correspondence:** Cristina Iulia Mitran (cristina.iulia.mitran@gmail.com)

**Background**

Interleukin 6 (IL-6) is involved in the pathogenesis of inflammation, infections, neoplasms and autoimmune diseases. Its biological activity is exerted through two mechanisms which involve the binding of IL-6 to a specific membrane receptor, abbreviated as IL-6R (classic signaling pathway) or the binding of IL-6 to the soluble form of the receptor, abbreviated as sIL-6R (trans signaling pathway); this step is followed by the interaction between the previously formed complex and the transducer subunit (gp 130). The investigation of the intracellular signaling pathways, by which IL-6 mediates pro- and anti-inflammatory effects in viral infections, represents the aim of our prospective study.

**Methods**

In our study we included 136 subjects divided into 4 groups: group A – 40 patients with viral infections (HIV, HCV, HBV), group B – 40 patients with cutaneous malignant melanoma, group C – 16 patients with malignant melanoma associated with viral infections and group D – 40 healthy subjects. The determination of serum levels of IL-6, sIL-6R and soluble glycoprotein (sgp) 130 was performed by ELISA technique.

**Results**

Serum levels of IL-6 were significantly higher in patients with viral infections (31.2 ± 7.3 pg/mL p < 0.05), malignant melanoma (19.3 ± 6.8 pg/mL, p < 0.05) and malignant melanoma associated with viral infections (37.9 ± 11.5 pg/mL, p < 0.05) than in the control group (2.11 ± 0.85 pg/mL). Compared to controls (74.23 ± 2.17 ng/mL), serum levels of sIL-6R were moderately elevated in patients with viral infections (114.1 ± 33.6 ng/mL, p <0.05), malignant melanoma (96.3 ± 31.4 ng/mL, p > 0.05), and malignant melanoma associated with viral infections (127.1 ± 43.1 ng/mL, p < 0.05). Significant variations were also obtained for sgp 130, as follows: 281.4 ± 19.4 ng/mL in the control group, 309.8 ± 22.4 ng/mL, p > 0.05 in patients with viral infections, 363.8 ± 43.8 ng/mL, p < 0.05 in patients with malignant melanoma, 331.3 ± 29.5 ng/mL, p = 0.05 in patients with malignant melanoma associated with viral infections.

**Conclusions**

IL-6 is secreted in response to viral infections and malignant transformation. The sIL-6R subunit is overexpressed in viral infections. Soluble glycoprotein 130 is synthesized in small amounts in the studied viral infections. Therefore IL-6 may be involved in the regulation of inflammatory responses of the human body through many mechanisms. In viral infections IL-6 trans signaling pathway is overexpressed. Knowledge of intracellular signaling mechanisms of IL-6 has important consequences on the therapeutic strategies which are aimed at blocking the activity of this cytokine.

### A81 Acute viral hepatitis B with persistent HBsAg – description and evolution

#### George Ciprian Pribac^1,2^, Mirandolina Prisca^1,2^, Fulvia Ursoiu^1,2^, Carmen Neamtu^1,2^, Bogdan Totolici^1,2^, Coralia Cotoraci^1,2^, Aurel Ardelean^1^

##### ^1^Vasile Goldiș Western University, Arad, Romania; ^2^Clinical Emergency County Hospital, Arad, Romania

###### **Correspondence:** George Ciprian Pribac (georgepribac@gmail.com)

**Background**

We have studied the evolution of clinical, humoral and pathological parameters in patients with persistent HBsAg for more than 3 months after the acute phase.

**Methods**

We followed in this study 50 patients hospitalized in our clinic within 5 years. Patients in the study were diagnosed with acute B type hepatitis in patients with positive HBsAg. Have observed that our patients with persistent HBsAg over 3 months in 19 patients presenting more frequently than unfavorable evolution towards healing.

**Results**

Subjectively patients described signs such as weakness, fatigue, effort pain. Objectively we have detected the presence of hepatomegaly and splenomegaly. After 2 years of persistence of HBsAg in 3 patients vascular stars were present, which were later shown as signs of chronic active hepatitis. We have followed the dynamics of viral markers, evolving pursued hepatodepressive syndrome, syndrome retention cytolysis and inflammatory dysproteinemia. Humoral changes present in HBsAg persistent hepatitis are generally encountered in the acute phase (but with different intensity), plus they develop or intensify the inflammatory syndrome. In cases with persistent HBsAg the dynamic development of humoral syndromes is categorically different compared to the evolution of hepatitis with the same syndromes in cases of acute infection evolving with HBsAg negativity.

**Conclusions**

The persistence of HBsAg after the acute phase was detected in 64.4 % of cases. In 15.6 % we observed evolution towards chronicity. Evolution toward healing (seroconversion) was seen in 20 % of cases, after 12 months after the acute phase.

### A82 Prevalence of cervical pathogens in a population of pregnant female patients monitored in a tertiary care hospital in Bucharest, Romania

#### Simona Elena Albu^1,2^, Mara Carsote^1^, Beatrice Miclăuș^1^, Diana Mihai^2^, Oana Săndulescu^1^, Cristina Vasiliu^1,2^

##### ^1^Carol Davila University of Medicine and Pharmacy, Bucharest, Romania; ^2^University Emergency Hospital of Bucharest, Bucharest, Romania

###### **Correspondence:** Oana Săndulescu (oanasandulescu1@gmail.com)

**Background**

Bacterial infection or colonization during pregnancy may be innocuous or may lead to significant consequences for the mother or the fetus. Early diagnosis and appropriate antimicrobial treatment are essential steps towards the best possible pregnancy outcome.

**Methods**

We have performed a retrospective study to assess the prevalence of potentially pathogenic bacteria and fungi identified through smears and cultures of vaginal and cervical discharge collected from pregnant patients attending one gynecology/obstetrics ward of a tertiary care hospital in Bucharest, Romania from July 2015 to July 2016. The statistical analysis included the Chi-square test for categorical variables and the Mann–Whitney non-parametric test for continuous variables, and was performed with IBM SPSS Statistics v.22 (Armonk, USA).

**Results**

We have assessed 120 pregnant female patients, with a median (interquartile range) age of 30 (26.3, 33) years and a median (interquartile range) duration of pregnancy of 30 (26.5, 33) weeks at the time the smear and culture tests were performed. Roughly half (58, 48.3 %) of the patients presented the normal Gram-positive tinctorial predominance on vaginal smears while 39 (32.5 %) presented apparently pathogenic Gram-negative flora, although only 2 of the vaginal discharge cultures (1.7 %) grew Gram-negative bacilli, namely *E. coli*. Another 17 (14.2 %) vaginal cultures were positive for yeasts, and all were negative for *S. aureus*, *Klebsiella* spp., and *Trichomonas vaginalis*.

Among cervical cultures, 11 (9.2 %) grew *E. coli* (including the two cases that also had positive vaginal cultures for *E. coli*), 2 (1.7 %) grew *Klebsiella pneumoniae*, 12 (10 %) grew yeasts, 1 (0.8 %) grew *Enterococcus* spp. and none were positive for *S. aureus* or *Trichomonas vaginalis*. Of the 11 identified *E. coli* strains, almost half (n = 5) were resistant to ampicillin, 2 were resistant to ciprofloxacin, and all were susceptible to cephalosporins, carbapenems, and co-trimoxazole. *Klebsiella* strains were positive for extended-spectrum beta-lactamases and were significantly more frequent at lower gestational ages (p = 0.023, Z = −2.152), while fungal growth from cervical cultures was significantly associated with younger maternal ages (p = 0.040, Z = −2.056).

**Conclusions**

We have identified fairly low prevalences of cervical pathogens in a population of pregnant female patients closely monitored in a tertiary care hospital in Bucharest, Romania. Our results suggest that screening for *Klebsiella* should be performed earlier in pregnancy and that younger women have higher incidences of fungal infections.

### A83 Prevalence of group B *Streptococcus* during pregnancy in a cohort of patients monitored in a tertiary care hospital in Bucharest, Romania

#### Cristina Vasiliu^1,2^, Mara Carsote^1^, Corina Gorgoi^2^, Beatrice Miclăuș^1^, Diana Mihai^2^, Oana Săndulescu^1^, Simona Elena Albu^1,2^

##### ^1^Carol Davila University of Medicine and Pharmacy, Bucharest, Romania; ^2^University Emergency Hospital of Bucharest, Bucharest, Romania

###### **Correspondence:** Oana Săndulescu (oanasandulescu1@gmail.com)

**Background**

Group B streptococci (GBS) are major causes of morbidity and long-term disability in babies born-to-mothers colonized or infected with this germ [1]. Neonatal GBS infection is prevented by intra-partum antibiotics, and all asymptomatic pregnant women should be screened for GBS through vaginal and intra-rectal cultures at 35–37 weeks of pregnancy [2].

**Methods**

We performed a retrospective study to ascertain the prevalence of GBS colonization/infection during July 2015-July 2016, in pregnant patients monitored in a tertiary care hospital in Bucharest, Romania.

**Results**

We studied 120 pregnant women with a median (IQR) gestational age of 30 (26.5, 33) weeks. The patient sample was representative for Southern Romania: 82 (68.3 %) from urban areas, 79 (65.8 %) from the Bucharest-Ilfov area, 32 (26.7 %) Southern, 5 (4.2 %) South-Eastern and 2 (1.7 %) from the South-Western area of Romania, while two patients were from Ukraine and Moldova. One-fourth of the patients (32, 26.7 %) had previous obstetrical medical history: uterine scarring (n = 24, 75 %), uterine fibroids (n = 2, 6.3 %), history of preeclampsia (n = 2, 6.3 %), and cervical scarring, septate uterus, history of placenta praevia, or history of intrauterine death (n = 1, 3.1 % each).

None of the vaginal cultures yielded GBS while 7 (5.8 %) of the cervical cultures grew GBS. The patients did not consent to intra-rectal cultures, therefore the real prevalence of GBS colonization may be somewhat underestimated.

The mean age ± SD of patients with positive GBS cultures was 30.03 ± 6.4 years; cultures were collected at a median gestational age of 38.5 ± 1.6 weeks. Urine cultures were performed in all but one case of GBS colonization and only one of them yielded asymptomatic GBS bacteriuria requiring treatment during pregnancy along with standard intra-partum antimicrobial prophylaxis for GBS colonization.

The prevalence of GBS colonization was slightly higher in patients from urban areas (6/82, 7.3 % vs. 1/36, 2.8 %) but the difference failed to reach statistical significance, probably due to the small number of overall positive cultures (p = 0.674, χ(1) = 0.924). The prevalence of GBS was not influenced by obstetrical history (p = 0.187, χ(1) = 2.703), patient age (p = 0.951, Z = 0.062), or gestational age (p = 0.620, Z = −0.496), and GBS colonization increased the odds of C-section 12-fold (OR: 12.242, CI95 %: 2.420–61.933, p = 0.005, χ(1) = 13.392).

**Conclusions**

We identified a fairly low prevalence of GBS colonization (5.8 %) and one case of urinary GBS infection during pregnancy. Our study did not reveal major risk factors for GBS colonization or infection in this cohort of pregnant women from Southern Romania.

**References**

1. Chukwu MO, Mavenyengwa RT, Monyama CM, et al. Antigenic distribution of *Streptococcus agalactiae* isolates from pregnant women at Garankuwa hospital - South Africa. Germs. 2015;5:125–133.

2. Puopolo K, Madoff L, Baker C. Group B streptococcal infection in pregnant women. In: Post T, ed. UpToDate. Waltham, MA: UpToDate; 2016.

### A84 Infectious hematoma in the gastrocnemius muscle – case presentation

#### Amelia Blescun, Gelu Breaza

##### “Dr. Teodor Andrei” Lugoj County Hospital, Lugoj, Romania

###### **Correspondence:** Amelia Blescun (amelia.blescun@gmail.com)

**Background**

In the absence of a clinically evident trauma it is difficult to diagnose a muscle hematoma which can be easily mistaken for a profound thrombophlebitis.

**Case report**

A 74 years old patient coming from the rural area, with a history of penile tumor with lymph nodes expansion (2008), operated on, diabetic and coronary patient, under treatment with Trombex for one year, is hospitalized accusing fever, dysuria, cloudy urine. Objective, without modifications on admission, paraclinically only a prostate hypertrophy is retained on ultrasound. Initially interpreted as urinary infection, therapy with ciprofloxacin is started after urine culture is collected. On the day of admission, the patient falls from his own height while going to the bathroom, without apparent trauma, without loss of consciousness. On the second day from admission, the patient accuses right lower back and right lower leg pain, without objective cutaneous modifications. The third day the pain centralizes on the right lower leg, with gradual evolution towards posterior edema, with positive Hoffman sign. The situation is interpreted as being deep vein thrombosis and, therefore, anticoagulant therapy is initiated. After three days of treatment the patient is afebrile, still presenting edema and pain in the calf, neutrophilic leukocytosis appears, inflammatory evidence increases, metabolic imbalance is present, urine culture is sterile. The treatment is changed to ceftriaxone. The ultrasound of the right lower leg section shows a fluid collection of 15/3.5 cm. Puncture is performed in the area of the collection but nothing externalizes. A vascular surgery consultation in Timișoara refutes the diagnosis of thrombophlebitis thus maintaining the suspicion of possibly infectious hematoma. The patient is transferred to the surgery ward where the suspicion is confirmed surgically and the collection is evacuated.

**Conclusions**

A calf muscle hematoma can be easily mistaken, clinically, for a profound thrombophlebitis in the absence of cutaneous modifications. For a correct diagnosis, extended investigation is needed since therapy is completely different in these two situations.

**Consent**

Written informed consent was obtained from the patient for publication of this Case report and any accompanying images. A copy of the written consent is available for review by the Editor of this journal.

### A85 Reflections towards the underexplored HTLV Romanian viral circulation - adult T‐cell leukemia/lymphomas, a case series

#### Sabina Vintila^1^, Felicia Mihai^2^, Meilin Omer^2^, Cornel Dragan^2^, Daniela Pitigoi^1,3^

##### ^1^National Institute for Infectious Diseases “Prof. Dr. Matei Balș”, Bucharest, Romania; ^2^Colentina Clinical Hospital, Bucharest, Romania; ^3^Carol Davila University of Medicine and Pharmacy, Bucharest, Romania

###### **Correspondence:** Sabina Vintila (sabina@vintila.eu)

**Background**

More than 15 million individuals are infected by the T-lymphotropic virus (HTLV) through the world, the first described human oncovirus. Etiologically linked to the adult T‐cell leukemia/lymphoma (ATLL), the spread of the virus is endemic in Japan, Africa, Central and South America but little is known about the infection rates in Romania, where testing of blood-donors in the only structured epidemiologic ongoing program regarding the HTLV infection. Starting from the direct mutagenic effects of the virus, the present paper wants to raise awareness towards this endemic problem we are facing.

**Methods**

Under high clinical suspicion, the ATLL diagnosis was established in the Hematology department of the Clinical Hospital Colentina – Bucharest, from January 2014 to March 2016, by analyzing the lymphocyte morphology, immunophenotype, histology of the tissues affected in the pure lymphoma forms. The HTLV-1/2-specific antibodies were detected using an enzyme-linked immunosorbent assay (ELISA) and were confirmed by real-time PCR assay. For the confirmed cases we prospectively recorded the age, sex, immune status, the form and the severity of the disease, the response to the first, and if applied, to the second line of chemotherapy, in some instances in association with antiretroviral therapy. The epidemiological link to a possible HTLV infection source was explored using a standard questionnaire.

**Results**

During the survey period the department worked on 16 cases of ATLL, with a mean surviving period of 14 months post-diagnosis. Hyper-CVAD, or CHOP- regimen based chemotherapy were implemented, in association with antiretroviral therapy in 5 (30 %) of the managed cases. Only 3 (18.5 %) patients were known as HTLV positive patients before the oncologic diagnostic, being followed in monitoring infectious diseases centers; in one case a vertical transmission was identified after the mother’s diagnosis as a ATLL; 9 (56 %) of the patients presented with a HBV or HCV co-infection and 4 (25 %) of the patients declared they travelled and practiced at-risk activities in HTLV endemic areas.

**Conclusions**

Despite major advances in understanding the pathogenesis of the disease, management of these patients remains a challenge for clinicians as they do not respond or achieve only transient responses. Easy and constant access to antiretroviral therapy is essential and stem‐cell transplantation should be considered but prevention programs, standardized nationally and integrated internationally, need to become a public health priority.

### A86 A febrile confusion syndrome with acute onset – case presentation

#### Mirela Ciucu^1^, Marius-Dan Ionescu^1^, Cristina Roskanovic^1^, Valentina Barbu^1^, Iulian Diaconescu^1,2^, Florentina Dumitrescu^1,2^, Irina Niculescu^1,2^

##### ^1^“Victor Babeș” Clinical Hospital of Infectious Diseases and Pneumology, Craiova, Romania; ^2^University of Medicine and Pharmacy, Craiova, Romania

###### **Correspondence:** Mirela Ciucu (ciucu_mire@yahoo.com)

**Background**

In clinical practice, sometimes we meet cases that prove to be a real professional challenge.

**Case report**

We present a male patient, 68 years old, retired, hospitalized between 03.05–08.06.2016 in the Adult Infectious Disease Clinic, Victor Babeș Hospital, Craiova, being transferred from the Intensive Care Unit of Clinical Regional Hospital, Craiova. The disease had an acute onset, a week before the presentation in the Infectious Disease Clinic, with psychomotor agitation, language disorders, and confusion, being admitted in the Neurology Clinic with the suspicion of a stroke, infirmed subsequently by the imagistic explorations. The presence of fever in a confused patient without a stroke determines the solicitation of an infectious disease specialist consult, who then performs a lumbar puncture. Following the lumbar puncture the diagnosis of meningoencephalitis with clear cerebrospinal fluid (CSF) was formulated, the patient is then transferred in the Intensive Care Unit for sustaining vital functions receiving also etiologic target treatment (ceftriaxone, acyclovir, DOT). Three days later, due to favorable evolution, the patient is transferred in the Infectious Disease Clinic. From the anamnestic data, relevant are: the contact with his tuberculosis infected parents, daily alcohol consumption (75 gr/day) former smoker. Physical examination at admission reveals average general state, no fever, aware, cooperating, balanced cardio-respiratory, stiff neck. Stage diagnosis: meningoencephalitis with clear CSF. Biological examination reveals: complete blood count (normal leukocytes number and balanced leukocyte formula), moderate biological inflammatory syndrome (ESR = 27/55 mm), slight hepatocytolysis syndrome (TGP = 133.8 U/L, TGO = 50.4 U/L), negative HIV serology, the lumbar puncture performed in dynamics highlights CSF with persistent albumin cytological dissociation (elements count = 30/cmm, Pandy +++), decrease of chlorides (684 mg%). Pneumophtiziology consult recommends continuation of DOT, ENT consult diagnoses oropharyngeal candidosis, the rest of the exploration being in normal limits. Positive diagnosis: Meningoencephalitis of probable bacillary etiology. Drug-induced toxic hepatitis. He has received treatment with acyclovir and ceftriaxone (up to 10 days), DOT (initially H300 R600 E1600 Z2000 7/7 with further shift because of the hepatocytolysis syndrome to H300R450E1600 7/7), Nistatin, corticosteroids (dexamethasone 1fx2/day), mannitol, hepatoprotective and symptomatic drugs. The evolution was slowly favorable, raising management issues because the hepatocytolysis syndrome.

**Conclusions**

This bacillary meningoencephalitis raised some diagnosis issues because of the atypical onset (acute), the presence of a rough meningeal syndrome and therapy problems linked to the appearance of the hepatocytolysis syndrome.

**Consent**

Written informed consent was obtained from the patient for publication of this Case report and any accompanying images. A copy of the written consent is available for review by the Editor of this journal.

### A87 Retrobulbar optic neuritis in a HIV-positive patient - case report

#### Mihaela Ionică^1^, Ramona-Alexandra Zamfir^1^, Alina Cozma^1^, Otilia Elisabeta Benea^1,2^

##### ^1^National Institute for Infectious Diseases “Prof. Dr. Matei Balș”, Bucharest, Romania; ^2^Carol Davila University of Medicine and Pharmacy, Bucharest, Romania

###### **Correspondence:** Mihaela Ionică (mihaela_ionica@yahoo.com)

**Background**

Ocular impairment in HIV-positive patients appears mostly in severe immunodeficiency and involves especially opportunistic germs. There are also cases where HIV virus itself can cause ocular manifestations.

**Case report**

We present the case of a 26 years old woman known with HIV infection, admitted to National Institute for Infectious Diseases “Prof. Dr. Matei Balș” for sudden visual acuity decrease associated with blurred vision and eye pain exacerbated by motions. At the time of admission CD4 = 5/cmm, HIV RNA = 1655124 copies/mL patient dropped-off antiretroviral therapy several years ago. Investigations have ruled out other infections (viral, bacterial, fungal, TBC), neoplasia and autoimmune diseases. An ophthalmic examination revealed normal ocular fundus and a right visual field defect. Cerebrospinal fluid examination obtained at lumbar puncture was cell free, with proteins, glucose, chlorides on normal ranges, negative China ink test, negative *Cryptococcus* antigen test, negative VDRL, undetectable PCR-BK, undetectable Polyomavirus JC DNA, HIV RNA = 218468 copies/mL, negative cultures for bacteria, fungi and BK. Brain MRI recommended by the neurologist revealed swollen optic nerve without brain demyelinating lesions. A favorable course was noticed with ful recovery of visual acuity under i.v. treatment with dexamethasone and resumption of antiretroviral therapy.

**Conclusions**

HIV induced optic nerve damage may occur in HIV-positive patients with severe immunodeficiency. HIV related retrobulbar optic neuritis can be demonstrated by imaging study (MRI) and the exclusion of other infectious and noninfectious causes. We highlight the favorable outcome with cortisone and antiretroviral therapy. The effective antiretroviral treatment, resulting in increasing CD4 and undetectable HIV RNA, significantly decreasing up to extinction the incidence of the ocular manifestations of HIV virus infection.

**Consent**

Written informed consent was obtained from the patient for publication of this Case report and any accompanying images. A copy of the written consent is available for review by the Editor of this journal.

### A88 A rare presentation of Q fever – case presentation

#### Alexandra-Sînziana Dumitru^1^, Daniela-Ioana Munteanu^1^, Violeta Niță^1^, Cristina Popescu^1,2^, Iulia Bodosca^1^, Angelica Tenita^1^, Viorica Ispas^1^, Victoria Aramă^1,2^

##### ^1^The National Institute for Infectious Diseases “Prof. Dr. Matei Balș”, Bucharest, Romania; ^2^Carol Davila University of Medicine and Pharmacy, Bucharest, Romania

###### **Correspondence:** Alexandra-Sînziana Dumitru (dumitru.sinziana@gmail.com)

**Background**

Q fever is a zoonosis caused by *Coxiella burnetii*. The clinical presentation is polymorphic and nonspecific, but most often characterized by: self-limited influenza-like illness, pneumonia or hepatitis. On the other hand, there are other clinical forms, which are less frequent like: cardiovascular, neurologic, obstetric manifestations and dermatologic (5–20 %) in the form of erythema nodosum or other nonspecific exanthemas, maculopapular rash or diffuse punctiform pruritic rash.

**Case report**

We report the case of a 53 year old Caucasian female, consumer of raw milk, who presented for clinical and diagnostic reevaluation of a persistent fever. Initially, the case was diagnosed as an acute acalculous cholecystitis (cholestatic syndrome associated with hepatic cytolysis and thick gallbladder wall at CT) and treated with ertapenem and ciprofloxacin, under which the patient became afebrile, but the fever reappeared as soon as she left the hospital. At the time of admission in our clinic, the case was considered as an angiocholitis and treated initially with ampicillin/sulbactam, but after 48 h we escalated to piperacillin/tazobactam and doxycycline and then to meropenem and doxycycline (9 days of meropenem and 5 days of doxycycline) because the patient presented relapsing fever under treatment (maximum 39 °C). At day 6 of hospitalization, the patient developed erythematous, indurated nodules, not sharply marginated, with 2–3 cm in diameter, located on the anterior lower legs. Investigations were extended for finding the etiology of this new manifestation, diagnosed after the histopathologic examination as erythema nodosum. We excluded: pulmonary tuberculosis, sarcoidosis, streptococcal, *Yersinia, Salmonella* and *Mycoplasma* infections, cholangiocarcinoma, genital cancer. The only positive finding was positive IgM antibodies for *Coxiella burnetii*. We reintroduced doxycycline, despite lack of response at the beginning of hospitalization and soon after that the fever remitted. Repeating the *Coxiella* serology we still found high antibodies titer and the favorable evolution of erythema nodosum under doxycycline pleaded for the diagnosis of Q fever. Moreover, it is important to mention that the patient forgot to take her treatment for 1 day, which determined the apparition of new nodules. The evolution of these new lesions was favorable, a compatible aspect of a therapeutic confirmation of the diagnosis, which confirmed once more the efficacy of our management.

**Conclusions**

The case is highlighting a rare clinical form of Q fever and its particularities of evolution with a slow response to the usual therapy. At the same time our case is illustrating the differential diagnosis of persistent fever and associated erythema nodosum.

**Consent**

Written informed consent was obtained from the patient for publication of this Case report and any accompanying images. A copy of the written consent is available for review by the Editor of this journal.

### A89 Tinea incognita – case presentation

#### Vasile Benea, Simona Roxana Georgescu, Mircea Tampa, Diana Oana Leahu, Cristina Maria Safta, Mihaela Anca Benea

##### Clinical Hospital of Infectious and Tropical Diseases “Dr. Victor Babeș”, Bucharest, Romania

###### **Correspondence:** Vasile Benea (beneav@yahoo.com)

**Background**

Tinea incognita is an atypical manifestation of dermatophytoses. Most often it is due to inappropriate treatment with corticosteroids (steroid-modified tinea), systemic or topical, prescribed for some pre-existing pathology or given mistakenly for the treatment of misdiagnosed tinea. The inflammation and scaly, typical clinical signs of dermatophytoses, are suppressed making the diagnosis difficult. The usual sites where this problem occurs are the face, groins, lower legs, and hands. Long-standing steroid modified cases may require prolonged oral antifungal therapy.

**Case report**

We present the case of a 74 year-old female patient who for several months had an erythemato-squamous eruption located on the face (distributed especially over the malar region) and on the dorsum of the hands. Because of the distribution of the eruption on photo-exposed areas, it was established clinical diagnosis of lupus erythematosus and began treatment with topical corticosteroids. After an initial improvement of clinical manifestations, rash reappeared and continued to expand gradually. The patient is referred to our clinic for the diagnosis and therapeutic management. In addition to clinical manifestations described above, dermatological examination revealed nail changes, including on the fingers. Usual laboratory investigations were within normal limits, and HIV serology was negative. Immunological investigations for lupus erythematosus were inconclusive and direct mycological examination of the lesions (after a week period of treatment’s cessation) was negative. A biopsy was carried out on one of the face’s lesions, and histopathological examination revealed fungal elements. It was initiated topical and systemic antifungal treatment, as the rash disappeared; after three months of treatment the nail changes remitted also.

**Consent**

Written informed consent was obtained from the patient for publication of this Case report and any accompanying images. A copy of the written consent is available for review by the Editor of this journal.

### A90 Incidence and risk factors associated with TORCH infections during pregnancy

#### Oana Săndulescu^1^, Octavian Munteanu^1,2^, Roxana Bohâlțea^1,2^, Livia Trașcă^2^, Monica Cîrstoiu^1,2^

##### ^1^Carol Davila University of Medicine and Pharmacy, Bucharest, Romania, ^2^University Emergency Hospital of Bucharest, Bucharest, Romania

###### **Correspondence:** Oana Săndulescu (oanasandulescu1@gmail.com)

**Background**

Infections during pregnancy may significantly impact fetal development, while causing minimal maternal morbidity. This is particularly true for a subset of pathogens such as *Toxoplasma*/others/rubella/cytomegalovirus (CMV)/herpesviruses (TORCH). Among these, the highest risk of congenital malformation is associated with early pregnancy acute rubella infection, but CMV and *Toxoplasma* also account for significant fetal developmental issues, while herpesviruses pose the highest threat to newborns, if infection is transmitted vertically.

**Methods**

We have studied the incidence of acute *Toxoplasma*, rubella and CMV infection (serum IgM screening) and the seroprevalence of serum IgG antibodies in pregnant females attending a reference center for obstetrics-gynecology in Romania. The study was implemented through the Norwegian Financial Mechanism and access to the screening program was available to pregnant females from all over Romania in any trimester of pregnancy. To date, 500 patients have been screened for TORCH in this project. The statistical analysis was performed with SPSS Statistics for Windows (v22.0, IBM Corp, USA), using Pearson Chi-squared test for multi-group comparisons and independent-samples t-test for normally-distributed continuous variables.

**Results**

We present the preliminary results for the first 200 patients screened, with a mean ± standard deviation age of 31.2 ± 4.9 years, most of them (81 %) from urban areas, with wide national coverage: 66.5 % Bucharest-Ilfov, 26 % South, 3.5 % South-West, 2.5 % South-East and 1.5 % North-East Romania. Screening was performed at a median (interquartile range-IQR) gestational age of 21.1 (11.8, 21.1) weeks. The highest seroprevalence was recorded for CMV (95 %, with a median [IQR] value of 435.2 [317, 500] AU/mL), followed by rubella (90 %, median [IQR] 88.2 [42.6, 161] IU/mL) and *Toxoplasma* (27.5 %, median [IQR] 46.9 [26.2, 107.7] IU/mL). Anti-*Toxoplasma* IgM (or IgM + IgG) were positive in 4.5 % (4 %) of patients, 1.5 % (1 %) for rubella and 4 % for CMV (all 4 % IgM + IgG). Patients with positive anti-CMV IgM had higher gestational ages at screening: 29.4 ± 9.7 vs. 21.6 ± 10.9 weeks (p = 0.048, t(193) = −1.988, high effect-size, d = 0.8). Patients with positive anti-*Toxoplasma* IgM were significantly more likely to cumulate pregnancy-associated adverse-behaviors (e.g., smoking, p = 0.008, χ(2) = 9.620), while those with anti-Rubella IgM were frequently ex-smokers (p = 0.004, χ(2) = 11.102). Rural dwelling was not a risk factor for acute infections.

**Conclusions**

We report high seroprevalence for major pregnancy pathogens, suggestive for protective immunity, but also posing risk of reactivation during pregnancy. The incidence of acute infections in pregnancy was fairly low, but patients with such acute infections during pregnancy also cumulated pregnancy-associated adverse-behaviors, requiring close monitoring and specialized counselling.

**Acknowledgement**

RO19.10 project: Initiatives in Public Health–Norwegian Financial Mechanism.

### A91 Acute respiratory failure in critical patients with sepsis

#### Doina Viorica Iovănescu^1^, Cleo Nicoleta Roșculeț^1^, Andrei Rogoz^1^, Cătălin Gabriel Apostolescu^1,2^, Viorica Daniela Mitescu^1^, Tudor Gheorghe Vladoiu^1^, Dalila Toma^1^, Catrinel Ciuca^1^

##### ^1^National Institute for Infectious Diseases “Prof. Dr. Matei Balș”, Bucharest, Romania; ^2^Carol Davila University of Medicine and Pharmacy, Bucharest, Romania

###### **Correspondence:** Doina Viorica Iovănescu (liviuiovanescu@yahoo.com)

**Background**

Acute respiratory infections are the most common cause of hospitalization in ICU in INBI Matei Balș so from 86 patients with severe sepsis admitted, 34 patients experienced respiratory dysfunction with ARDS, 19 patients experienced cardiac dysfunction and 17 patients showed dysfunction NSC. ARDS is defined as an acute inflammatory syndrome characterized with bilateral parenchymal lung infiltrates on chest x ray and PaO_2_/FiO_2_ ratio < 200, resulting from causes other than acute left ventricular dysfunction. Inflammatory lung lesions may be induced by different causes like: viruses, pathogenic germs, parasites, fungi, etc. The outcome of patients is depending on the comorbidities, age, and status of immunocompromised pattern and the stage of infection in the moment of admission in ICU.

**Methods**

Monitoring and analysis of clinical data, biological imaging, microbiological and evolution of gases in the blood, guides us on the effectiveness of therapy and management to follow.

**Results**

Monitoring data presented above have demonstrated that of the 34 patients with acute respiratory failure (ARF), 26 patients developed severe sepsis of bacterial etiology with ARDS with respiratory starting point (nosocomial pneumonia). Pathogens involved were put out in pathological products as: sputum - 1 case, tracheal aspiration - 8 cases, hemoculture- 5 cases; invasive devices: catheters, e.g.: IOT (5 cases), CVC (4 cases), urinary catheter (4 cases). Cocci pathogens accounted for: 12 cases (pneumococcus - 8, MRSA - 3, *Staphylococcus aureus* - 2, *Enterococcu* spp. - 2). Gram-negative bacilli: 13 cases (*A. baumanii* - 3, *Pseudomonas* - 5, *Klebsiela pneumoniae* - 5). Fungi: 3 cases (*C. krusei, C. albicans, C. glabrata*). Of the 26 developed nosocomial pneumonia 11 had unfavorable evolution, 42 % death rate. Median age of affected patients was 62.5 years. Antimicrobial therapy, according to microbiological evidence included associations of last generation antibiotics in individualized doses, ± antifungals ± antivirals (oseltamivir) in case of flu. ARF required specific protective measures using noninvasive ventilation or mechanical ventilation. ARDS in influenza was analyzed separately.

**Conclusions**

Great progress has been made in the management of ARDS. Nowadays novel agents and techniques are available, including antimicrobial drugs, extracorporeal membrane oxygenation, as well as supportive treatment (advanced mechanical ventilation). It seems that only the subgroup of patients with relative mild, early ARDS are likely to benefit from non-invasive ventilation.

### A92 Cochleo-vestibular deficit secondary to *Granulicatella elegans* meningitis

#### Mădălina Georgescu

##### Carol Davila University of Medicine and Pharmacy, Bucharest, Romania

**Background**

*Granulicatella* species are nutritionally variant streptococci. They are a normal component of the oral flora, but have been associated with a variety of invasive infections in humans, especially bacterial endocarditis or abdominal abscesses. Recently, *Granulicatella elegans* was isolated from the central nervous system (CNS) as well. CNS involvement has to be early recognized since *Granulicatella* infections have an increased morbidity and mortality as well as great bacteriologic failure and relapse rates.

**Case report**

We present a case of a 53 years-old woman with meningitis, first considered to be tuberculosis (TB). She received antiTB treatment, including streptomycin. Seven days after treatment initiation, the general condition improved, but the patient experienced bilateral deafness as well as severe disequilibrium, documented by pure tone audiometry and vestibular investigations (posturography, videonystagmography and video head impulse test – HIT). These permanent sensorial deficits have a huge negative impact on quality of life – patient cannot communicate anymore, walk alone or perform daily activities. Together with appropriate new antibiotic treatment for *Granulicatella* infection, hearing aid and vestibular rehabilitation program were recommended. Very few cases of *Granulicatella* meningitis are reported and none with hearing and equilibrium deficit secondary to this infection. We consider this case as a cochleo-vestibular bilateral deficit secondary to meningitis more than to ototoxic treatment, since in the majority of adult cases with ototoxic lesions, aminoglycosides affects only one of the sensorial structures from the inner ear – hearing sensory epithelium or vestibular one, but not both.

**Conclusions**

*Granulicatella* induce invasive infections. Rapid initiation of adequate antibiotic therapy minimizes the severity of the disease as well as long-term sequels. In this case, sudden severe bilateral onset of the hearing loss limited communication and permanent hearing loss will be partially compensated by the hearing aids. Regarding bilateral vestibular deficit, rehabilitation is very difficult and long-lasting, with dramatically limitations in daily activities.

**Consent**

Written informed consent was obtained from the patient for publication of this Case report and any accompanying images. A copy of the written consent is available for review by the Editor of this journal.

### A93 Influenza 2015/2016 – clinical, epidemiological and virological characteristics of cases admitted in three infectious diseases hospitals

#### Daniela Pițigoi^1,2,3^, Alina Elena Ivanciuc^3^, Mihaela Lazar^3^, Teodora Ionescu^4^, Carmen Maria Cherciu^3^, Cristina Țecu^3^, Maria Elena Mihai^3^, Maria Nițescu^1,2^, Rodica Bacruban^2^, Delia Azamfire^2^, Aura Dumitrescu^2^, Elena Ianosik^2^, Daniela Leca^5^, Elena Duca^6^, Andra Teodor^5^, Codrina Bejan^5^, Emanoil Ceaușu^1,7^, Simin-Aysel Florescu^1,7^, Corneliu Popescu^1,7^, Grațiela Târdei^7^, Codrina Juganariu^8^, Emilia Lupulescu^3^

##### ^1^Carol Davila University of Medicine and Pharmacy, Bucharest, Romania; ^2^National Institute for Infectious Diseases “Prof. Dr. Matei Balș”, Bucharest, Romania; ^3^National Institute for Research Cantacuzino, Bucharest, Romania; ^4^National Institute of Endocrinology “C. I. Parhon”, Bucharest, Romania; ^5^“Gr.T.Popa” University of Medicine and Pharmacy, Iași, Romania; ^6^Public Health Authority, Iași, Romania; ^7^Clinical Hospital of Infectious and Tropical Diseases “Dr. Victor Babeș”, Bucharest, Romania; ^8^Infectious Diseases Hospital “Sf Parascheva”, Iași, Romania

###### **Correspondence:** Alina Elena Ivanciuc (abaetel@cantacuzino.ro)

**Background**

Seasonal influenza is a public health problem, associated every year with an important number of hospitalizations especially in elderly population.

**Methods**

The aim of our study is to describe the clinical, epidemiological and virological characteristics of the influenza cases in people aged 65 years or older, admitted in three infectious diseases hospitals from Romania (two in Bucharest and one in Iași) in the season 2015–2016. The study was funded by I-MOVE+ project.

We screened patients admitted for signs and symptoms compatible with influenza infection in three infectious diseases hospitals, from week 53/2015 till week 20/2016. We collected information on demographics, vaccination and underlying conditions using a standardized questionnaire. No personal data were transmitted with questionnaires and patients gave a written informed consent to be swabbed. Cases were patients RT-PCR positive for influenza and controls those negative for any influenza virus.

**Results**

The influenza activity started in Romania in the week 4/2016, reached the peak in week 9 and lasted until week 19/2016 and was characterized by dominance of influenza A(H1N1)pdm09 viruses. We enrolled in our study 190 subjects; 168 met the influenza clinical case definition and the inclusion criteria and were analysed. Sixty-six patients (39.9 %) were influenza laboratory confirmed. Six of them (9.1 %) were vaccinated with seasonal influenza vaccine. Median age was 74.5 years and 51.5 % were female. The most frequent signs and symptoms presented at admission were: cough (98.5 %), fever (95.5 %), malaise (87.9 %), shortness of breath and myalgia (69.7 %). 90.9 % had at least one medical chronic condition. Heart diseases (77.3 %), diabetes (37.9 %) and lung diseases (21.2 %) were the most frequent. Seven patients (10.7 %) died, no one vaccinated. Majority of cases received antiviral treatment. 57 cases (86.4 %) were positive for A/H1N1, 7 (10.6 %) for influenza A/H3N2, 2 (3 %) for B. All strains AH1N1 pdm09 isolated were antigenically related to vaccine strain, A/California/7/09-like. The influenza viruses AH3N2 detected belonging to the genetic group 3C, subgroup 3C.2a and the type B detected belonging to B/Victoria lineage (B/Brisbane/60/2008).

**Conclusions**

People over 65 years old are at greater risk of complications from influenza, that might result in hospitalization or death. The most effective way to prevent influenza remains vaccination. Also, the antiviral treatment may reduce severe complications and deaths.

**Acknowledgement**

We acknowledge hospitals teams, patients, laboratory staff and EPICONCEPT team. The project has received funding from the European Union’s Horizon 2020 research and innovation programme under grant agreement No 634446.

## Other studies in infectious diseases

### A94 Severe complications of varicella requiring hospitalization in previously healthy children in Brașov county

#### Ligia Rodina^1^, Maria Elena Cocuz^2^

##### ^1^Infectious Diseases Hospital, Brașov, Romania; ^2^Faculty of Medicine, Transylvania University, Brașov, Romania

###### **Correspondence:** Ligia Rodina (ligiarodina@yahoo.com)

**Background**

Chickenpox is a common childhood infectious disease, very contagious, usually benign and self-limited, and complications are believed to be rare.

**Methods**

The purpose of this study is to describe the epidemiology of varicella complications in immunologically healthy children admitted to Infectious Disease Hospital of Brașov within the period January 2013 – July 2016, and to analyze the clinical and evolutive aspects of studied cases.

**Results**

Out of 178 cases hospitalized during this period with chickenpox diagnosis, 165 presented various complications. The majority of complications occurred in preschool age children, with an impressive number of cases (40 – 22.4 %) under one year of age. We found a gender predominance (56.1 % male, 43.9 % female), primarily within the urban population (74.1 %). The most frequent complication of varicella was bacterial superinfection of skin lesions (40.6 %) and pneumonia (36.3 %). Cellulitis occurred in 67 cases, 11 cases presented abscess with various locations (facial, extremities, trunk), requiring multidisciplinary treatment. *Streptococcus pyogenes* was the leading cause of bacterial infections (46.2 %), but also *Staphylococcus aureus* was found in a few cases. Other cutaneous complications include hemorrhagic varicella (3 cases) or purpura fulminans (1 case) which is associated with thrombocytopenia and disseminated intravascular coagulation. Invasive group A streptococcal disease (GAS) appeared in four cases, presenting positive blood culture, and multiorgan involvement in two cases, with favorable evolutions after treatment. Other complications were neurologic, which were reported in 15 children (6 %); meningitis was the leading diagnosis, followed by encephalitis and cerebellitis. One case of decease occurred at a child with purpura fulminans.

**Conclusions**

Bacterial infections complicating chickenpox cause significant morbidity and mortality, mainly in patients younger than 5 years of age. Varicella increases the risk for acquiring GAS disease. Cellulitis is the most common infection, deep-seated and septic infections can occur in immunologically healthy children with varicella. Chickenpox continues to cause significant morbidity in the pediatric population.

### A95 Clinical forms of *Clostridium difficile* colitis in children

#### Gheorghiță Jugulete^1,2^, Adina Stăncescu^2^, Cristina Elena Popescu^2^, Luminița Marin^1,2^, Diana Zaharia^2^, Cristina Dumitrescu^2^, Endis Osman^2^

##### ^1^Carol Davila University of Medicine and Pharmacy, Bucharest, Romania; ^2^National Institute for Infectious Diseases “Prof. Dr. Matei Balș”, Bucharest, Romania

###### **Correspondence:** Gheorghiță Jugulete (georgejugulete@yahoo.com)

**Background**

*Clostridium difficile* colitis is currently a public health issue because of the rising numbers of cases reported both in children and adults. This condition arises because of excessive antibiotic use but also because of the immunodepression caused by chemotherapy in oncological treatments. We propose to appraise the clinical forms of *Clostridium difficile* colitis in children as well as the evolution under treatment.

**Methods**

We have carried out a retrospective study on all cases of acute *C. difficile* infection in children admitted in the Pediatric Department of the National Institute of Infectious Diseases “Prof. Dr. Matei Balș” between 2013 and 2016. In all patients we have monitored age, sex, immunological status, clinical form of disease, and evolution under treatment. The diagnosis of colitis was established based on clinical criteria and confirmed through laboratory methods (detection of *C. difficile* A/B toxin from stool). All cases received treatment according to standard protocol except for one case where allergies to all antibiotics from the therapeutic schemes prompted us to perform fecal microbiota transplantation.

**Results**

In the aforementioned period we have registered 24 cases of *Clostridium difficile* colitis in children. The most affected categories were the 4–8 years age group (41.6 %), female patients (75 %), with 25 % of the patients being immunosuppressed. No deaths were registered, and 50 % of cases relapsed (3 cases - 1 relapse, 4 cases - 2 relapses, 2 cases - 3 relapses, 3 cases - more than 3 relapses). In cases with more than 3 relapses we have performed fecal microbiota transplantation. In one case with multiple antibiotic allergies, we performed fecal microbiota transplantation as the first choice of treatment without any previous antibiotic treatment. All fecal transplantation procedures were successful, without any incidents, and none of the cases registered any relapses.

**Conclusions**

*Clostridium difficile* colitis represents an important cause of morbidity in children, with high numbers of relapses and sometimes, with a severe life threatening evolution. Fecal microbiota transplantation represents an efficient alternative therapy to *Clostridium difficile* colitis which is unresponsive to standard treatment.

### A96 Community-acquired pneumonia – demographic, clinical and etiological aspects

#### Irina Niculescu^1,2^, Augustin Cupșa^1,2^, Iulian Diaconescu^1,2^, Florentina Dumitrescu^1,2^, Livia Dragonu^1,2^, Andreea Stoian^1^, Lucian Giubelan^1,2^, Cristina Roskanovic^2^

##### ^1^University of Medicine and Pharmacy Craiova, Craiova, Romania; ^2^“Victor Babeș” Clinical Hospital of Infectious Diseases and Pneumology, Craiova, Romania

###### **Correspondence:** Irina Niculescu (iri_nic@yahoo.com)

**Background**

Community-acquired pneumonia (CAP) is a common disease caused by a wide variety of pathogens and may be a professional challenge. In 1901, Sir William Osler noted in the fourth edition of his book, “The Principles and Practice of Medicine”, that pneumonia is the most important cause of morbidity and mortality of all acute diseases. More than a century later, the prominence of pneumonia did not change, the pneumonia being one of the most important causes of morbidity and mortality worldwide. Objectives: to establish the demographic and clinical aspects, evolution and etiology of CAP.

**Methods**

Retrospective study (July 2015-December 2015) on hospitalized patients with CAP in Adult Infectious Diseases Clinics of “Victor Babeș” Hospital Craiova; we analyzed demographic, clinical and laboratory data and the evolution of cases.

**Results**

We identified 238 patients with CAP, which constituted the study lot. General data of the study lot: gender distribution: 52 % male and 48 % female, 57 % (138) of patients were from urban areas, 43 % (100) patients were from rural areas with the predominance of elderly patients (50 % of cases aged over 60 years). The duration of hospitalization in the study group was significantly higher than the duration of hospitalization in patients with other infectious diseases (7.06 days vs. 6.11 days, p < 0.0001). Most cases of CAP (69 %) were typical pneumonia (by Laennec classification). Unfavorable outcome was recorded in 4 % of cases. Evaluation by criteria of severity (i.e., CURB-65 score and sepsis criteria) in the study group showed that most subjects met severity criteria, as follows: CURB-65 ≥ 3 points - 85 % of cases and sepsis defined by 2016 consensus criteria - 97 % of the cases. In most cases, patients in the study group, associated comorbidities, the most common comorbidity was chronic lung disease (37 % of cases). CAP etiology was identified in 15 % of cases. The etiology most frequently involved in CAP in the study group was represented by *Streptococcus pneumoniae*.

**Conclusions**

CAP patients were often elderly patients from urban areas. The duration of hospitalization in subjects with CAP was significantly higher vs subjects with other infectious diseases. The most common clinical presentation was a typical pneumonia, meeting criteria of severity (i.e., score CURB-65 ≥ 3 points and/or presence of sepsis criteria), in a patient associated comorbidities and having sometimes a fatal outcome. The most common identified etiology was *S. pneumoniae.*

### A97 Acute myocarditis in an adult patient with chickenpox - Case report

#### Ramona-Alexandra Zamfir^1^, Mihaela Ionica^1^, Otilia-Elisabeta Benea^1,2^

##### ^1^National Institute for Infectious Diseases “Prof. Dr. Matei Balș”, Bucharest, Romania; ^2^Carol Davila University of Medicine and Pharmacy, Bucharest, Romania

###### **Correspondence:** Ramona-Alexandra Zamfir (amzamfir@gmail.com)

**Background**

Varicella is an eruptive disease with mild clinical evolution. Adult patients can develop severe forms due to the complications that might occur. The cardiac complications caused by varicella zoster virus are rare, but with potentially high mortality rate, myocarditis being the most frequent of them.

**Case report**

We present the case of a 19 year old female patient diagnosed prior to admission to our clinic, with generalized skin blisters and rash, and fever (38 °C), chills, dyspnea at rest, precordial chest pain with irradiation to both shoulders. The patient had no personal and pathological antecedents or family history of cardiovascular disease. During clinical examination, the patient presented mild fever (37,3 °C), maculo-papular generalized rash, dyspnea at rest, 96 % SO_2_, normal breathing sounds, tachycardia (108 bpm), ventricular gallop, mitral systolic murmur of I^st^/II^nd^ degree. Laboratory tests: troponin I 1.1 ng/mL, positive IgM VZV serology. Echo, Coxsackie, CMV, Epstein Barr and *Toxoplasma* IgM were all negative. VDRL, HIV serology negative. Normal lung chest x-ray. ECG on admission showed sinus rhythm 100 bpm, negative T waves in precordial leads V3-V5, in DII, DIII, aVF. Echocardiography showed global hypokinesia, systolic dysfunction LVEF 45 %, mitral valve prolapse, moderate mitral regurgitation II^nd^ degree. The diagnosis of intra-infectious myocarditis was established according to: 1) minimum 2 elements objectivized at clinical heart exam (tachycardia, gallop rhythm, mitral systolic murmur of I^st^/II^nd^ degree), 2) etiological context (maculo-papular generalized rash, Ig M VZV positive), 3) abnormal enzymes values: troponin I 1.1 ng/mL, 4) ECG abnormalities (negative T waves DII, DIII, aVF, V3-V5 - changes that did not exist on previous ECG), 5) elements of myocardial dysfunction (echocardiography). After 5 days of treatment with acyclovir 4 grams/day, beta-blocker and bed rest the evolution was favorable with precordial chest pain and dyspnea remission and normal troponin I values. Echocardiography performed after 7 days showed an improved LVEF 66 %, with persistent moderate mitral regurgitation. ECG normalization was achieved in a few weeks.

**Conclusions**

Although varicella pneumonia is the most common cause of dyspnea, myocarditis can be a complication of chickenpox that determines shortness of breath. Early diagnosis and appropriate treatment of this rare form of myocarditis can lead to complete recovery of the patient.

**Consent**

Written informed consent was obtained from the patient for publication of this Case report and any accompanying images. A copy of the written consent is available for review by the Editor of this journal.

### A98 Caustic oropharyngeal wound with acute group F streptococcal superinfection mimicking diphtheria – case report and differential diagnosis

#### Maria-Cristina Sîrbu^1^, AnaMaria Dobrotă^1^, Alina Cristina Neguț^1,2^, Roxana Duda^1^, Rodica Bacruban^1^, Daniela Pițigoi^1,2^, Cristiana Cerasella Dragomirescu^2,3^, Daniela Tălăpan^1,2^, Olga Dorobăț^1^, Adrian Streinu-Cercel^1,2^, Anca Streinu-Cercel^1,2^

##### ^1^National Institute for Infectious Diseases “Prof. Dr. Matei Balș”, Bucharest, Romania; ^2^Carol Davila University of Medicine and Pharmacy, Bucharest, Romania; ^3^National Institute of Research “Cantacuzino”, Bucharest, Romania

###### **Correspondence:** Maria-Cristina Sîrbu (sirbu_cristina84@yahoo.com)

**Background**

Diphtheria has been mostly eliminated, as a result of routine childhood introduction of anti-diphtheria vaccination worldwide; in Romania the last case being identified in 1989.

**Case report**

A 34 year-old female with no medical history presented to an Otorhinolaryngology (ENT) Department in Bucharest for sore throat, odynophagia, mild fever, malaise and bilateral referred otalgia dating back 3 days, and recently installed shortness of breath. The clinical ENT exam and the laryngoscopy revealed: swollen palatine and lingual tonsils, covered by purulent exudates, with edema of the posterior pharyngeal wall, uvula, epiglottis and interarytenoid mucosa, as well as narrowed glottis cleft. No throat swabs were performed at that moment. She was diagnosed with probable epiglottic abscess and received treatment with ceftriaxone, gentamicin, metronidazole and corticotherapy. Given the absence of clinical improvement and the result of laryngoscopy: coalescing, adherent pseudo-membranes spreading from the tonsils to the pharyngeal area, the patient was referred to our clinic with the suspicion of diphtheria. At admission in our clinic, a detailed anamnesis revealed that she had developed shortness of breath 30 minutes after she had applied propolis tincture on the left tonsil. Epidemiological investigation identified that the patient was completely immunized during childhood and didn’t travel to endemic areas. The clinical exam showed afebrile patient with a satisfactory general state, without palpable lymph nodes, with balanced cardiovascular and respiratory functions. The lab reports showed leukocytosis with neutrophilia and acute inflammatory syndrome. Throat cultures were negative for *Corynebacterium diphtheriae*, but positive for group F *Streptococcus*. The levels of anti-diphtheria toxin IgG antibodies had protective value. In order to monitor the evolution of the lesions, daily ENT consult and flexible fiberoptic laryngoscopy were performed. Considering all this data, we interpreted the case as viral acute tonsillitis followed by caustic wound generated by the application of propolis tincture, superinfected with group F streptococcus. The patient received treatment with penicillin G and dexamethasone with full recovery after 9 days.

**Conclusions**

Although it is exceptionally rare now, diphtheria should not be excluded as differential diagnosis, especially given the latest Romanian epidemiological report that shows a decrease to 65 % in childhood vaccination rate and no immunization booster in adulthood. Natural therapy may at times have a harmful effect, such as the one described in the aforementioned case, or it can produce drug-drug interactions which can increase/decrease the efficacy of medical therapy.

**Acknowledgement**

Carol Davila University of Medicine and Pharmacy, Young Researchers Grant 28.335/04.11.2013

**Consent**

Written informed consent was obtained from the patient for publication of this Case report and any accompanying images. A copy of the written consent is available for review by the Editor of this journal.

### A99 *Clostridium difficile* infection in HIV-positive patients admitted in the National Institute for Infectious Diseases “Prof. Dr. Matei Balș” in 2015

#### Mihaela Ionica^1^, Ramona-Alexandra Zamfir^1^, Alina Cozma^1^, Otilia Elisabeta Benea^1,2^

##### ^1^National Institute for Infectious Diseases “Prof. Dr. Matei Balș”, Bucharest, Romania; ^2^Carol Davila University of Medicine and Pharmacy, Bucharest, Romania

###### **Correspondence:** Mihaela Ionica (mihaela_ionica@yahoo.com)

**Background**

Diarrhea commonly occurs among HIV-infected patients, with many possible causes, including infectious agents (especially opportunistic germs because of immunity loss), cancer or antiretroviral therapy by itself. Multiple prior hospital admissions, antibiotics use as treatment or prophylaxis increase the risk of *Clostridium difficile* infection (CDI) in these cases.

**Methods**

We retrospectively analyzed the HIV-infected patients with diarrhea (defined as at least 3 loose stools in 24 hours) admitted in the National Institute for Infectious Diseases “Prof. Dr. Matei Balș”, between 1 January 2015 and 31 December 2015. Incident cases were defined as first positive *C. difficile* toxin A and/or B using enzyme immunoassay technique, or PCR for toxin A/B gene in stool samples using GENEXPERT method. The relapses of CDI were excluded from our study.

**Results**

18 patients completed the inclusion criteria, representing a total of 24 % of the HIV-positive patients with diarrhea, and 75 % of the HIV-positive patients with infectious etiology of diarrhea, in which CDI was the most frequent agent. Male gender prevailed (61,19 %), with a mean age in our group of 40 years (ranging from 26 to 60 years) and an average hospitalization period of 27 days (ranging from 2 to 68 days). In 94.5 % of the cases, the diagnosis was established by PCR. 94.44 % of cases had previous hospitalizations within the last 3 months and 72 % had a recent antibiotic treatment, other than regimens used for opportunistic infection prophylaxis. As for the biological abnormalities, the mean WBC count was 6300/cmm (ranging from 1990 to 12690/cmm). The average value of CD4 cell count was 231 cells/cmm (ranging from 4 to 1044 cells/cmm). 61.11 % of the patients had a CD4 cell count under 200 cells/cmm. A sustained under-treatment viral suppression was noticed in 61.11 % cases from a total of 84 % of cases being under antiretroviral therapy. Remission of the symptoms was obtained by using oral treatment with vancomycin in 72.22 % of the patients, metronidazole in 22.22 % and intravenous treatment with tigecycline in 5.55 %. No case of fulminant CDI was reported and no death case directly attributable to CD infection. 5 patients of all (27.77 %) encountered relapses.

**Conclusions**

In our study, conformable to international medical data, *Clostridium difficile* proved to be the most frequent cause for infectious diarrhea in HIV-positive patients.

### A100 Title: Epidemiology of *Candida* oral infections (stomatitis) in Romania

#### Sergiu Fendrihan^1,2,3^, Ecaterina Scortan^4^, Mircea Ioan Popa^1,2^

##### ^1^Carol Davila University of Medicine and Pharmacy, Bucharest, Romania; ^2^“Cantacuzino” National Research Institute, Bucharest, Romania; ^3^Vasile Goldiș Western University, Arad, Romania; ^4^IMS Health Romania, Bucharest, Romania

###### **Correspondence:** Sergiu Fendrihan (ecologos23@yahoo.com)

**Background**

*Candida* superficial mycosis are very common fungal infection, one of the most frequent being *Candida* stomatitis. It is usually associated with immunocompromised status and with certain nutrition habits. A serious epidemiological evaluation for Romania was not published, yet.

**Methods**

The raw data were obtained from the Medical Statistic Direction of the Romanian Ministry of Health (2008–2012 data from the entire country). A statistical analysis based on usual methods (mean, standard deviation, Annova, Fisher tests, T Student, and other tests were performed in order to validate the results.

**Results**

We identified more that 3963 cases with an annual average of 793 cases. Most were registered in urban areas (2311 cases, 463/year), comparing with rural areas (1652 cases, 331/year) affecting mainly men (232 cases/year). Comparing with the other *Candida* infections the stomatitis represents an average of 46.6 %. The incidence of cases is higher at 0–4 years old group (51.2 %), mainly in male children between 0–1 years old. Over age 4 the percentage tends to reverse. The tests showed a significant correlation with age of the patients.

**Conclusions**

*Candida* stomatitis is more frequent in urban areas (463 cases/year), more than half being men (232/year) and little children. More studies are needed in order to evaluate the involvement of different *Candida* species in the infections. More laboratory confirmation data are needed and a very good collaboration between laboratory doctors, clinicians and epidemiologists is strictly necessary.

**Acknowledgement**

The study was made under the Sectorial Programme Human Resources Development (SOPHRD) financed by the European Social Fund and the Romanian Government under the contract number POSDRU 141531.

### A101 Anthrax case series in south-eastern Romania

#### Corneliu P Popescu^1,2^, Șerban N Benea^1,3^, Andra E Petcu^4,5^, Adriana Hristea^1,3^, Adrian Abagiu^3^, Iuliana A Podea^6^, Raluca E Jipa^1,3^, Georgeta Ducu^3^, Raluca M Hrișcă^3,7^, Dragoș Florea^1,3^, Manuela Nica^1,2^, Eliza Manea^3^, Simona Merișor^3^, Cristian M Nicolae^3^, Simin A Florescu^1,2^, Irina M Dumitru^4,5^, Emanoil Ceaușu^1,2^, Sorin Rugină^4,5^, Ruxandra V Moroti^1,3^

##### ^1^Carol Davila University of Medicine and Pharmacy, Bucharest, Romania; ^2^Clinical Hospital of Infectious and Tropical Diseases “Dr. Victor Babeș”, Bucharest, Romania; ^3^National Institute for Infectious Diseases “Prof. Dr. Matei Balș”, Bucharest, Romania; ^4^Faculty of Medicine, “Ovidius” University, Constanța, Romania; ^5^Clinical Infectious Diseases Hospital of Constanța, Constanța, Romania; ^6^Emergency County Hospital, Pitești, Romania; ^7^Central Universitary Emergency Military Hospital Dr Carol Davila, Bucharest, Romania

###### **Correspondence:** Ruxandra V Moroti (ruxandra_moroti@yahoo.com)

**Background**

Anthrax is considered a rare disease in the European countries. Twenty-one cases were reported (14 confirmed) in 2012, in Europe (last update by ecdc.europa.eu). In Romania, the National Institute of Public Health confirmed one case in 2013, 2 probable cases in 2014 (among 7 suspicions) and 2 confirmed cases in 2015 (among 6 suspicions): all cutaneous form, one meningo-encephalitis. All were related to animals and/or animal products exposure. The aim of our study is to describe the anthrax cases recently diagnosed in south-eastern Romania.

**Methods**

We reviewed the data of the patients admitted with anthrax suspicion, in 3 infectious diseases (ID) hospitals from south-eastern Romania: National Institute ID-Bucharest, Pitesti-County Hospital and Constanța-ID Hospital, between January 2013 and August 2016. Along with ECDC definitions of “confirmed” and “probable”, we used the category of “suspected” case from CDC* definitions. (*http://wwwn.cdc.gov/nndss/conditions/anthrax/case-definition/2010/).

**Results**

Among 9 suspicions, 4 were “confirmed”, 2 “probable” and 3 remained “suspected”. All had cutaneous lesions. In one, the main form was meningo-encephalitis. Median age was 43 year-old (34–67), male:female ratio 5:4. All live in rural areas and have been in contact with cows/goats/sheep. Case 1/2013: confirmed, 34 year-old man, butcher: black eschar and malignant edema on left upper limb; subsequently developed necrotizing fasciitis, superinfected with MDR Gram-negative bacilli, requiring adapted salvage antibiotic therapy and surgical decompression. Cases 2, 3&4/2014: suspected cases, females (39, 51, 57 year-old), painless red-vesicular lesion and black eschar on fingers, surrounding edema, after contact with similar lesions on animals. All had positive *B. anthracis* in-house serology. Case 5/2015: a 43 year-old shepherd with meningo-encephalitis: fever, psycho-motor agitation, hemiparesis, aphasia, neck-stiffness, CSF compatible with acute bacterial meningitis; black eschars on both hands. From CSF and cutaneous lesions, DNA-*Bacillus anthracis* was positive PCR-techniques performed in Victor Babeș Clinical Hospital, Bucharest, where the patient was transferred in ICU). Cases 6,7,8&9/July 2016: 2 confirmed, 2 probable, all shepherds with deceased animals, all cutaneous forms: a cluster of 3 (2 men and a woman: 67, 42 and 61 year-old) and a 34 year-old man. All patients had favorable evolution.

**Conclusions**

There is an important increase in anthrax cases in south-eastern Romania, all in animal-breeders, connected with an inappropriate veterinary surveillance. Not all cutaneous forms had a rapid good evolution, one developed potential fatal complications. The anthrax meningo-encephalitis described here had an unexpected favorable outcome, probable due to the young age, good previous health, early suspicion and prompt treatment.

**Consent**

Written informed consent was obtained from the patients for publication of this Case report and any accompanying images. A copy of the written consent is available for review by the Editor of this journal.

### A102 Knowledge, risk perception and attitudes of healthcare workers at the National Institute for Infectious Diseases “Prof. Dr. Matei Balș” regarding Ebola

#### Daniela Pițigoi^1,2^, Teodora Ionescu^3^, Oana Săndulescu^1,2^, Maria Nițescu^1,2^, Bogdan Nițescu^1^, Iulia Monica Mustaţă^4^, Sorina Claudia Boldeanu^4^, Florentina Furtunescu^1^, Adrian Streinu-Cercel^1,2^

##### ^1^Carol Davila University of Medicine and Pharmacy, Bucharest, Romania; ^2^National Institute for Infectious Diseases “Prof. Dr. Matei Balș”, Bucharest, Romania; ^3^Infection Control Department, National Institute of Endocrinology “C. I. Parhon”, Bucharest, Romania; ^4^Public Health Authority, Bucharest, Romania

###### **Correspondence:** Daniela Pițigoi (danielapitigoi@yahoo.co.uk)

**Background**

The outbreak of Ebola virus disease (EVD) in 2014 has exerted a great pressure on healthcare systems both inside, but also outside the African continent, in the attempt to identify and manage imported cases in order to prevent further transmission. In Romania, the National Institute for Infectious Diseases “Prof. Dr. Matei Balş” was the designated medical facility to manage possible cases of EVD. Our study aims to evaluate the knowledge, attitudes and risk perception of the Institute’s healthcare workers (HCWs) regarding Ebola virus disease and the recent epidemic.

**Methods**

We designed a cross-sectional survey based on a self-administered printed questionnaire comprised of 26 questions. In June 2016, all medical doctors and nurses were invited to take part voluntarily and anonymously in the study. Data were analyzed using Microsoft Excel (2013) and IBM SPSS Statistics (v. 22).

**Results**

Questionnaires were filled out by 157 HCWs. Physicians represent 36 % of all respondents. All participants are familiar with Ebola virus disease, but only 54 % of nurses had learned about it before the 2014 epidemic, as opposed to 75 % of medical doctors. The most frequent symptoms and main routes of Ebola virus transmission were correctly identified by the majority of respondents, whereas the possibility of airborne transmission was the most common misperception (55 %). During the epidemic, participants were slightly more worried for the health of their families, mean score 6.25/10, rather than for personal risk of contracting Ebola, 5.88/10 (p = 0.025). HCWs who had been involved in the medical care of Ebola suspect cases are two times more likely to volunteer to care for such patients in the future (RR = 2.475, 95 % CI 1.283–4.774, p = 0.039). Participants obtained most of the information on Ebola at their workplace (82 %); 92 % of HCWs attended at least one specific training and 64 % believe there are adequate personal protection methods available at the Institute. Donning/removing personal protective equipment was the most frequented course (85 %). Many participants think they were provided with sufficient information on EVD (66 %), but wanted to know more about preventive methods, treatment and the preparedness plan of Romania.

**Conclusions**

Most HCWs possess significant knowledge about Ebola virus disease. During the African epidemic, they experienced a moderate degree of worry and relied mostly on information provided at the Institute. Our study reveals the importance of professional education offered to employees by medical institutions and could help improve training sessions held during public health emergency situations.

### A103 A case of abdominopelvic actinomycosis with successful short-term antibiotic treatment

#### Diana Gabriela Iacob, Simona Alexandra Iacob, Mihaela Gheorghe

##### National Institute for Infectious Diseases “Prof. Dr. Matei Balș”, Bucharest, Romania

###### **Correspondence:** Diana Gabriela Iacob (dianagiacob@gmail.com)

**Background**

Extensive masses due to abdominopelvic actinomycosis raise challenging diagnostic and therapeutic problems due to frequent confusions with malignant tumors. First-line therapy includes high doses of penicillin over a prolonged period up to 6 months. Data concerning other antibiotics or shorter courses of therapy in actinomycosis are limited. We present a case of abdominopelvic actinomycosis with a complete remission after 42 days of penicillin combined with 21 days of tigecycline, in a 42 year-old woman hospitalized in “Matei Balș” National Institute for Infectious Diseases between 22.02-05.04.2016, who was later followed for 12 weeks after treatment.

**Case report** A 43 year-old woman with solitary right kidney and a 12-year history of an intrauterine device presented to the urology department of “Sf. Ioan” Clinical Emergency Hospital for a 2-month history of nausea, abdominal pain, 8 kg weight loss and dysuria. Imaging and laboratory investigations revealed severe anemia, obstructive renal failure, right ureterohydronephrosis and a large retroperitoneal mass of 10 cm × 9 cm × 10.4 cm involving the right ovary and uterus, invading the sigmoid, rectum, urinary bladder and compressing the right ureter. A double J stent and a urinary catheter were placed. The patient was consulted in the surgery ward for what was believed to be a metastatic ovarian carcinoma. Exploratory laparoscopy revealed dense but friable adhesions allowing only a left anexectomy with partial omentectomy and impeding a complete resection. Histological samples showed an inflammatory granulomatous tissue with multiple abscesses containing colonies of *Actinomyces* spp. and the patient was referred to the “Matei Balș” National Institute for Infectious Diseases. The patient was started on penicillin 12 million units/day for a total of 42 days. After the first 10 days of penicillin, tigecycline (100 mg/day) was added for 21 days for the treatment of concurrent urinary infection with MDR *Klebsiella pneumoniae* resistant to colistin and sensitive only to tigecycline and fosfomycin. The patient had a favorable clinical and biologic outcome. A CT assessment after 4 and 12 weeks revealed complete remission of the abdominopelvic mass with right ureterohydronephrosis and a few small abdominal adenopathies (<11 mm).

**Conclusions**

The case highlights the favorable effect of tigecycline for 21 days associated with the standard therapy of penicillin in a case of abdominopelvic actinomycosis with a remission at 6 weeks of therapy.

**Consent**

Written informed consent was obtained from the patient for publication of this Case report and any accompanying images. A copy of the written consent is available for review by the Editor of this journal.

### A104 A case of pneumonia caused by *Raoultella planticola*

#### Iulian Diaconescu^1,2^, Irina Niculescu^1,2^, Floretina Dumitrescu^1,2^, Lucian Giubelan^1,2^

##### ^1^University of Medicine and Pharmacy Craiova, Craiova, Romania; ^2^“Victor Babeș” Clinical Hospital of Infectious Diseases and Pneumology, Craiova, Romania

###### **Correspondence:** Iulian Diaconescu (diaconescu_ig@yahoo.com)

**Background**

*Raoultella planticola* is a gram-negative bacillus that was primarily considered as environmental bacteria, belonging to family Enterobacteriaceae and related to *Klebsiella* spp. *R. planticola* is sensitive to cephalosporins, aminoglycosides, fluoroquinolones; strains of *R. planticola* with resistance to almost all clinically available antibiotics except tigecycline and colistin have been reported. The reports of *R. planticola* infections are limited; this bacterium has been reported in cholecystitis, urinary tract infections, skin and soft tissue infections, pancreatitis and pneumonia.

**Case report**

We present a case of a 70 years old male from rural areas hospitalized in the Adult Infectious Diseases Clinics of “Victor Babeș” Hospital Craiova. The onset of illness was 7 days before admission with fever, chills and productive cough with purulent sputum. Medical history: hypertension and congestive heart failure class III NYHA. Admission exam: afebrile, right basal crackles at chest exam. Biological examination reveals: complete blood count - leukocytes number 7760 cells/mL with granulocytes percentage of 72.4 %; the rest of the biological exam in normal limits. Chest X-ray: right infrahilar internal alveolar opacity and cardiomegaly. Sputum culture reveals bacteria initially considered as *Klebsiella* spp. and further identified as *R. planticola* sensitive to amoxicillin/clavulanate, ceftriaxone, cefuroxime, trimethoprim/sulfamethoxazole, tetracycline, moxifloxacin and resistant to ampicillin. The patient received moxifloxacin with favorable evolution after 7 days of treatment.

**Conclusions**

*R. planticola* infections are recently reported in human pathology and there are very few published cases of pneumonia. The bacteria can be confused with *Klebsiella* spp. and this could be an explanation of the scarcity of cases recorded in literature. Our case is not a health care associated infection; the patient comes from rural areas. In general, sensitivity to common antibiotics does not raise special problems (as was the presented case), but there are clearly aggressive, multiresistant strains, that could problematize treatment in the future.

**Consent**

Written informed consent was obtained from the patient for publication of this Case report and any accompanying images. A copy of the written consent is available for review by the Editor of this journal.

### A105 Vitamin D deficiency and sepsis in childhood

#### Adriana Slavcovici^1^, Raluca Tripon^1^, Roxana Iubu^2^, Cristian Marcu^2^, Mihaela Sabou^1^, Monica Muntean^1^

##### ^1^Department of Infectious Diseases, Iuliu Hațieganu University of Medicine and Pharmacy, Cluj-Napoca, Romania; ^2^Teaching Hospital of Infectious Diseases, Cluj-Napoca, Romania

###### **Correspondence:** Adriana Slavcovici (aslavcovici@yahoo.com)

**Background**

Vitamin D, a secosteroid, has different immunomodulatory properties. Several recent studies have demonstrated the relationship between vitamin D deficiency and the severity of illness and mortality. The purpose of this study was to assess the vitamin D (25(OH)D) status in pediatric patients with sepsis and the association with severity score and biochemical characteristics.

**Methods**

The study was conducted at the pediatric department of Teaching Hospital of Infectious Diseases, Cluj-Napoca, between February and March 2016. We performed a prospective clinical observational study among children 1–18 years of age, admitted to our department. All consecutive patients with infection were screened for sepsis criteria at admission and were stratified in two groups with and without sepsis. Serum vitamin D measurement was performed in all patients within 2 days of admission and deficiency was defined as concentration of 25(OH)D <20 ng/mL. Electronic database included age, gender, diagnosis, identified microorganisms, Pediatric Risk of Mortality III score (PRISM-III), hematological and biochemical characteristics. Statistical analysis was performed by using GraphPad-Prism5. Mann–Whitney test was used to compare nonparametric variables. The relationship between vitamin D deficiency and clinical or biochemical variables was determined by relative risk and Spearman’s rank correlation.

**Results**

Twenty-eight children with sepsis and 21 without sepsis were enrolled. In all children enrolled, we found a negative correlation of 25(OH)D levels with PRISM-III score (R = −0.333, p = 0.01) and a positive correlation with iron, total and ionized calcium levels (R = 0.312, p = 0.03, R = 0.394, p = 0.007, respectively R = 0.515, p = 0.001). Patients with sepsis had a significantly lower median levels of 25(OH)D compared to cases without sepsis (21.35 ng/mL versus 34.8 ng/mL, p = 0.0004). The relative risk for sepsis was 2.22 (p = 0.03, specificity 0.79) and for PRISM-III >5 was 3 (p = 0.03, specificity 0.729) at vitamin D concentration <20 ng/mL. Compared to the group without sepsis, the sepsis group had significantly lower levels of HDL-cholesterol (22 mg/dL vs. 38.4 mg/dL, p = 0.015), albumine (3.6 g/dL vs. 4.08 g/dL, p = 0.005), total and ionized calcium (9.4 mg/dL vs. 9.8 mg/dL, p = 0.01, respectively 4.3 mg/dL vs. 4.8 mg/dL, p = 0.02).

**Conclusions**

In pediatric population vitamin D deficiency was associated with sepsis and higher PRISM-III score.

### A106 The clinical and epidemiological aspects and prophylaxis of Lyme disease among patients who presented with tick bites to the Clinical Infectious Disease Hospital “Toma Ciorbă”

#### Ion Chiriac^1^, Tiberiu Holban^2^, Liviu Tazlavanu^1^

##### ^1^Clinical Hospital of Infectious Diseases, Chișinău, Republic of Moldova; ^2^State University of Medicine and Pharmacy, “Nicolae Testemițanu”, Chișinău, Republic of Moldova

###### **Correspondence:** Ion Chiriac (ionchiriac@hotmail.com)

**Background**

There were seen 45 patients who presented to the Infectious Disease Hospital, “Toma Ciorbă” with complains of insects bites during May and June 2016. Thirty of them were bitten by *Ixodes* ticks.

**Methods**

We studied the clinical and epidemiological aspects and prophylaxis of Lyme disease among patients who presented with tick bites to the Clinical Infectious Disease Hospital “Toma Ciorbă”, Chișinău, Republic of Moldova.

**Results**

Among 30 patients with tick bites 21 were women with the average age of 40.8 years and 9 men, aged 58.1. 25 persons were from urban and 5 from rural areas. Seventeen patients (1^st^ group) did not show symptoms of Lyme borreliosis while 13 (2^nd^ group) were diagnosed with Lyme disease (LD). The mean span, while ticks were attached to the host was in average 1.7 days among persons in the first group while in second one - 2.5 days. The incubation period in LD patients varied from 7 to 32 days, and lasted in average 13 days. The diagnosis was established based on clinical presentations, erythema migrans (EM) and was confirmed by ELISA IgM and IgG anti *Borrelia burgdorferi* (Bb) antibodies. The most frequently revealed were complains on EM, pain and local burning sensations. Patients from the first group were given prophylactic treatment with doxycycline 200 mg as a single dose or amoxicillin in case of contraindications. None of them developed LD during a 45 days follow up period. Treatment for LD patients was successful in most of the cases; 4 patients were given doxycycline 100 mg twice daily (BD) for 14 days, 2 - for 10 days and 1 had doxycycline 100 mg BD for 21 days. Amoxicillin 500 mg orally TID for 14 days was prescribed to 3 patients and for 10 days in the same dosage to 2 patients. One person was given ceftriaxone 1 g BD for 21 days. Most of the patients had treatment as outpatients and just in 1 case the patient needed hospital treatment. During 45 days follow up period 1 patient was still complaining of anxiety, headaches and insomnia. Another one complained of general weakness. In one case skin lesions persisted despite of long lasting courses of antibiotics.

**Conclusions**

During a 60 days period LD was diagnosed in 13 patients who presented to the hospital with a history of tick bite. Fourteen patients were given doxycycline or amoxicillin as a prophylactic treatment which proved to be effective in all cases. Patients with LD were treated successfully with doxycycline or amoxicillin for a course lasting from 10 to 14 days as outpatients. In most of the acute LD cases outcomes were favorable.

### A107 Drug-resistant tuberculosis in HIV infected patients

#### Raluca Jipa^1^, Eliza Manea^1^, Roxana Cernat^2^, Kezdi Iringo^3^, Andrei Vâță^4^, Manuela Arbune^5^, Teodora Moisil^6^, Adriana Hristea^1^

##### ^1^National Institute for Infectious Diseases “Prof. Dr. Matei Balș”, Bucharest, Romania; ^2^Clinical Infectious Diseases Hospital of Constanța, Constanța, Romania; ^3^Clinical County Hospital Mureș, Tîrgu Mureș, Romania; ^4^Infectious Diseases Hospital “Sf Parascheva”, Iași, Romania; ^5^Infectious Diseases Hospital “Sfanta Paracheva”, Galați, Romania; ^6^Dr. Victor Babeş Clinical Hospital of Infectious Diseases and Pneumology, Timişoara, Romania

###### **Correspondence:** Raluca Jipa (ralucajipa@yahoo.com)

**Background**

Drug-resistant (DR) tuberculosis (TB) is a growing problem in HIV-infected patients, being associated with high rates of treatment failure and mortality. According to WHO, in 2014, 312 (2.1 %) patients diagnosed with TB were HIV-infected. The rate of multidrug resistant TB (MDR-TB) was 2.8 % (1.8–4.2) in the newly diagnosed TB cases and 11 % (8–15) in the retreatment group. Objective: Our aim was to describe clinical and laboratory characteristics of DR-TB in HIV infected patients.

**Methods**

We retrospectively analyzed HIV-infected patients diagnosed between January and December 2015 with TB in six tertiary care facilities in Romania. We included patients with diagnosis of DR-TB based on genotypic (MTB/RIF Xpert) and/or Löwenstein-Jensen culture plus drug susceptibility testing.

**Results**

We identified 150 HIV-infected patients with TB, of which 102 (68 %) had microbiological confirmation. Nineteen (19 %) patients had DR-TB: six (6 %) patients had resistance to isoniazid, 7 (7 %) to rifampicin and 6 (6 %) had MDR-TB. HIV diagnosis was established before TB diagnosis in 14 (74 %) patients with DR-TB, respectively 59 (58 %) patients with drug susceptible (DS) TB (p = 1, OR[95 % CI] 1.1[0.3–3.5]). The median age was 28 years both in the DR-TB group (IQR 26–40) and in the DS-TB group (IQR 26–37) (p = 0.648). Males represented 68 % in DR-TB group vs 67 % in DS-TB group (p = 1, OR[95 % CI] 0.9[0.3–2.7]). In the DR-TB group, 14 (74 %) patients had pulmonary TB, 2(10 %) extrapulmonary TB and 3 (16 %) patients had concomitant pulmonary and extrapulmonary TB. In the DS-TB group, 47 (57 %) patients had pulmonary TB, 15 (18 %) extrapulmonary TB and 21(25 %) patients had concomitant pulmonary and extrapulmonary TB. At TB diagnosis 9 (64 %) patients with DR-TB were on antiretroviral therapy vs 43 (52 %) patients with DS-TB (p = 0.8, OR[95 % CI] 0.8[0.3–2.2]). Seven (50 %) patients in the DR-TB group vs 33 (40 %) in the DS-TB group were on co-trimoxazole preventive therapy (p = 1, OR[95 % CI] 0.8[0.3–2.4]). Six (32 %) patients with DR-TB had previous treatment vs 10 (12 %) patients with DS-TB (p < 0.005, OR[95 % CI] 5.5[1.7–17.8]). The rate of MDR was 4.6 % in the newly diagnosed TB cases and 12.5 % in the retreatment group. In-hospital mortality rate was higher in patients with DR-TB (37 %) vs patients with DS-TB (14 %) (p = 0.016, OR[95 % CI] 3.6[1.2–10.5]). The median CD4 cell count in patients with DR-TB vs those with DS-TB was 18 (10–96) cells/cmm vs 84 (21–189) cells/cmm (p = 0.032).

**Conclusions**

In HIV-infected patients DR-TB caused was associated with previous treatment, lower median CD4 cell-count and increased in-hospital mortality. There were no statistically significant differences between the two groups regarding the TB site. The rate of MDR was greater than in HIV non-infected patients.

### A108 Kidney injury molecule-1 and urinary tract infections

#### Corina-Daniela Ene^1^, Ilinca Nicolae^2^, Roxana Simona Georgescu^2,3^

##### ^1^Carol Davila Clinical Hospital of Nephrology, Bucharest, Romania; ^2^Clinical Hospital of Infectious and Tropical Diseases “Dr. Victor Babeș”, Bucharest, Romania; ^3^Carol Davila University of Medicine and Pharmacy, Bucharest, Romania

###### **Correspondence:** Corina-Daniela Ene (koranik85@yahoo.com)

**Background**

Some urinary infections are considered to be triggers for interstitial nephropathies. The most frequent urinary infections are determined by bacteria, rarely by viruses or yeasts. The authors aimed to evaluate if kidney injury molecule-1 (KIM-1) could represent a specific serological marker in patients with urinary tract infections susceptible to develop kidney dysfunction.

**Methods**

The authors developed a prospective study on 55 patients with urinary tract infections caused by *Escherichia coli* and 30 control cases. All the subjects were adults, with normal serum lipids, normal weight and a balanced diet. Serum profile of KIM-1 (ELISA method) was analyzed by correlation with markers of renal dysfunction. The renal function was evaluated by serum urea, serum creatinine, estimated glomerular filtration rate (eGFR), albumin/creatinine ratio and uric acid/creatinine ratio.

**Results**

Compared to the control group (158 ± 39 pg/mL), the serum levels of KIM-1 were higher in patients with urinary infections (246 ± 82 pg/mL, p = 0.002). A negative correlation between KIM-1 levels and eGFR was observed in patients with urinary tract infections (r = −0.542, p = 0.001), and a positive association between KIM-1 and serum creatinine (r = 0.329, p = 0.015). No significant correlation between KIM-1 and other analyzed parameters were determined.

**Conclusions**

These results sustain the idea that KIM-1 could be a prediction factor for renal dysfunction in patients with urinary infections.

### A109 The impact of microbiological agents on serum gangliosides in patients with benign prostate hyperplasia

#### Corina-Daniela Ene^1^, Cosmin-Victor Ene^2,3^, Roxana Simona Georgescu^2,4^, Marilena Ciortea ^2^, Lucreția Dulgheru^5^, Ilinca Nicolae^4^

##### ^1^Carol Davila Clinical Hospital of Nephrology, Bucharest, Romania; ^2^Carol Davila University of Medicine and Pharmacy, Bucharest, Romania; ^3^Saint John Clinical Hospital of Emergency, Bucharest, Romania; ^4^“Victor Babeș” Clinical Hospital of Infectious Diseases and Pneumology, Craiova, Romania; ^5^Victor Babeș Center of Diagnosis and Treatment, Bucharest, Romania

###### **Correspondence:** Corina-Daniela Ene (koranik85@yahoo.com)

**Background**

Benign prostate hyperplasia (BPH) is a multifactorial pathology that affects life quality in elderly men. Recurrent urinary tract infections, frequently present in patients with obstructive benign prostate hyperplasia (BPH), could determine molecular disorders. The aim of the study was to investigate the effects of urinary tract infections on serum gangliosides.

**Methods**

The study included 60 subjects with benign prostate hyperplasia grouped, as follow: group A – 30 cases of benign prostate hyperplasia with urinary tract infections, group B - 30 cases of benign prostate hyperplasia without urinary tract infections. Gangliosides status by prostate specific antigen (PSA), C-reactive-protein (CRP), prostate volume and IPSS were determined. Serum levels of gangliosides were determined by spectrophotmoteric method using resorcinol- clorhidre, evaluated at 580 nm. PSA was determined by ELISA, CRP by immunoturbidimetry.

**Results**

Serum levels of gangliosides were significantly higher in patients with BPH and urinary tract infections compared with those without infection (28.4 ± 4.1 mg/dL in group A versus 22.7 ± 6.3 mg/dL in group B, p = 0.022). The statistical analysis showed the following correlations: between serum levels of gangliosides and PSA: r = 0.339, p = 0.053 in group A, r = 0.128, p = 0.042 in group B; between serum gangliosides and CRP: r = 0.301, p = 0.027 in group A, r = 0.326, p = 0.054 in group B, between serum gangliosides and IPSS: r = 0.367, p = 0.047 in group A, r = 0.209, p = 0.058 in group B. No relation between serum gangliosides and prostatic volume was observed.

**Conclusions**

Increased levels of serum gangliosides in patients with BPH could be considered a defense mechanism against infections, important in maintaining a vital biological balance. Serum gangliosides could represent a useful monitoring parameter in patients with obstructive prostate benign hyperplasia and urinary infections.

### A110 Toxocariasis - the experience of the Iași Infectious Diseases Hospital between 2013–2015

#### Mihaela Cătălina Luca^1,2^, Ioana-Alina Harja-Alexa^2^, Roxana Nemescu^1,2^, Mădălina Popazu^2^, Andrei Ștefan Luca^1^

##### ^1^“Gr.T.Popa” University of Medicine and Pharmacy, Iași, Romania; ^2^Infectious Diseases Hospital “Sf Parascheva”, Iași, Romania

###### **Correspondence:** Mihaela Cătălina Luca (Catalina_luca2006@yahoo.com)

**Background**

Toxocariasis is caused by a series of related nematode species that routinely infect dogs and cats throughout the world. This study is a warning on geographical regions underestimated. In the region of Moldova (Iași) toxocariasis is frequent in dogs, cats and foxes.

**Methods**

In the studied period (2013–2015), 180 cases of toxocariasis were admitted to the Iași Infectious Diseases Hospital.

**Results**

Toxocariasis prevalence in our geographic region is 51.7 %, with no significant gender differences. We can say that the majority of cases (66.5 %) were in the age group of 30–39 years and 40–49 years, while the minimum number of cases was found in the age group 60–69 years – (4 cases). The gender distribution of cases shows that 91 % of cases were women, 122 cases (67.77 %) being owners of pets (dogs). General clinical manifestations were represented by latch edema (44.44 %), headache (43.33 %), paresthesia (36.66 %), sweating (35.55 %), hoarseness (27.77 %), exanthemas (21.11 %) and abdominal pains (11.11 %). Locating ocular larvae of *T. canis* occurred more often in older children (10–16 years) and in adults. In the studied cases we met decreased visual acuity in 9 cases, 6 cases of uveitis, 2 cases with retinal granuloma, and 4 cases of endophthalmitis, all cases being hospitalized in Ophthalmology Clinics for appropriate therapy. Biochemical tests useful for diagnosis were represented by: major hypereosiniphilia, hyperleukocytosis, increasing ESR and gammaglobulin. In our study, leukocytosis was found in 62 % of cases and hypereosiniphilia in 88 %. For the certain diagnosis of toxocariasis we used serological methods which consisted in determination of specific *Toxocara* antibodies. *Toxocara* antibodies were present in all studied patients. The result was positive in 96 (69.56 %) cases and negative in 42 (30.43 %) cases. An IgG antibody titer of 1/100 was found in 40.5 % cases. The described complications and sequelae were met in 42 cases: respiratory disorders, ocular larva migrans, splenomegaly, uveitis, chorioretinitis and hepatomegaly.

**Conclusions**

Toxocariasis is characterized by expression of polymorphic clinical manifestations, from asymptomatic to various clinical forms with severe expression. What is needed in terms of future control programs is the radically development of new approaches, such as the effectiveness of molecular vaccines or based on DNA, offering the possibility of lifelong protection.

### A111 Species of anaerobic Gram-positive cocci involved in odontogenic abscesses

#### Gabriela Bancescu, Bogdan Dabu, Adrian Bancescu

##### Carol Davila University of Medicine and Pharmacy, Bucharest, Romania

###### **Correspondence:** Gabriela Bancescu (gabi.bancescu@gmail.com)

**Background**

The most pyogenic infections in the oro-maxillo-facial region are of odontogenic origin and are mixed infections, involving aerobic and anaerobic bacteria. The microbiological investigation is not commonly performed in such infections. The aim of the present study was to identify at species level a collection of 31 isolates of anaerobic Gram-positive cocci, stored in ultra-freezer at the laboratory of the Microbiology Department, Faculty of Dentistry, University of Medicine and Pharmacy (U.M.F.) “Carol Davila” - Bucharest. The strains had been previously isolated from pus samples collected by needle aspiration from 31 patients among those treated for different types of odontogenic abscesses (vestibular abscess, submandibular abscess, etc.) at the Department of Oro-Maxillo-Facial Surgery, U.M.F. “Carol Davila” - Bucharest, during March 2012 - March 2014.

**Methods**

The isolates of anaerobic Gram-positive cocci were identified at species level using the Rapid ID 32 A system (BioMérieux, Marcy-l’Etoile, France).

**Results**

All isolates except one belonged to *Parvimonas micra* (formerly *Peptostreptococcus micros* and *Micromonas micros*). The remaining strain was identified as *Finegoldia magna* (formerly *Peptostreptococcus magnus*).

**Conclusions**

*Parvimonas micra* is by far the predominant species of anaerobic Gram-positive cocci involved in odontogenic abscesses. Species identification must not be neglected in oro-maxillo-facial infections due to its contribution to a better understanding of the etiopathogenesis of such infections.

**Acknowledgement**

The present study was part of the internal research plan for 2015–2016 of the Department of Microbiology, Faculty of Dentistry, in collaboration with the head of the Department of Epidemiology, Faculty of Medicine, U.M.F. “Carol Davila” - Bucharest.

### A112 *Clostridium difficile* infection recurrences

#### Eliza Manea^1^, Raluca Jipa^1,2^, Adriana Hristea^1,2^

##### ^1^National Institute for Infectious Diseases “Prof. Dr. Matei Balș”, Bucharest, Romania; ^2^Carol Davila University of Medicine and Pharmacy, Bucharest, Romania

###### **Correspondence:** Eliza Manea (elizamanea10@yahoo.com)

**Background**

One of the most challenging aspects of *Clostridium difficile* infection (CDI) is its propensity to recur. First-line therapies were associated with high rates of recurrences. Objective: To assess the factors associated with recurrences in patients hospitalized with CDI.

**Methods**

Retrospective cohort study of adults admitted between September 2014 and August 2015 for CDI. We included patients diagnosed on clinical symptoms and detection of CD toxin in stool using polymerase chain reaction, which were treated with metronidazole, vancomycin and vancomycin plus at least 7 days of tigecycline. We excluded episodes in whom data to calculate Atlas severity score were not available. Recurrence was defined as another episode of diarrhoea, with positive toxin test within 8 weeks after the onset of the previous episode.

**Results**

From a total of 660 episodes of CDI we included 437 episodes (in 359 patients), of which 40 (9 %) were treated with metronidazole, 328 (75 %) with vancomycin and 69 (16 %) with tigecycline associated with vancomycin. Eighty-eight (20 %) episodes were CDI recurrences after the included episode. Forty-four (50 %) episodes from the recurrences group versus 156 (45 %) episodes from the group without recurrences were men (p = 0.22). The median age was similar for both groups: 71 years for patients with recurrences versus 70 years for patients without recurrences. There was no difference regarding the median Charlson score (4 for both groups) and the median Atlas severity score (3 for both groups). A neoplastic disease was diagnosed in 14 (16 %) CDI recurrence episodes versus 49 (14 %) CDI non-recurrence episodes (p = 0.423). The CDI recurrence episodes included in our analysis were treated with metronidazole in 9 (10 %) cases, vancomycin in 68 (77 %) cases and vancomycin plus tigecycline in 11(13 %) cases (p = 0.617). Thirty-three (38 %) episodes with recurrence as outcome of the included episode were preceded by recurrences versus 108 (31 %) episodes without recurrences after the current episode (p = 0.148). We did not find any significant association between CDI recurrences and age over 70 years (p = 0.283), albumin value less than 3 g/dL (p = 0.201), leukocytes number greater than 15000/cmm (p = 0.552), presence of acute renal insufficiency (p = 0.236) or concomitant systemic antibiotic therapy (p = 0.371).

**Conclusions**

CDI recurrences were not correlated with older age, presence of a neoplastic disease, administration of concomitant systemic antimicrobial therapy, history of another CDI recurrence, severity of CDI, expressed by lower albumin, high number of leukocytes or presence of acute renal insufficiency. Associating tigecycline did not reduce the recurrences compared to conventional therapy (metronidazole or vancomycin).

### A113. Differential diagnosis of staphylococcal and tuberculous osteodiscitis – case report

#### Adina Elena Ilie^1^, Săftica-Mariana Pohrib^1^, Alina Cristina Neguț^1,2^, Maria-Sabina Tache^1^, Maria Magdalena Moțoi^1^, Oana Săndulescu^1,2^, Ion Aurel Iliescu^3^, Adrian Streinu-Cercel^1,2^

##### ^1^National Institute for Infectious Diseases “Prof. Dr. Matei Balș”, Bucharest, Romania; ^2^Carol Davila University of Medicine and Pharmacy, Bucharest, Romania; ^3^University Emergency Hospital of Bucharest, Bucharest, Romania

###### **Correspondence:** Săftica-Mariana Pohrib (pohrib.mariana@yahoo.com)

**Background**

With the incidence of tuberculous osteodiscitis on the rise, *Mycobacterium tuberculosis* is currently considered the second etiological agent of osteodiscitis.

**Case report**

A 65 year-old female presented to our clinic for low-grade fever with intense back pain that progressed to the medial side of both lower limbs, with paresthesia in the left leg and pain in the right iliac fossa dating back 14 days. Medical history revealed stage 2 hypertension, right sylvian hemorrhagic stroke (2010) with left sequel hemiparesis and bilateral pulmonary thromboembolism (2010). The clinical exam showed left sequel hemiparesis, slightly diminished deep tendon reflexes on the left side and intensely impaired mobility. The lab reports showed acute inflammatory syndrome, coagulation disorders and elevated serum creatinine. A lumbosacral spine MRI scan conducted one day prior to admission described L4-L5 spondylodiscitis with massive anterior epidural abscesses and right paravertebral abscess. It was initially labeled as probably staphylococcal osteodiscitis and therapy was initiated with rifampin and levofloxacin with no clinical improvement and persistence of low-grade fever after 5 days, when rifampin was changed to linezolid. The evolution was apparently favorable (afebrile with decrease in biological inflammation markers), but the MRI conducted after one month of treatment revealed lesion progression. She was transferred to neurosurgery where a L4-L5 laminectomy was performed and during surgery a gray-yellow mass suggestive for *Mycobacterium tuberculosis* (TB) etiology was found. Wound and blood cultures were negative, Gram stain showed no bacteria and Ziehl-Neelsen stain failed to show acid-fast bacilli. Still, the histopathologic examination was highly suggestive for *Mycobacterium tuberculosis* etiology and anti-TB therapy was initiated with the 4-drug-regimen: isoniazid, rifampin, pyrazinamide, ethambutol, with clinical and biological improvement. So far, the patient has undergone anti-TB therapy for 3 months with neurological improvement and a repeat MRI scan is scheduled when the patient completes 5 months of therapy.

**Conclusions**

In the presented case, the clinical and radiological findings were insufficient to allow the differentiation between tuberculous and staphylococcal etiology. Additionally, the activity of anti-staphylococcal agents such as rifampin and linezolid on *Mycobacterium tuberculosis* masked the clinical progression of the disease and further prolonged the time to etiological diagnosis. Bacteriological tests failed to identify the etiologic agent, and the definite diagnosis was eventually based on histopathologic examination, highlighting the importance of obtaining a tissue specimen to allow accurate diagnosis and correct interdisciplinary management.

**Consent**

Written informed consent was obtained from the patient for publication of this Case report and any accompanying images. A copy of the written consent is available for review by the Editor of this journal.

### A114. Severe clinical forms of respiratory syncytial virus infections

#### Cristina Tecu^1^, Maria-Elena Mihai^1^, Mihaela Lazăr^1^, Carmen Cherciu^1^, Alina Ivanciuc^1^, Daniela Pițigoi^1,2^, Emilia Lupulescu^1^

##### ^1^Cantacuzino National Institute of Research, Bucharest, Romania; ^2^Carol Davila University of Medicine and Pharmacy, Bucharest, Romania

###### **Correspondence:** Cristina Tecu (ctecu@cantacuzino.ro)

**Background**

Respiratory syncytial virus (RSV) is an important pathogen that causes respiratory infections often severe and even death especially in children younger than 6 months. There are certain population groups that may be vulnerable to severe RSV infections, as pregnant women, immunocompromised patients and persons with chronic diseases. Usually the clinical aspect does not differ from those determined by other respiratory viruses and RSV often causes co-infections with other respiratory viruses.

**Methods**

We examined nasopharyngeal swabs taken from 646 patients diagnosed with SARI (severe acute respiratory infection) and admitted to 20 hospitals from six counties and Bucharest. Laboratory techniques: (1) real-time RT-PCR (reverse transcription-polymerase chain reaction) for the detection of influenza viruses type A and type B; (2) multiplex RT-PCR commercial kit RT-PCR One-Step ACE Seeplex (Seegene) for the detection of other respiratory viruses, and (3) sequencing of a fragment of 212 base pairs of the gene *SH* RSV B for identification and confirmation of RSV.

**Results**

Of the 646 samples, 339 were positive: 309 for Influenza Virus-IV and 30 for other respiratory viruses; 19 RSV, 1 hMPV (human MetaPneumoVirus), 2 Coronavirus 229E, 1 Adenovirus, 2 Rhinovirus, 5 Bocavirus. Three of the patients positive for RSV died (one death occurred in a woman of 82 years, one in a woman aged 59 years and one in a child 5 months). The three deceased patients had associated diseases. We mentioned that in February 2015 we reported a death from a child aged 2.9 years with type B of RSV disease and who didn’t present associated diseases. Lung samples were sequenced from this patient to confirm the presumptive diagnosis of RSV infection caused by type B. Sequencing showed 100 % identity between the two pieces of lung and 97–98 % identity with sequences from GenBank.

**Conclusions**

Although type B of RSV didn’t give severe clinical forms, in the case of the child aged 2.9 years, RSV type B induced a severe clinical form which resulted in death. In patients with associated chronic diseases, RSV can cause either death or serious infections. Infections like flu and infections due to RSV can spread easily in the community. Pathogenicity mechanisms for understanding this infection are needed along with more studies for genotyping and gene sequencing of RSV.

### A115 *Acinetobacter baumannii* postoperative sepsis associated with *Clostridium difficile* enterocolitis in an immune suppressed elderly patient

#### Mirela Paliu, Manuela Curescu, Bianca Cerbu, Iosif Marincu

##### Dr. Victor Babeş Clinical Hospital of Infectious Diseases and Pneumology, Timişoara, Romania

###### **Correspondence:** Mirela Paliu (madutzamada87@yahoo.com)

**Background**

Postoperative sepsis occurring in immunocompromised elderly patients, imposes complex clinical-therapeutic monitoring, and is known as an important cause of mortality in this population. The purpose of the paper is to present a clinical case with *Acinetobacter baumannii* sepsis associated with *Clostridium difficile* enterocolitis in an elderly immune suppressed patient.

**Case report**

The authors present the case of a patient of 83 years from urban environment with *Acinetobacter baumanii* sepsis associated with *C. difficile* enterocolitis. The patient was discharged from the Vascular Surgery Clinic, where he had undergone a right femoral-popliteal bypass. He is transferred to the Clinic of Nephrology with massive edema. After a day from hospitalization in the Clinic of Nephrology, he presents multiple diarrheal stool with positive Toxin A/B of *C. difficile* and he is sent to the Clinic of Infectious Diseases for specialized treatment. After five days of hospitalization, he presents a febrile episode (T = 38 °C), associated with chills and sweating. The blood culture which is harvested after 24 hours, was positive for *Acinetobacter baumannii* and other blood tests results include: RBC = 2.09*10^6^/μL, WBC = 6.86*10^3^/μL, PLT = 86*10^3^/μL, NEUT = 87.5 %, LYMPH = 8 %, MONO = 3.4 %, EO = 0.7 %, BASO = 0.4 %, HGB = 6.8 g/dL, HCT = 23.9 %, ESR = 20 mm/1 h, fibrinogen = 1.37 g/L, serum uric acid = 2.43 mg/dL, AF = 23.7 U/L, serum C-reactive protein = 59.38 mg/L, chol = 45 mg/dL, trigly = 22 mg/dL, procalcitonin = 3.75 ng/mL. During hospitalization he received therapy with metronidazole (3x500 mg/day orally), vancomycin (4x250 mg/day orally for *C. difficile* enterocolitis) and colistin (3x1 MUI/day), amikacin (4x250 mg/day), Sinerdol (3x300 mg/day) according to antibiogram results, and hydro-electrolyte rebalancing solutions. Intrainfectious severe anemia (HGB = 6.8 g/dL) imposed the erythrocyte mass administration. Evolution is favorable with remission of the diarrheal stools and the fever, together with correcting anemia (HGB = 13 g/dL at discharge).

**Conclusions**

Early detection of postoperative sepsis associated with infection with *C. difficile*, alongside effective therapeutic clinical monitoring can help increase life expectancy in elderly immunocompromised patients.

**Consent**

Written informed consent was obtained from the patient for publication of this Case report and any accompanying images. A copy of the written consent is available for review by the Editor of this journal.

### A116 Risk factors and their impact on psychopathology and quality of life among people living with HIV/AIDS in Romania

#### Fulvia Ursoiu, Mirandolina Prișcă, George Ciprian Pribac

##### “Vasile Goldiș” Western University, Arad, Romania

###### **Correspondence:** Fulvia Ursoiu (fulviacarm@yahoo.com)

**Background**

The aim of this study was to identify the risk factors and their impact on psychopathology and quality of life among people living with HIV/AIDS in Romania, in order of obtaining a risk psychological profile.

**Methods**

For this purpose, 85 HIV-positive patients (18–57 years, 45 men and 40 women) under antiretroviral therapy, were evaluated using psychometric clinical instruments: 5 scales, 3 questionnaires and 2 inventories for measuring depression, anxiety, psychosis, mania, posttraumatic stress, substance use disorders, personality dimensions of the big five model and the quality of life with the main domains: physical health, psychological health, level of independence, social relationships, environment, spirituality/religion/personal beliefs. We analyzed the following probable risk factors: age, sex, urban/rural location, level of education attained, marital status, serostatus (asymptomatic/symptomatic/AIDS converted), modes of HIV transmission and perception of health state.

**Results**

For every studied variable (psychopatological or quality of life indicators) a generalized linear model (GLM) was elaborated where we included the probable risk factors. Level of education attained, sex and marital status are important factors of risk for the state of health (both psysical and psychological) and quality of life among people living with HIV/AIDS. Level of education is significantly associated with depressive disorder (<0.001), anxiety disorders (0.019; 0.002; <0.001), post traumatic stress disorder (0.004), obsessive-compulsive disorder (0.011), substance use abuse (0.01), neuro-cognitive function (<0.001) and with quality of life indicators: health (0.04), psychological (<0.001), social relationships (0.001), environment (<0.001), and overall quality of life perception (0.007). Sex is strongly associated with global cognitive function (0.030), depressive disorder (0.011), substance use abuse (0.017), somatic symptom disorder (0.026), physical (0.009) and spirituality domain (0.01). Marital status is associated with global cognitive function (0.016), depressive disorder (0.005), anxiety disorders (0.012; 0.039; <0.001), obsessive-compulsive disorder (0.006), emotional stability (0.041) and psysical domain (0.036), social relationships (0.009) and overall quality of life perception (0.003). Age is a risk factor regarding cognitive decline (0.021; 0.042; 0.049), while the serostatus (asymptomatic/symptomatic/AIDS converted) is associated with depression (0.022), social phobia (0.003), substance use disorders (0.007), perceived stress (0.041) and general health condition (0.004). Subjects who perceived themselves as sick have a higher risk of substance abuse (0.004), isolation (0.024) and antisocial behaviour (0.048).

**Conclusions**

Our study identified a psychological risk profile of people living with HIV/AIDS in Romania and we concluded that every single risk factor should be addressed properly by a specialist multidisciplinary team.

### A117 Antivirals susceptibility of influenza viruses circulating in Romania

#### Maria Elena Mihai, Carmen Maria Cherciu, Alina Elena Ivanciuc, Cristina Tecu, Emilia Lupulescu

##### Cantacuzino National Institute for Research, Bucharest, Romania

###### **Correspondence:** Maria Elena Mihai (maria_elena_mihai@yahoo.com)

**Background**

The WHO Experts on Influenza Antiviral Susceptibility work closely to promote understanding of the means of prevention, treatment and control of influenza virus diseases, including the emergence of antiviral resistance. Due to the high prevalence of resistance to the adamantane class of drugs among influenza A(H3N2) and A(H1N1) 2009 pandemic strain, neuraminidase inhibitors (NAIs) are currently the only antivirals used in treatment of respiratory infections caused by influenza viruses and less in their prevention - in Romania. Because current pharmaceutical options for treating influenza infection and the opportunity of their use in prophylaxis therapy are limited, there remains a critical need for surveillance on NAI susceptibility of influenza viruses circulating worldwide and also in Romania. The National Influenza Center of the Cantacuzino Institute performs laboratory diagnosis and virological surveillance of influenza viruses, including antiviral susceptibility testing.

**Methods**

In the Cantacuzino Institute, antiviral susceptibility testing is performed mainly by phenotypic methods, only able to detect the degree of reduction of neuraminidase inhibition, respectively highly reduced inhibition (HRI) indicating a resistant influenza virus strain. The concentrations of the antiviral substance required to inhibit 50 % of the neuraminidase enzymatic activity (IC50) generated in this assay are used to evaluate the NAI-susceptibility. In the last epidemic season the use of fluorescent instead of chemiluminescent substrate was introduced in phenotypic assay, the method which led to a more reliable analysis, functional and increasing the number of samples tested. The viruses strains with significantly elevated IC50s are further analyzed by conventional sequencing to identify known markers of NAI resistance or novel changes in the NA.

**Results**

During the epidemic seasons of years 2013–2016 164 strains of influenza virus were tested phenotypically (type A subtype H1N1 or H3N2 and type B). With the exception of two strains which exhibited IC50 values at the upper limit of normal inhibitions, all were within the sensitive criteria to oseltamivir and zanamivir.

**Conclusions**

Due to the implementation of sensitive and functional testing for antiviral susceptibility of influenza viruses circulating during the last 3 epidemic seasons, the number of samples tested in Romania has been increasing. Recorded values are in the baseline of the characteristic strains, still being sensitive to antivirals. Continuous monitoring of antiviral susceptibility is needed to allow early detection of resistant strains.

### A118 Retrospective study of hospitalized cases of sepsis at the Hospital Clinic of Infectious Diseases “Toma Ciorbă”

#### Irina Bunescu^1^, Tiberiu Holban^1^, Ana Pasnin^2^, Stela Semeniuc^1^, Raisa Popovici^3^, Galina Chiriacov^3^

##### ^1^Department of Infectious Diseases and Medical Parasitology, Medical and Pharmaceutical University “Nicolae Testemițanu”, Chișinău, Republic of Moldova; ^2^Department of Laboratory Medicine, Medical and Pharmaceutical University “Nicolae Testemițanu”, Chișinău, Republic of Moldova; ^3^Republican Clinical Hospital of Infectious Diseases “Toma Ciorbă”, Chișinău, Republic of Moldova

###### **Correspondence:** Irina Bunescu (bunescu.irina@yahoo.com)

**Background**

Sepsis remains a major problem of modern medicine despite considerable medical advances in recent decades. The undeniable relevance of this condition is explained by the increasing incidence, high mortality, and high economic costs related to treatment. More than 750,000 cases of sepsis occur annually in the United States and this number has been increasing over the years, and the cost of treatment is estimated to be 17 billion USD per year. In Europe the number of sepsis cases annually recorded is approximately 500,000 cases, and according to the data reported by several authors the number of fatalities would vary between 30 % and 90 %.

**Methods**

We conducted a retrospective study of hospital records of 15 patients admitted to the Infectious Diseases Hospital “T. Ciorbă” with the diagnosis of sepsis. Data elements assessed included demographic information, physiological variables, comorbidities, laboratory measurements, suspected source of infection, and administered treatment.

**Results**

A total of 15 patients were admitted. The most affected age range was 55–64 years and males were more commonly affected than women. The diagnosis of referral was as follows: 4 patients have been directed with sepsis, 2 patients came with acute or chronic hepatitis, 1 with acute respiratory infection, 1 with salmonellosis, and 7 patients had no diagnosis of reference. At admission the diagnosis of sepsis was established in 5 patients, fever of unknown origin in 4 patients, enteroviral infection in 2 patients, and in other cases the diagnosis of acute hepatitis, influenza, food poisoning and yersiniosis was established. The identification of the primary source of infection was possible in 8 patients: infectious endocarditis 2 cases, urogenital sepsis 2 cases, HIV + tuberculosis 1 case, generalized form of salmonellosis 1 case, 1 case of lung infection (medial-lobe pneumonia), and 1 case of skin infection. Identification of etiologic agent was possible in 10 patients by bacteriological examinations. The causative agents most frequently found were the species of *Staphylococcus*: *Staphylococcus aureus* - 1 case, *Staphylococcus epidermidis* - 3 cases, *Staphylococcus hemolyticus* - 4 cases, 1 case of *Salmonella enteritidis*, *Mycobacterium tuberculosis* - 1 case. The most commonly used antibiotics were generation III and IV cephalosporins, alone or associated with other antibiotics: metronidazole, fluoroquinolones, aminoglycosides, and in 2 cases vancomycin ± 1 aminoglycoside was administered.

**Conclusions**

The most common pathogens identified in the etiology of sepsis were the species of *Staphylococcus*, which corresponds to some literature data.

